# Supplementary material for: Mitochondrial outer membrane integrity regulates a ubiquitin-dependent and NF-κB-mediated inflammatory response
Source: EMBO J. 2024 Feb 9;43(6):904–30. doi: 10.1038/s44318-024-00044-1 (PMC10943237; doi:10.1038/s44318-024-00044-1)
Supplement: Supplementary file 1 — Appendix [file 44318_2024_44_MOESM1_ESM.pdf]

Table of Contents

Appendix Table S1

Intramitochondrial localisation of proteins found to be ubiquitylated upon MOMP page 1

Appendix Table S1

Gene names	Student's T-test Significant	Localization	Source
Acad11	Ubiquitylated upon MOMP	Inner Mitochondrial Membrane	Mitocarta3.0
Acot9;Acot10	Ubiquitylated upon MOMP	Inner Mitochondrial Membrane	Mitocarta3.0
Agk	Ubiquitylated upon MOMP	Inner Mitochondrial Membrane	Mitocarta3.0
Agpat5	Ubiquitylated upon MOMP	Outer Mitochondrial Membrane	Mitocarta3.0
Aifm1	Ubiquitylated upon MOMP	Inner Mitochondrial Membrane	Mitocarta3.0
Ak2	Ubiquitylated upon MOMP	Mitochondrial Intermembrane Space	Mitocarta3.0
Akap1	Ubiquitylated upon MOMP	Outer Mitochondrial Membrane	Mitocarta3.0
Apool	Ubiquitylated upon MOMP	Inner Mitochondrial Membrane	Mitocarta3.0
Armc10	Ubiquitylated upon MOMP	Outer Mitochondrial Membrane	Mitocarta3.0
Armcx3	Ubiquitylated upon MOMP	Outer Mitochondrial Membrane	Mitocarta3.0
Atad1	Ubiquitylated upon MOMP	Outer Mitochondrial Membrane	Mitocarta3.0
Atad3	Ubiquitylated upon MOMP	Inner Mitochondrial Membrane	Mitocarta3.0
Atp5b	Ubiquitylated upon MOMP	Inner Mitochondrial Membrane	Mitocarta3.0
Atp5i	Ubiquitylated upon MOMP	Inner Mitochondrial Membrane	Mitocarta3.0
Bak1	Ubiquitylated upon MOMP	Outer Mitochondrial Membrane	Mitocarta3.0
Bax	Ubiquitylated upon MOMP	Outer Mitochondrial Membrane	Mitocarta3.0
Bcl2l1	Ubiquitylated upon MOMP	Outer Mitochondrial Membrane	Mitocarta3.0
Bcl2l13	Ubiquitylated upon MOMP	Outer Mitochondrial Membrane	Mitocarta3.0
Bnip3l	Ubiquitylated upon MOMP	Outer Mitochondrial Membrane	Mitocarta3.0
Card19	Ubiquitylated upon MOMP	Unknown mitochondrial localisation	Uniprot
Ccdc127	Ubiquitylated upon MOMP	Unknown mitochondrial localisation	Mitocarta3.0
Ccdc51	Ubiquitylated upon MOMP	Inner Mitochondrial Membrane	Mitocarta3.0
Ccdc58	Ubiquitylated upon MOMP	Mitochondrial Intermembrane Space	Mitocarta3.0
Chchd3	Ubiquitylated upon MOMP	Inner Mitochondrial Membrane	Mitocarta3.0
Chchd4	Ubiquitylated upon MOMP	Mitochondrial Intermembrane Space	Mitocarta3.0
Chchd6	Ubiquitylated upon MOMP	Inner Mitochondrial Membrane	Mitocarta3.0
Cisd1	Ubiquitylated upon MOMP	Outer Mitochondrial Membrane	Mitocarta3.0
Coa3	Ubiquitylated upon MOMP	Inner Mitochondrial Membrane	Mitocarta3.0
Comtd1	Ubiquitylated upon MOMP	Unknown mitochondrial localisation	Mitocarta3.0
Cox11	Ubiquitylated upon MOMP	Inner Mitochondrial Membrane	Mitocarta3.0
Cox14	Ubiquitylated upon MOMP	Inner Mitochondrial Membrane	Mitocarta3.0
Cox15	Ubiquitylated upon MOMP	Inner Mitochondrial Membrane	Mitocarta3.0
Cox17	Ubiquitylated upon MOMP	Mitochondrial Intermembrane Space	Mitocarta3.0
Cox20	Ubiquitylated upon MOMP	Inner Mitochondrial Membrane	Mitocarta3.0
Cox4i1	Ubiquitylated upon MOMP	Inner Mitochondrial Membrane	Mitocarta3.0
Cox6b1	Ubiquitylated upon MOMP	Inner Mitochondrial Membrane	Mitocarta3.0
Cox6c	Ubiquitylated upon MOMP	Inner Mitochondrial Membrane	Mitocarta3.0
Cox7a2	Ubiquitylated upon MOMP	Inner Mitochondrial Membrane	Mitocarta3.0
Cox7b	Ubiquitylated upon MOMP	Inner Mitochondrial Membrane	Mitocarta3.0
Cpt1a	Ubiquitylated upon MOMP	Outer Mitochondrial Membrane	Mitocarta3.0
Cs	Ubiquitylated upon MOMP	Mitochondrial matrix	Mitocarta3.0
Cyb5b	Ubiquitylated upon MOMP	Outer Mitochondrial Membrane	Mitocarta3.0
Cyb5r1	Ubiquitylated upon MOMP	Unknown mitochondrial localisation	Uniprot
Cyb5r3	Ubiquitylated upon MOMP	Outer Mitochondrial Membrane	Mitocarta3.0
Cyc1	Ubiquitylated upon MOMP	Inner Mitochondrial Membrane	Mitocarta3.0

Dcakd	Ubiquitylated upon MOMP	Unknown mitochondrial localisation	Mitocarta3.0
Dhfr	Ubiquitylated upon MOMP	Unknown mitochondrial localisation	Uniprot
Dhodh	Ubiquitylated upon MOMP	Inner Mitochondrial Membrane	Mitocarta3.0
Diablo	Ubiquitylated upon MOMP	Mitochondrial Intermembrane Space	Mitocarta3.0
Dlat	Ubiquitylated upon MOMP	Mitochondrial matrix	Mitocarta3.0
Dnajc11	Ubiquitylated upon MOMP	Outer Mitochondrial Membrane	Mitocarta3.0
Exog	Ubiquitylated upon MOMP	Inner Mitochondrial Membrane	Mitocarta3.0
Fam162a	Ubiquitylated upon MOMP	Inner Mitochondrial Membrane	Mitocarta3.0
Fam73b	Ubiquitylated upon MOMP	Outer Mitochondrial Membrane	Mitocarta3.0
Fis1	Ubiquitylated upon MOMP	Outer Mitochondrial Membrane	Mitocarta3.0
Fkbp8	Ubiquitylated upon MOMP	Outer Mitochondrial Membrane	Mitocarta3.0
Fundc2	Ubiquitylated upon MOMP	Outer Mitochondrial Membrane	Mitocarta3.0
Ghitm	Ubiquitylated upon MOMP	Inner Mitochondrial Membrane	Mitocarta3.0
Glrx5	Ubiquitylated upon MOMP	Mitochondrial matrix	Mitocarta3.0
Glud1	Ubiquitylated upon MOMP	Mitochondrial matrix	Mitocarta3.0
Gpam	Ubiquitylated upon MOMP	Unknown mitochondrial localisation	Mitocarta3.0
Gpd2	Ubiquitylated upon MOMP	Inner Mitochondrial Membrane	Mitocarta3.0
Gramd4	Ubiquitylated upon MOMP	Unknown mitochondrial localisation	Uniprot
Gsr	Ubiquitylated upon MOMP	Mitochondrial matrix	Mitocarta3.0
Hccs	Ubiquitylated upon MOMP	Mitochondrial Intermembrane Space	Mitocarta3.0
Hk1	Ubiquitylated upon MOMP	Unknown mitochondrial localisation	Uniprot
Hk2	Ubiquitylated upon MOMP	Unknown mitochondrial localisation	Uniprot
Hsdl1	Ubiquitylated upon MOMP	Unknown mitochondrial localisation	Mitocarta3.0
Hspd1	Ubiquitylated upon MOMP	Unknown mitochondrial localisation	Mitocarta3.0
Htra2	Ubiquitylated upon MOMP	Inner Mitochondrial Membrane	Mitocarta3.0
Immt	Ubiquitylated upon MOMP	Inner Mitochondrial Membrane	Mitocarta3.0
Letm1	Ubiquitylated upon MOMP	Inner Mitochondrial Membrane	Mitocarta3.0
Maoa	Ubiquitylated upon MOMP	Outer Mitochondrial Membrane	Mitocarta3.0
Marc2	Ubiquitylated upon MOMP	Outer Mitochondrial Membrane	Mitocarta3.0
March5	Ubiquitylated upon MOMP	Outer Mitochondrial Membrane	Mitocarta3.0
Mavs	Ubiquitylated upon MOMP	Outer Mitochondrial Membrane	Mitocarta3.0
Mcl1	Ubiquitylated upon MOMP	Outer Mitochondrial Membrane	Mitocarta3.0
Mdh2	Ubiquitylated upon MOMP	Mitochondrial matrix	Mitocarta3.0
Mff	Ubiquitylated upon MOMP	Outer Mitochondrial Membrane	Mitocarta3.0
Mfn1	Ubiquitylated upon MOMP	Outer Mitochondrial Membrane	Mitocarta3.0
Mfn2	Ubiquitylated upon MOMP	Outer Mitochondrial Membrane	Mitocarta3.0
Mgst1	Ubiquitylated upon MOMP	Unknown mitochondrial localisation	Mitocarta3.0
Micu1	Ubiquitylated upon MOMP	Inner Mitochondrial Membrane	Mitocarta3.0
Micu2	Ubiquitylated upon MOMP	Inner Mitochondrial Membrane	Mitocarta3.0
Mp68	Ubiquitylated upon MOMP	Inner Mitochondrial Membrane	Mitocarta3.0
Mpc1	Ubiquitylated upon MOMP	Inner Mitochondrial Membrane	Mitocarta3.0
Mtch1	Ubiquitylated upon MOMP	Outer Mitochondrial Membrane	Mitocarta3.0
Mtch2	Ubiquitylated upon MOMP	Outer Mitochondrial Membrane	Mitocarta3.0
Mtco2	Ubiquitylated upon MOMP	Unknown mitochondrial localisation	Uniprot
Mtfr1l	Ubiquitylated upon MOMP	Unknown mitochondrial localisation	Mitocarta3.0
Mtx1	Ubiquitylated upon MOMP	Outer Mitochondrial Membrane	Mitocarta3.0
Mtx2	Ubiquitylated upon MOMP	Outer Mitochondrial Membrane	Mitocarta3.0
Mul1	Ubiquitylated upon MOMP	Outer Mitochondrial Membrane	Mitocarta3.0

Ndufa3	Ubiquitylated upon MOMP	Inner Mitochondrial Membrane	Mitocarta3.0
Ndufa4	Ubiquitylated upon MOMP	Inner Mitochondrial Membrane	Mitocarta3.0
Ndufa8	Ubiquitylated upon MOMP	Inner Mitochondrial Membrane	Mitocarta3.0
Ndufb1	Ubiquitylated upon MOMP	Inner Mitochondrial Membrane	Mitocarta3.0
Ndufb10	Ubiquitylated upon MOMP	Inner Mitochondrial Membrane	Mitocarta3.0
Ndufb5	Ubiquitylated upon MOMP	Inner Mitochondrial Membrane	Mitocarta3.0
Ndufc2	Ubiquitylated upon MOMP	Inner Mitochondrial Membrane	Mitocarta3.0
Ndufs5	Ubiquitylated upon MOMP	Inner Mitochondrial Membrane	Mitocarta3.0
Ociad1	Ubiquitylated upon MOMP	Outer Mitochondrial Membrane	Mitocarta3.0
Parl	Ubiquitylated upon MOMP	Inner Mitochondrial Membrane	Mitocarta3.0
Pet117	Ubiquitylated upon MOMP	Inner Mitochondrial Membrane	Mitocarta3.0
Pgam5	Ubiquitylated upon MOMP	Outer Mitochondrial Membrane	Mitocarta3.0
Phb	Ubiquitylated upon MOMP	Inner Mitochondrial Membrane	Mitocarta3.0
Phb2	Ubiquitylated upon MOMP	Inner Mitochondrial Membrane	Mitocarta3.0
Plgrkt	Ubiquitylated upon MOMP	Unknown mitochondrial localisation	Mitocarta3.0
Ppif	Ubiquitylated upon MOMP	Mitochondrial matrix	Mitocarta3.0
Prdx3	Ubiquitylated upon MOMP	Mitochondrial matrix	Mitocarta3.0
Ptges2	Ubiquitylated upon MOMP	Inner Mitochondrial Membrane	Mitocarta3.0
Ptrh2	Ubiquitylated upon MOMP	Outer Mitochondrial Membrane	Mitocarta3.0
Rdh13	Ubiquitylated upon MOMP	Inner Mitochondrial Membrane	Mitocarta3.0
Rhot1	Ubiquitylated upon MOMP	Outer Mitochondrial Membrane	Mitocarta3.0
Rhot2	Ubiquitylated upon MOMP	Outer Mitochondrial Membrane	Mitocarta3.0
Samm50	Ubiquitylated upon MOMP	Outer Mitochondrial Membrane	Mitocarta3.0
Sco1	Ubiquitylated upon MOMP	Inner Mitochondrial Membrane	Mitocarta3.0
Scp2	Ubiquitylated upon MOMP	Inner Mitochondrial Membrane	Mitocarta3.0
Sdha	Ubiquitylated upon MOMP	Inner Mitochondrial Membrane	Mitocarta3.0
Sfxn1	Ubiquitylated upon MOMP	Inner Mitochondrial Membrane	Mitocarta3.0
Sfxn2	Ubiquitylated upon MOMP	Inner Mitochondrial Membrane	Mitocarta3.0
Shmt2	Ubiquitylated upon MOMP	Mitochondrial matrix	Mitocarta3.0
Slc25a10	Ubiquitylated upon MOMP	Inner Mitochondrial Membrane	Mitocarta3.0
Slc25a12	Ubiquitylated upon MOMP	Inner Mitochondrial Membrane	Mitocarta3.0
Slc25a13	Ubiquitylated upon MOMP	Inner Mitochondrial Membrane	Mitocarta3.0
Slc25a20	Ubiquitylated upon MOMP	Inner Mitochondrial Membrane	Mitocarta3.0
Slc25a22	Ubiquitylated upon MOMP	Inner Mitochondrial Membrane	Mitocarta3.0
Slc25a24	Ubiquitylated upon MOMP	Inner Mitochondrial Membrane	Mitocarta3.0
Slc25a25	Ubiquitylated upon MOMP	Inner Mitochondrial Membrane	Mitocarta3.0
Slc25a3	Ubiquitylated upon MOMP	Inner Mitochondrial Membrane	Mitocarta3.0
Slc25a4	Ubiquitylated upon MOMP	Inner Mitochondrial Membrane	Mitocarta3.0
Slc25a40	Ubiquitylated upon MOMP	Inner Mitochondrial Membrane	Mitocarta3.0
Slc25a46	Ubiquitylated upon MOMP	Outer Mitochondrial Membrane	Mitocarta3.0
Slc25a5	Ubiquitylated upon MOMP	Inner Mitochondrial Membrane	Mitocarta3.0
Smim37	Ubiquitylated upon MOMP	Unknown mitochondrial localisation	Uniprot
Stmp1	Ubiquitylated upon MOMP	Unknown mitochondrial localisation	Uniprot
Surf1	Ubiquitylated upon MOMP	Inner Mitochondrial Membrane	Mitocarta3.0
Synj2bp	Ubiquitylated upon MOMP	Outer Mitochondrial Membrane	Mitocarta3.0
Tdrkh	Ubiquitylated upon MOMP	Unknown mitochondrial localisation	Mitocarta3.0
TIMM10	Ubiquitylated upon MOMP	Inner Mitochondrial Membrane	Mitocarta3.0
TIMM13	Ubiquitylated upon MOMP	Inner Mitochondrial Membrane	Mitocarta3.0

TIMM21	Ubiquitylated upon MOMP	Inner Mitochondrial Membrane	Mitocarta3.0
TIMM22	Ubiquitylated upon MOMP	Inner Mitochondrial Membrane	Mitocarta3.0
TIMM23	Ubiquitylated upon MOMP	Inner Mitochondrial Membrane	Mitocarta3.0
TIMM50	Ubiquitylated upon MOMP	Inner Mitochondrial Membrane	Mitocarta3.0
TIMM8a1	Ubiquitylated upon MOMP	Inner Mitochondrial Membrane	Mitocarta3.0
TIMM8b	Ubiquitylated upon MOMP	Inner Mitochondrial Membrane	Mitocarta3.0
TIMM9	Ubiquitylated upon MOMP	Inner Mitochondrial Membrane	Mitocarta3.0
Tmem126a	Ubiquitylated upon MOMP	Inner Mitochondrial Membrane	Mitocarta3.0
Tmem14c	Ubiquitylated upon MOMP	Inner Mitochondrial Membrane	Mitocarta3.0
TOMM20	Ubiquitylated upon MOMP	Outer Mitochondrial Membrane	Mitocarta3.0
TOMM22	Ubiquitylated upon MOMP	Outer Mitochondrial Membrane	Mitocarta3.0
TOMM40	Ubiquitylated upon MOMP	Outer Mitochondrial Membrane	Mitocarta3.0
TOMM5	Ubiquitylated upon MOMP	Outer Mitochondrial Membrane	Mitocarta3.0
TOMM7	Ubiquitylated upon MOMP	Outer Mitochondrial Membrane	Mitocarta3.0
TOMM70a	Ubiquitylated upon MOMP	Outer Mitochondrial Membrane	Mitocarta3.0
Trabd	Ubiquitylated upon MOMP	Unknown mitochondrial localisation	Proteinatlas
Tspo	Ubiquitylated upon MOMP	Outer Mitochondrial Membrane	Mitocarta3.0
Uqcc3	Ubiquitylated upon MOMP	Inner Mitochondrial Membrane	Mitocarta3.0
Uqcrc2	Ubiquitylated upon MOMP	Inner Mitochondrial Membrane	Mitocarta3.0
Uqcrfs1	Ubiquitylated upon MOMP	Inner Mitochondrial Membrane	Mitocarta3.0
Uqcrh	Ubiquitylated upon MOMP	Inner Mitochondrial Membrane	Mitocarta3.0
Uqcrq	Ubiquitylated upon MOMP	Inner Mitochondrial Membrane	Mitocarta3.0
Usp30	Ubiquitylated upon MOMP	Outer Mitochondrial Membrane	Mitocarta3.0
Vdac1	Ubiquitylated upon MOMP	Outer Mitochondrial Membrane	Mitocarta3.0
Vdac2	Ubiquitylated upon MOMP	Outer Mitochondrial Membrane	Mitocarta3.0
Vdac3	Ubiquitylated upon MOMP	Outer Mitochondrial Membrane	Mitocarta3.0
Xrcc6bp1	Ubiquitylated upon MOMP	Mitochondrial Intermembrane Space	Mitocarta3.0
Yme1l1	Ubiquitylated upon MOMP	Inner Mitochondrial Membrane	Mitocarta3.0
