# Supplementary material for: Mitochondrial outer membrane integrity regulates a ubiquitin-dependent and NF-κB-mediated inflammatory response
Source: EMBO J. 2024 Feb 9;43(6):904–30. doi: 10.1038/s44318-024-00044-1 (PMC10943237; doi:10.1038/s44318-024-00044-1)
Supplement: Supplementary file 3 — Movie EV2 [file 44318_2024_44_MOESM3_ESM.zip › Movie EV2/Movie EV2 Legend.docx]

**Movie EV2**

U2OS cells expressing GFP-NEMO were treated with ABT-737/S6/QVD and imaged over time using live-cell confocal microscopy. Scalebar is 20μM.
