# Supplementary figures and images for: Mitochondrial outer membrane integrity regulates a ubiquitin-dependent and NF-κB-mediated inflammatory response (part 1 of 2)
Source: EMBO J. 2024 Feb 9;43(6):904–30. doi: 10.1038/s44318-024-00044-1 (PMC10943237; doi:10.1038/s44318-024-00044-1)

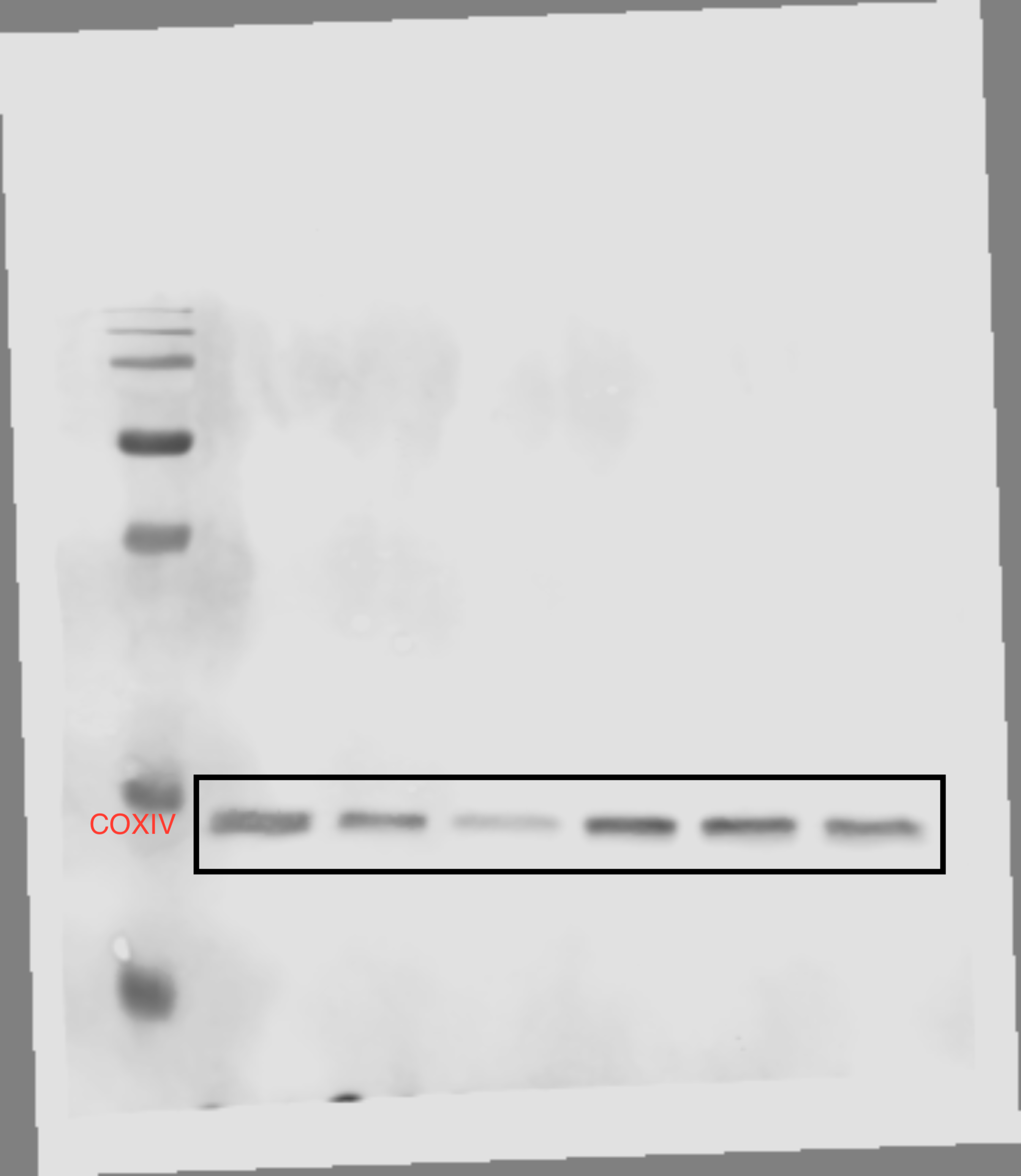

Supplement: Supplementary file 4 — Source Data Fig. 1 [file 44318_2024_44_MOESM4_ESM.zip › Fig 1/Fig 1A/Fig1A_BAX_BAK_COXIV.tif]

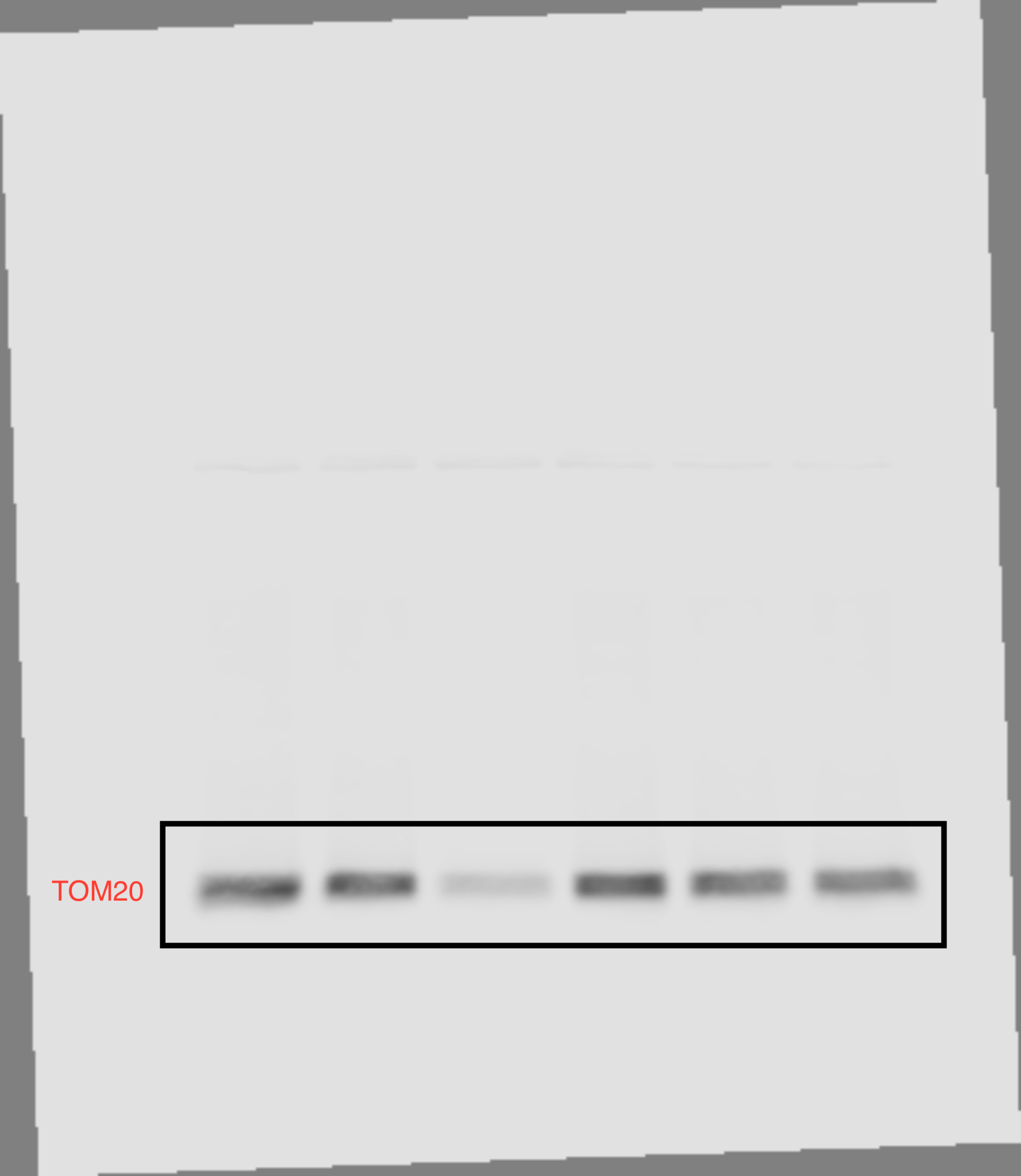

Supplement: Supplementary file 4 — Source Data Fig. 1 [file 44318_2024_44_MOESM4_ESM.zip › Fig 1/Fig 1A/Fig1A_BAX_BAK_COXIV_TOM20.tif]

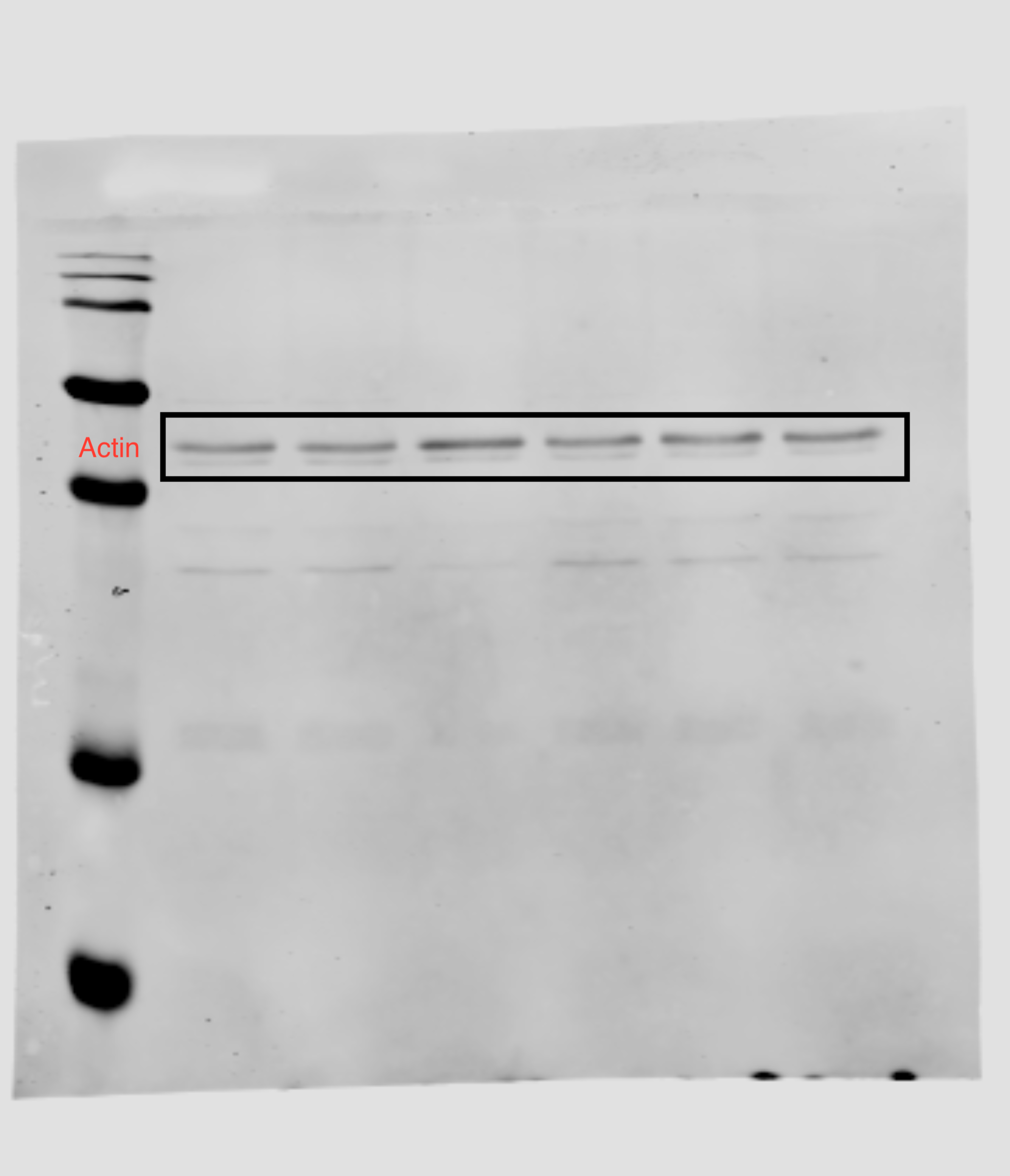

Supplement: Supplementary file 4 — Source Data Fig. 1 [file 44318_2024_44_MOESM4_ESM.zip › Fig 1/Fig 1A/Fig1A_Mitoprofile_actin.tif]

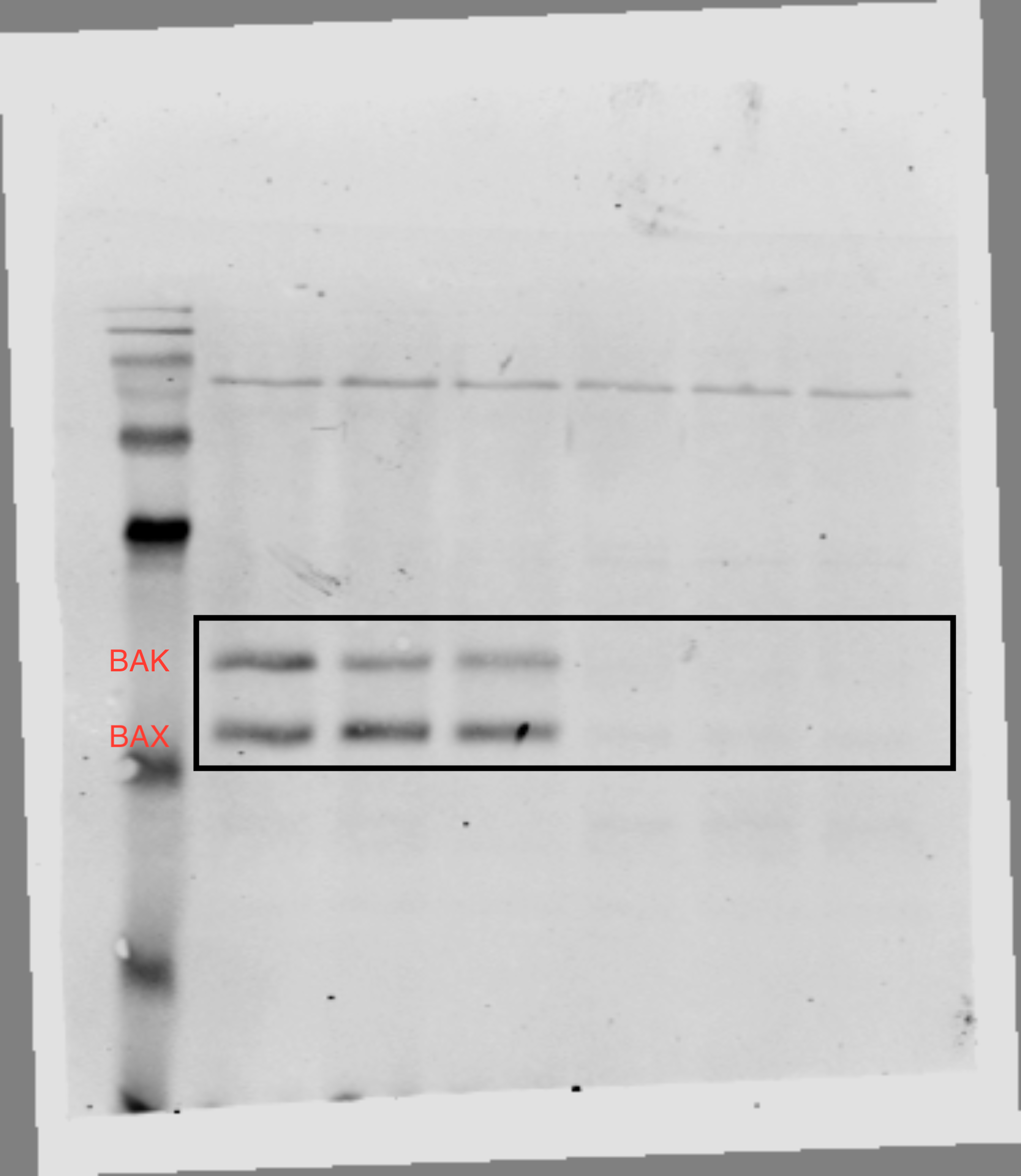

Supplement: Supplementary file 4 — Source Data Fig. 1 [file 44318_2024_44_MOESM4_ESM.zip › Fig 1/Fig 1A/Fig1A_BAX_BAK.tif]

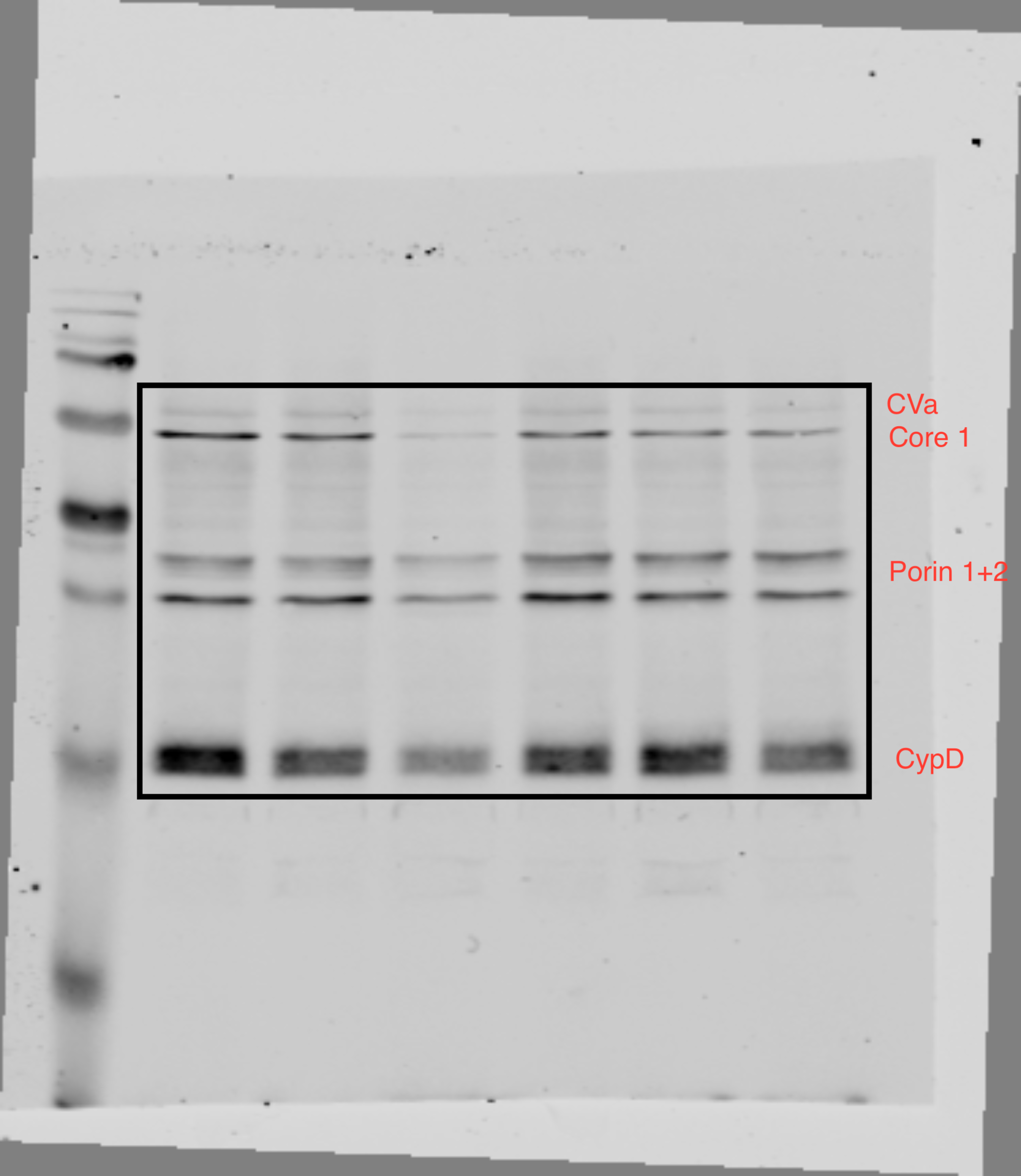

Supplement: Supplementary file 4 — Source Data Fig. 1 [file 44318_2024_44_MOESM4_ESM.zip › Fig 1/Fig 1A/Fig1A_Mitoprofile.tif]

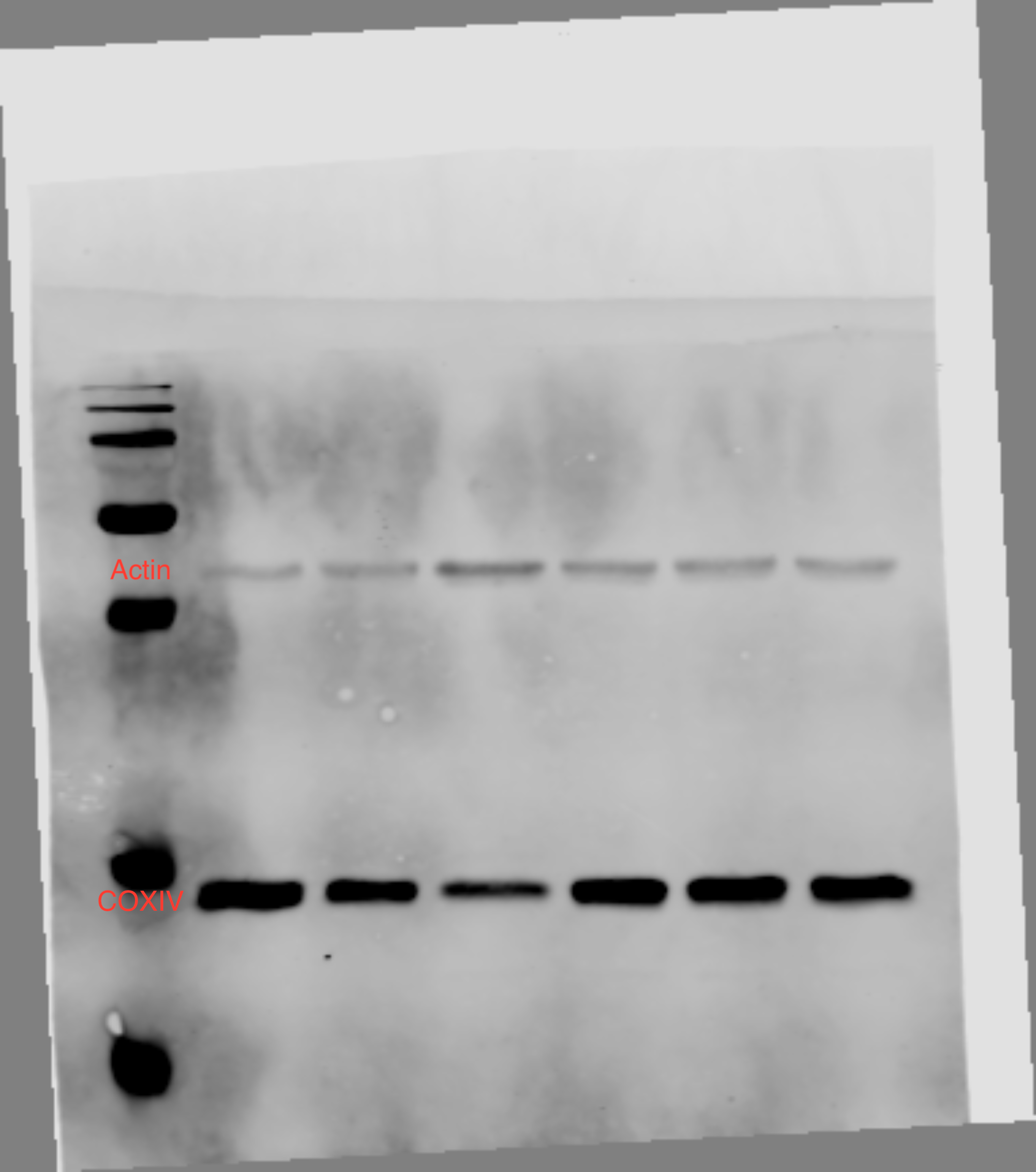

Supplement: Supplementary file 4 — Source Data Fig. 1 [file 44318_2024_44_MOESM4_ESM.zip › Fig 1/Fig 1A/Fig1A_BAX_BAK_COXIV_TOM20_actin.tif]

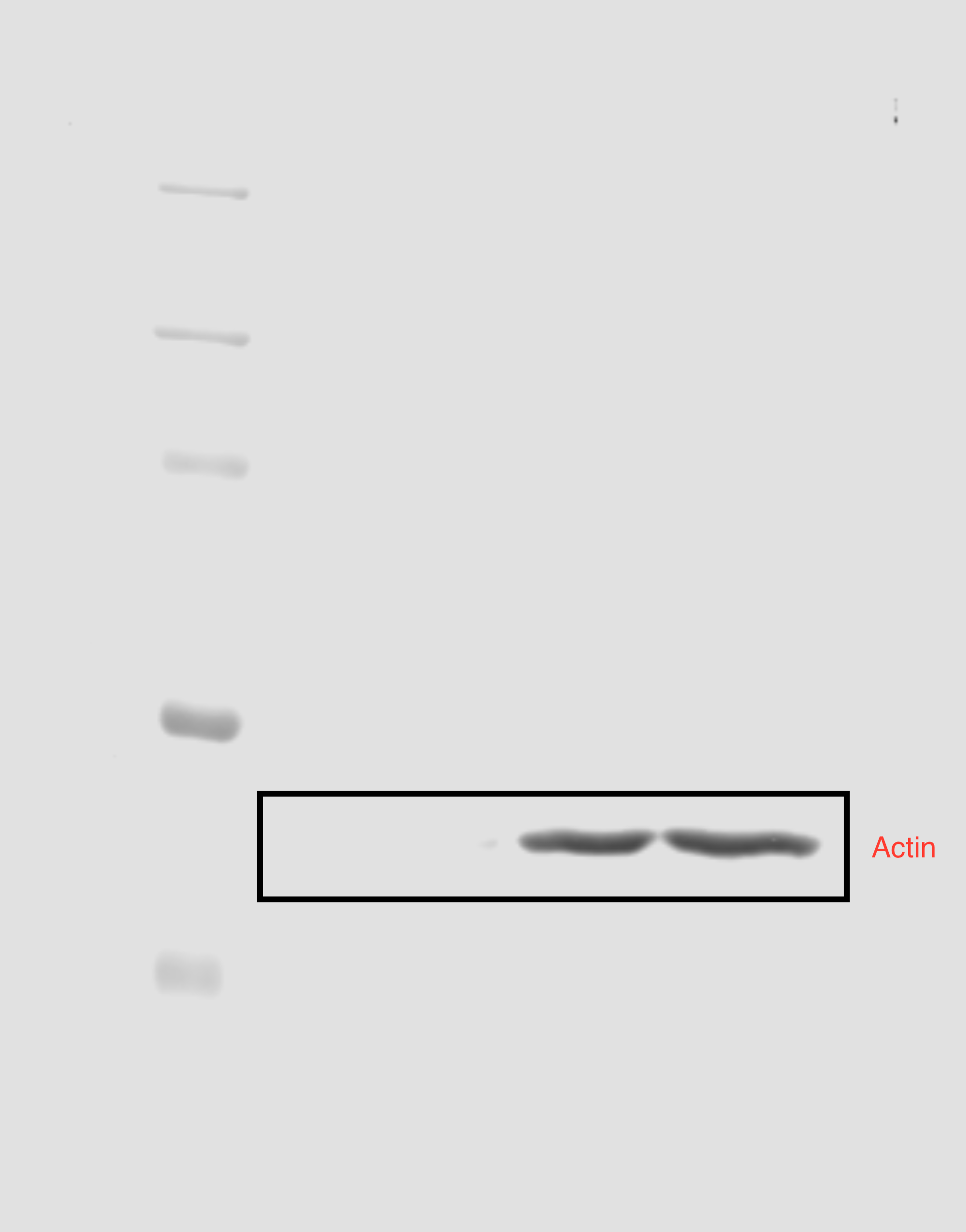

Supplement: Supplementary file 4 — Source Data Fig. 1 [file 44318_2024_44_MOESM4_ESM.zip › Fig 1/Fig 1D/Fig1D_UBCJ2_actin.tif]

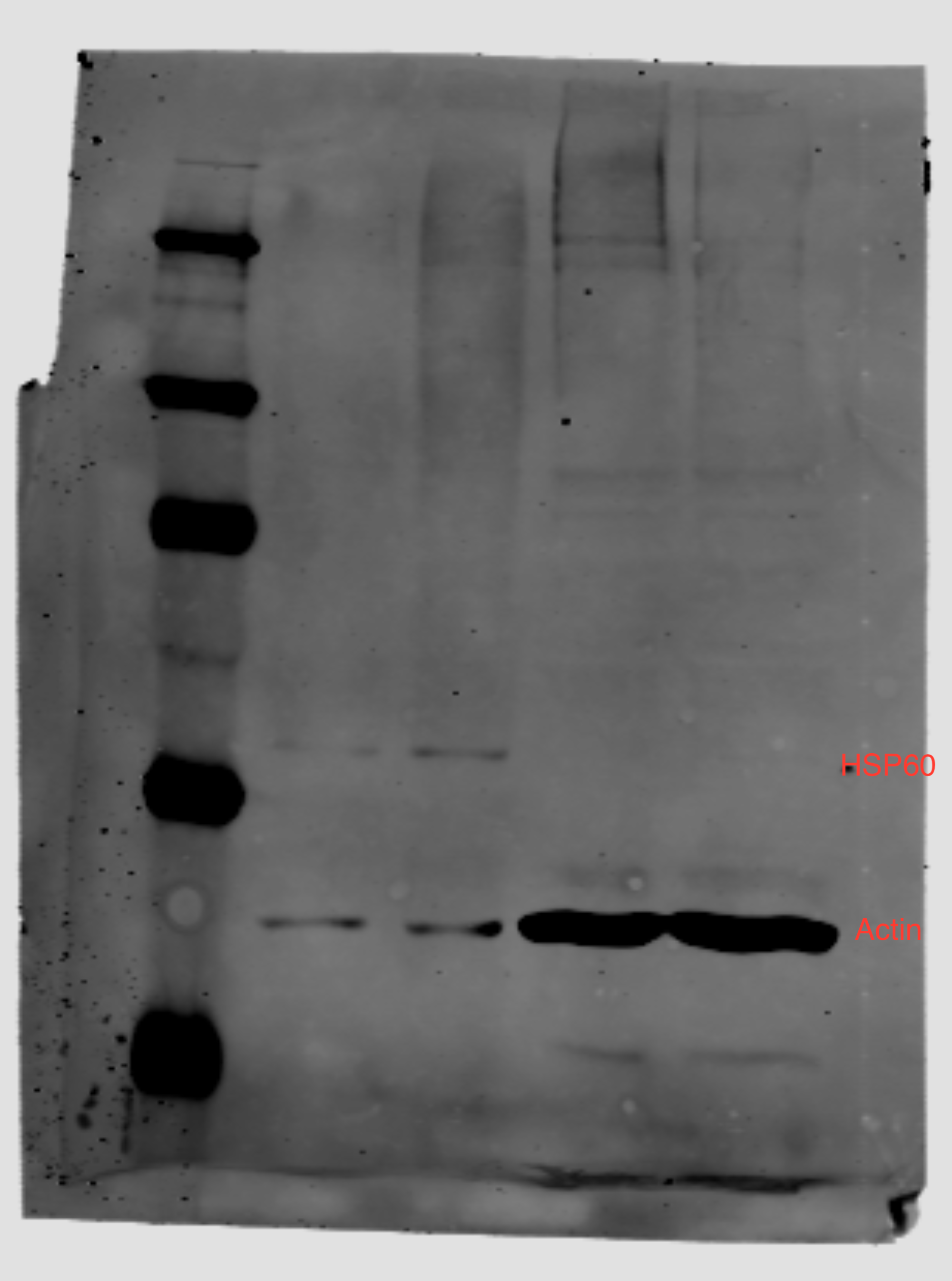

Supplement: Supplementary file 4 — Source Data Fig. 1 [file 44318_2024_44_MOESM4_ESM.zip › Fig 1/Fig 1D/Fig1D_UBCJ2_actin_hsp60.tif]

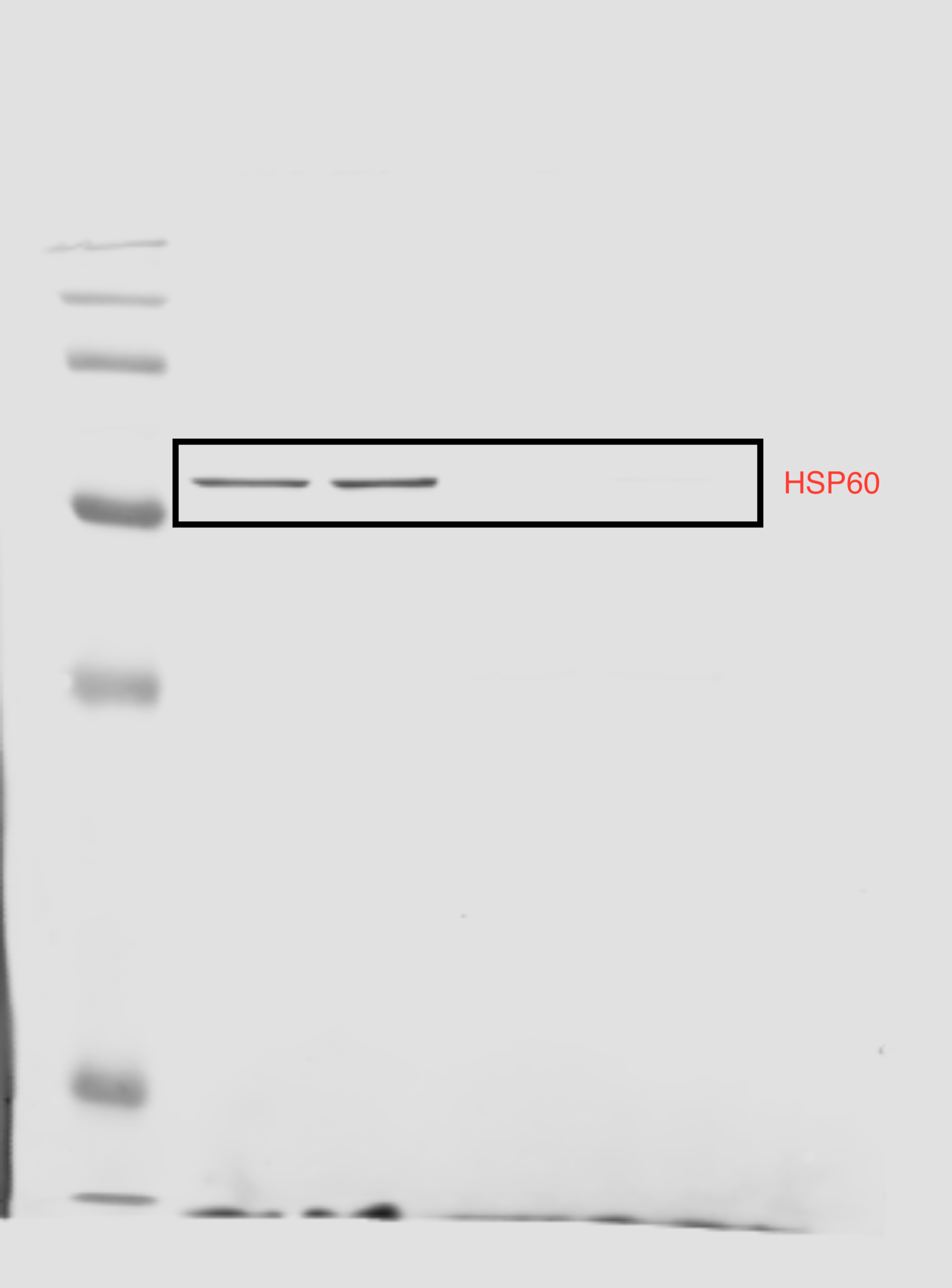

Supplement: Supplementary file 4 — Source Data Fig. 1 [file 44318_2024_44_MOESM4_ESM.zip › Fig 1/Fig 1D/Fig1D_TOM20_hsp60.tif]

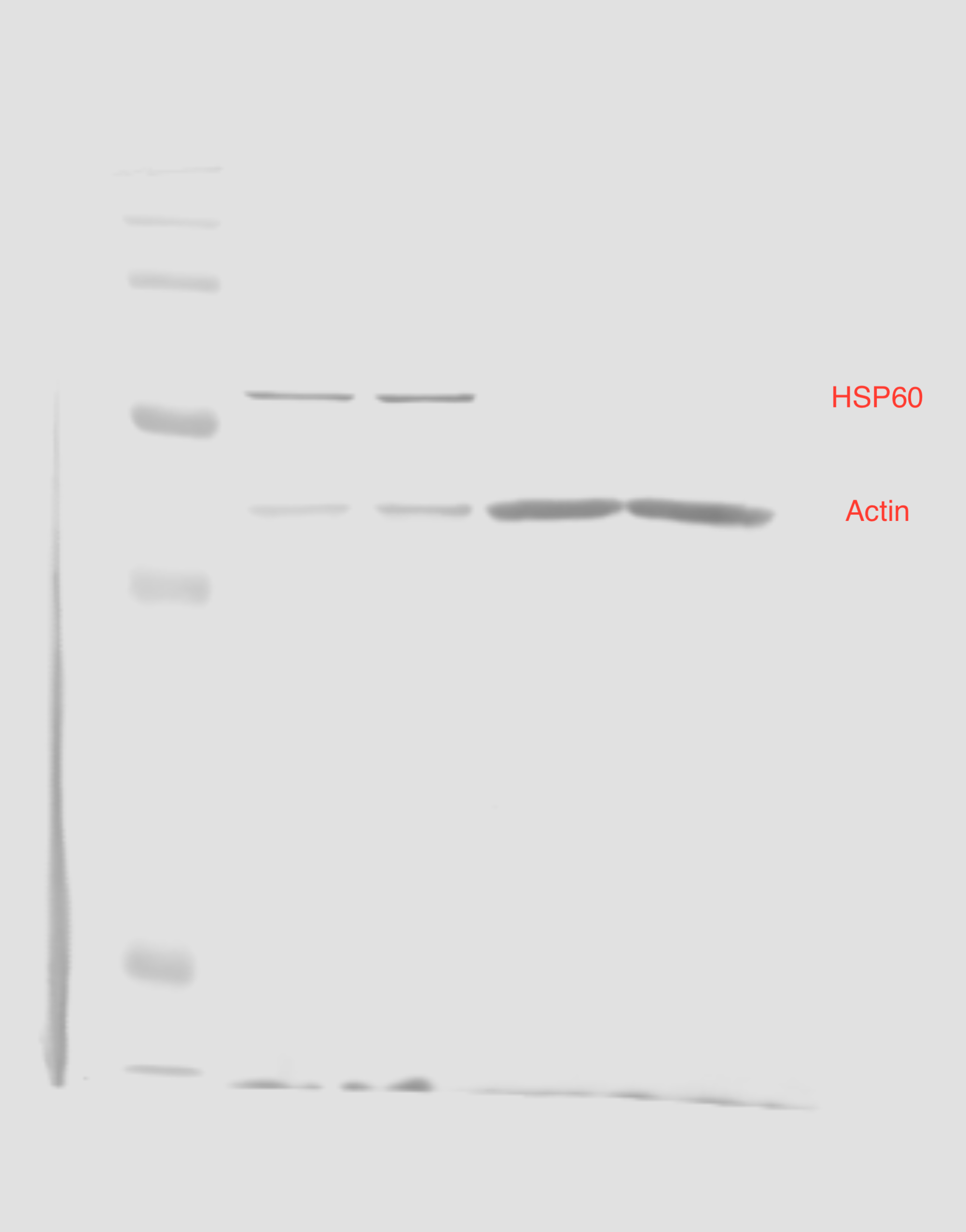

Supplement: Supplementary file 4 — Source Data Fig. 1 [file 44318_2024_44_MOESM4_ESM.zip › Fig 1/Fig 1D/Fig1D_TOM20_hsp60_actin.tif]

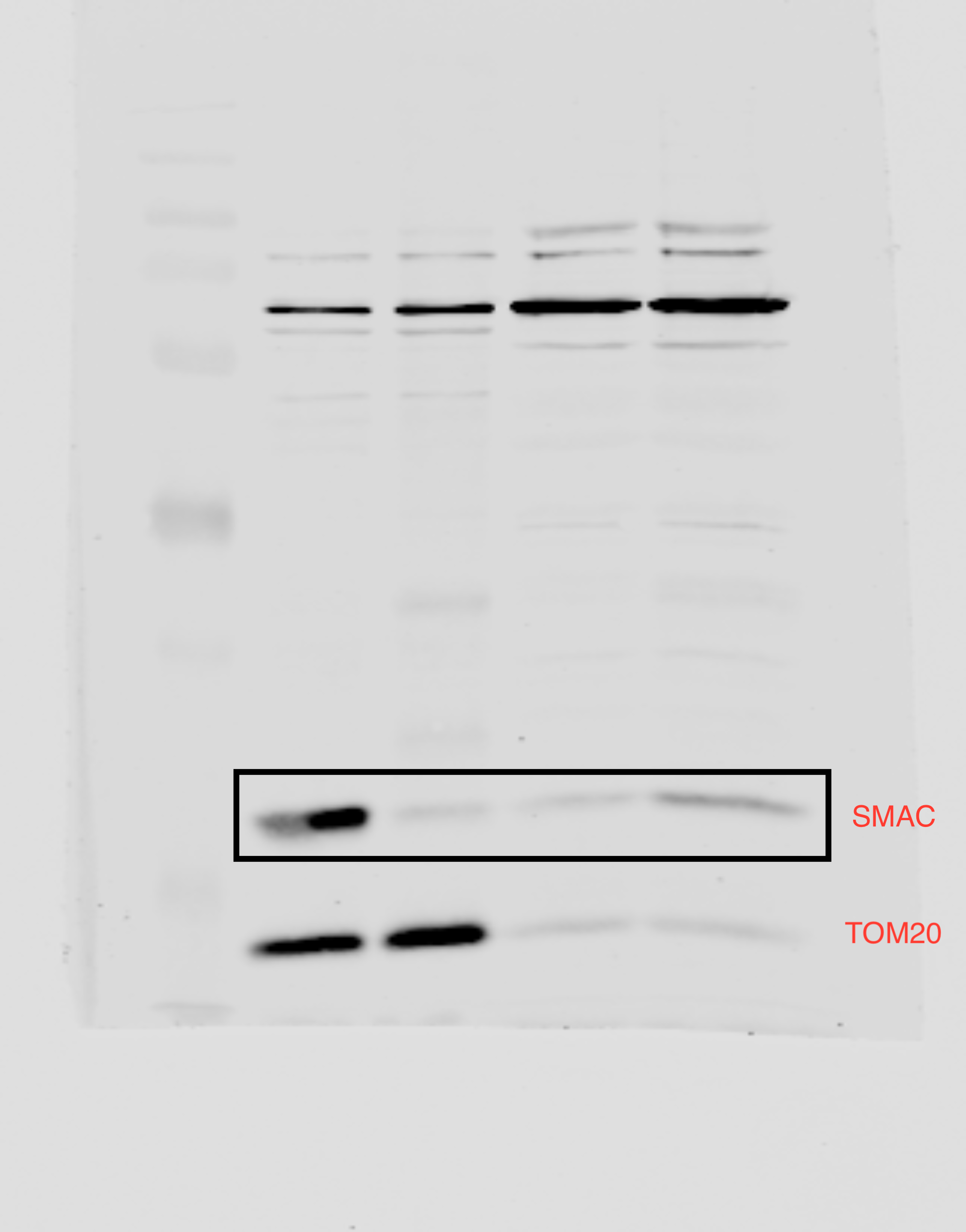

Supplement: Supplementary file 4 — Source Data Fig. 1 [file 44318_2024_44_MOESM4_ESM.zip › Fig 1/Fig 1D/Fig1D_TOM20_SMAC.tif]

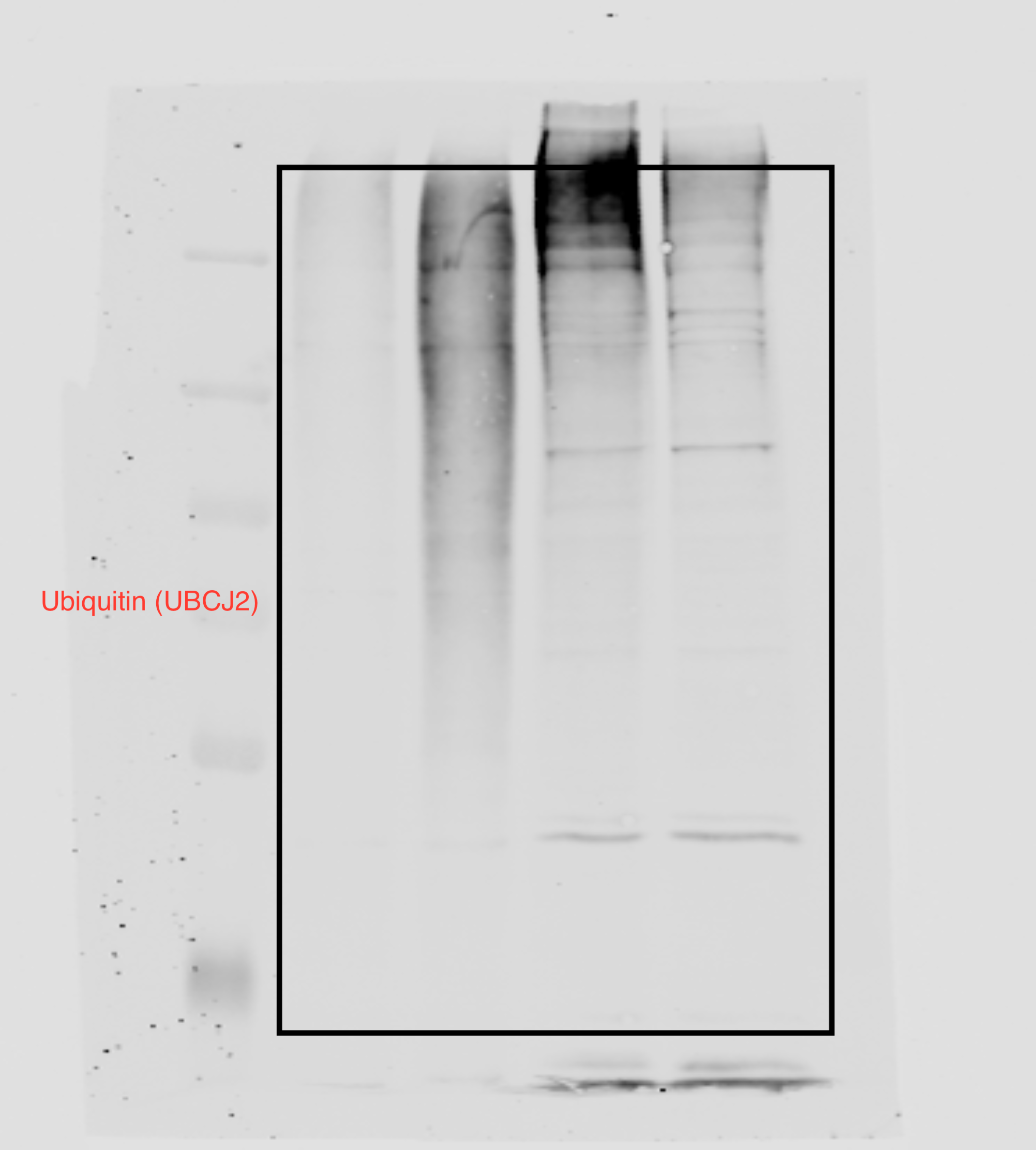

Supplement: Supplementary file 4 — Source Data Fig. 1 [file 44318_2024_44_MOESM4_ESM.zip › Fig 1/Fig 1D/Fig1D_UBCJ2.tif]

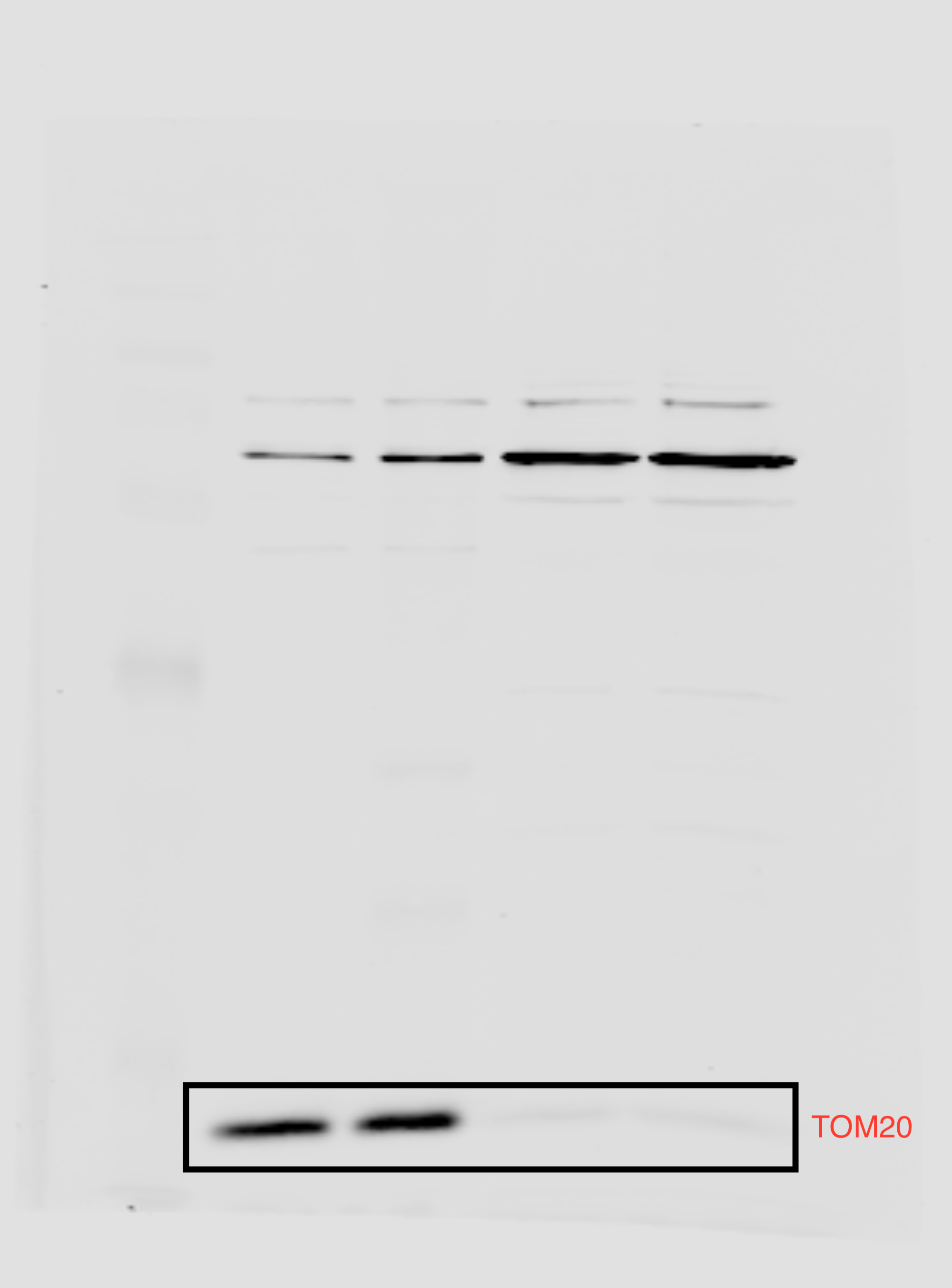

Supplement: Supplementary file 4 — Source Data Fig. 1 [file 44318_2024_44_MOESM4_ESM.zip › Fig 1/Fig 1D/FIG1D_TOM20.tif]

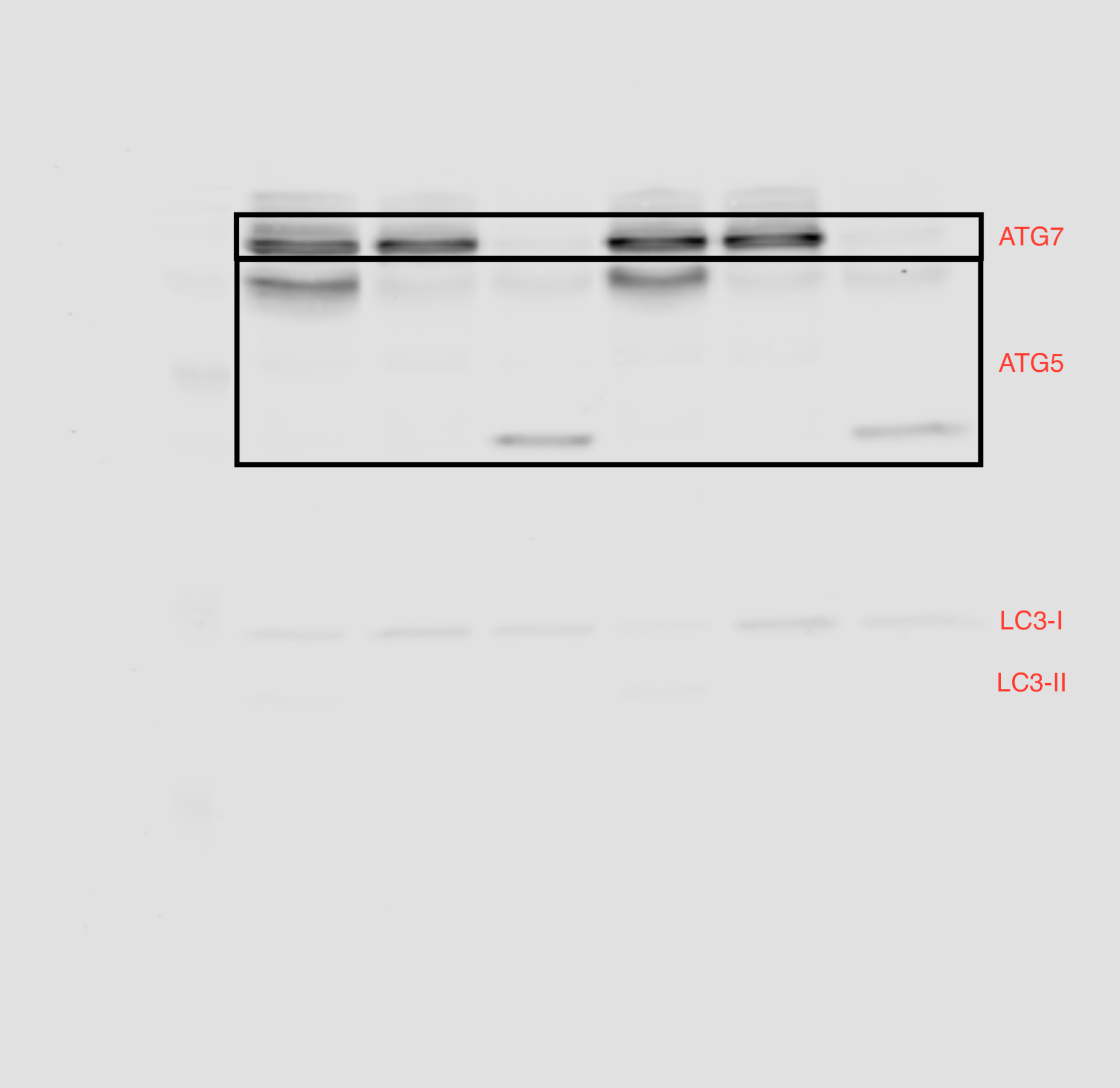

Supplement: Supplementary file 4 — Source Data Fig. 1 [file 44318_2024_44_MOESM4_ESM.zip › Fig 1/Fig 1G/Fig1G_LC3_ATG5-ATG7.tif]

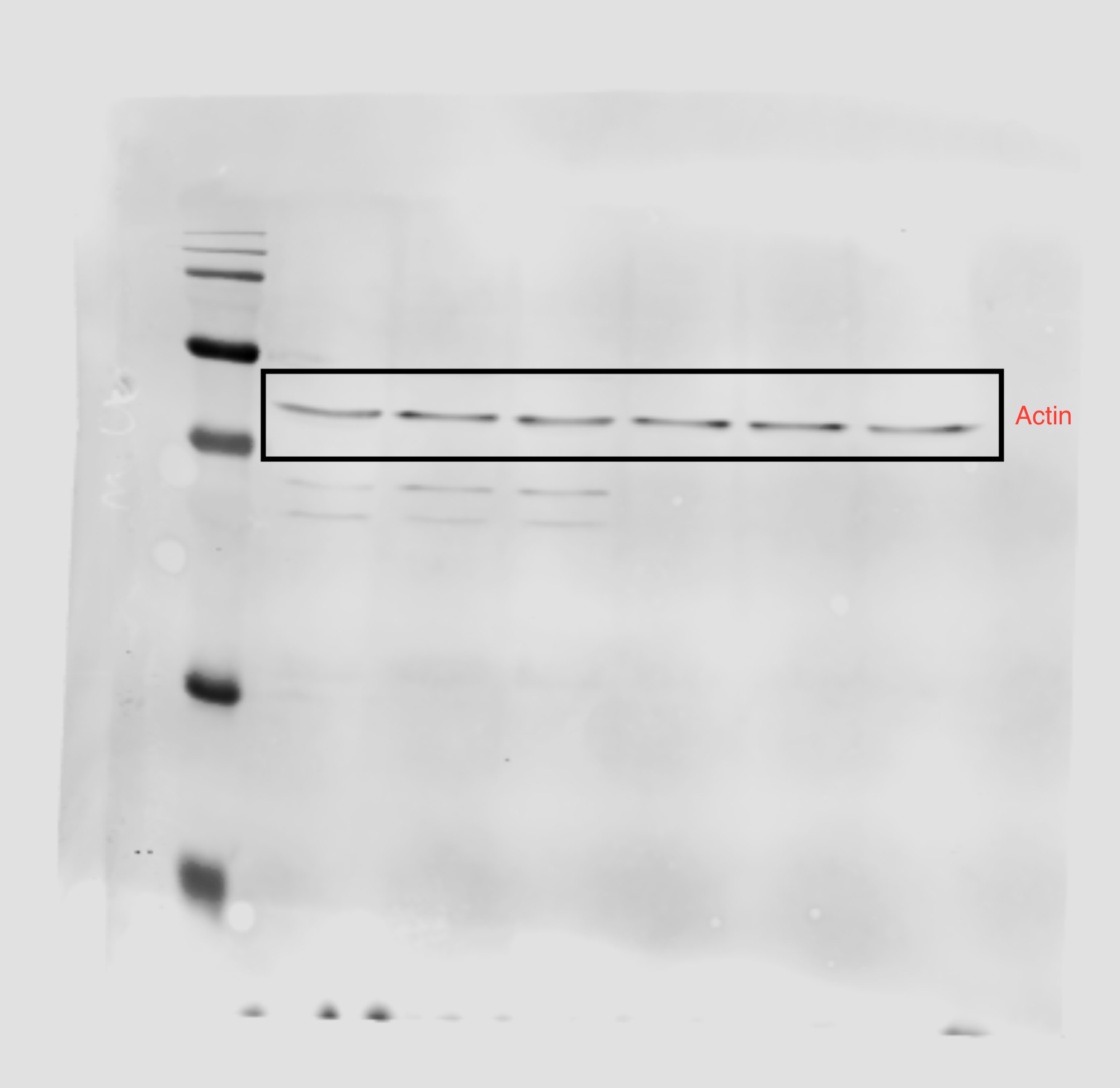

Supplement: Supplementary file 4 — Source Data Fig. 1 [file 44318_2024_44_MOESM4_ESM.zip › Fig 1/Fig 1G/Fig1G_mitoprofile_actin.tif]

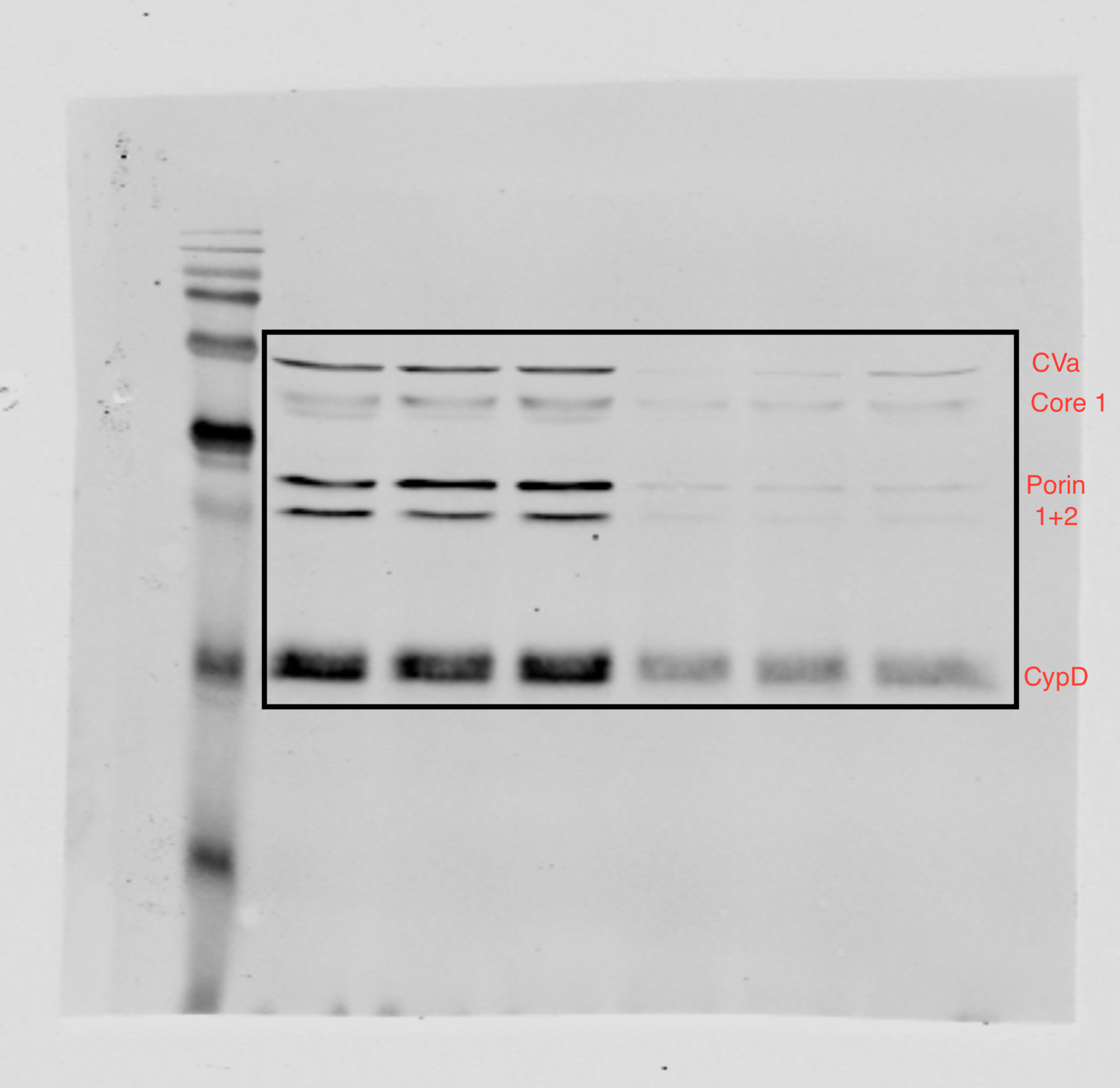

Supplement: Supplementary file 4 — Source Data Fig. 1 [file 44318_2024_44_MOESM4_ESM.zip › Fig 1/Fig 1G/Fig1G_mitoprofile.tif]

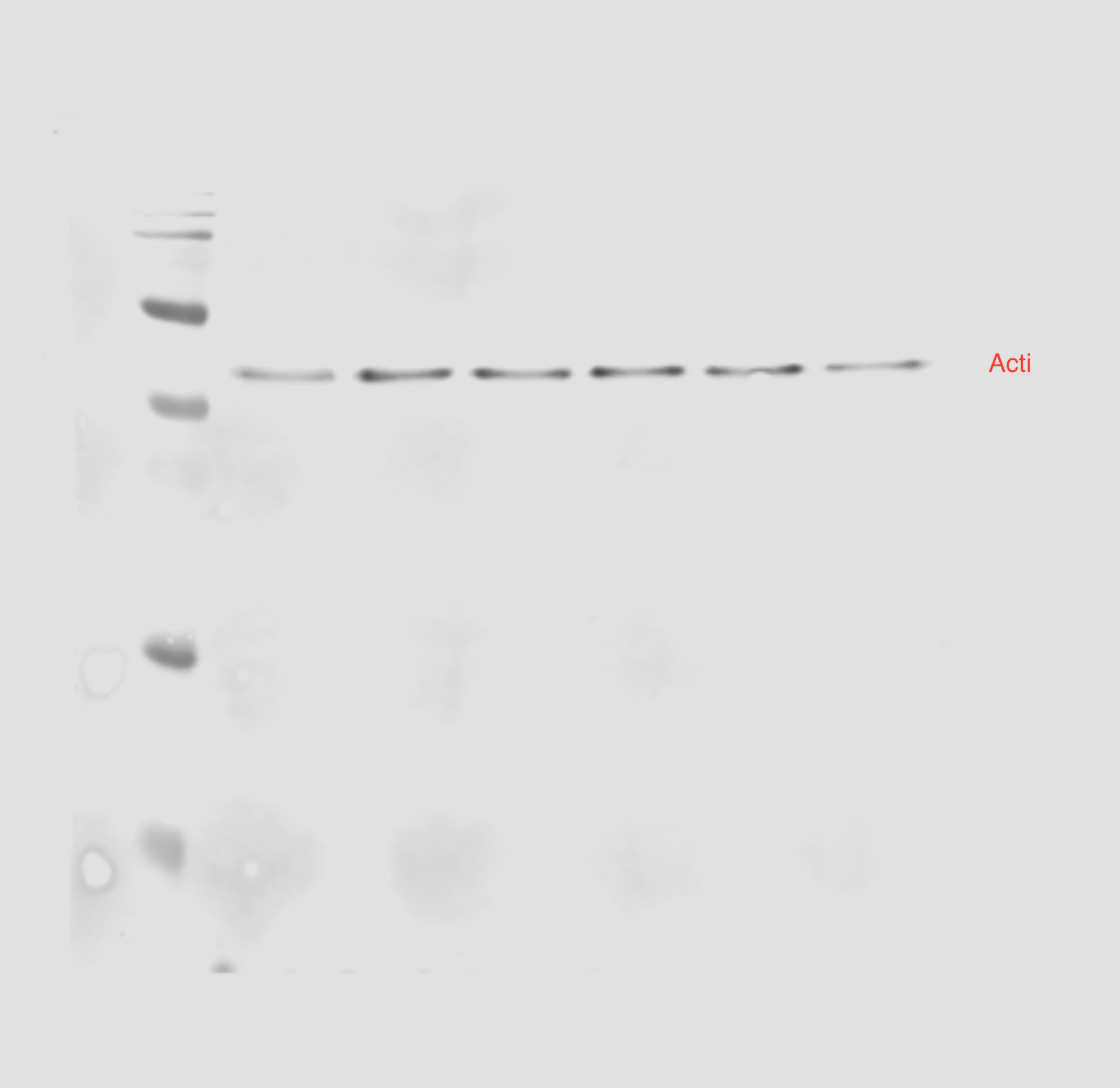

Supplement: Supplementary file 4 — Source Data Fig. 1 [file 44318_2024_44_MOESM4_ESM.zip › Fig 1/Fig 1G/Fig1G_LC3_ATG5-ATG7_actin.tif]

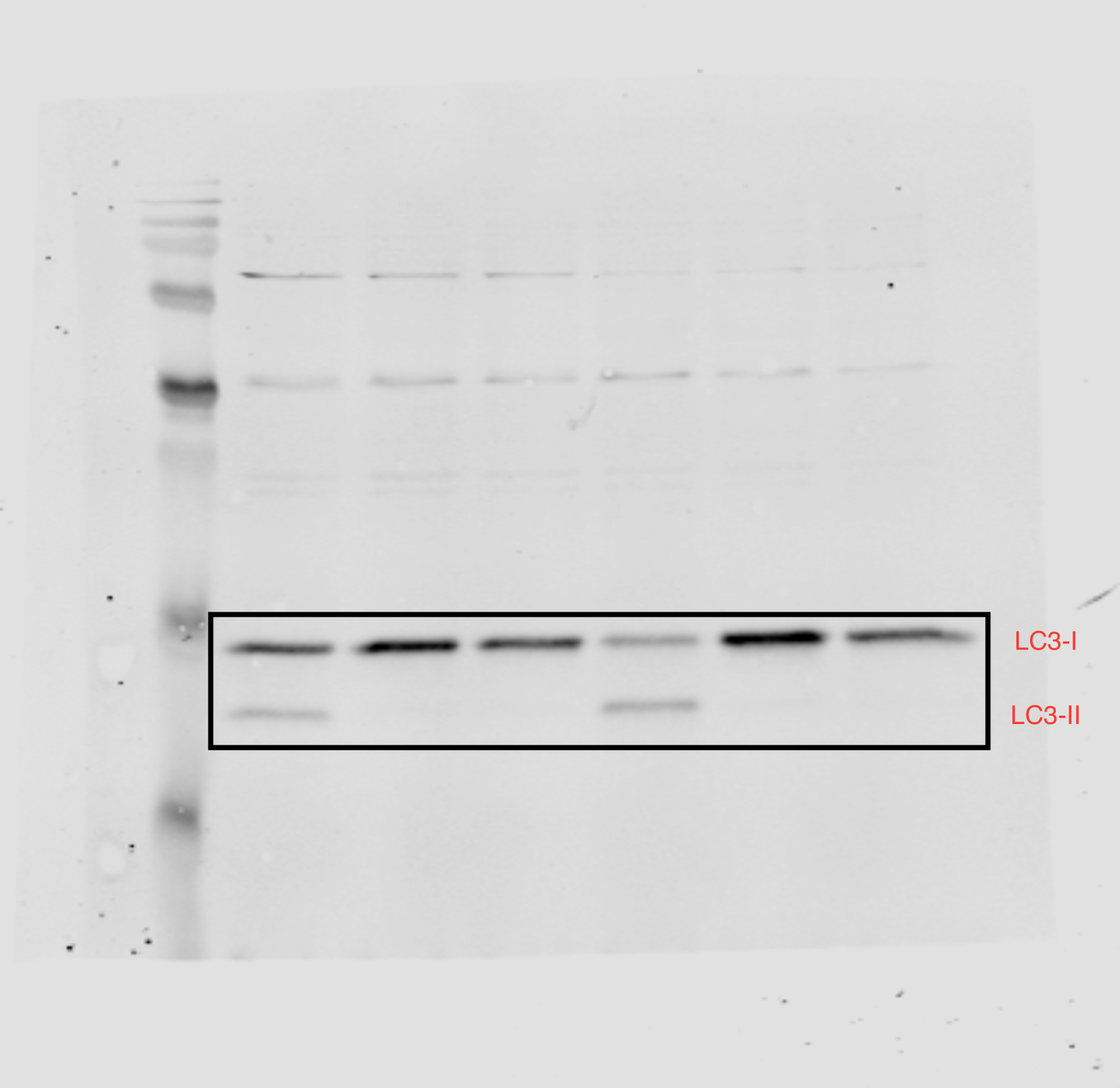

Supplement: Supplementary file 4 — Source Data Fig. 1 [file 44318_2024_44_MOESM4_ESM.zip › Fig 1/Fig 1G/Fig1G_LC3.tif]

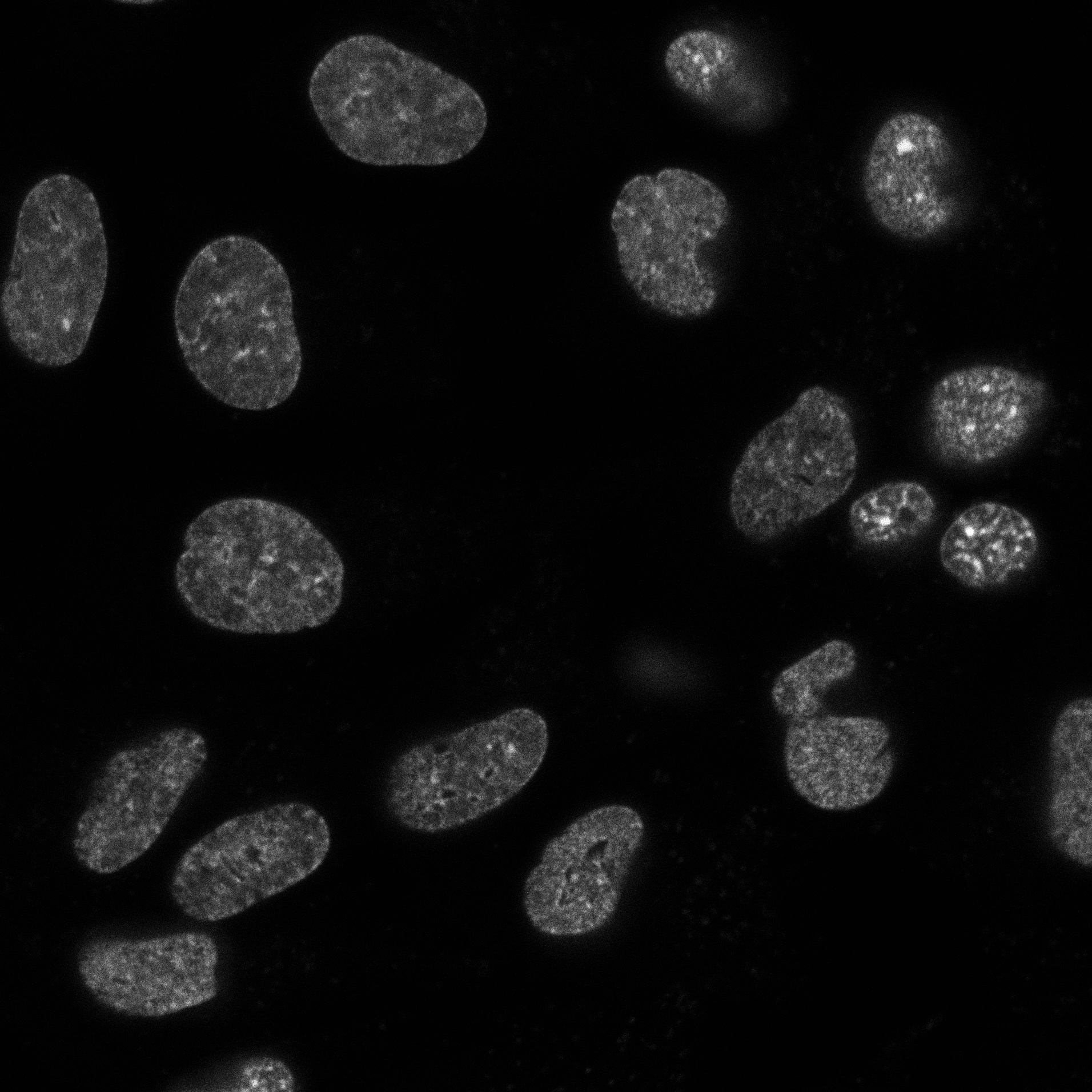

Supplement: Supplementary file 4 — Source Data Fig. 1 [file 44318_2024_44_MOESM4_ESM.zip › Fig 1/Fig 1C/Fig_1C-U2OS-BB-CICD-3h-mito-hoechst.tif]

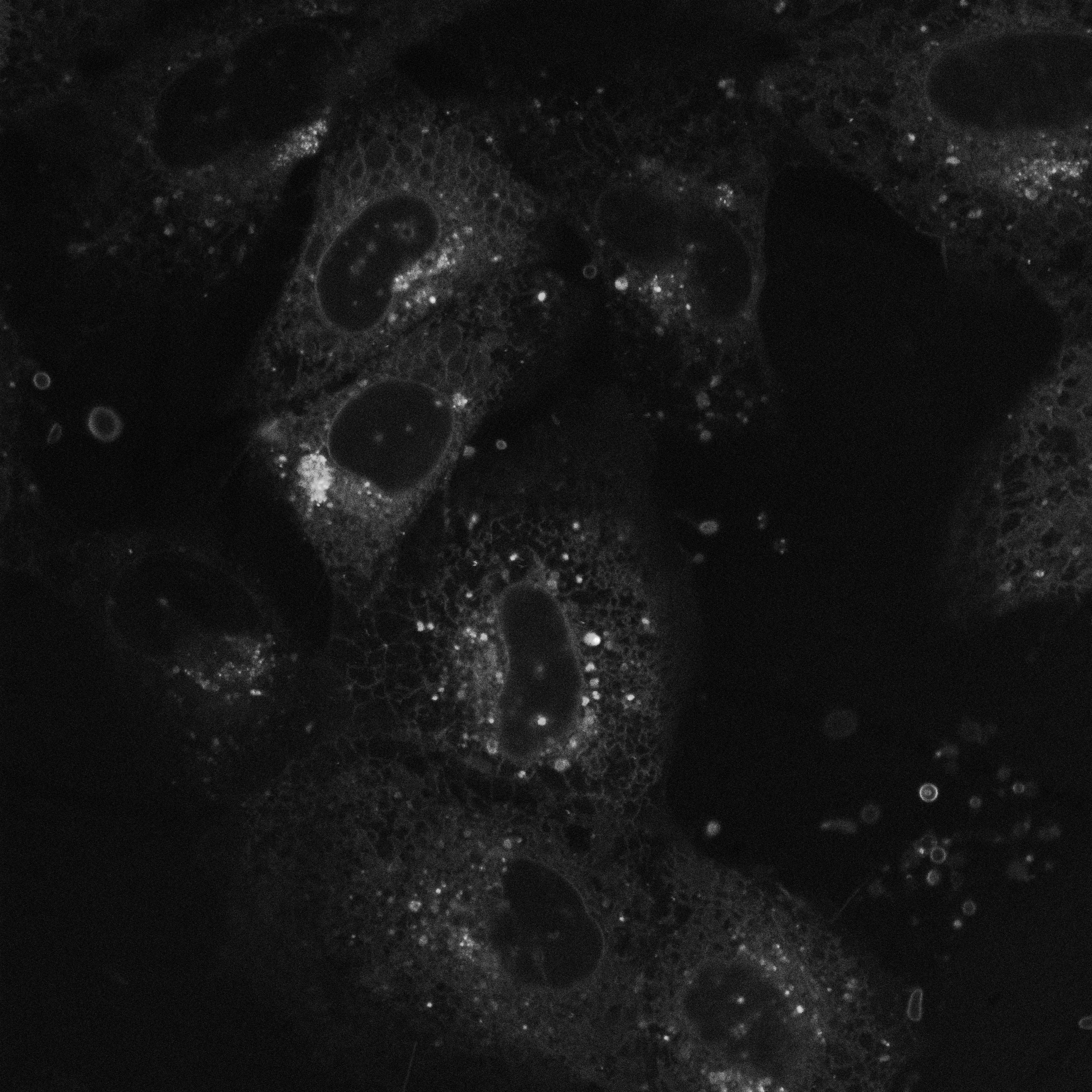

Supplement: Supplementary file 4 — Source Data Fig. 1 [file 44318_2024_44_MOESM4_ESM.zip › Fig 1/Fig 1C/Fig_1C-U2OS-eBB-CICD-24h-mito-MitoTracker.tif]

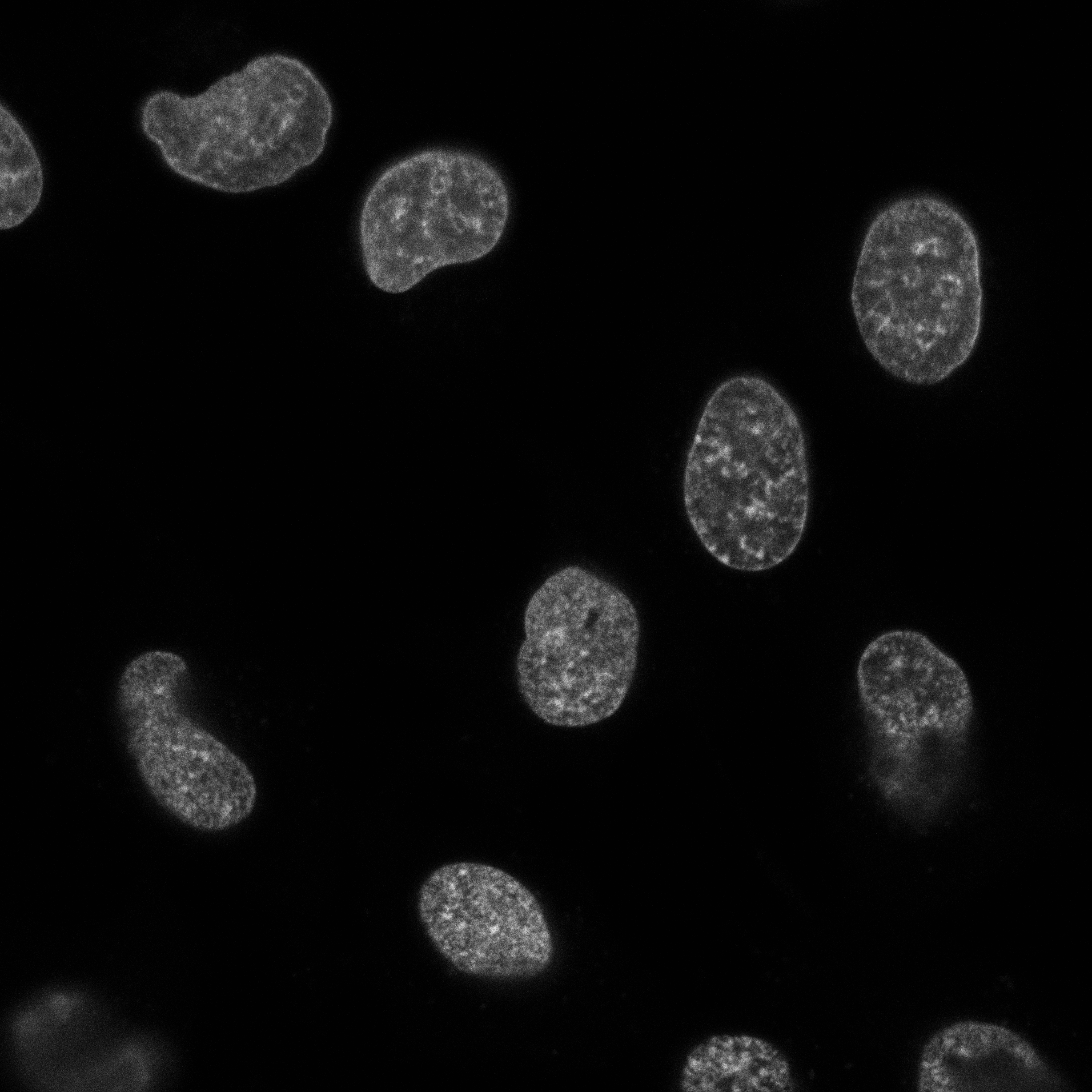

Supplement: Supplementary file 4 — Source Data Fig. 1 [file 44318_2024_44_MOESM4_ESM.zip › Fig 1/Fig 1C/Fig_1C-U2OS-eBB-CICD-3h-mito-hoechst.tif]

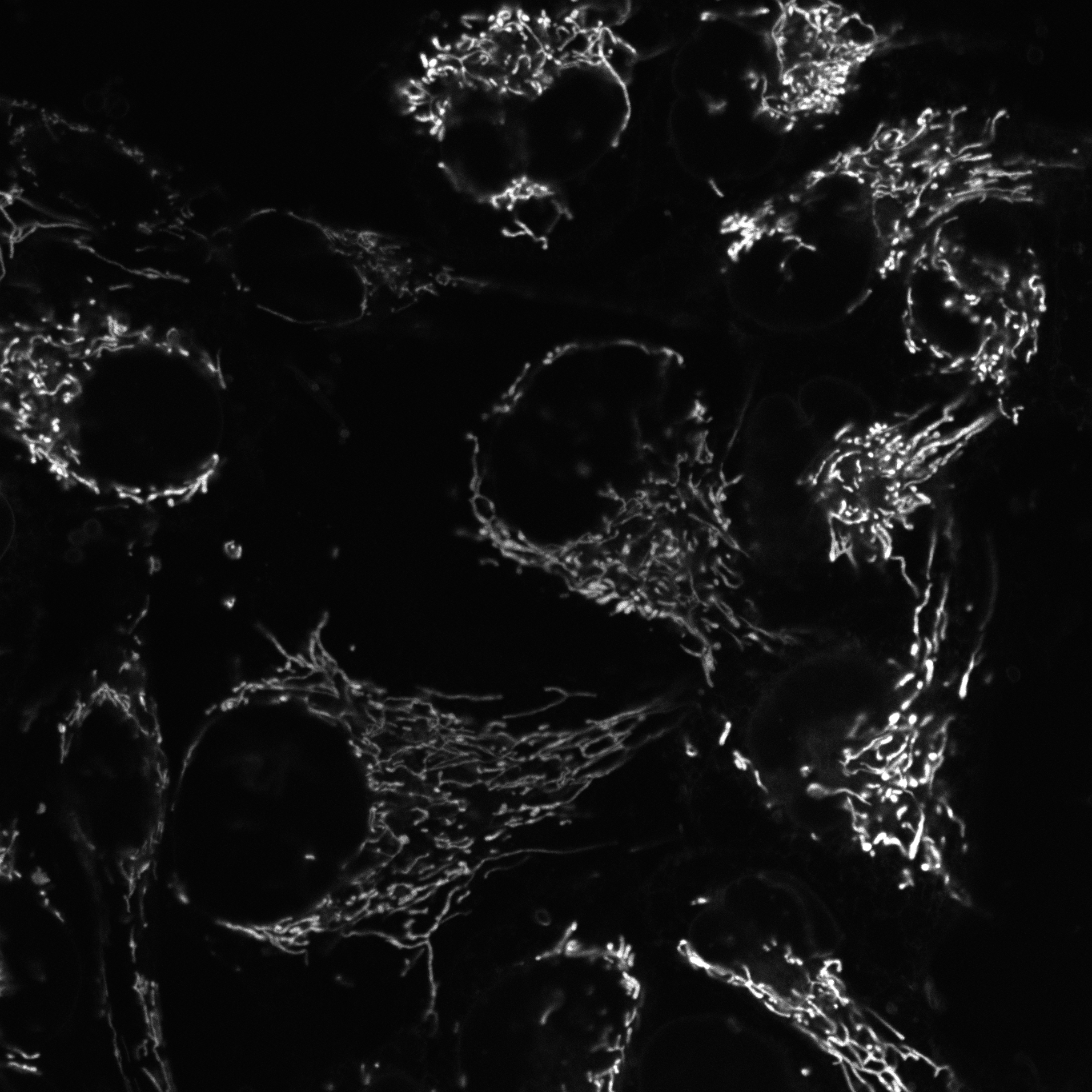

Supplement: Supplementary file 4 — Source Data Fig. 1 [file 44318_2024_44_MOESM4_ESM.zip › Fig 1/Fig 1C/Fig_1C-U2OS-BB-DMSO-3h-mito-MiotTracker.tif]

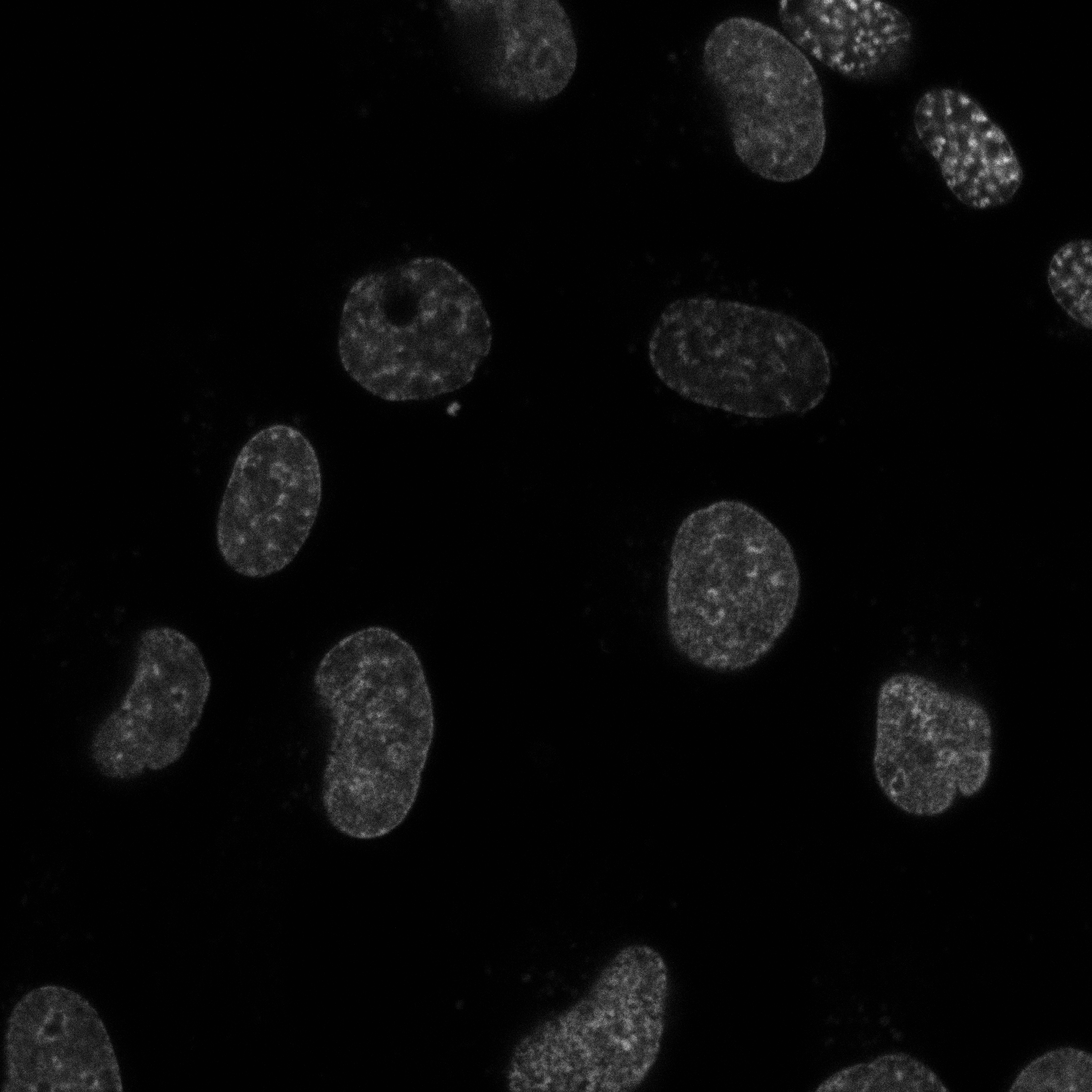

Supplement: Supplementary file 4 — Source Data Fig. 1 [file 44318_2024_44_MOESM4_ESM.zip › Fig 1/Fig 1C/Fig_1C-U2OS-BB-DMSO-24h-mito-hoechst.tif]

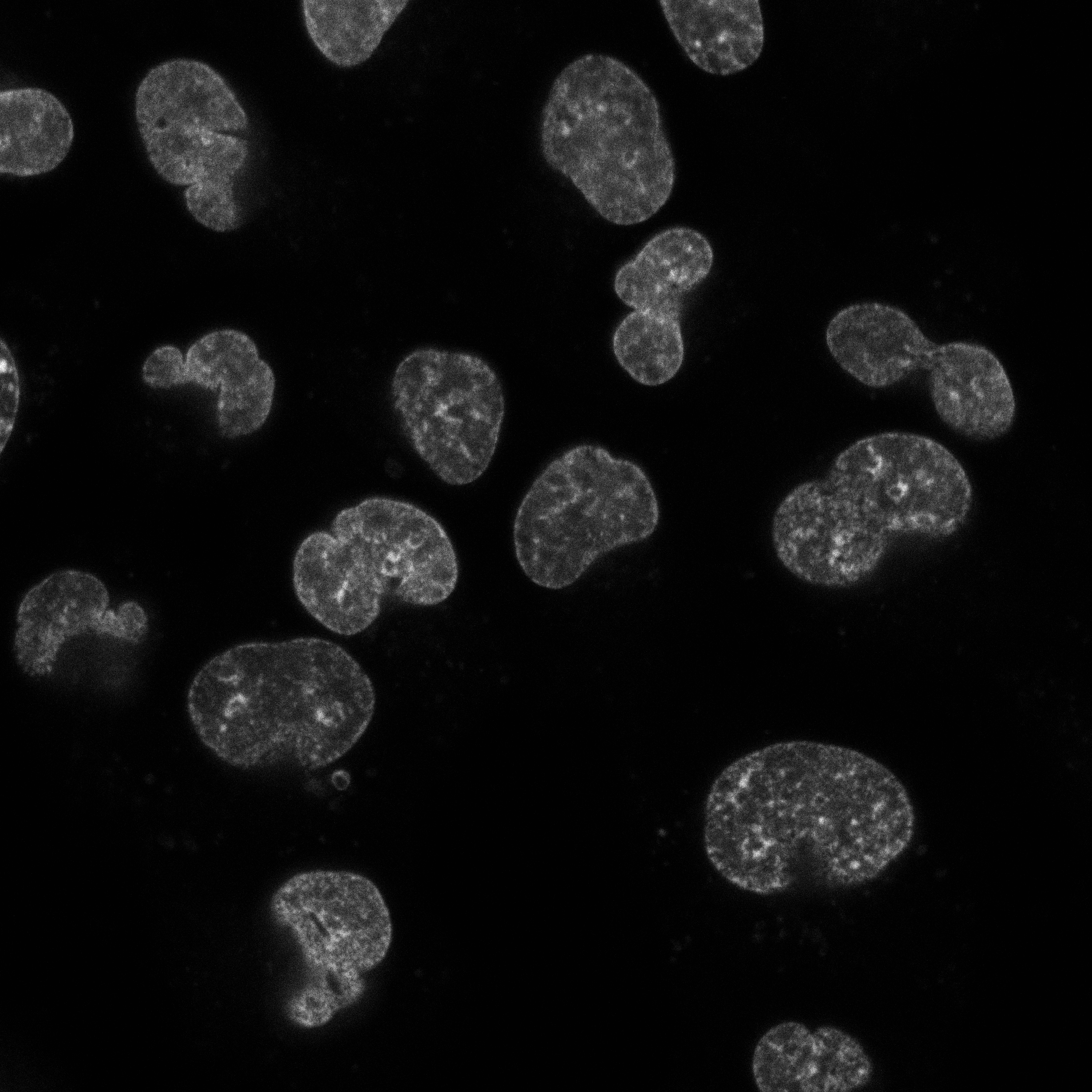

Supplement: Supplementary file 4 — Source Data Fig. 1 [file 44318_2024_44_MOESM4_ESM.zip › Fig 1/Fig 1C/Fig1_C-U2OS-eBB-DMSO-3h-mito-hoechsttif.tif]

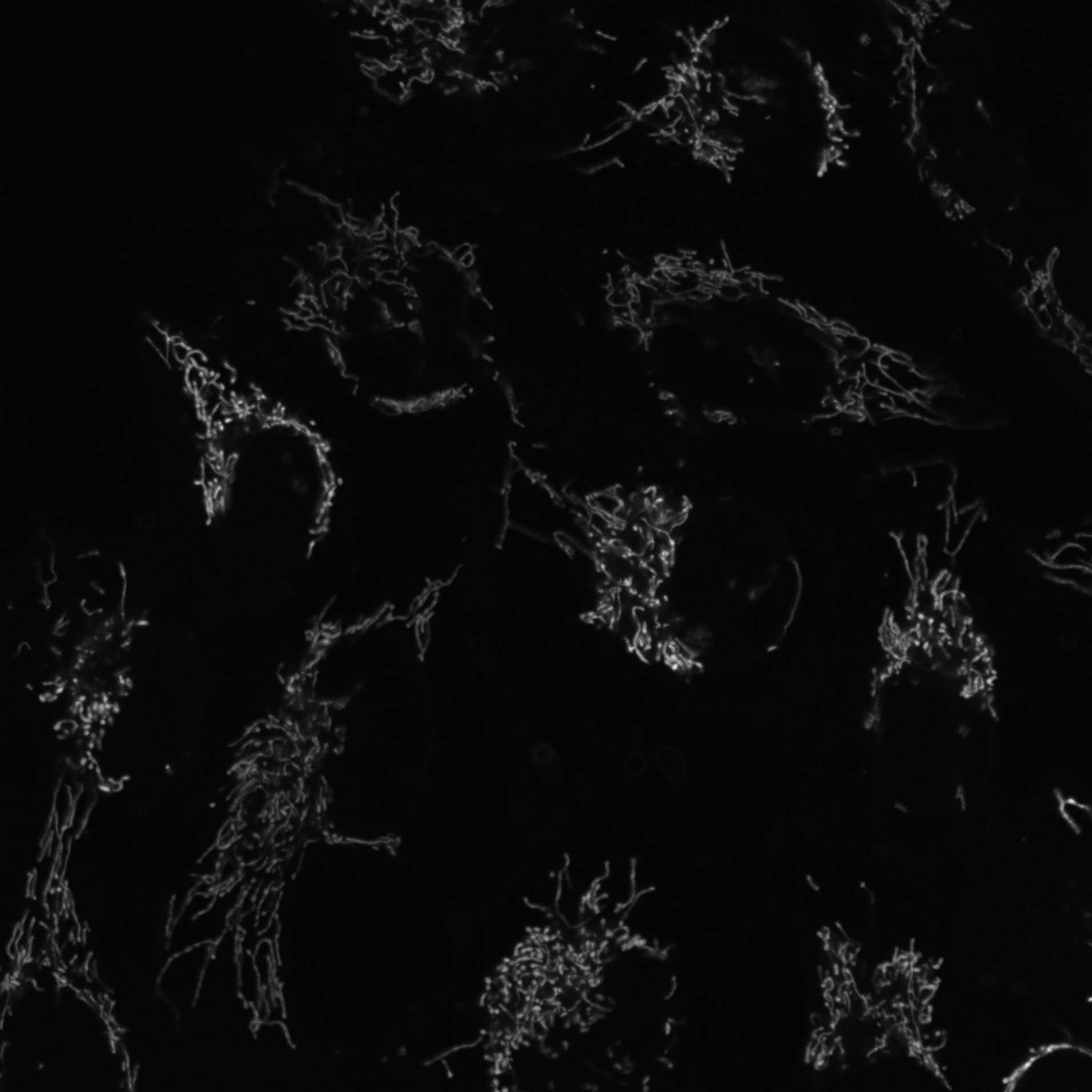

Supplement: Supplementary file 4 — Source Data Fig. 1 [file 44318_2024_44_MOESM4_ESM.zip › Fig 1/Fig 1C/Fig_1C-U2OS-BB-DMSO-24h-mito-MitoTracker.tif]

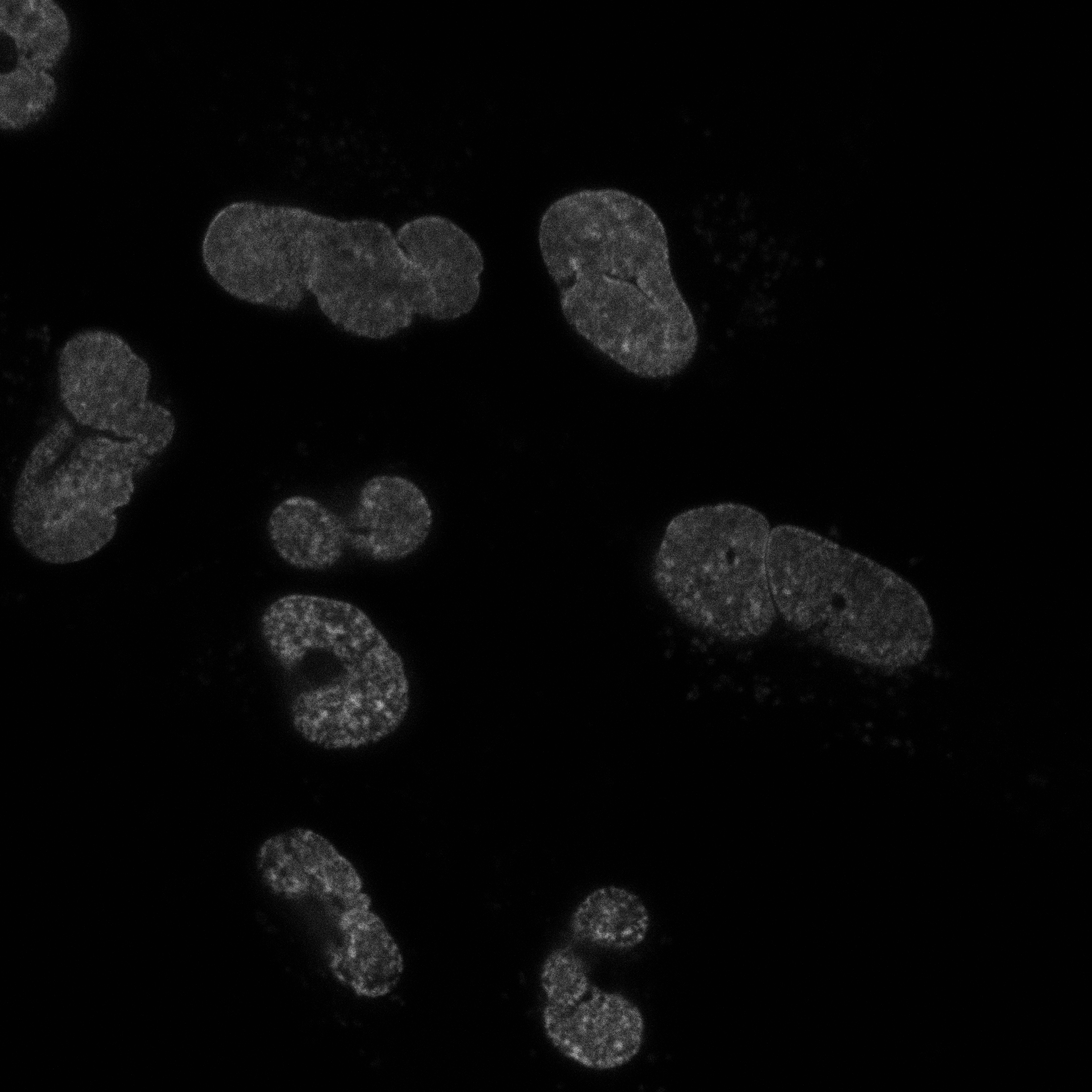

Supplement: Supplementary file 4 — Source Data Fig. 1 [file 44318_2024_44_MOESM4_ESM.zip › Fig 1/Fig 1C/Fig_1C-U2OS-BB-CICD-24h-mito-hoechst.tif]

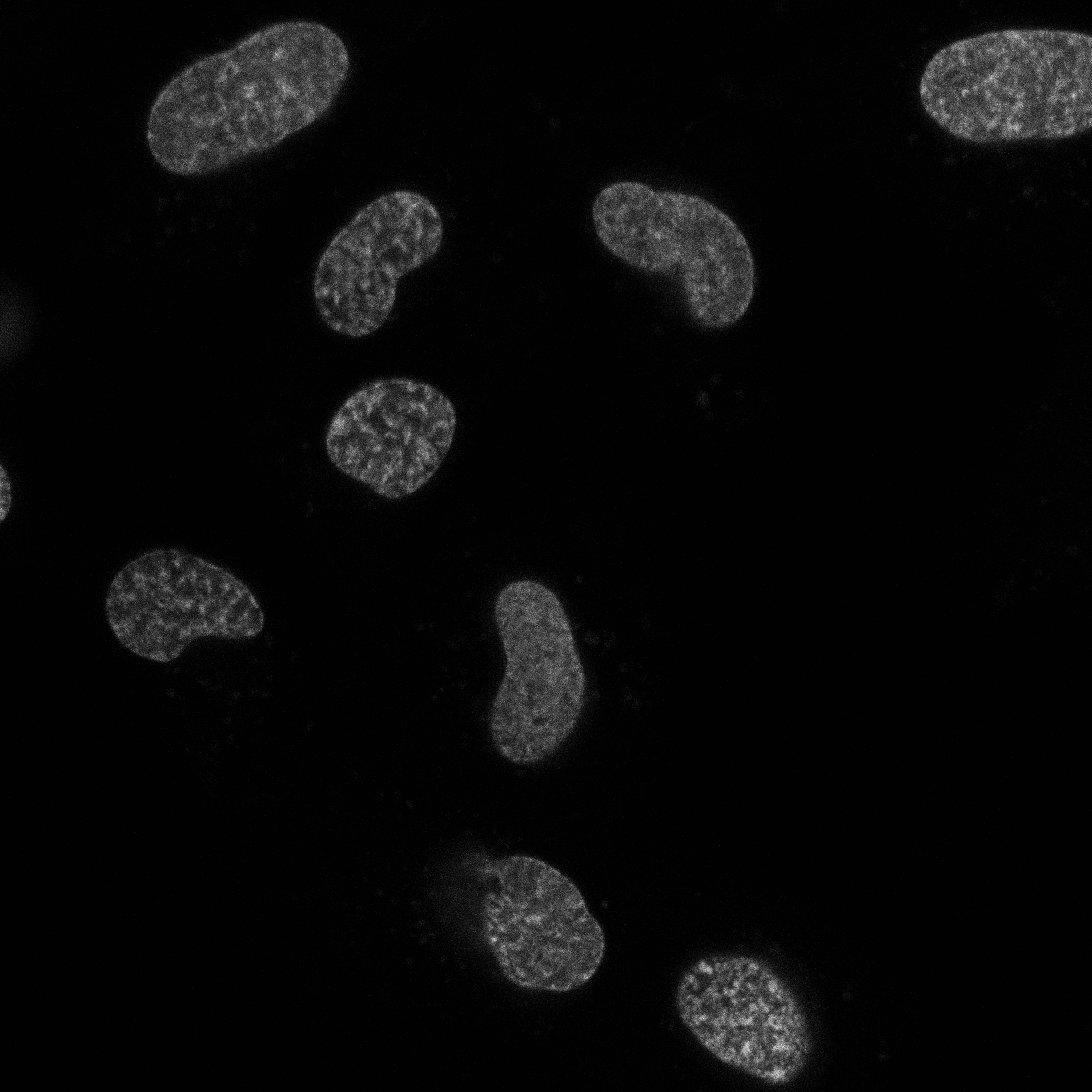

Supplement: Supplementary file 4 — Source Data Fig. 1 [file 44318_2024_44_MOESM4_ESM.zip › Fig 1/Fig 1C/Fig_1C-U2OS-eBB-CICD-24h-mito-hoechsttif.tif]

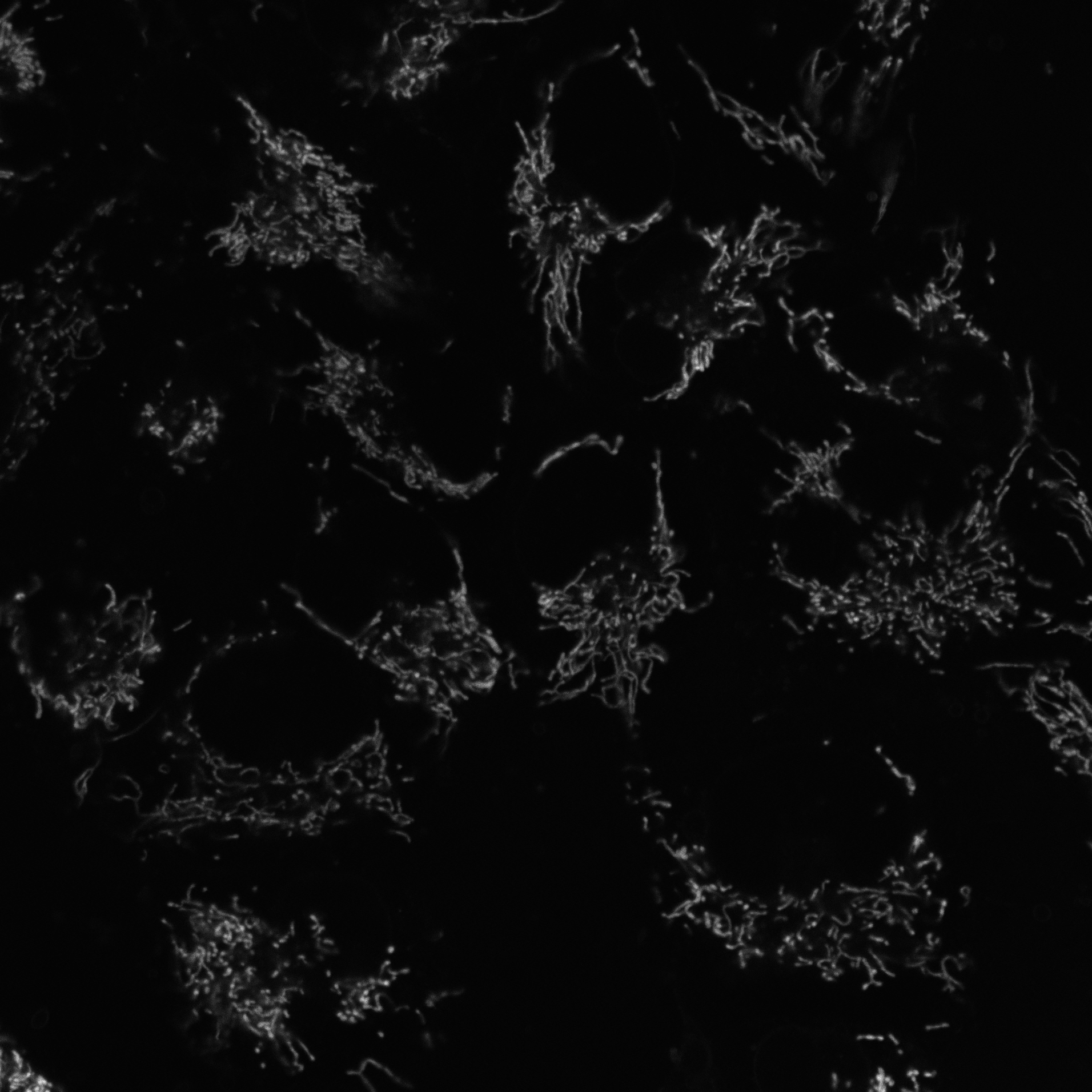

Supplement: Supplementary file 4 — Source Data Fig. 1 [file 44318_2024_44_MOESM4_ESM.zip › Fig 1/Fig 1C/Fig_1C-U2OS-eBB-DMSO-3h-mito-MitoTracker.tif]

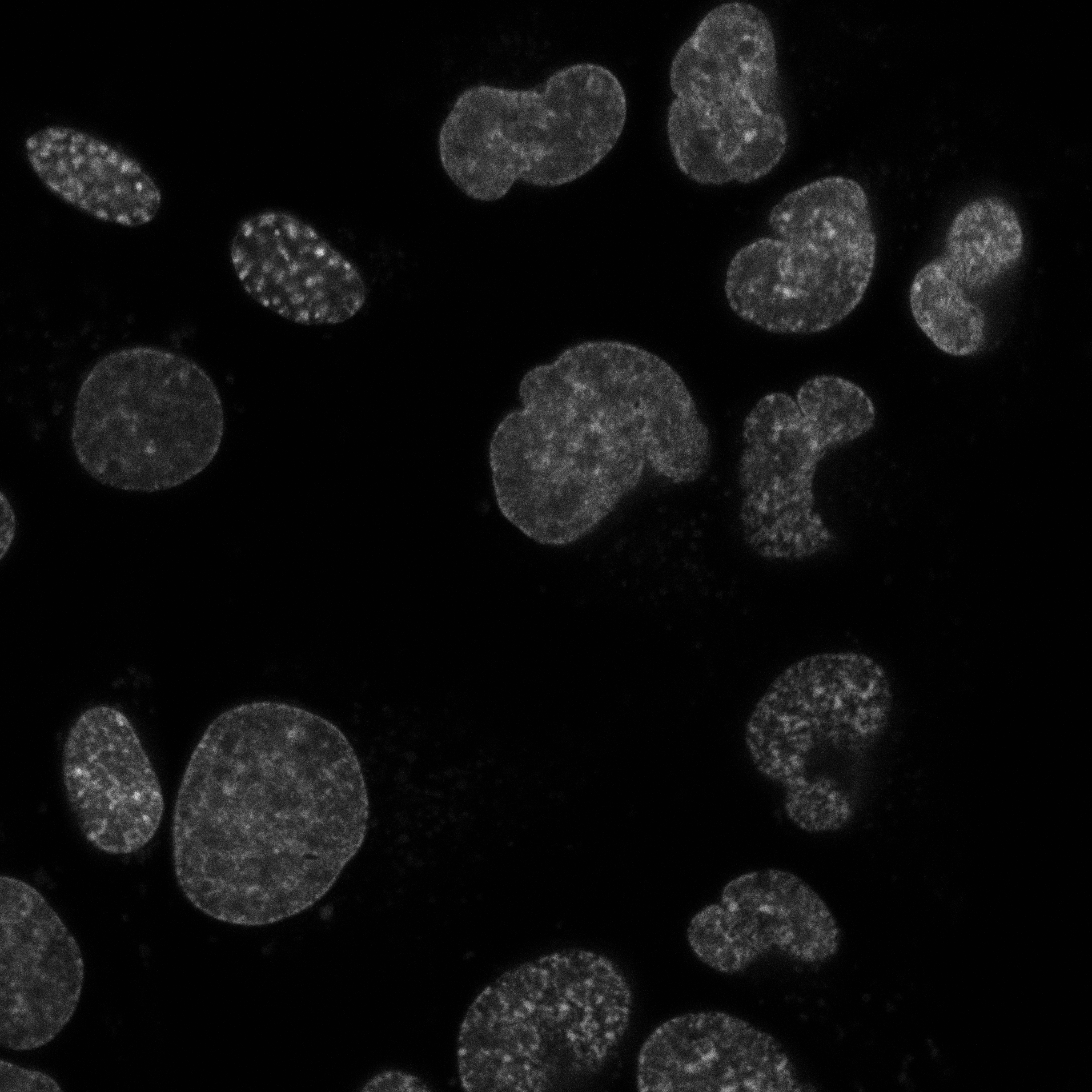

Supplement: Supplementary file 4 — Source Data Fig. 1 [file 44318_2024_44_MOESM4_ESM.zip › Fig 1/Fig 1C/Fig_1C-U2OS-BB-DMSO-3h-mito-hoechst.tif]

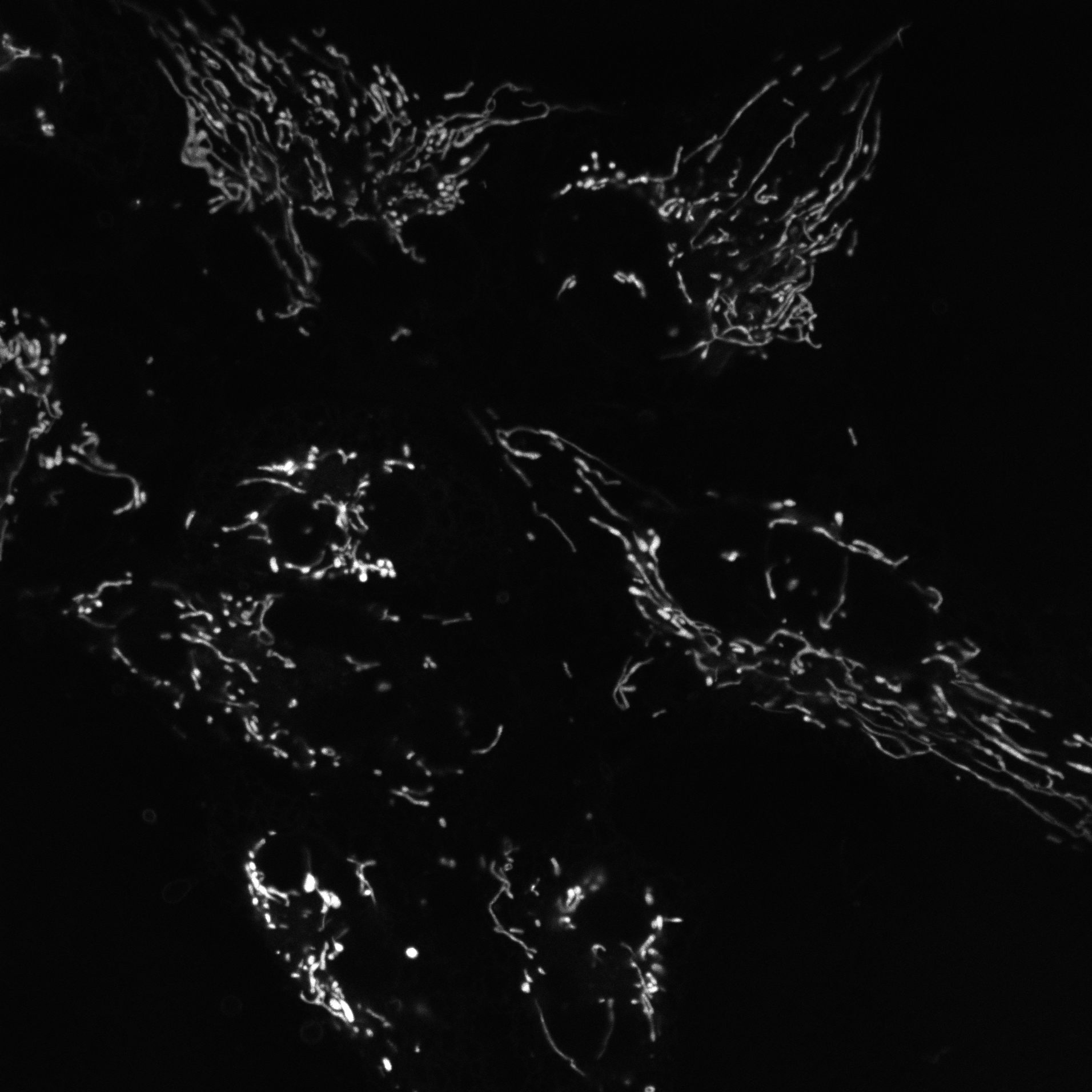

Supplement: Supplementary file 4 — Source Data Fig. 1 [file 44318_2024_44_MOESM4_ESM.zip › Fig 1/Fig 1C/Fig_1C-U2OS-BB-CICD-24h-mito-MitoTracker.tif]

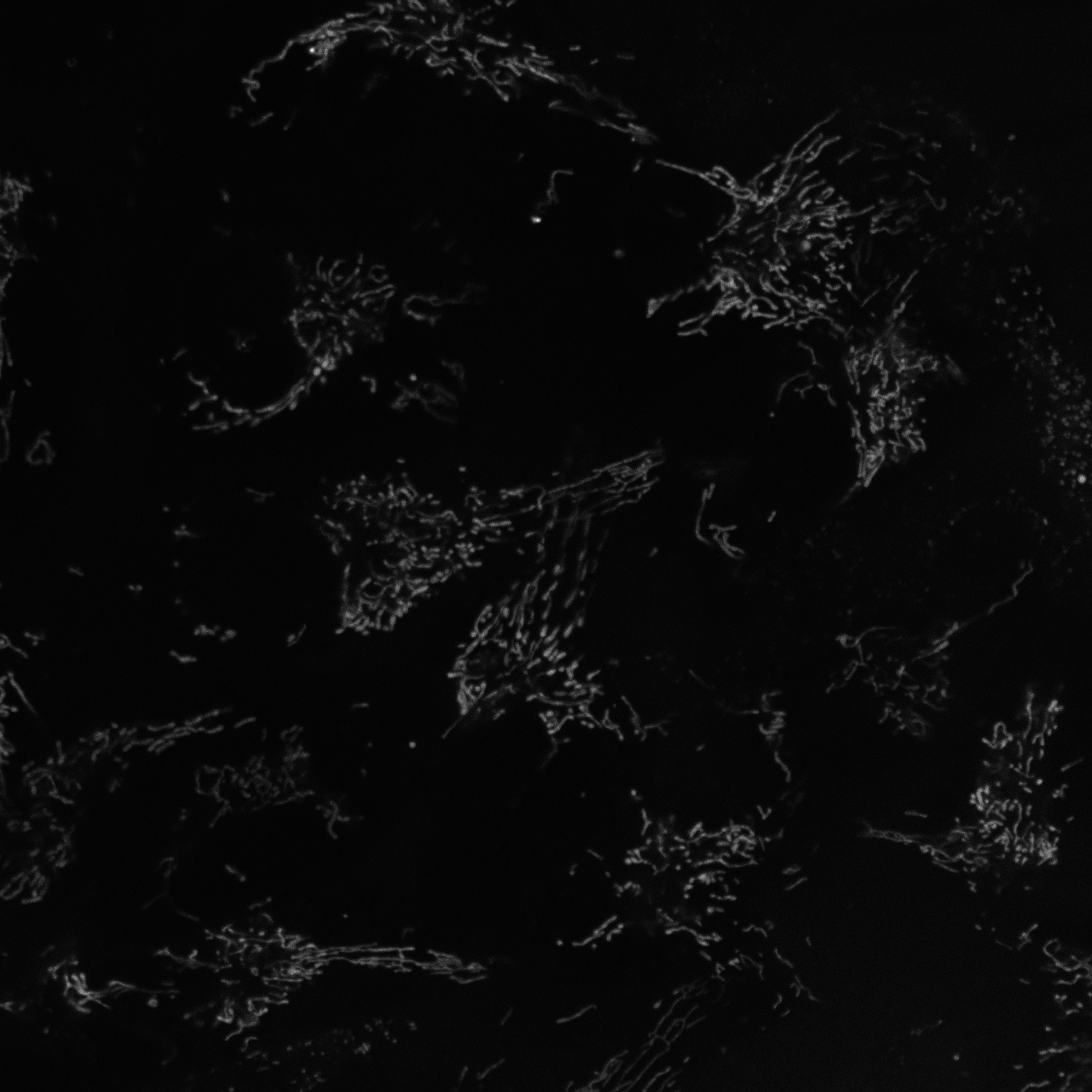

Supplement: Supplementary file 4 — Source Data Fig. 1 [file 44318_2024_44_MOESM4_ESM.zip › Fig 1/Fig 1C/Fig_1C-U2OS-BB-CICD-3h-mito-MitoTracker.tif]

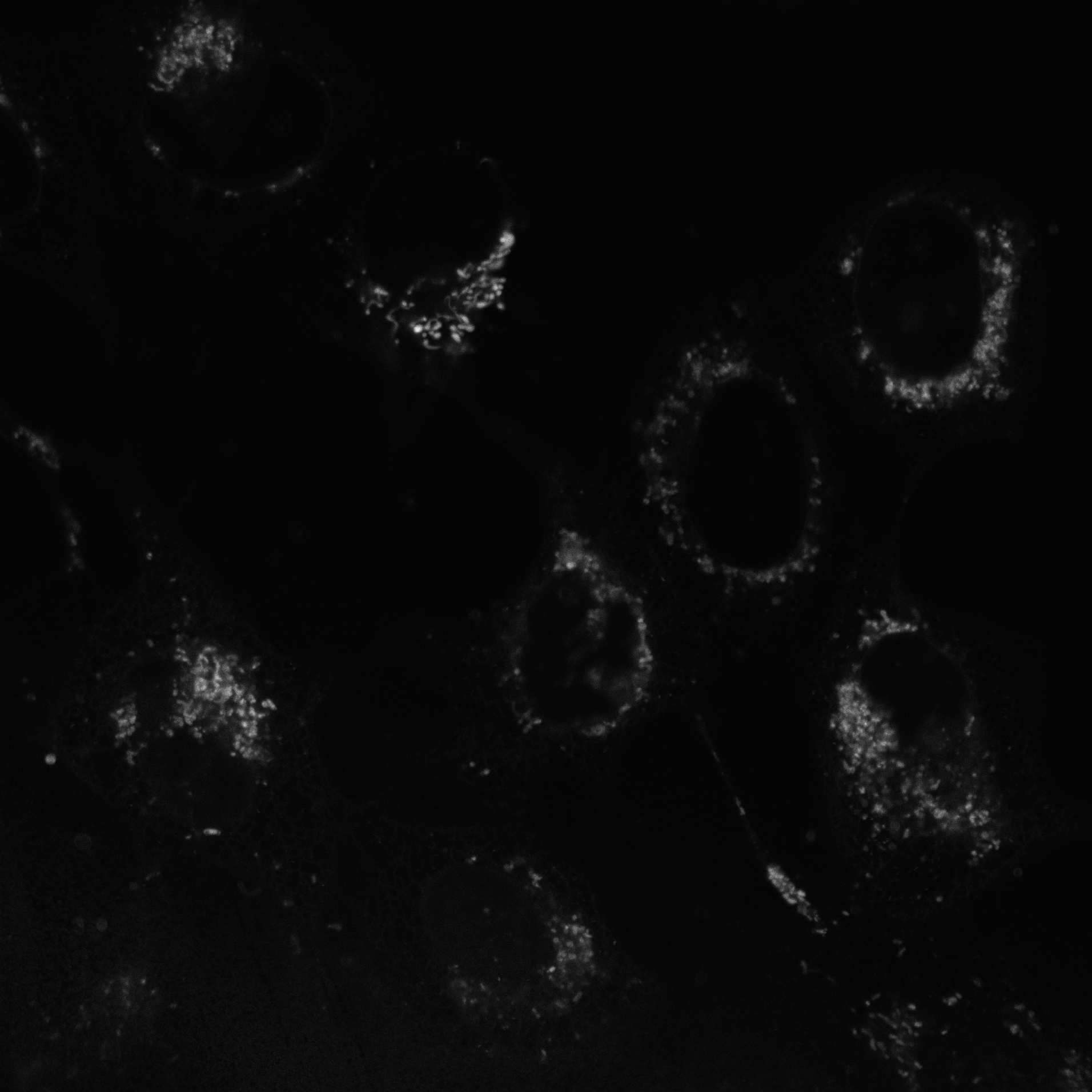

Supplement: Supplementary file 4 — Source Data Fig. 1 [file 44318_2024_44_MOESM4_ESM.zip › Fig 1/Fig 1C/Fig_1C-U2OS-eBB-CICD-3h-mito-MitoTracker.tif]

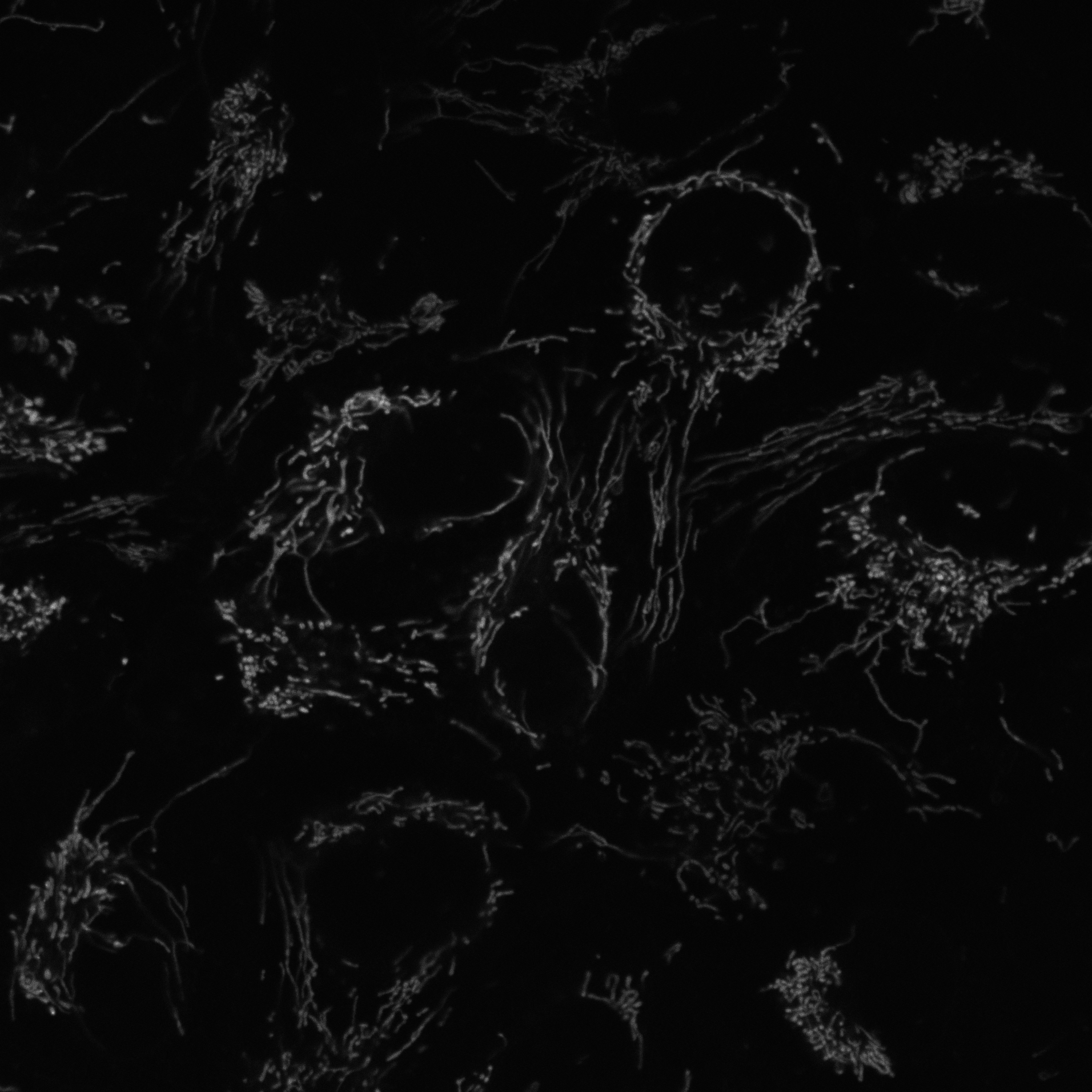

Supplement: Supplementary file 4 — Source Data Fig. 1 [file 44318_2024_44_MOESM4_ESM.zip › Fig 1/Fig 1C/Fig_1C-U2OS-eBB-DMSO-24h-mito-Mitotrackertif.tif]

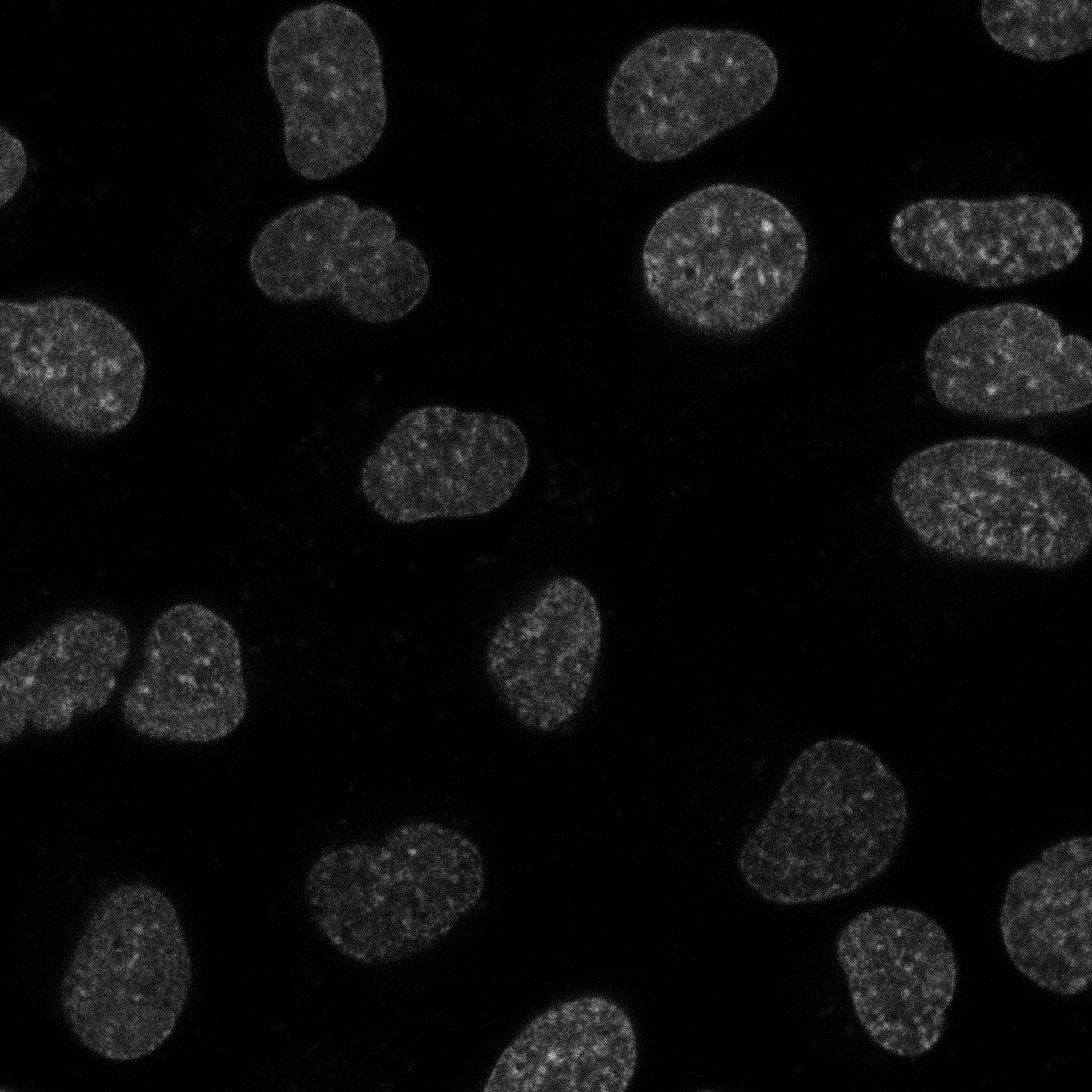

Supplement: Supplementary file 4 — Source Data Fig. 1 [file 44318_2024_44_MOESM4_ESM.zip › Fig 1/Fig 1C/Fig_1C-U2OS-eBB-DMSO-24h-mito-hoechst.tif]

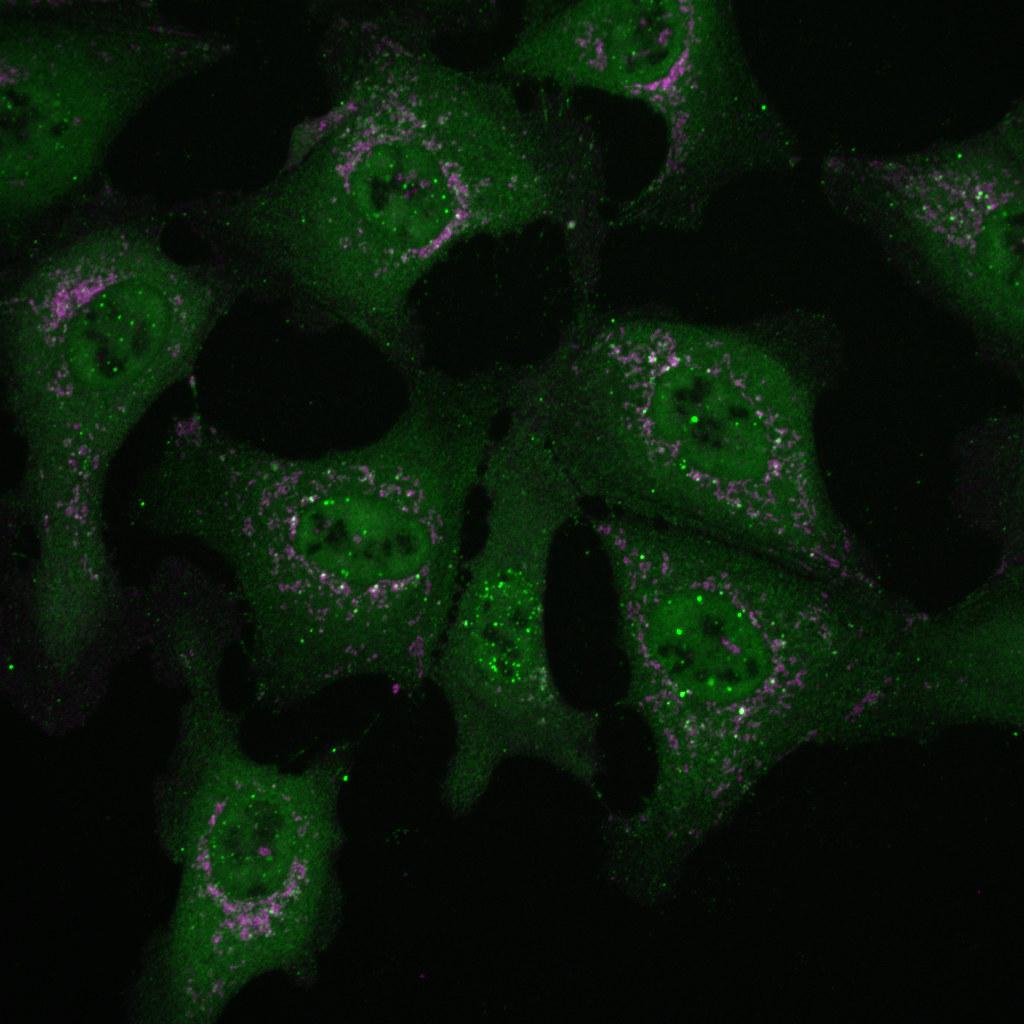

Supplement: Supplementary file 4 — Source Data Fig. 1 [file 44318_2024_44_MOESM4_ESM.zip › Fig 1/Fig 1E/Fig1E_Uncropped_MAX_2019-02-15 fk2 coxiv U20S empty cicd 2.nd2 (RGB).tif]

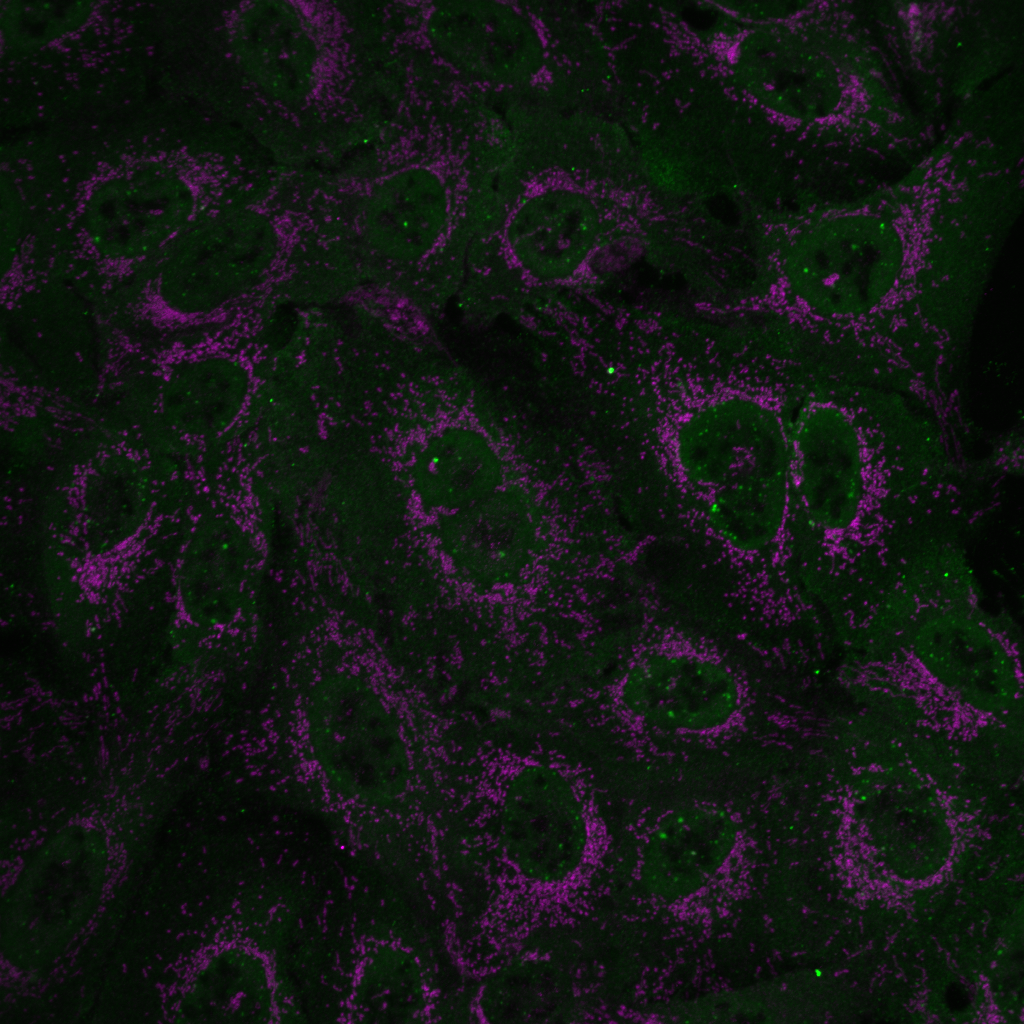

Supplement: Supplementary file 4 — Source Data Fig. 1 [file 44318_2024_44_MOESM4_ESM.zip › Fig 1/Fig 1E/Fig1E_Uncropped_MAX_2019-02-15 fk2 coxiv U20S bb cicd 1.nd2 (RGB).tif]

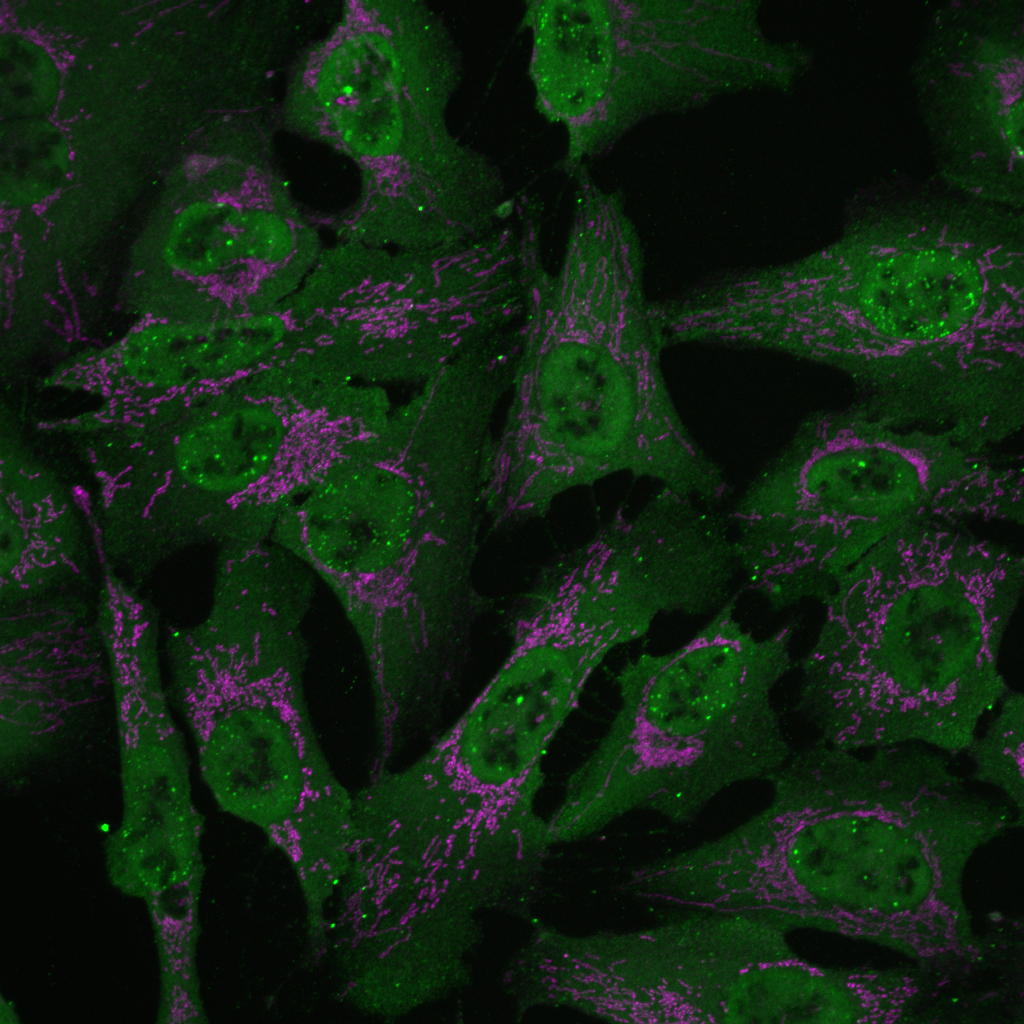

Supplement: Supplementary file 4 — Source Data Fig. 1 [file 44318_2024_44_MOESM4_ESM.zip › Fig 1/Fig 1E/Fig1E_Uncropped_MAX_2019-02-15 fk2 coxiv U20S empty crl 1.nd2 (RGB).tif]

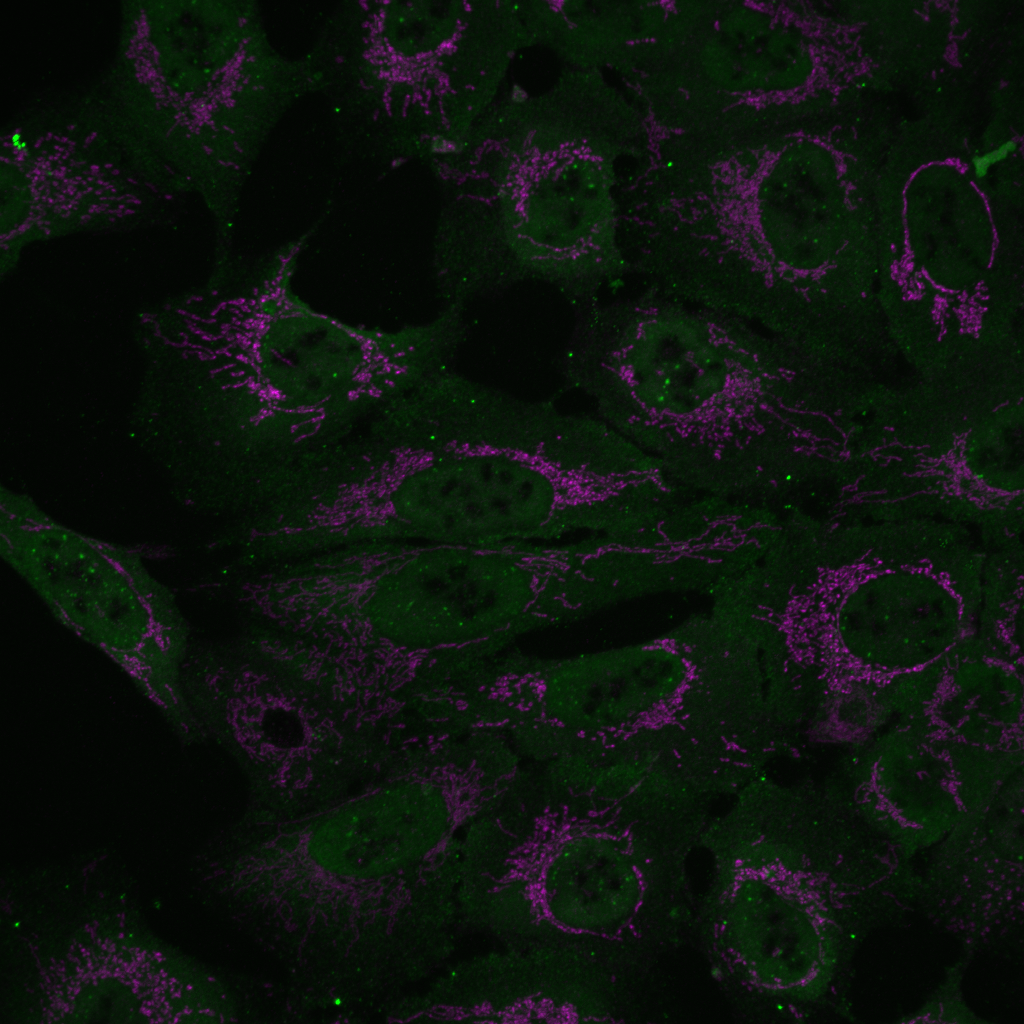

Supplement: Supplementary file 4 — Source Data Fig. 1 [file 44318_2024_44_MOESM4_ESM.zip › Fig 1/Fig 1E/Fig1E_Uncropped_MAX_2019-02-15 fk2 coxiv U20S bb crl 1.nd2 (RGB).tif]

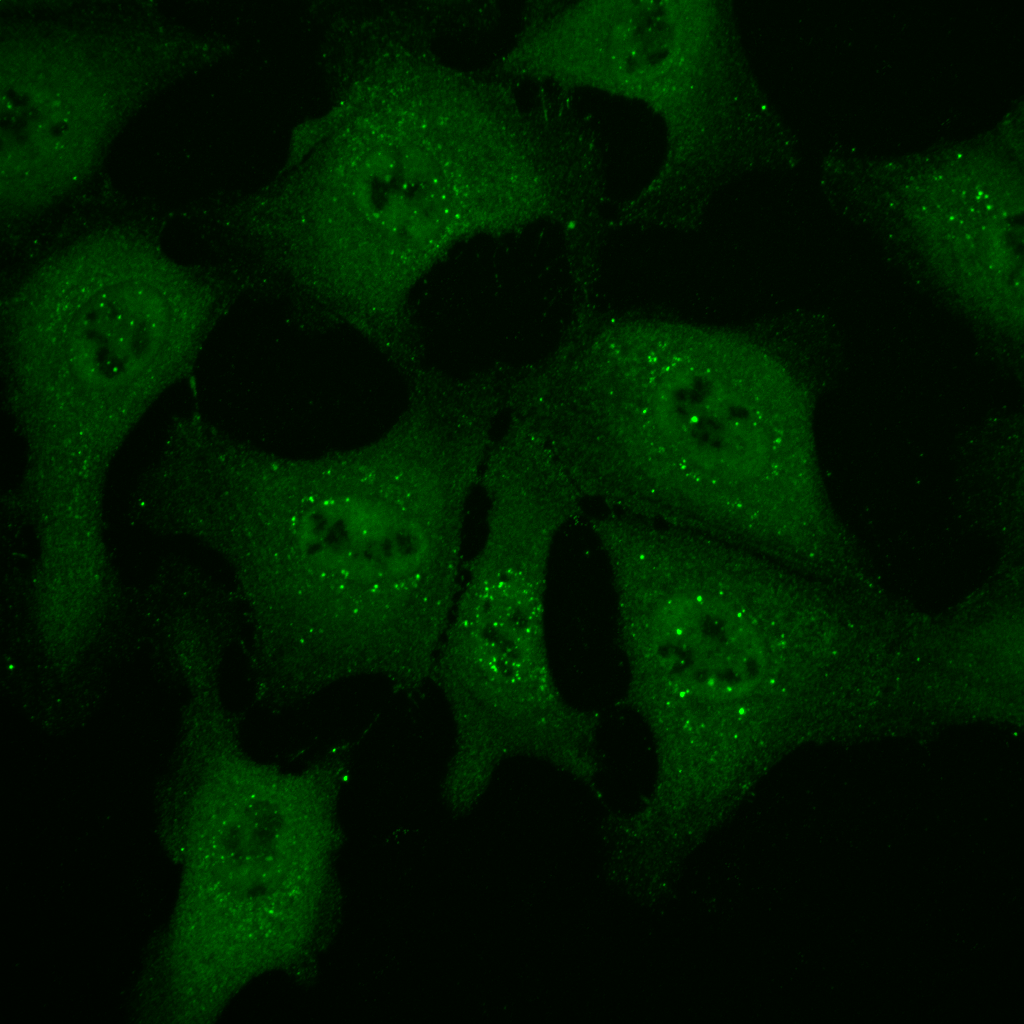

Supplement: Supplementary file 4 — Source Data Fig. 1 [file 44318_2024_44_MOESM4_ESM.zip › Fig 1/Fig 1E/Single channels/Fig1E_UB(green)_Uncropped_MAX_2019-02-15 fk2 coxiv U20S empty cicd 2.nd2 (RGB).tif]

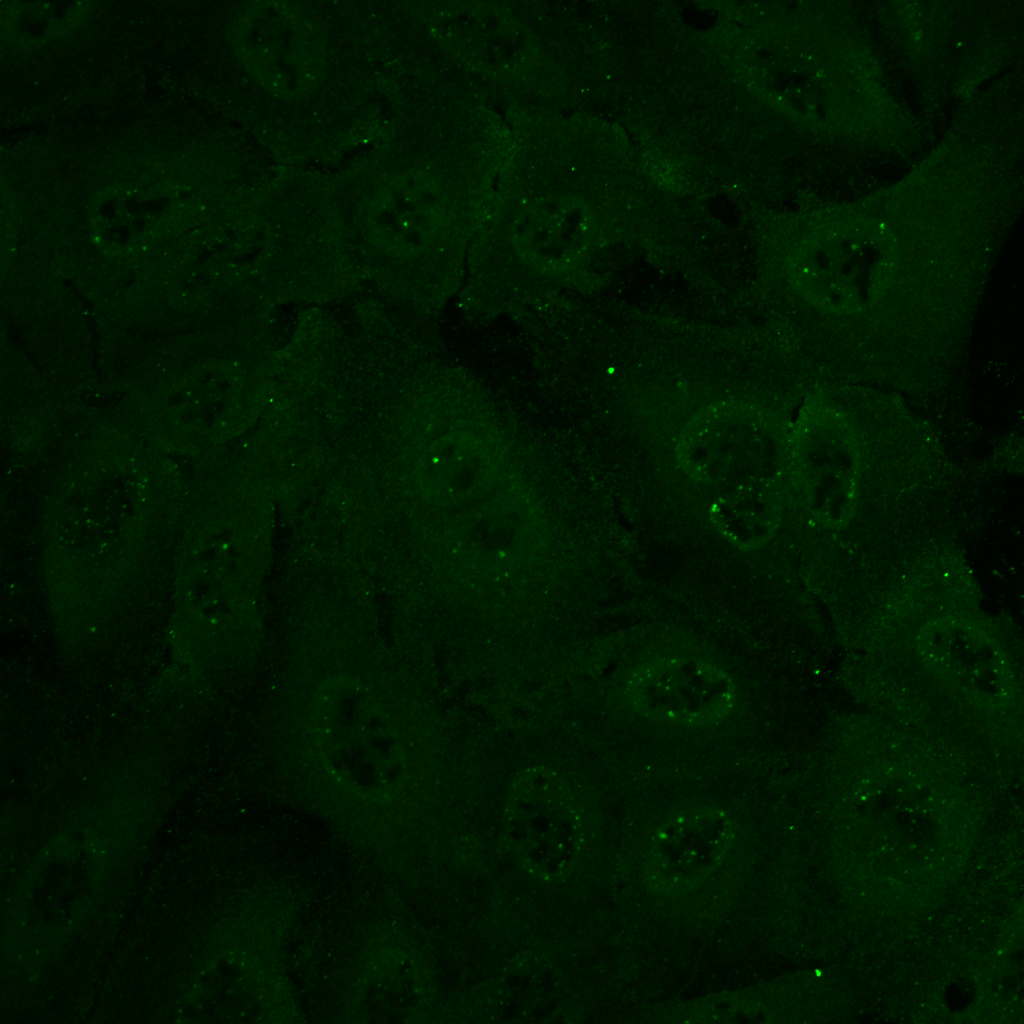

Supplement: Supplementary file 4 — Source Data Fig. 1 [file 44318_2024_44_MOESM4_ESM.zip › Fig 1/Fig 1E/Single channels/Fig1E_UB(green)_Uncropped_MAX_2019-02-15 fk2 coxiv U20S bb cicd 1.nd2 (RGB).tif]

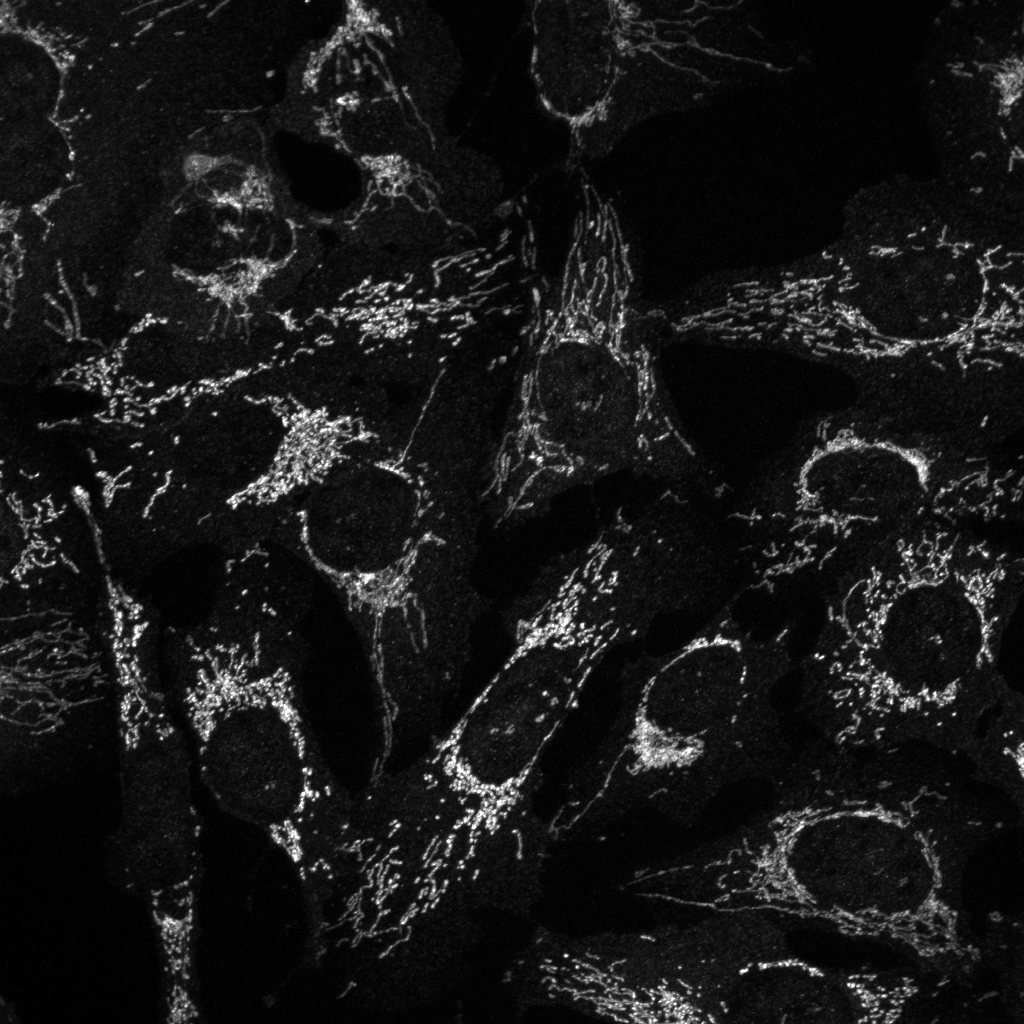

Supplement: Supplementary file 4 — Source Data Fig. 1 [file 44318_2024_44_MOESM4_ESM.zip › Fig 1/Fig 1E/Single channels/Fig1E_UB(green)_Uncropped_MAX_2019-02-15 fk2 coxiv U20S empty crl 1.nd2 (RGB).tif]

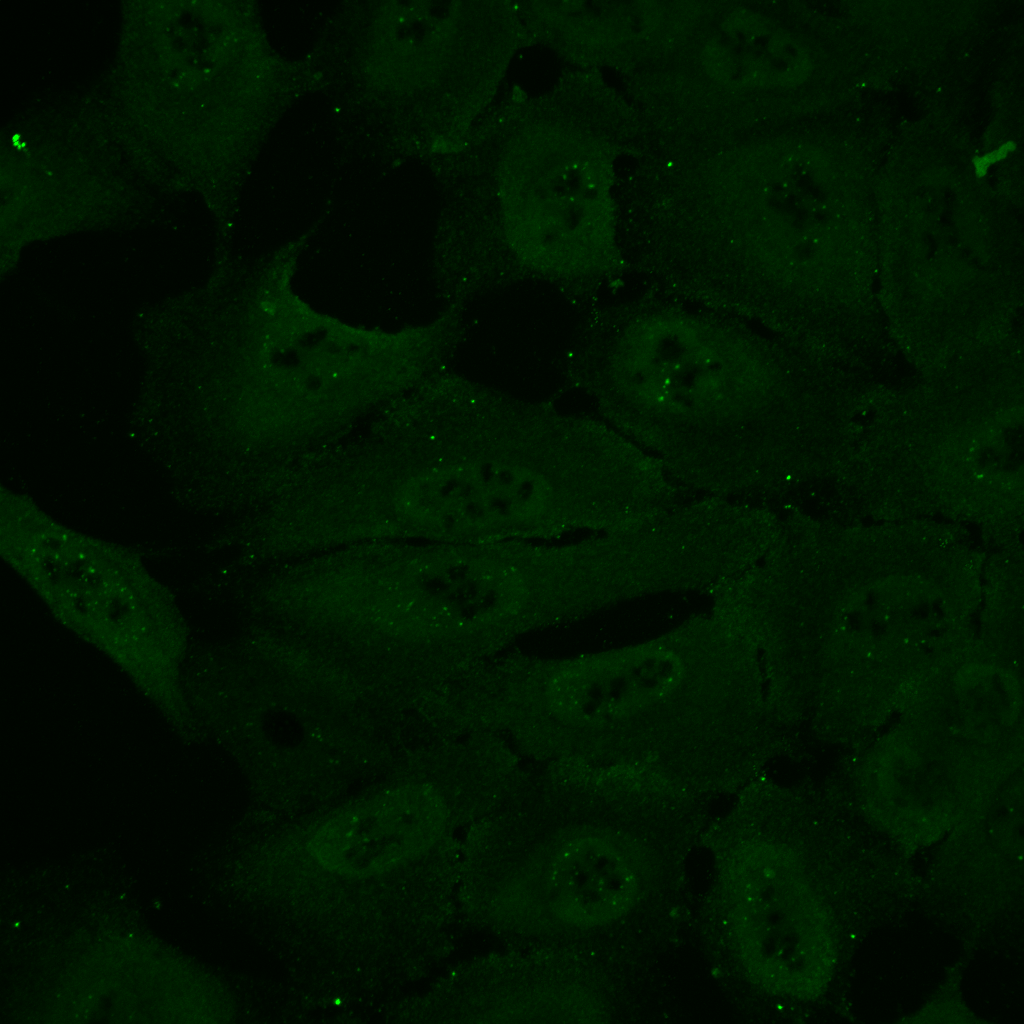

Supplement: Supplementary file 4 — Source Data Fig. 1 [file 44318_2024_44_MOESM4_ESM.zip › Fig 1/Fig 1E/Single channels/Fig1E_UB(green)_Uncropped_MAX_2019-02-15 fk2 coxiv U20S bb crl 1.nd2 (RGB).tif]

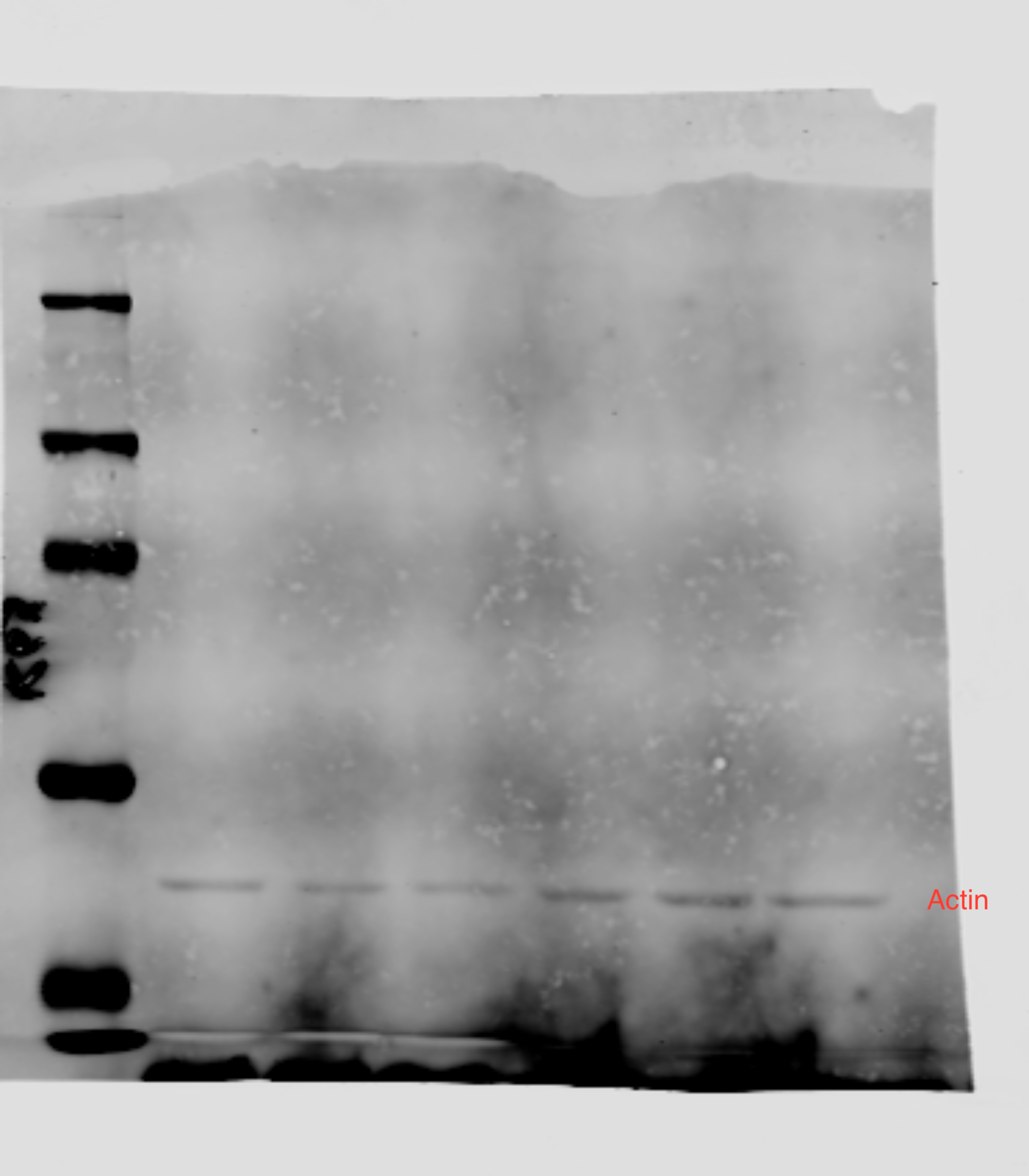

Supplement: Supplementary file 6 — Source Data Fig. 3 [file 44318_2024_44_MOESM6_ESM.zip › Fig 3/Fig 3A/Fig3A_K63_actin.tif]

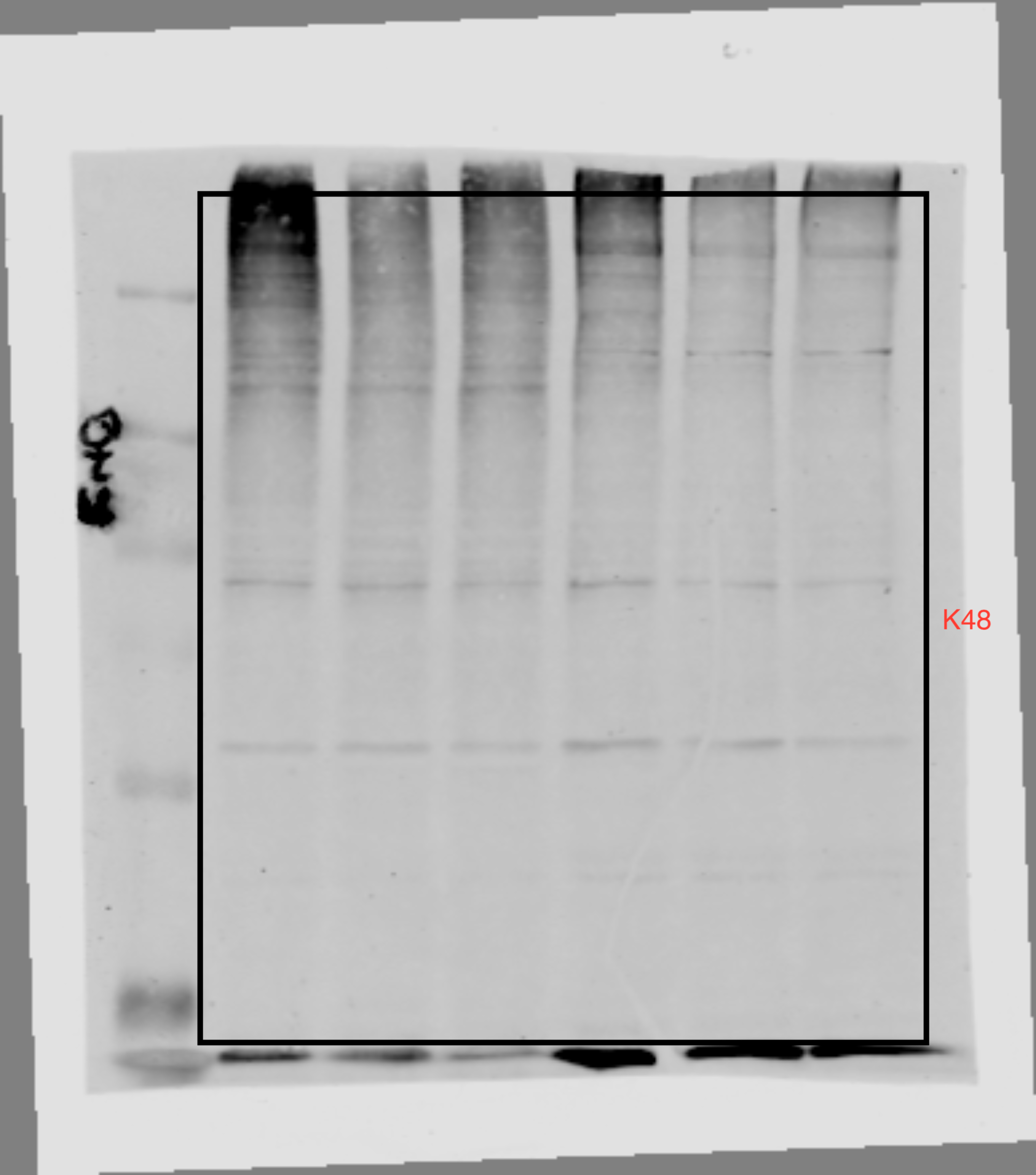

Supplement: Supplementary file 6 — Source Data Fig. 3 [file 44318_2024_44_MOESM6_ESM.zip › Fig 3/Fig 3A/Fig3A_K48.tif]

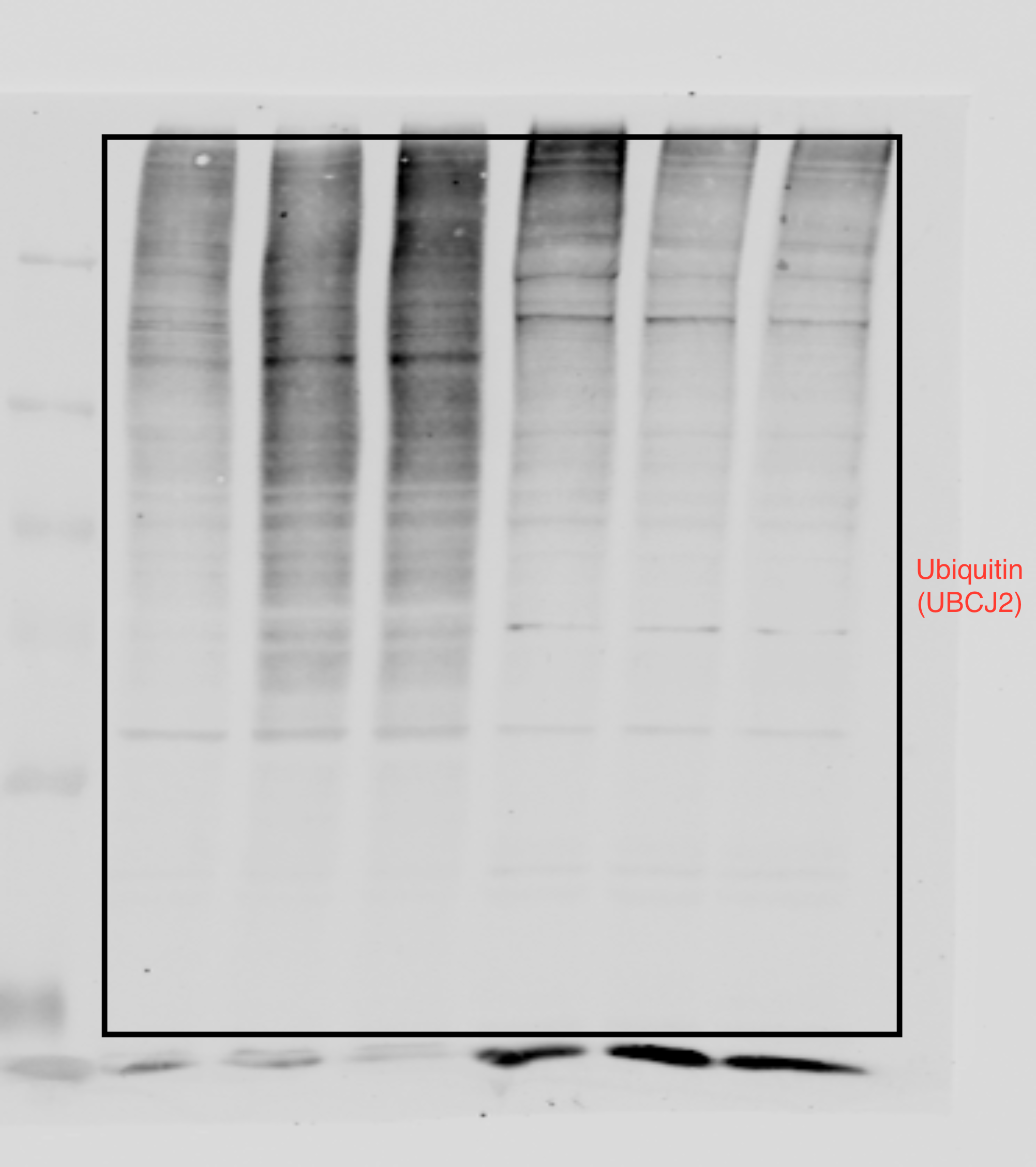

Supplement: Supplementary file 6 — Source Data Fig. 3 [file 44318_2024_44_MOESM6_ESM.zip › Fig 3/Fig 3A/Fig3A_ubiquitin.tif]

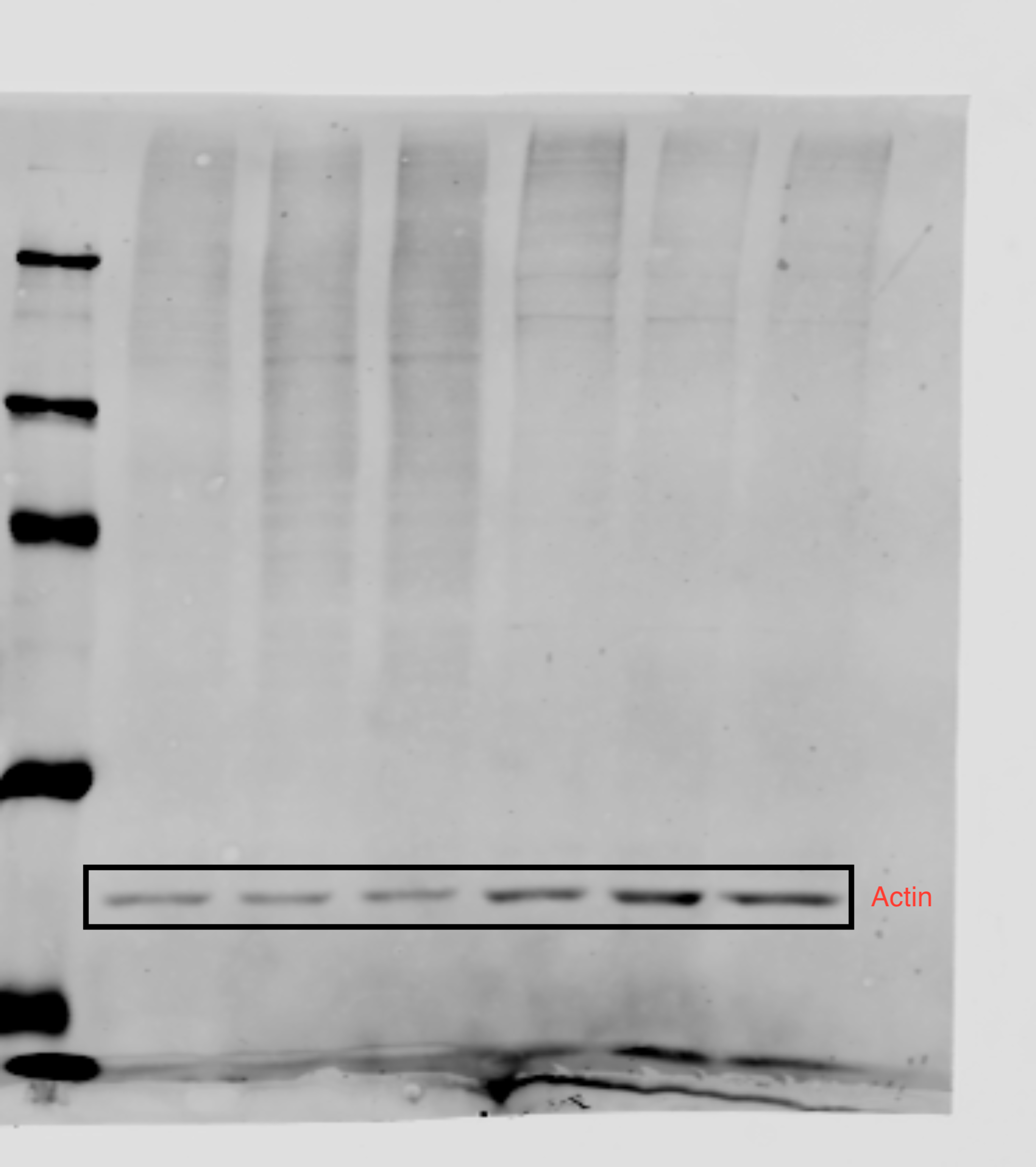

Supplement: Supplementary file 6 — Source Data Fig. 3 [file 44318_2024_44_MOESM6_ESM.zip › Fig 3/Fig 3A/Fig3A_ubiquitin_actin.tif]

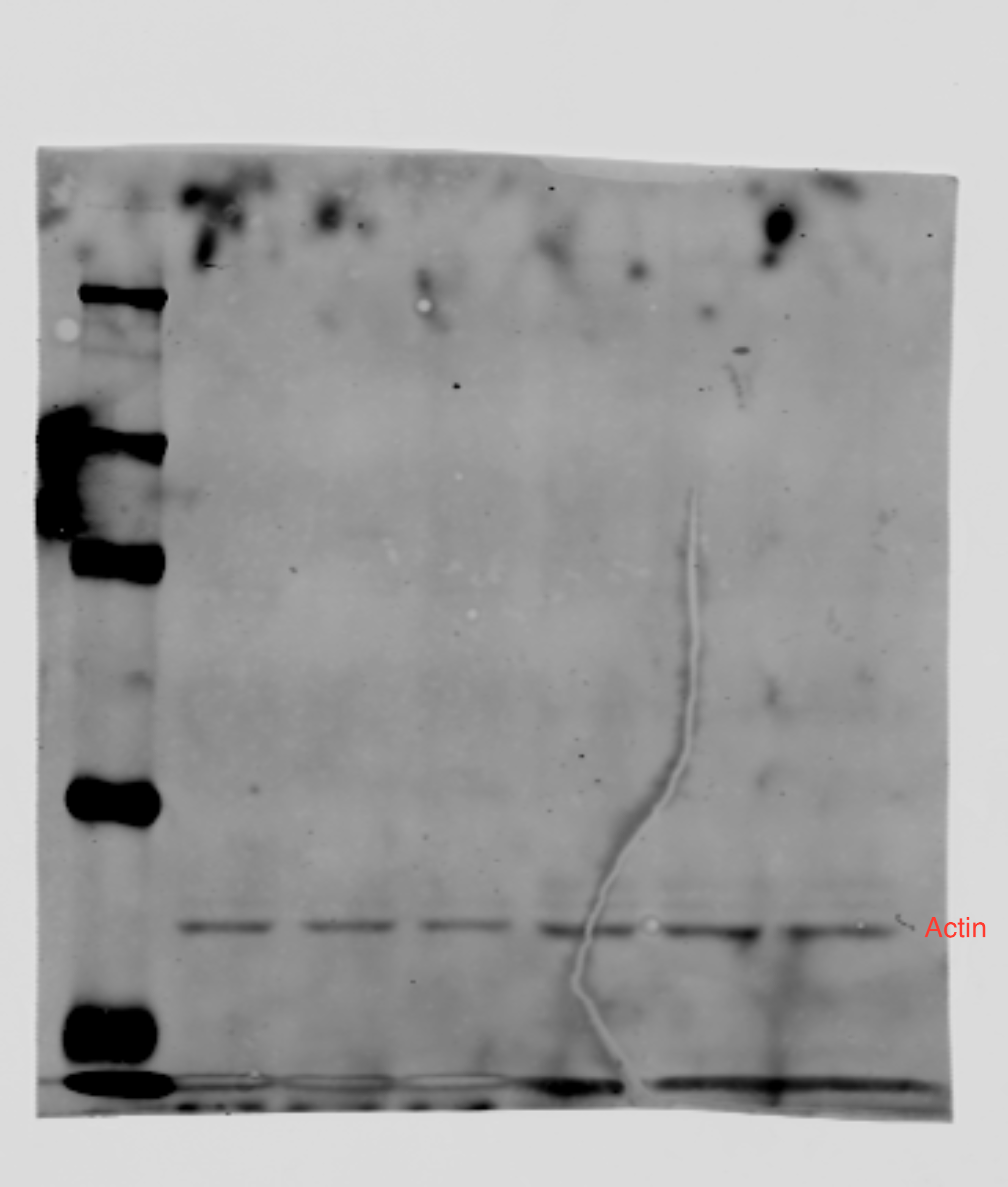

Supplement: Supplementary file 6 — Source Data Fig. 3 [file 44318_2024_44_MOESM6_ESM.zip › Fig 3/Fig 3A/Fig3A_K48_actin.tif]

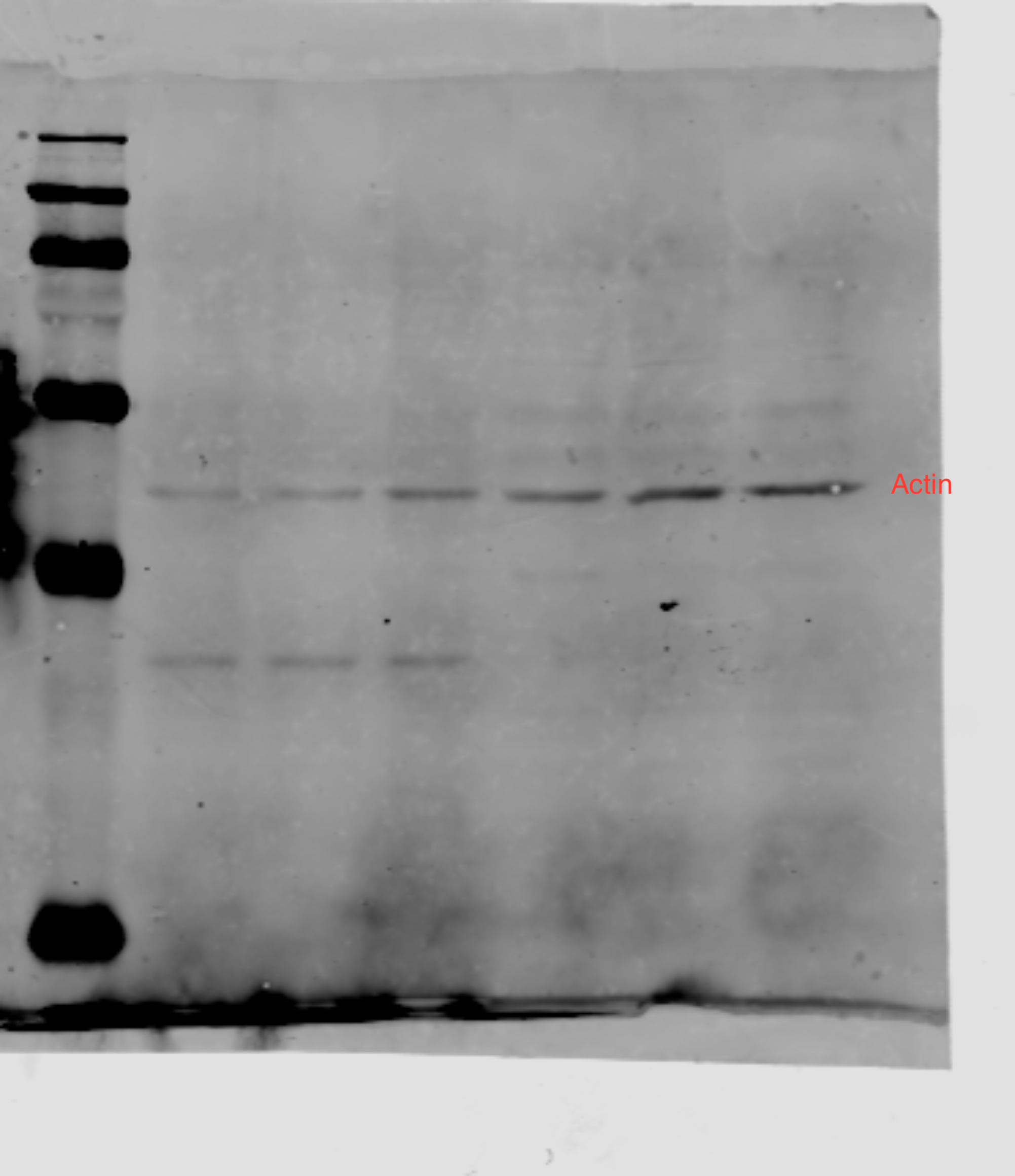

Supplement: Supplementary file 6 — Source Data Fig. 3 [file 44318_2024_44_MOESM6_ESM.zip › Fig 3/Fig 3A/Fig3A_TOM20_actin.tif]

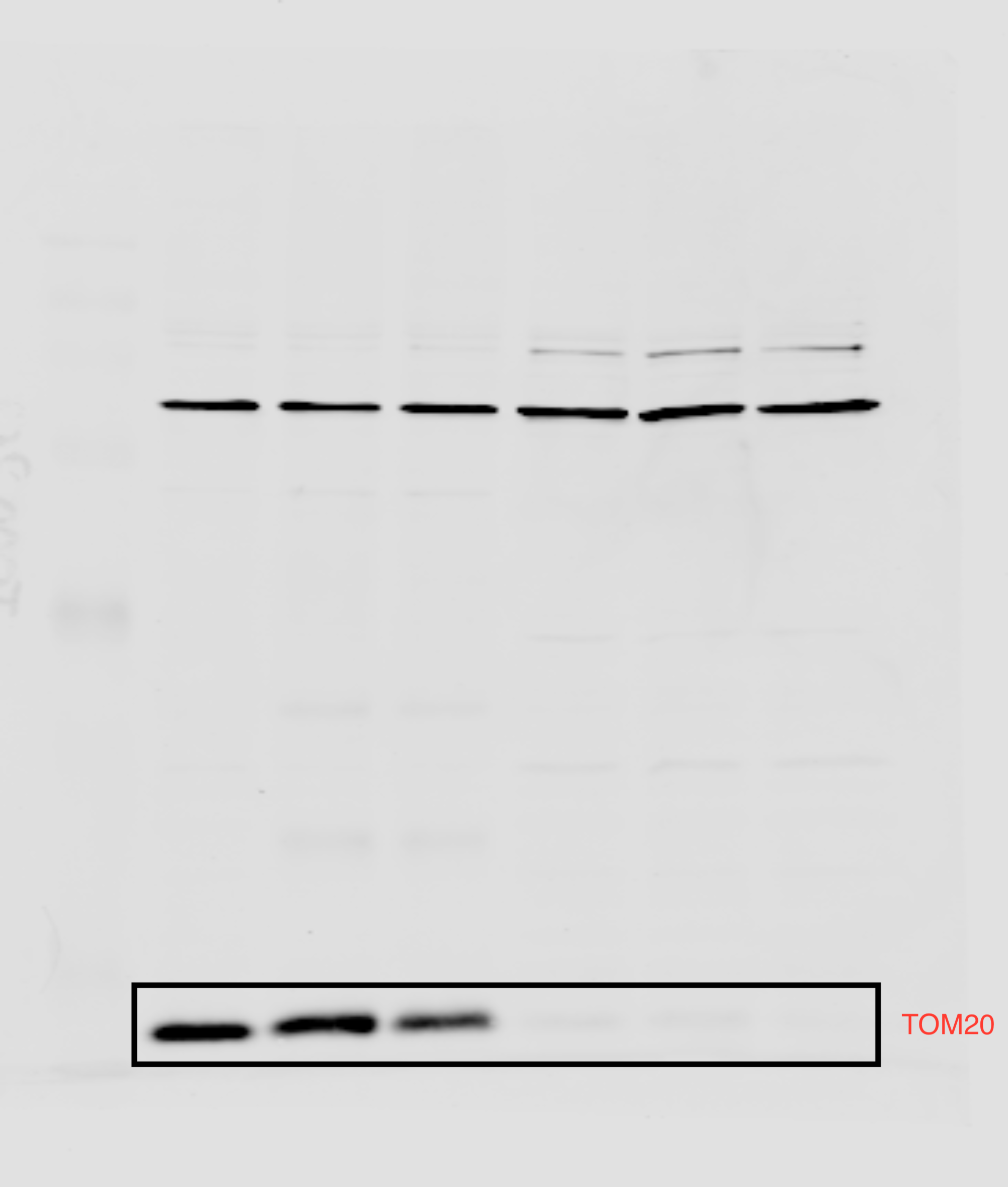

Supplement: Supplementary file 6 — Source Data Fig. 3 [file 44318_2024_44_MOESM6_ESM.zip › Fig 3/Fig 3A/Fig3A_TOM20.tif]

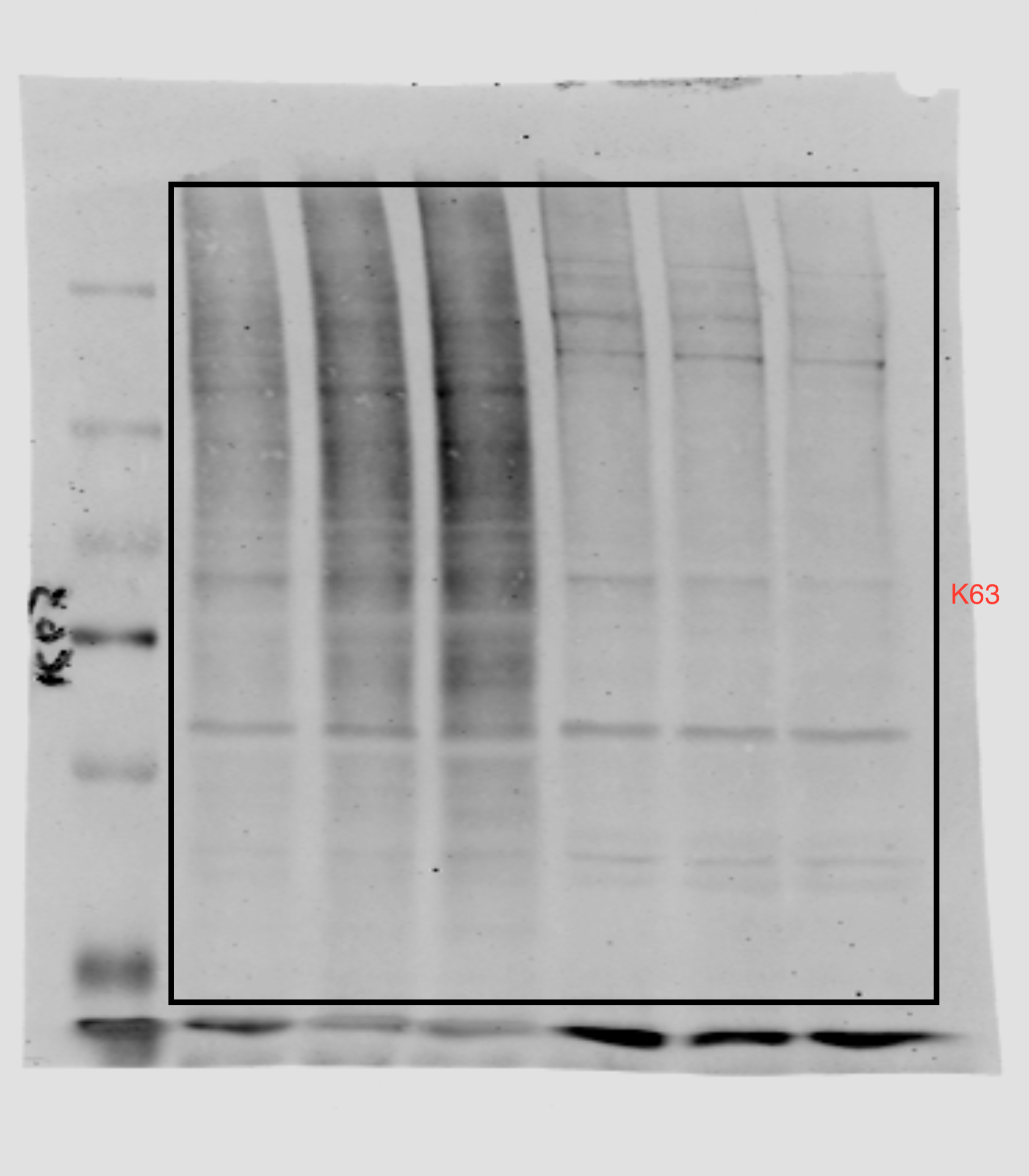

Supplement: Supplementary file 6 — Source Data Fig. 3 [file 44318_2024_44_MOESM6_ESM.zip › Fig 3/Fig 3A/Fig3A_K63.tif]

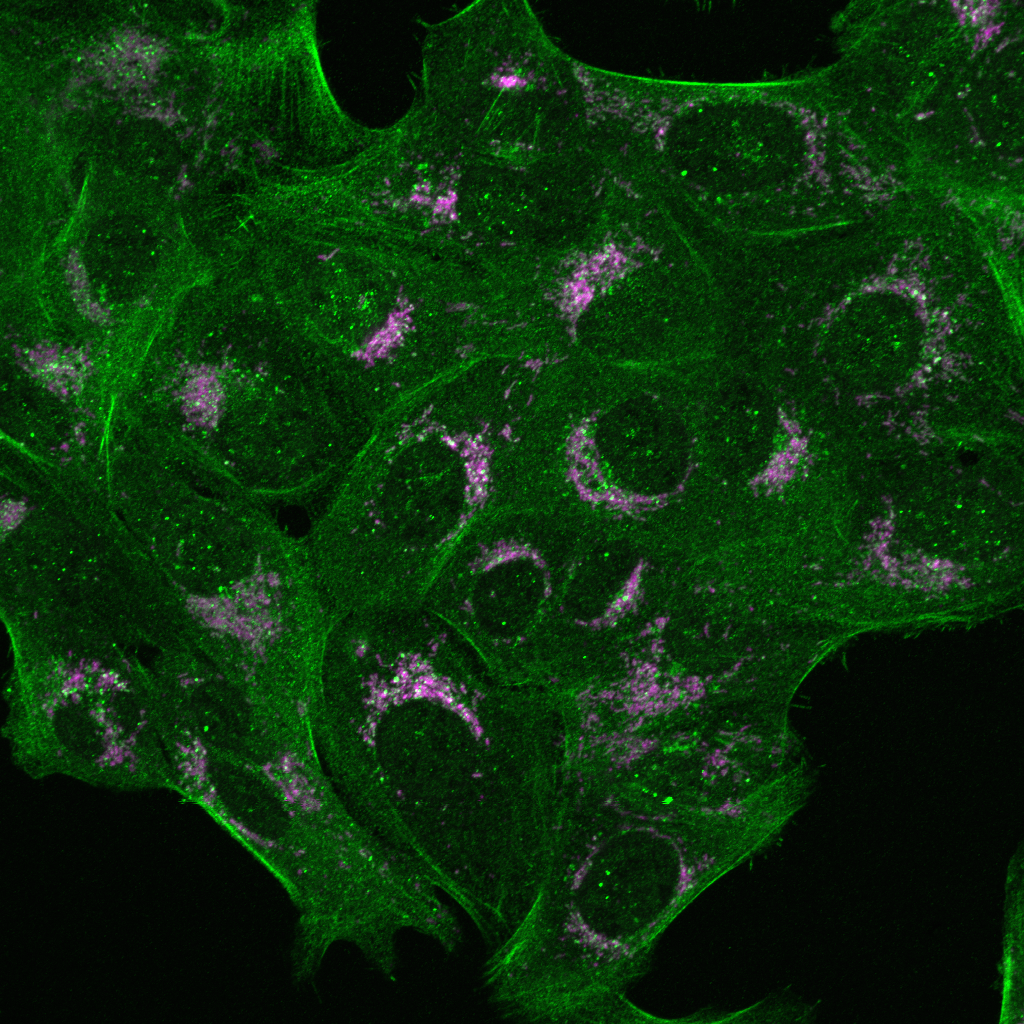

Supplement: Supplementary file 6 — Source Data Fig. 3 [file 44318_2024_44_MOESM6_ESM.zip › Fig 3/Fig 3B/Fig3B_Uncropped_MAX_U20S empty K63 COXIV cicd.tif (RGB).tif]

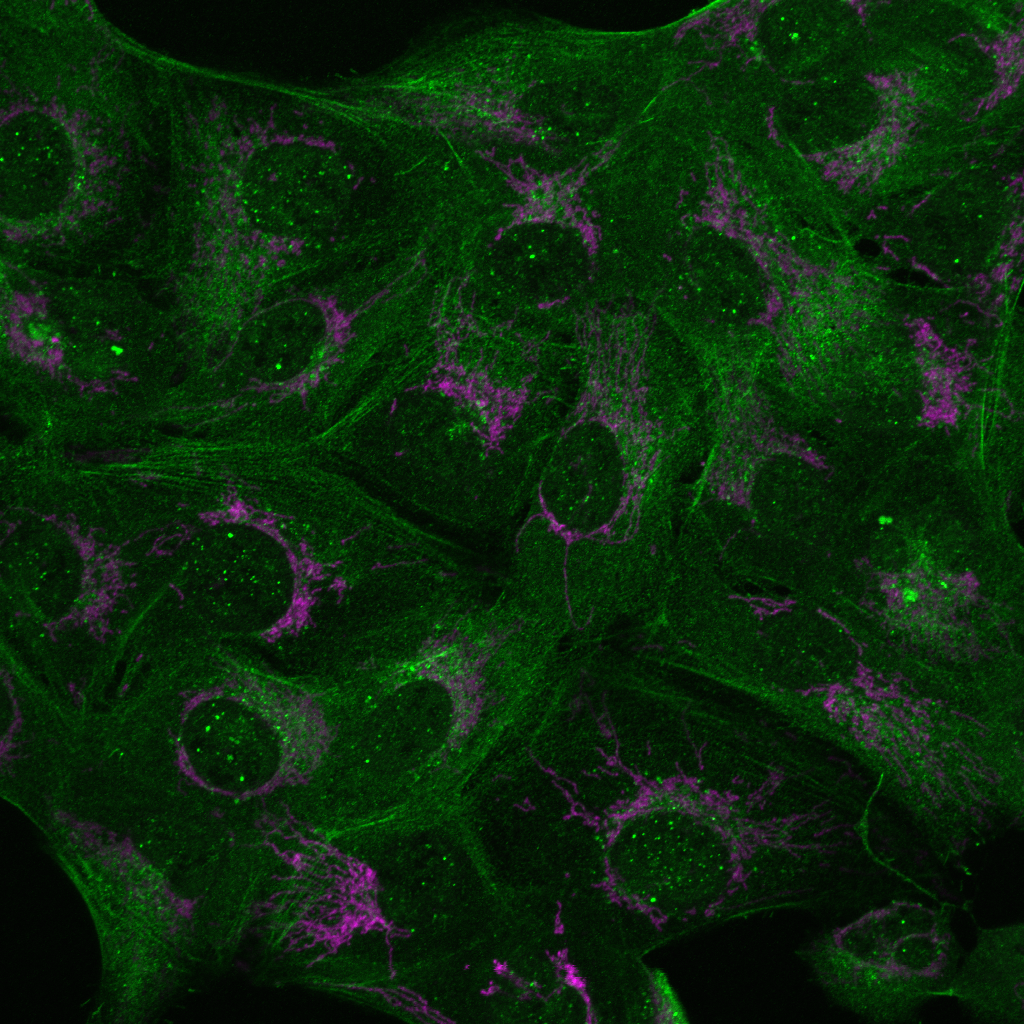

Supplement: Supplementary file 6 — Source Data Fig. 3 [file 44318_2024_44_MOESM6_ESM.zip › Fig 3/Fig 3B/Fig3B_Uncropped_MAX_U20S empty K63 COXIV ctrl.tif (RGB).tif]

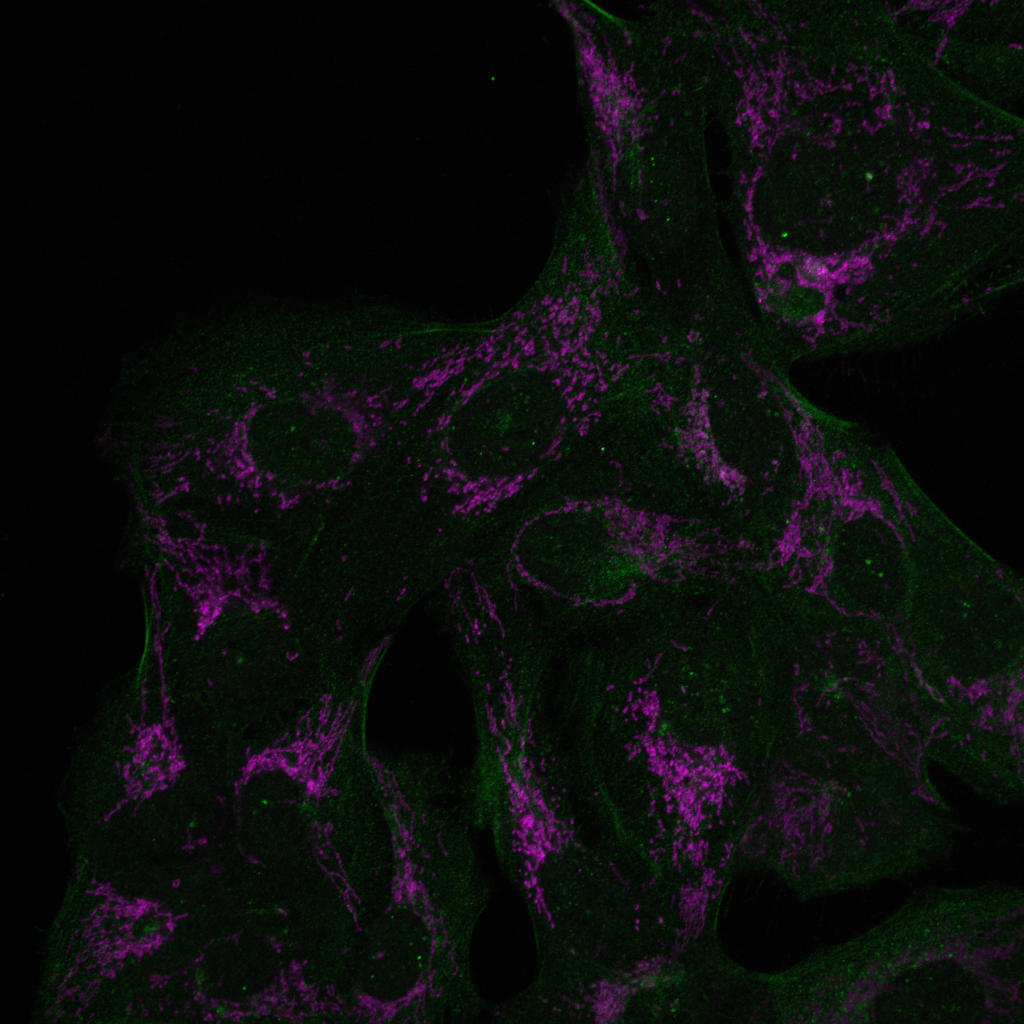

Supplement: Supplementary file 6 — Source Data Fig. 3 [file 44318_2024_44_MOESM6_ESM.zip › Fig 3/Fig 3B/Fig3B_Uncropped_MAX_U20S bb K63 COXIV cicd (RGB).tif]

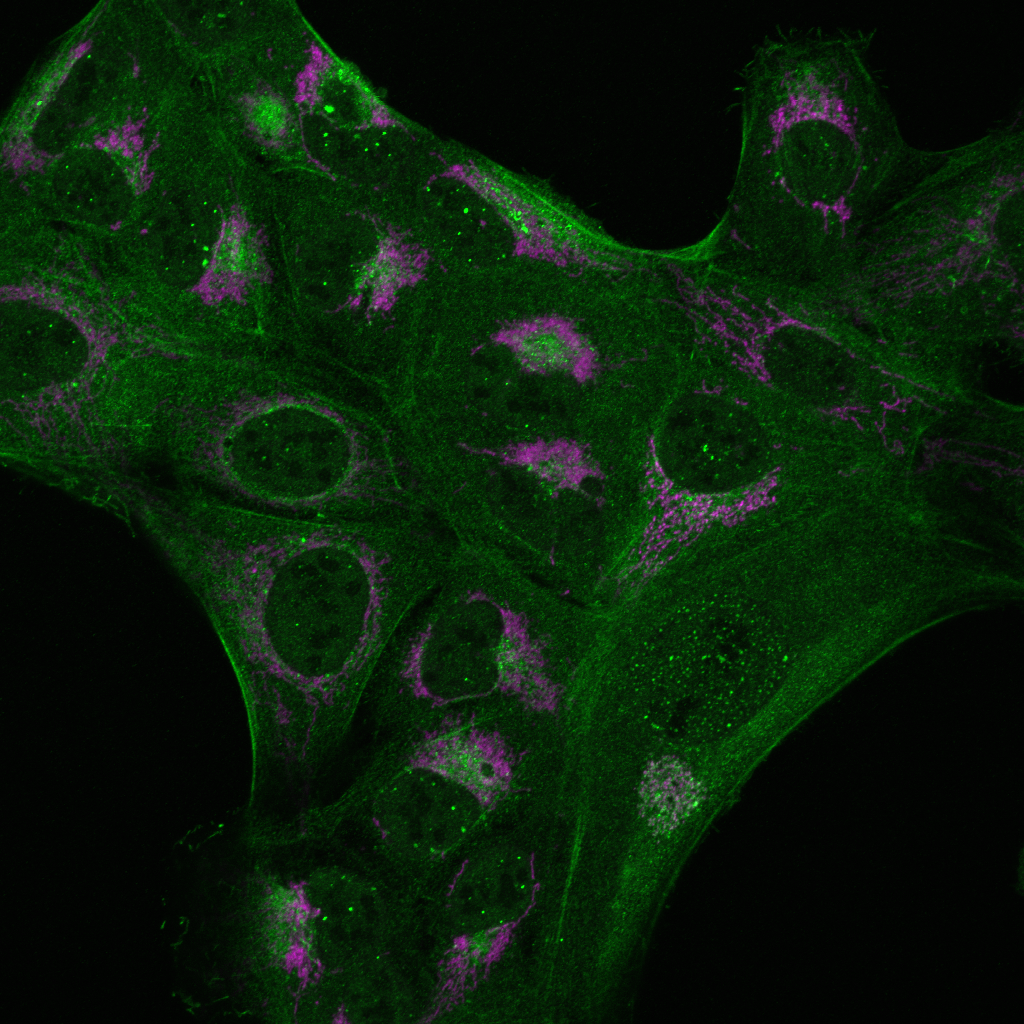

Supplement: Supplementary file 6 — Source Data Fig. 3 [file 44318_2024_44_MOESM6_ESM.zip › Fig 3/Fig 3B/Fig3B_Uncropped_MAX_ U20S bb K63 COXIV ctrl (RGB).tif]

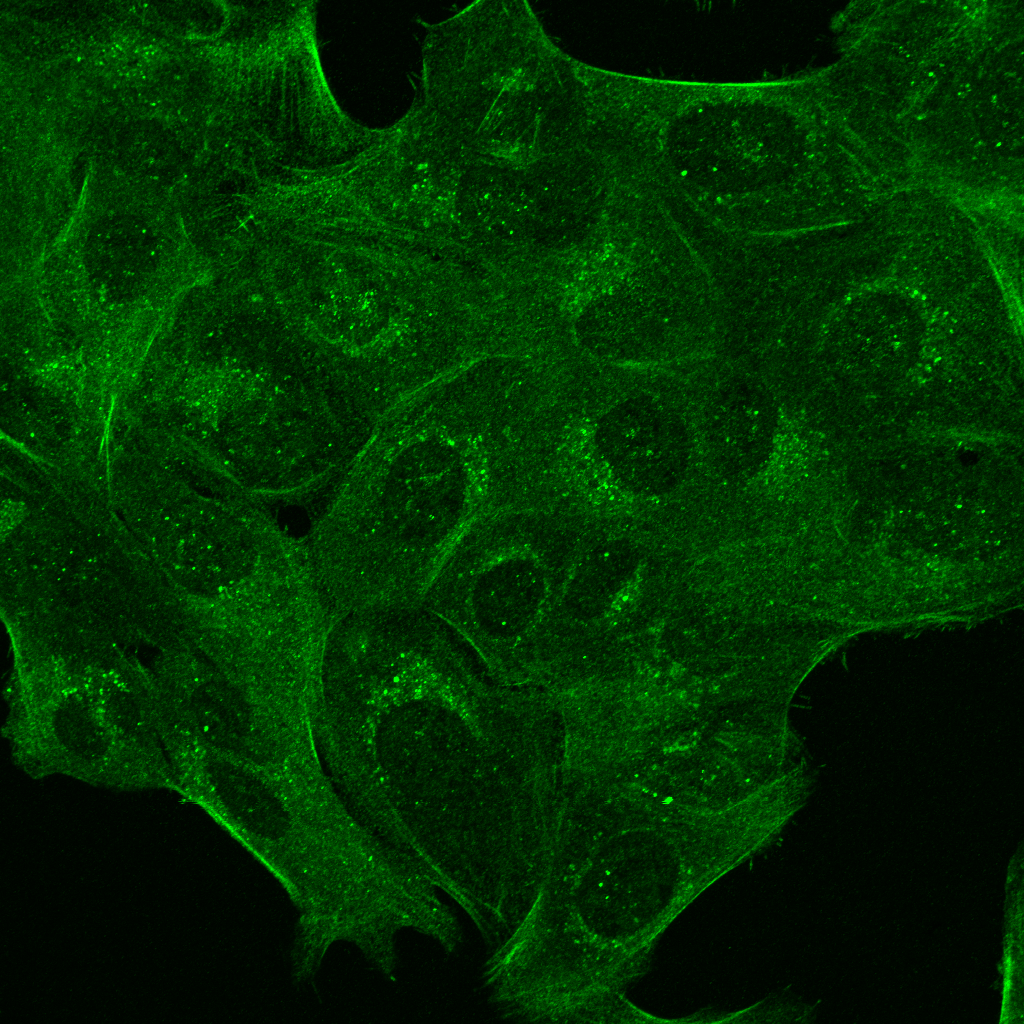

Supplement: Supplementary file 6 — Source Data Fig. 3 [file 44318_2024_44_MOESM6_ESM.zip › Fig 3/Fig 3B/Single channel/Fig3B_Uncropped_MAX_U20S empty K63 COXIV cicd (RGB)_K63ub(green).tif]

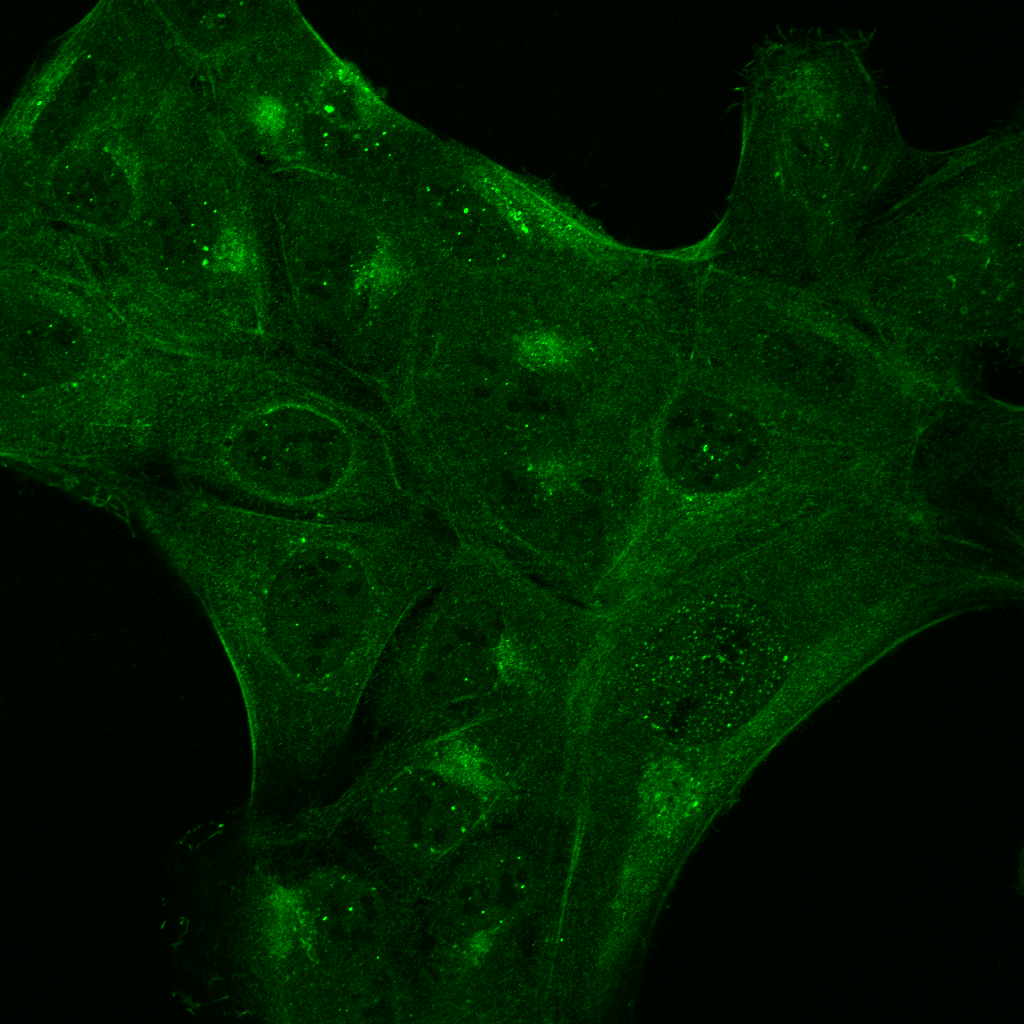

Supplement: Supplementary file 6 — Source Data Fig. 3 [file 44318_2024_44_MOESM6_ESM.zip › Fig 3/Fig 3B/Single channel/Fig3B_Uncropped_MAX_U20S bb K63 COXIV ctrl (RGB)_K63ub(green).tif]

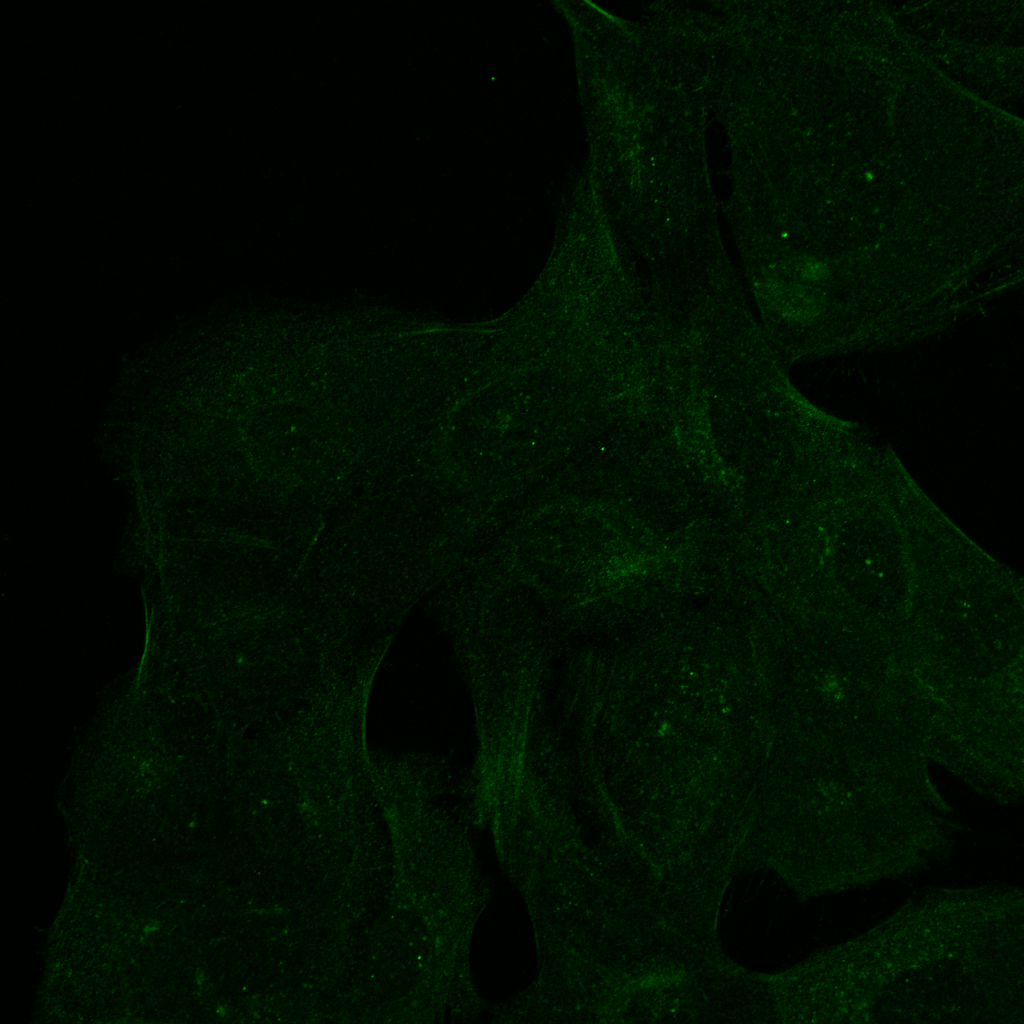

Supplement: Supplementary file 6 — Source Data Fig. 3 [file 44318_2024_44_MOESM6_ESM.zip › Fig 3/Fig 3B/Single channel/Fig3B_Uncropped_MAX_U20S bb K63 COXIV cicd.tif (RGB).tif_K63ub(green).tif]

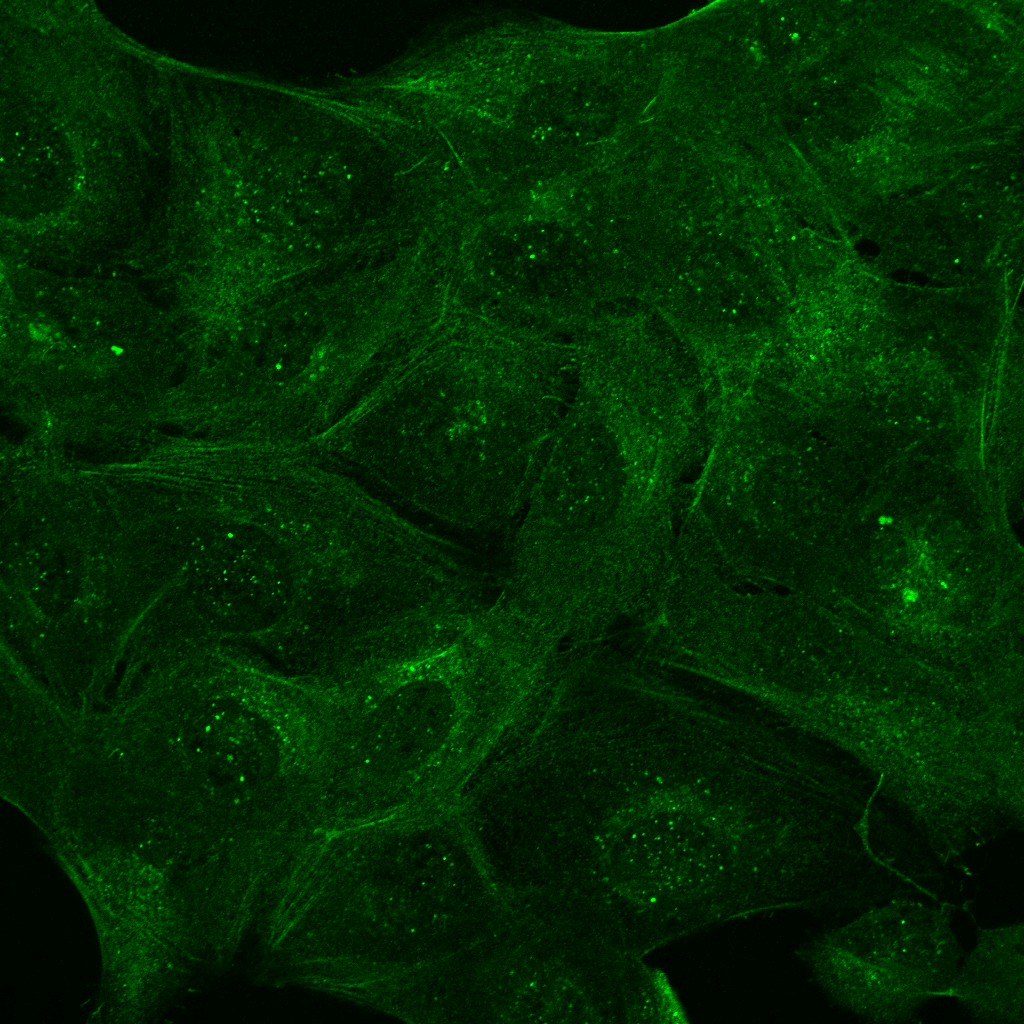

Supplement: Supplementary file 6 — Source Data Fig. 3 [file 44318_2024_44_MOESM6_ESM.zip › Fig 3/Fig 3B/Single channel/Fig3B_Uncropped_MAX_U20S empty K63 COXIV ctrl (RGB)_K63ub(green).tif]

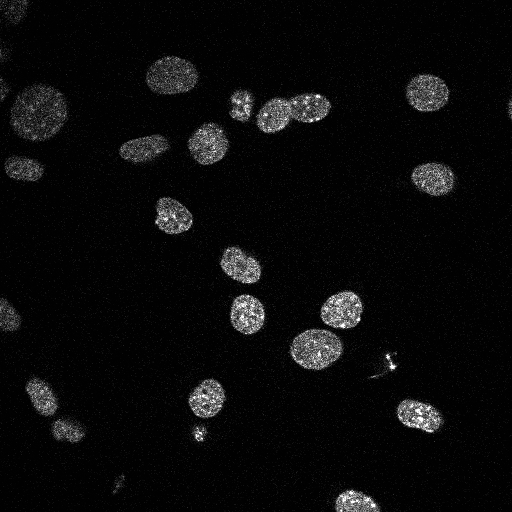

Supplement: Supplementary file 6 — Source Data Fig. 3 [file 44318_2024_44_MOESM6_ESM.zip › Fig 3/Fig 3D/TIFF/Fig_3D-SVEC M1-GFP tom20 cicd.tif]

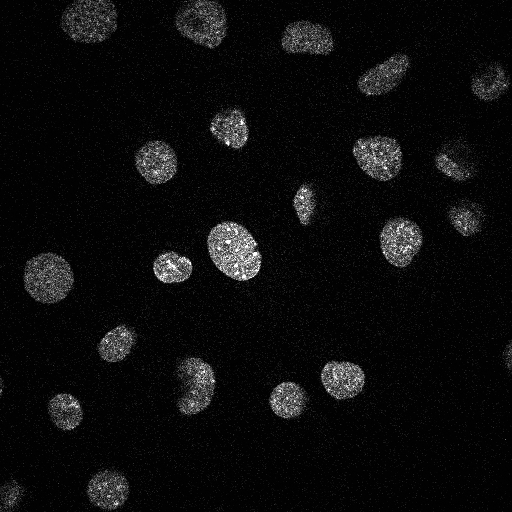

Supplement: Supplementary file 6 — Source Data Fig. 3 [file 44318_2024_44_MOESM6_ESM.zip › Fig 3/Fig 3D/TIFF/Fig_3D-SVEC K63-GFP tom20 cicd.tif]

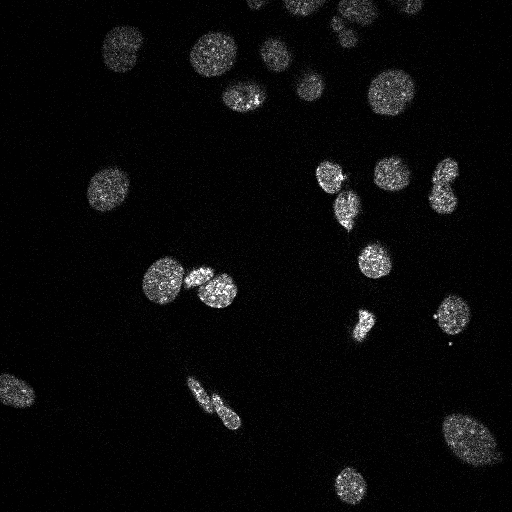

Supplement: Supplementary file 6 — Source Data Fig. 3 [file 44318_2024_44_MOESM6_ESM.zip › Fig 3/Fig 3D/TIFF/Fig_3D-SVEC GFP tom20 cicd.tif]

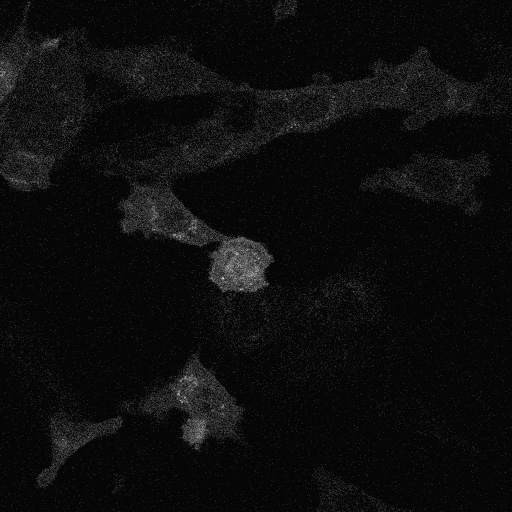

Supplement: Supplementary file 6 — Source Data Fig. 3 [file 44318_2024_44_MOESM6_ESM.zip › Fig 3/Fig 3D/TIFF/Single Channel/Fig_3D-SVEC M1-GFP tom20 cicd_GFP.tif]

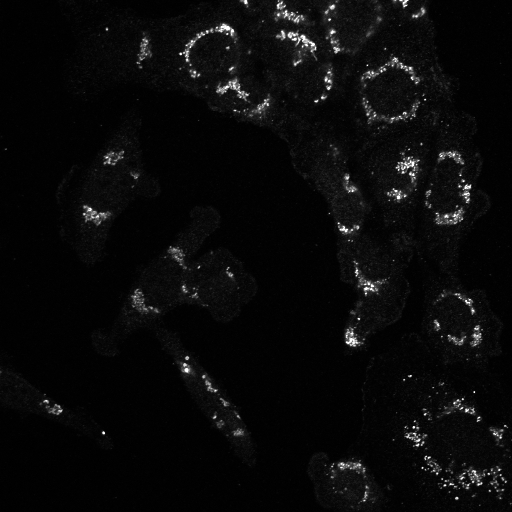

Supplement: Supplementary file 6 — Source Data Fig. 3 [file 44318_2024_44_MOESM6_ESM.zip › Fig 3/Fig 3D/TIFF/Single Channel/Fig_3D-SVEC GFP tom20 cicd_Tom20.tif]

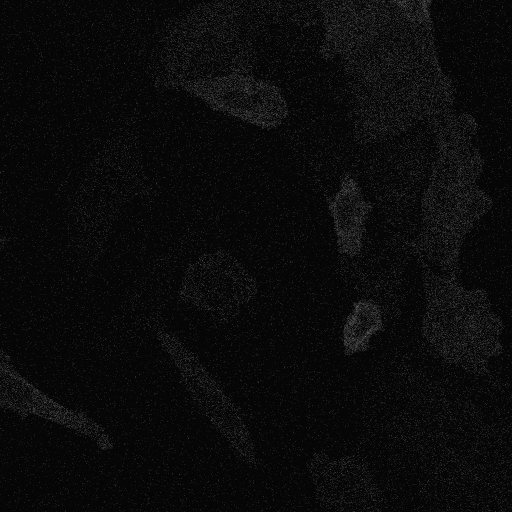

Supplement: Supplementary file 6 — Source Data Fig. 3 [file 44318_2024_44_MOESM6_ESM.zip › Fig 3/Fig 3D/TIFF/Single Channel/Fig_3D-SVEC GFP tom20 cicd_GFP.tif]

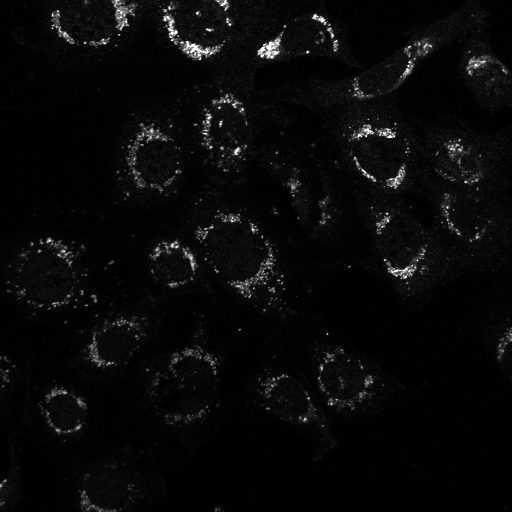

Supplement: Supplementary file 6 — Source Data Fig. 3 [file 44318_2024_44_MOESM6_ESM.zip › Fig 3/Fig 3D/TIFF/Single Channel/Fig_3D-SVEC K63-GFP tom20 cicd_TOM20.tif]

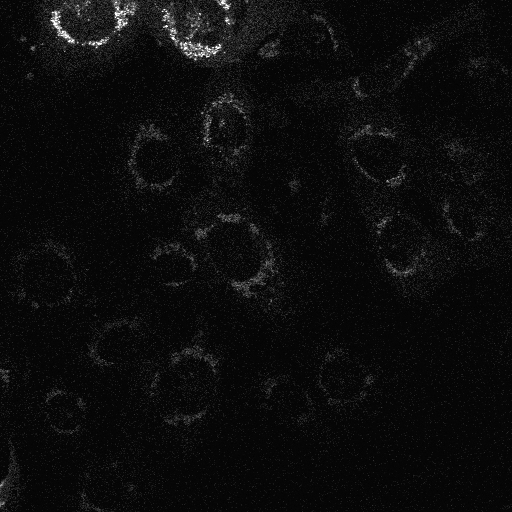

Supplement: Supplementary file 6 — Source Data Fig. 3 [file 44318_2024_44_MOESM6_ESM.zip › Fig 3/Fig 3D/TIFF/Single Channel/Fig_3D-SVEC K63-GFP tom20 cicd_GFP.tif]

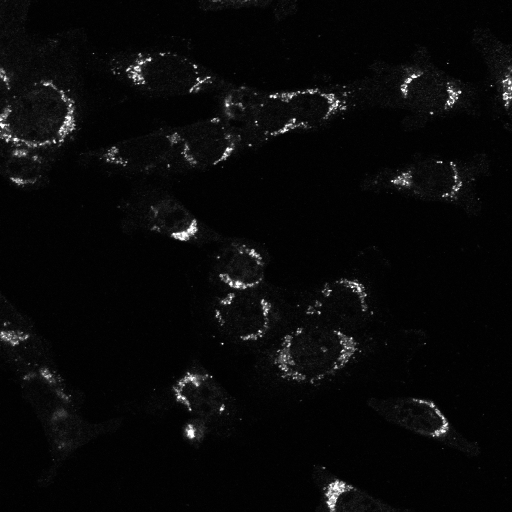

Supplement: Supplementary file 6 — Source Data Fig. 3 [file 44318_2024_44_MOESM6_ESM.zip › Fig 3/Fig 3D/TIFF/Single Channel/Fig_3D-SVEC M1-GFP tom20 cicd_TOM20.tif]

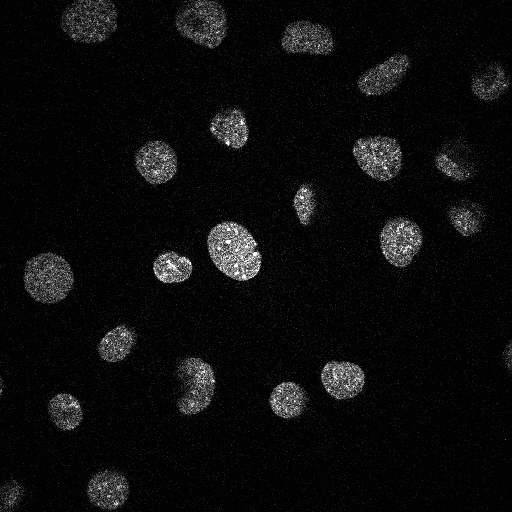

Supplement: Supplementary file 6 — Source Data Fig. 3 [file 44318_2024_44_MOESM6_ESM.zip › Fig 3/Fig 3D/TIFF/Single Channel/Fig_3D-SVEC K63-GFP tom20 cicd_Dapi.tif]

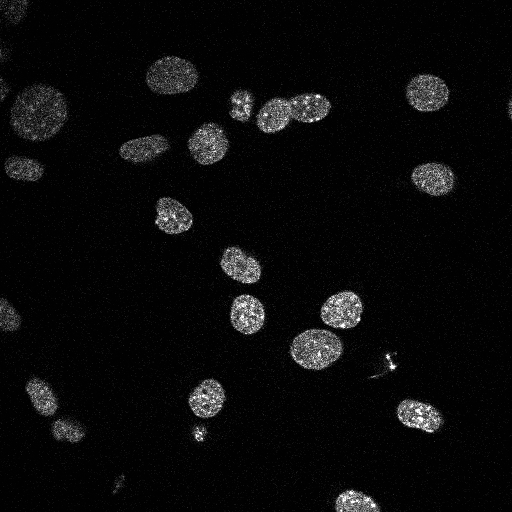

Supplement: Supplementary file 6 — Source Data Fig. 3 [file 44318_2024_44_MOESM6_ESM.zip › Fig 3/Fig 3D/TIFF/Single Channel/Fig_3D-SVEC M1-GFP tom20 cicd_Dapi.tif]

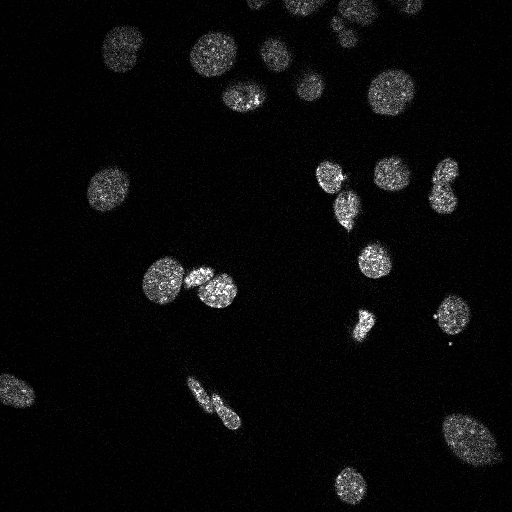

Supplement: Supplementary file 6 — Source Data Fig. 3 [file 44318_2024_44_MOESM6_ESM.zip › Fig 3/Fig 3D/TIFF/Single Channel/Fig_3D-SVEC GFP tom20 cicd_Dapi.tif]

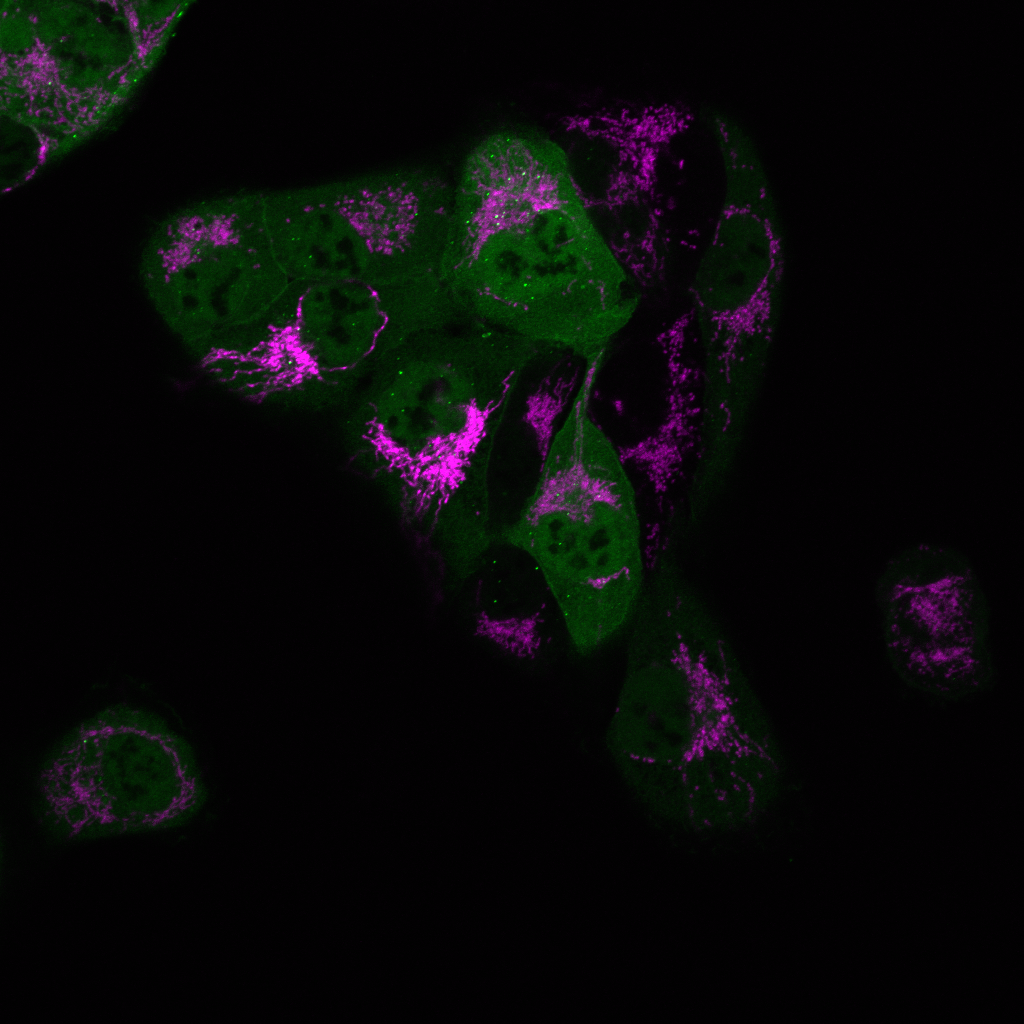

Supplement: Supplementary file 7 — Source Data Fig. 4 [file 44318_2024_44_MOESM7_ESM.zip › Fig 4/Fig 4D/Fig4D_Uncropped_MAX_ U20S BB CICD COXIV (RGB).tif]

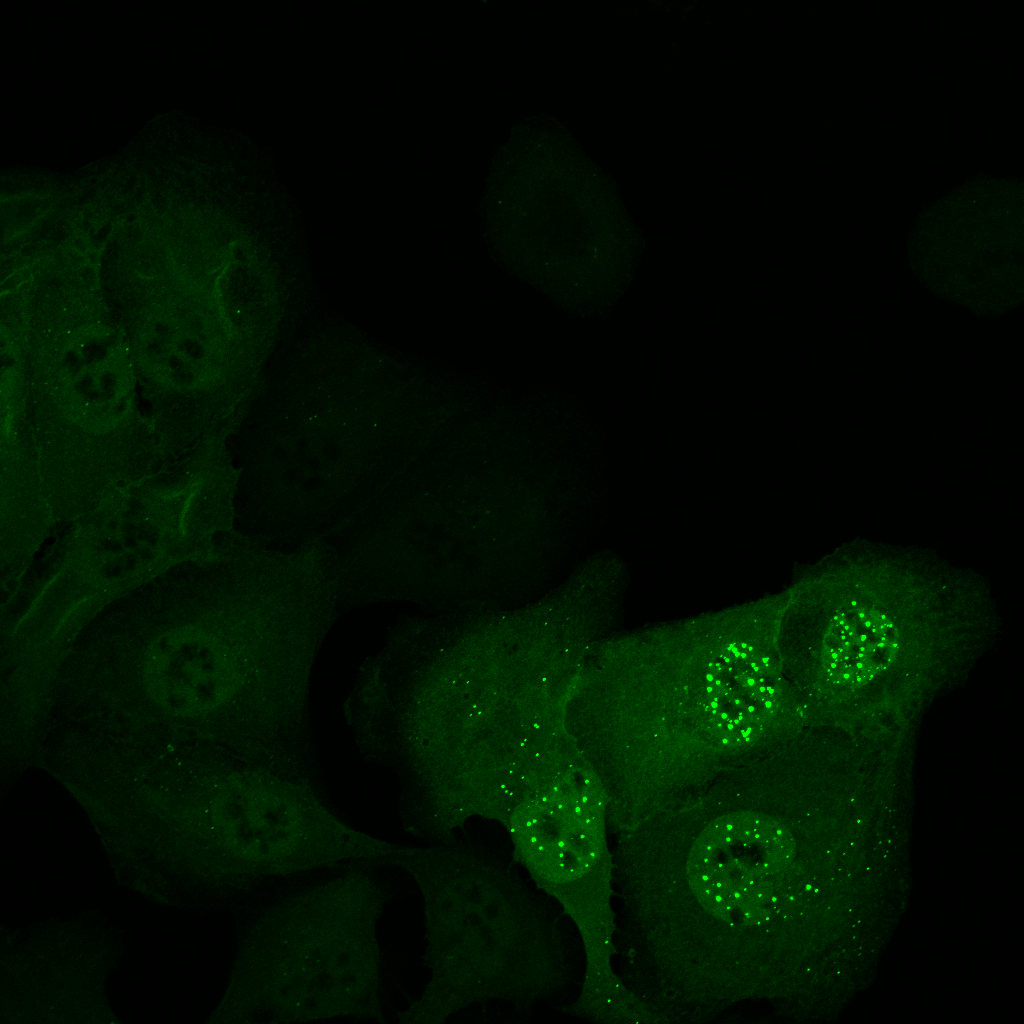

Supplement: Supplementary file 7 — Source Data Fig. 4 [file 44318_2024_44_MOESM7_ESM.zip › Fig 4/Fig 4D/Fig4D_Uncropped_MAX_U20S BB CTRL COXIV (RGB).tif]

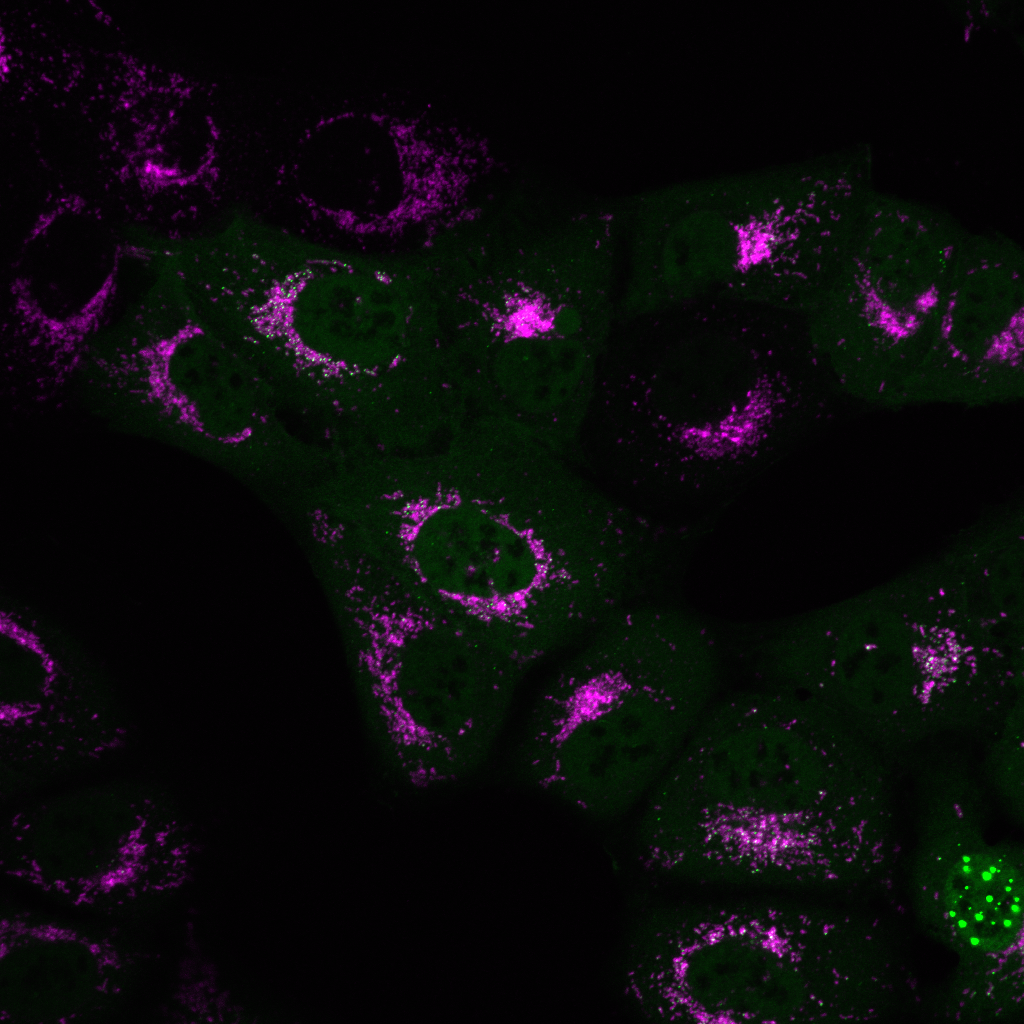

Supplement: Supplementary file 7 — Source Data Fig. 4 [file 44318_2024_44_MOESM7_ESM.zip › Fig 4/Fig 4D/Fig4D_Uncropped_MAX_U20S EMPTY CICD COXIV (RGB).tif]

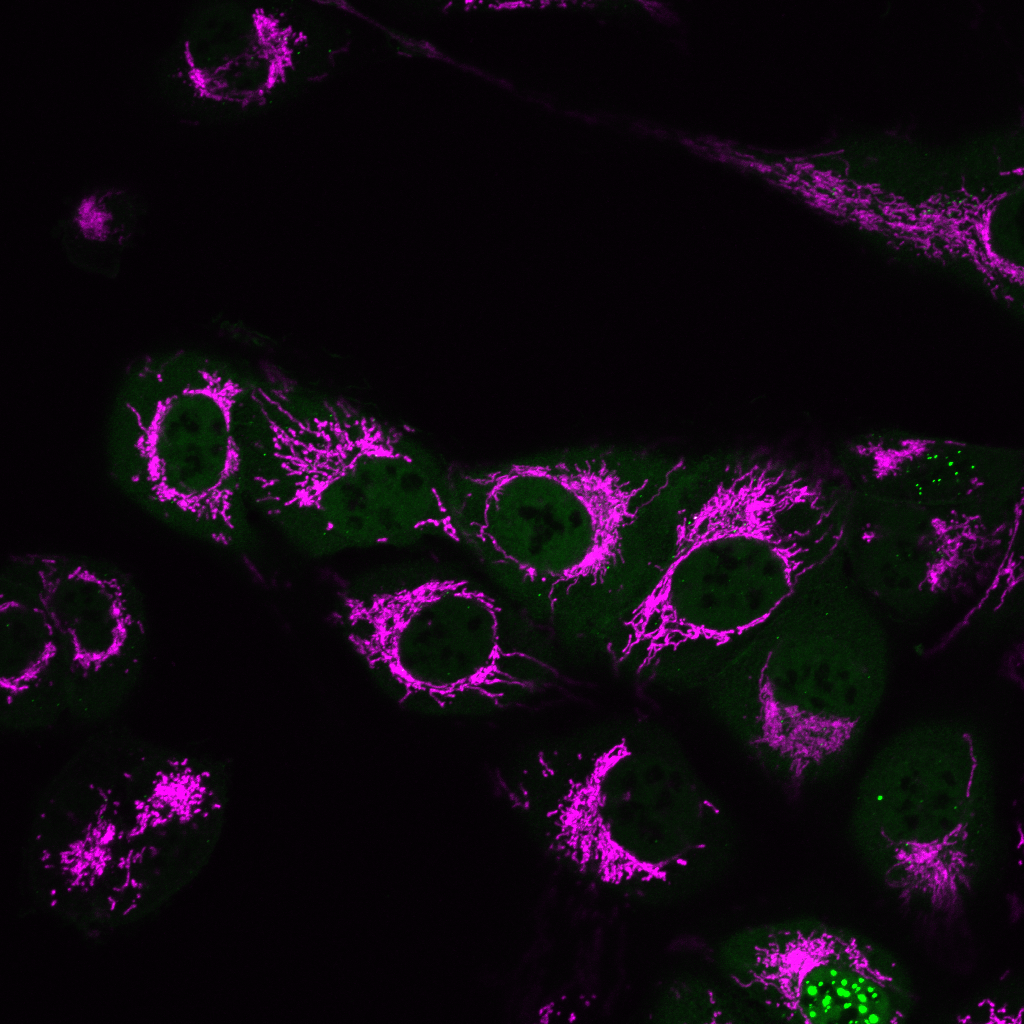

Supplement: Supplementary file 7 — Source Data Fig. 4 [file 44318_2024_44_MOESM7_ESM.zip › Fig 4/Fig 4D/Fig4D_Uncropped_MAX_U20S EMPTY CTRL COXIV (RGB).tif]

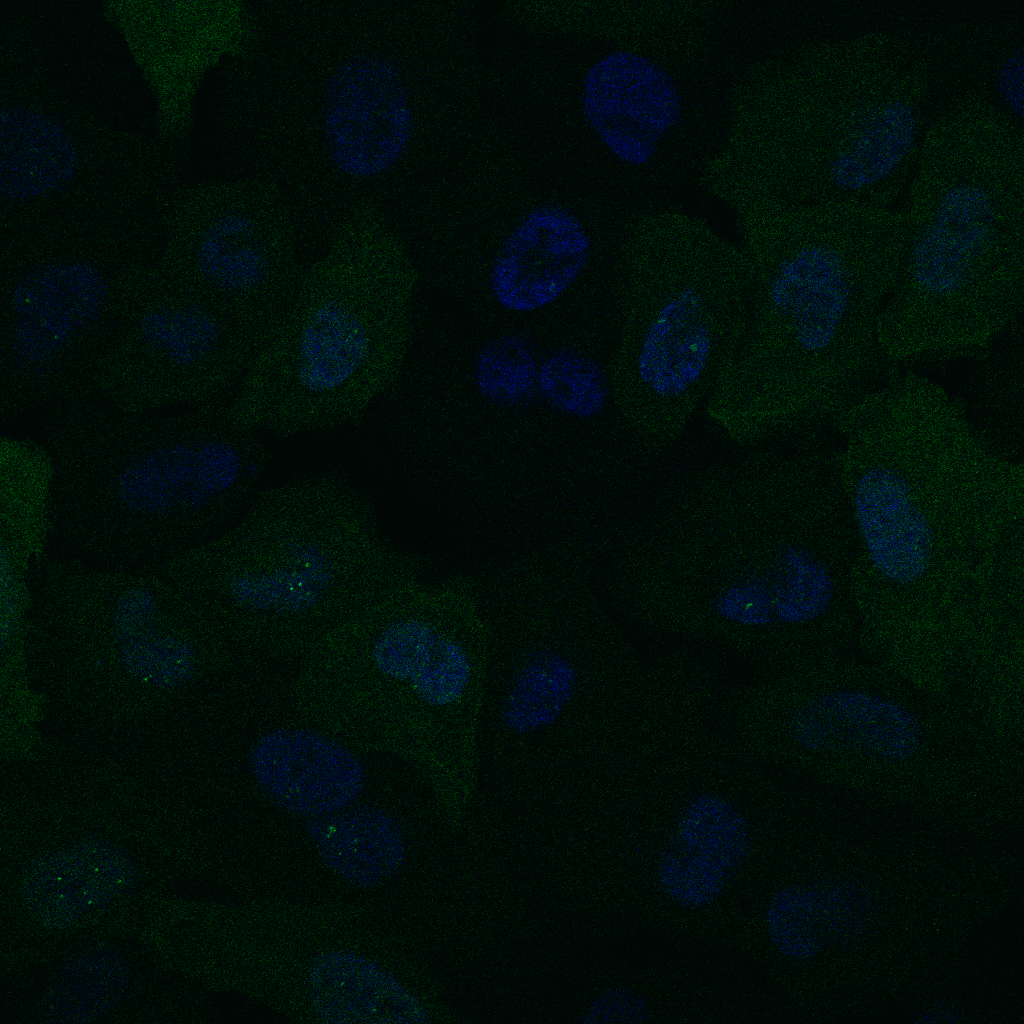

Supplement: Supplementary file 7 — Source Data Fig. 4 [file 44318_2024_44_MOESM7_ESM.zip › Fig 4/Fig 4F/Fig4F_DAPI_GFP_uncropped_U2OS GFP-NEMOD311N TOM20 3hr cicd (RGB).tif]

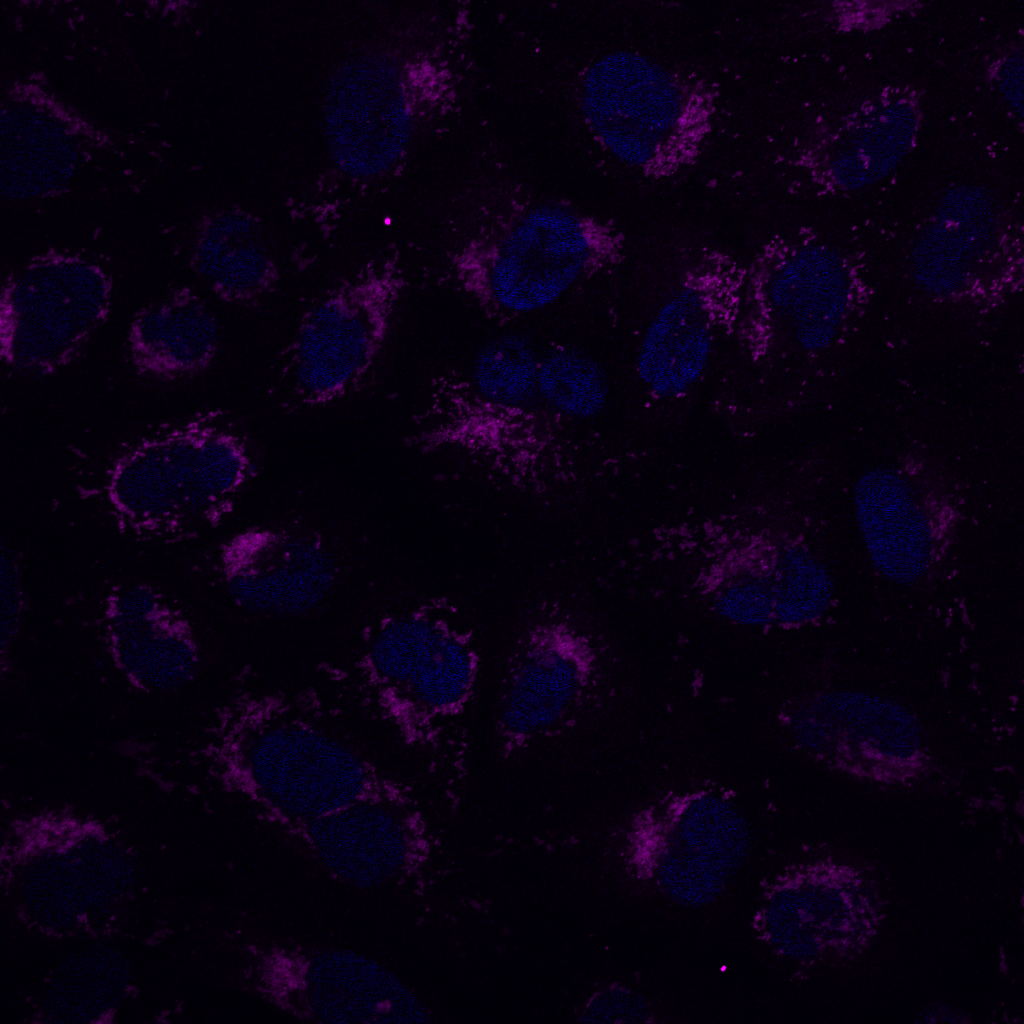

Supplement: Supplementary file 7 — Source Data Fig. 4 [file 44318_2024_44_MOESM7_ESM.zip › Fig 4/Fig 4F/Fig4F_DAPI_TOM20_uncropped_U2OS GFP-NEMOD311N TOM20 3hr cicd (RGB).tif]

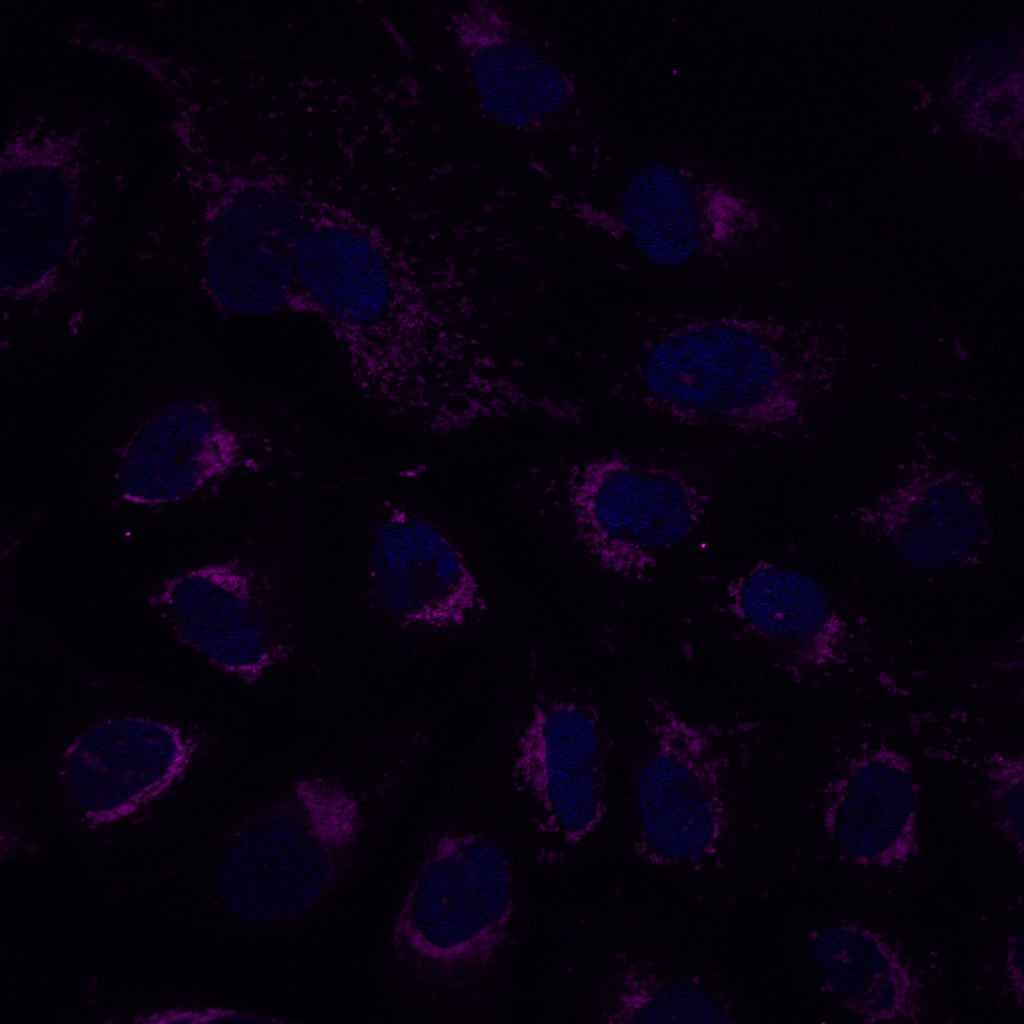

Supplement: Supplementary file 7 — Source Data Fig. 4 [file 44318_2024_44_MOESM7_ESM.zip › Fig 4/Fig 4F/Fig4F_DAPI_TOM20_uncropped_U2OS GFP-NEMOZF TOM20 3hr cicd (RGB).tif]

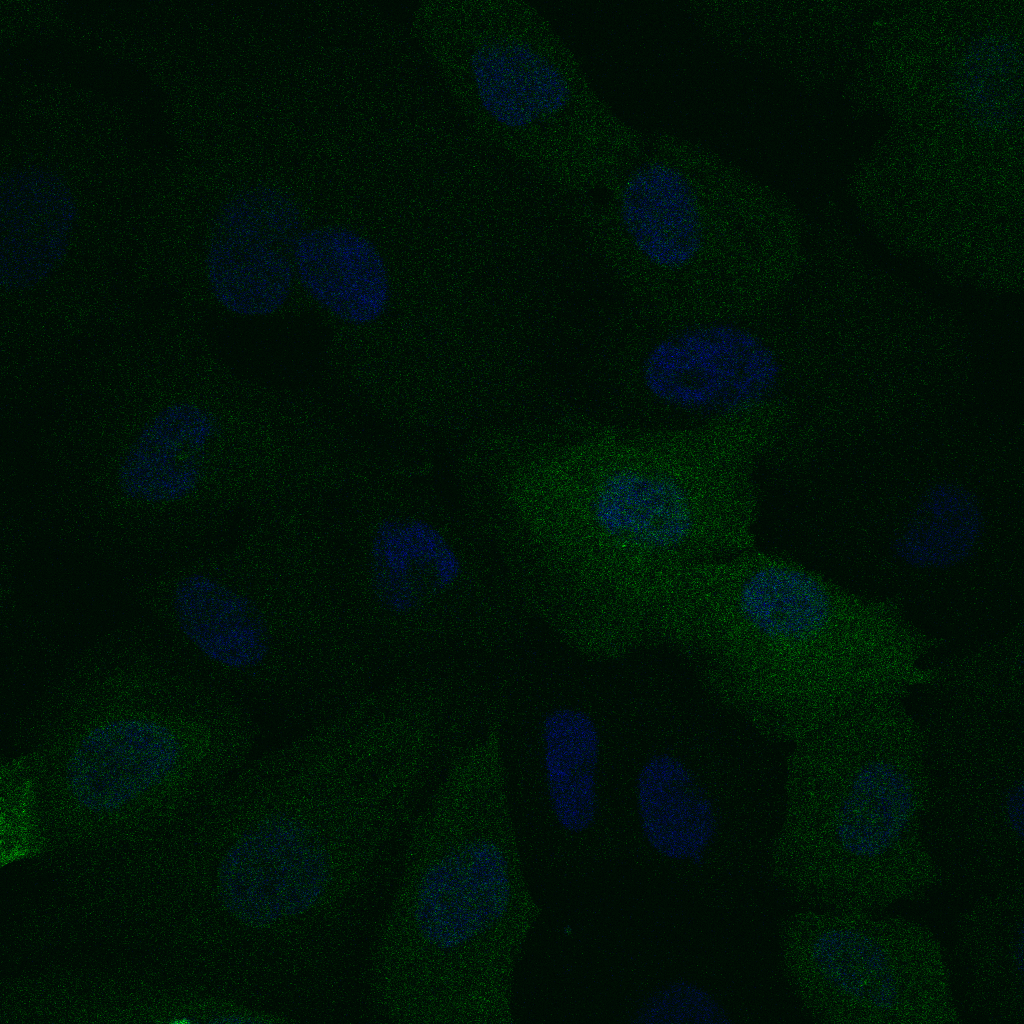

Supplement: Supplementary file 7 — Source Data Fig. 4 [file 44318_2024_44_MOESM7_ESM.zip › Fig 4/Fig 4F/Fig4F_DAPI_GFP_uncropped_U2OS GFP-NEMOZF TOM20 3hr cicd (RGB).tif]

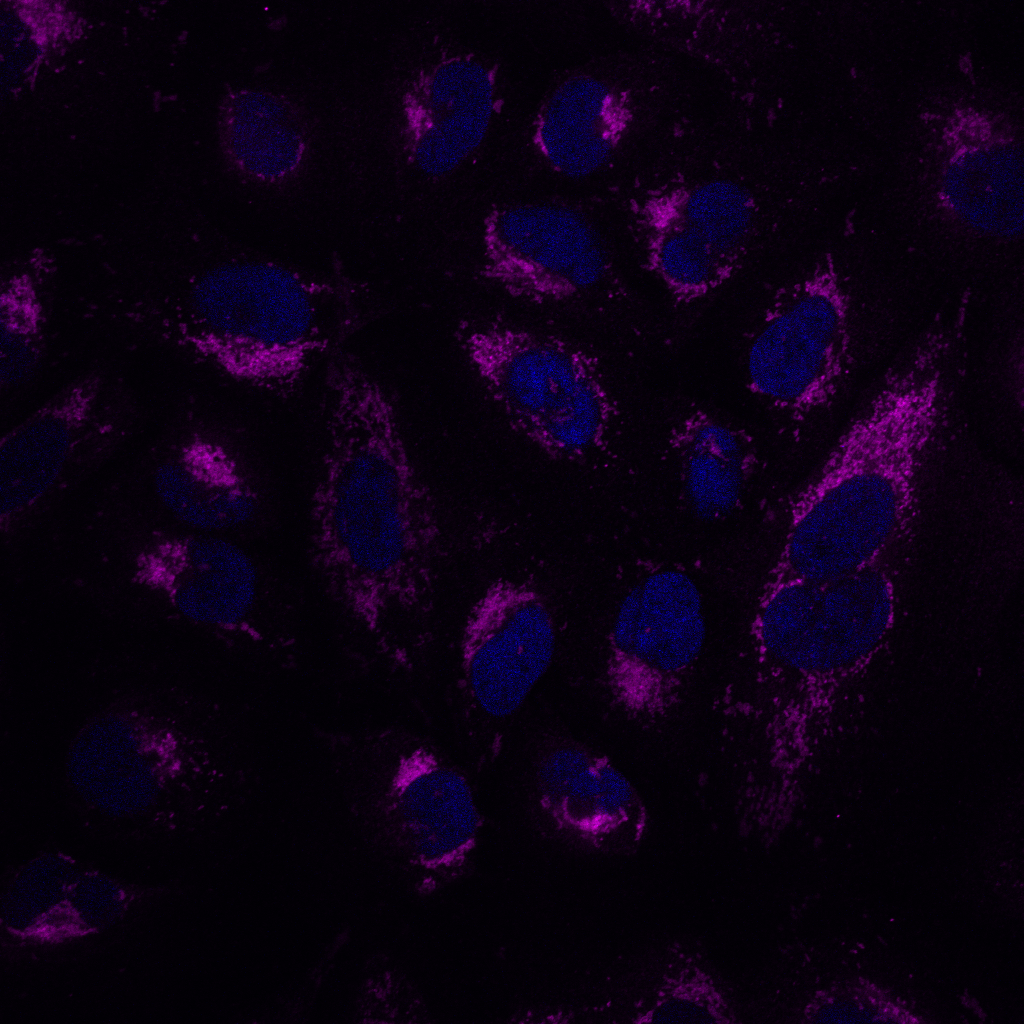

Supplement: Supplementary file 7 — Source Data Fig. 4 [file 44318_2024_44_MOESM7_ESM.zip › Fig 4/Fig 4F/Fig4F_DAPI_TOM20_uncropped_U2OS GFP-NEMO TOM20 3hr cicd (RGB).tif]

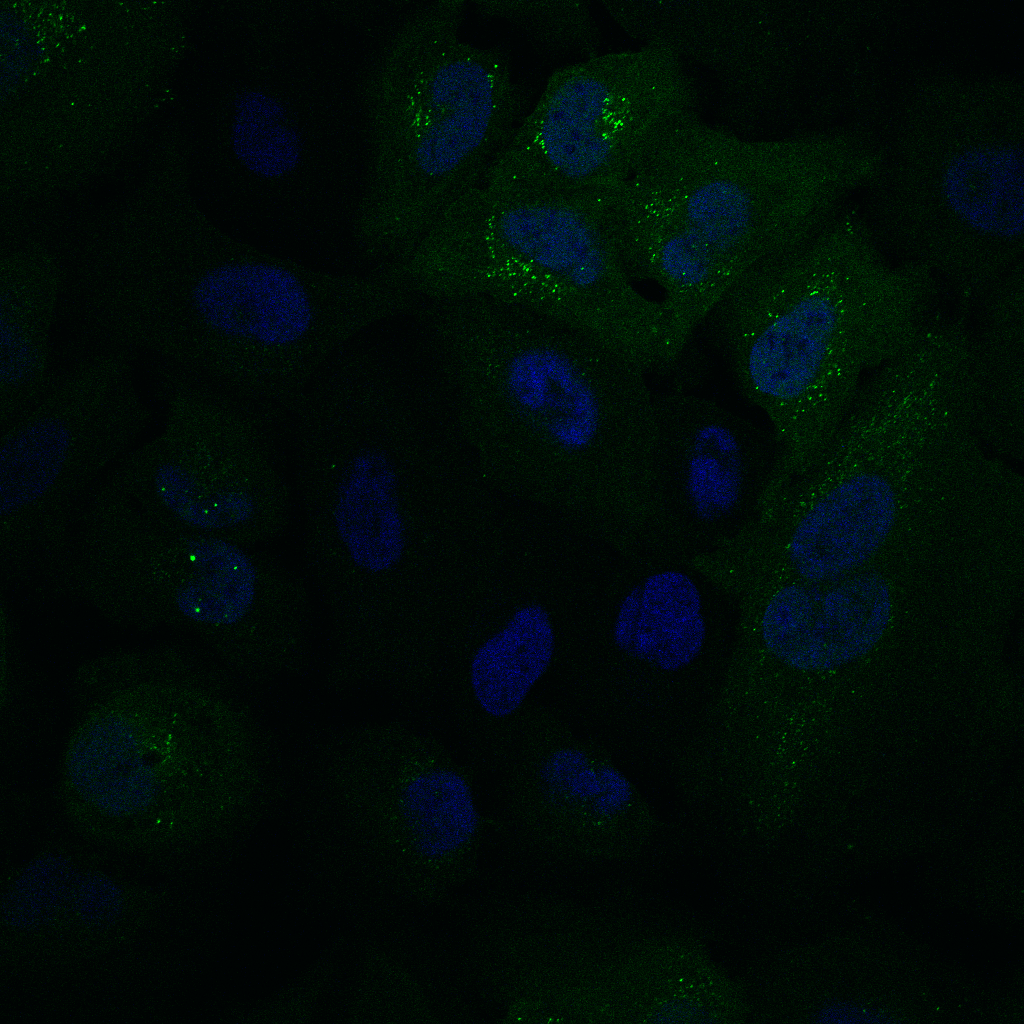

Supplement: Supplementary file 7 — Source Data Fig. 4 [file 44318_2024_44_MOESM7_ESM.zip › Fig 4/Fig 4F/Fig4F_DAPI_GFP_uncropped_U2OS GFP-NEMO TOM20 3hr cicd (RGB).tif]

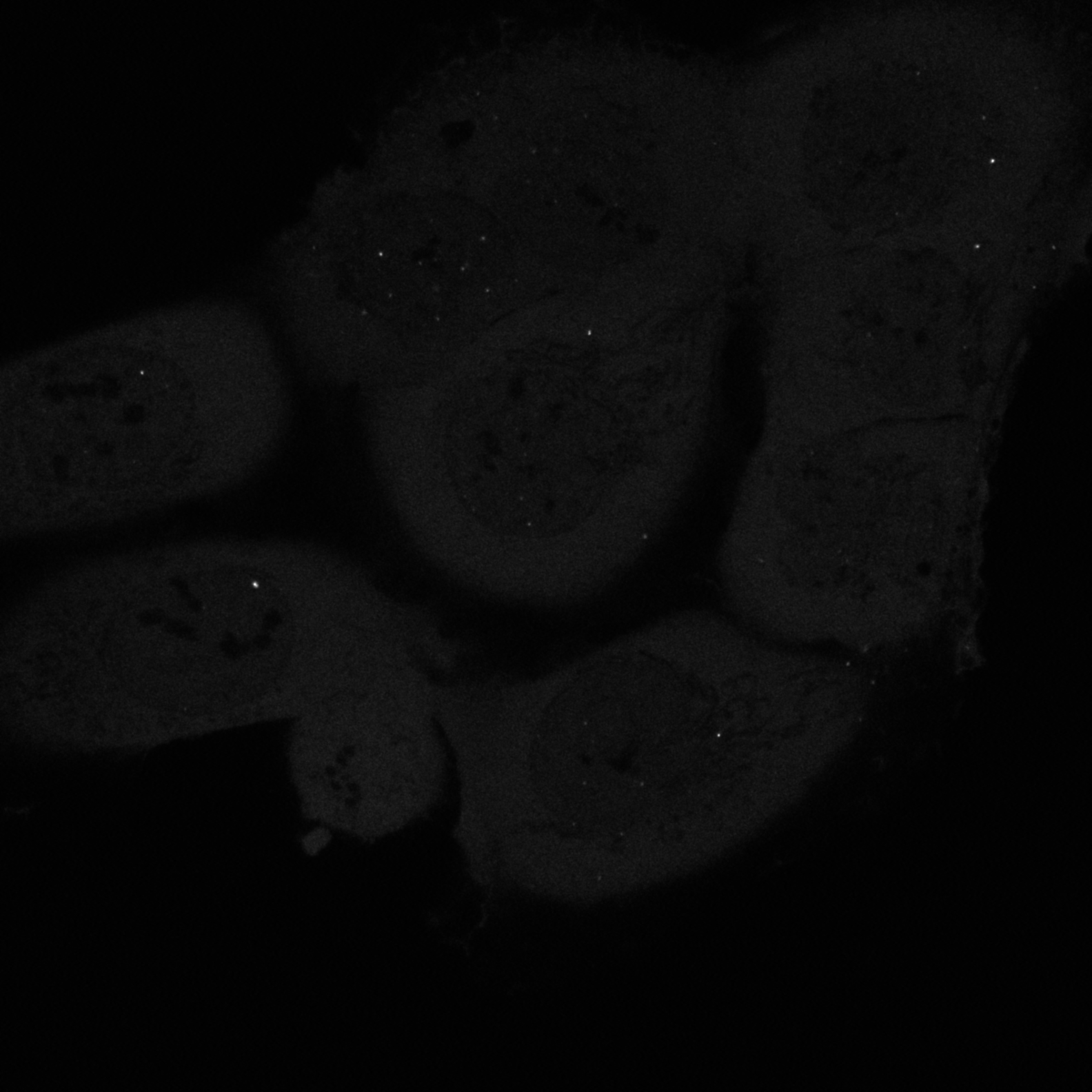

Supplement: Supplementary file 7 — Source Data Fig. 4 [file 44318_2024_44_MOESM7_ESM.zip › Fig 4/Fig 4A/Fig_4A-U2OS-DMSO_GFP-NEMO.tif]

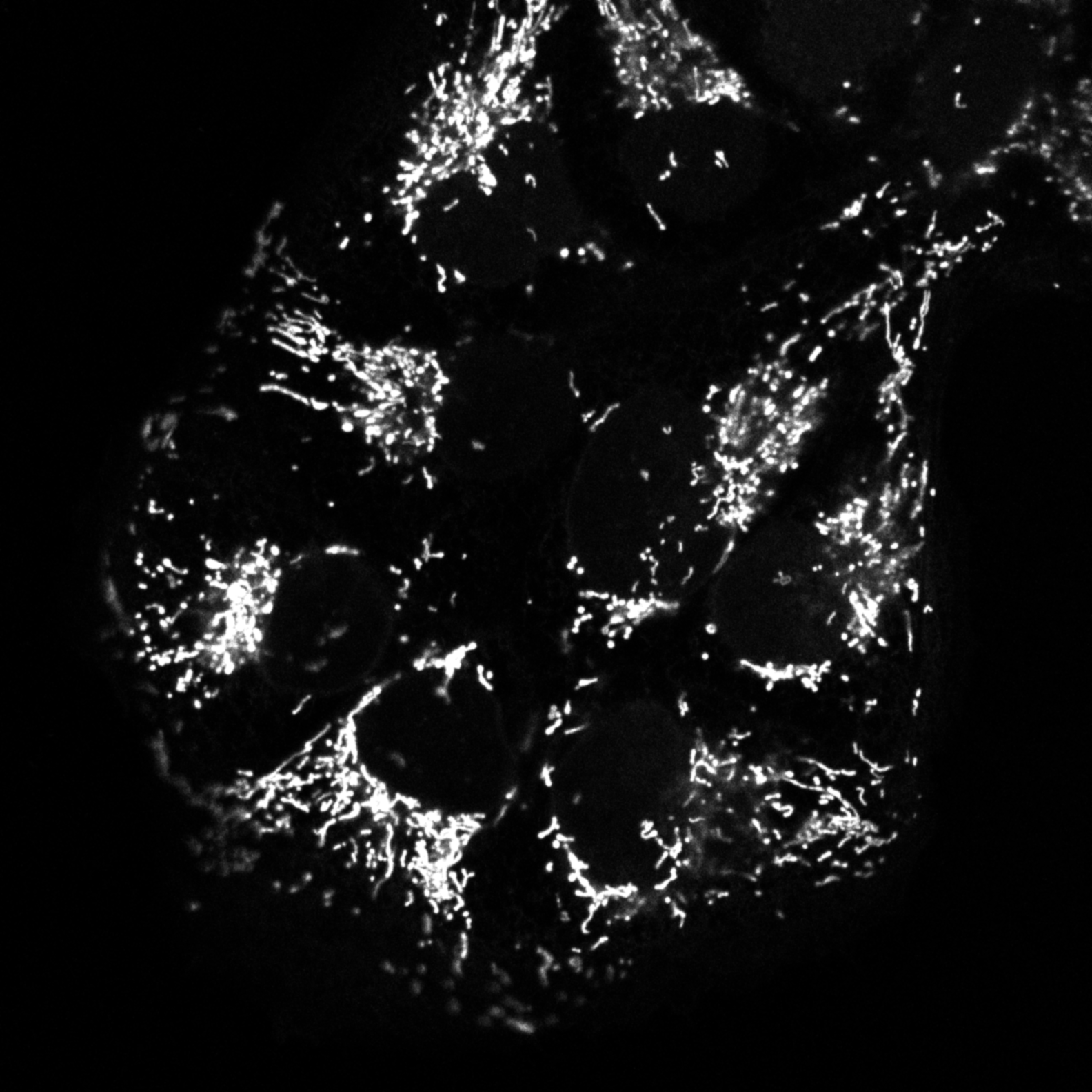

Supplement: Supplementary file 7 — Source Data Fig. 4 [file 44318_2024_44_MOESM7_ESM.zip › Fig 4/Fig 4A/Fig_4A-U2OS-CICD_PkMito.tif]

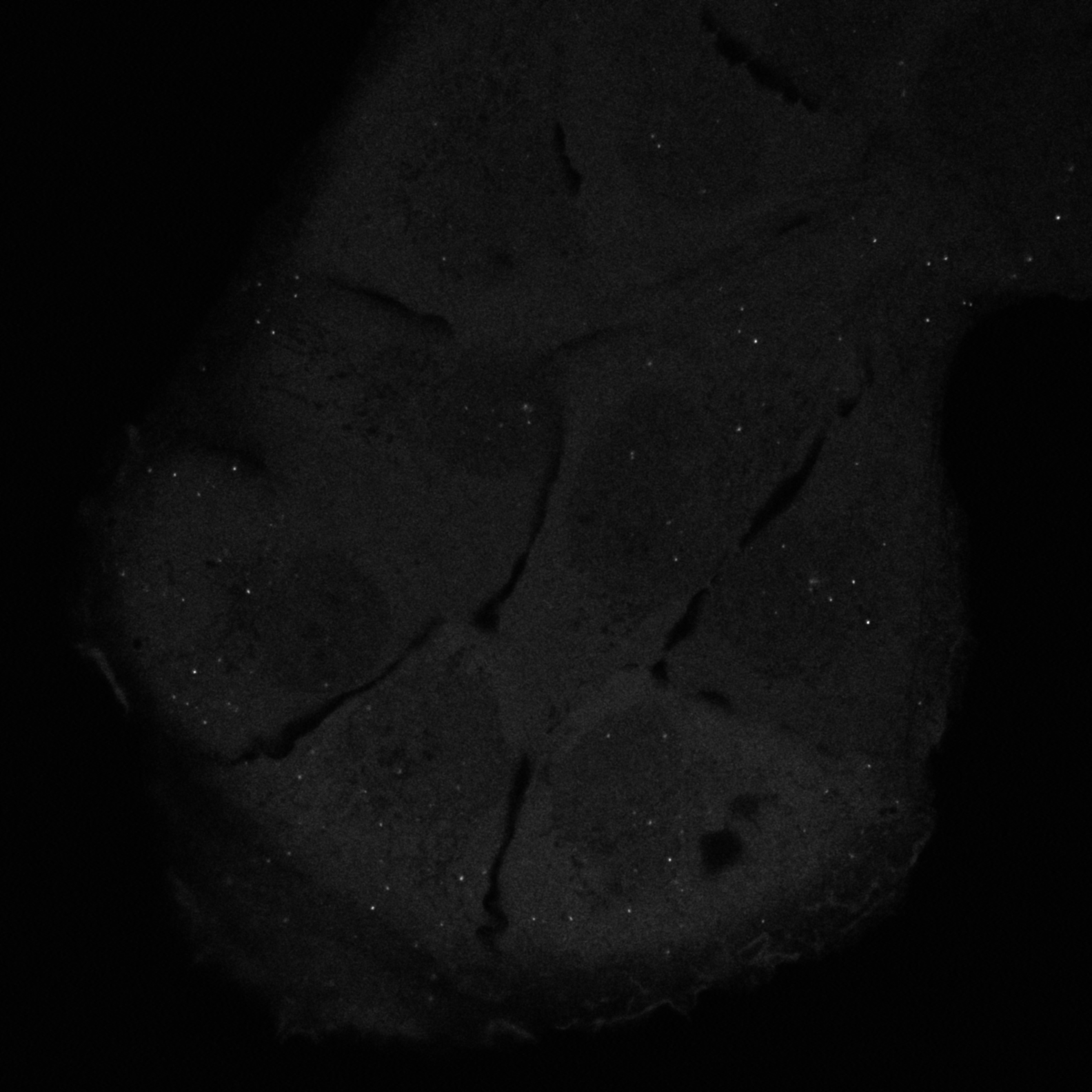

Supplement: Supplementary file 7 — Source Data Fig. 4 [file 44318_2024_44_MOESM7_ESM.zip › Fig 4/Fig 4A/Fig_4A-U2OS-CICD_GFP-NEMO.tif]

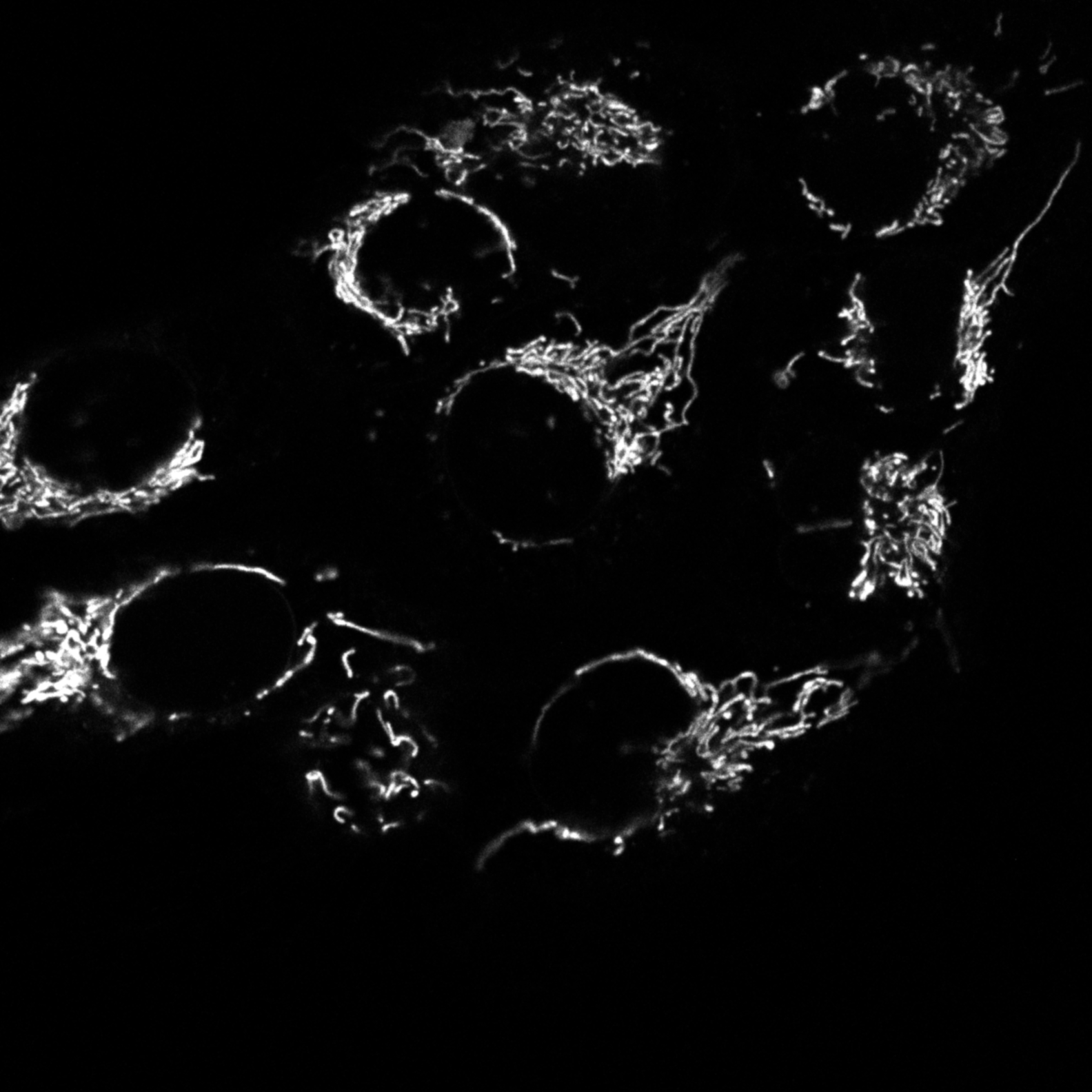

Supplement: Supplementary file 7 — Source Data Fig. 4 [file 44318_2024_44_MOESM7_ESM.zip › Fig 4/Fig 4A/Fig_4A-U2OS-DMSO_PkMito.tif]

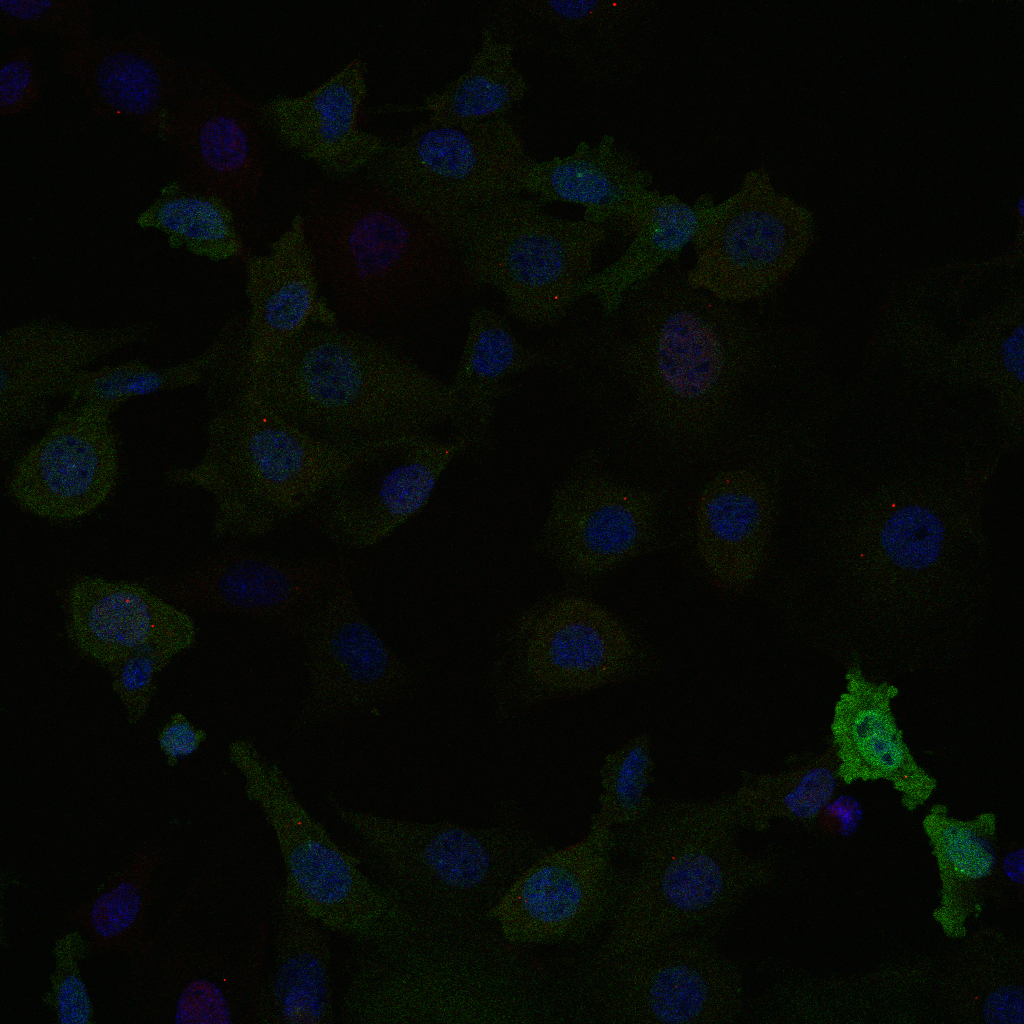

Supplement: Supplementary file 7 — Source Data Fig. 4 [file 44318_2024_44_MOESM7_ESM.zip › Fig 4/Fig 4H/Fig4H_SVEC GFP-NEMOD311N_DAPI_p65_cicd_merge(RGB).tif]

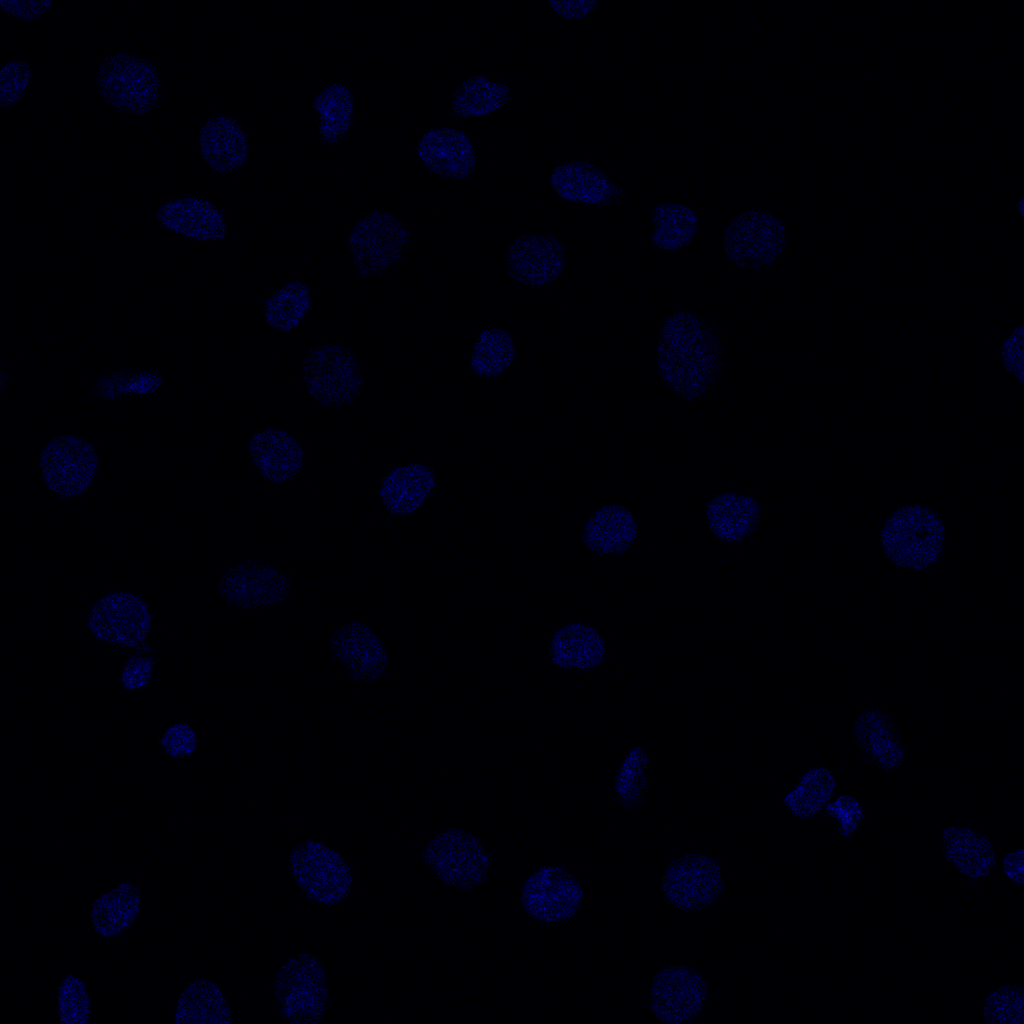

Supplement: Supplementary file 7 — Source Data Fig. 4 [file 44318_2024_44_MOESM7_ESM.zip › Fig 4/Fig 4H/Fig4H_SVEC GFP-NEMOD311N_DAPI_p65_cicd_DAPI (RGB).tif]

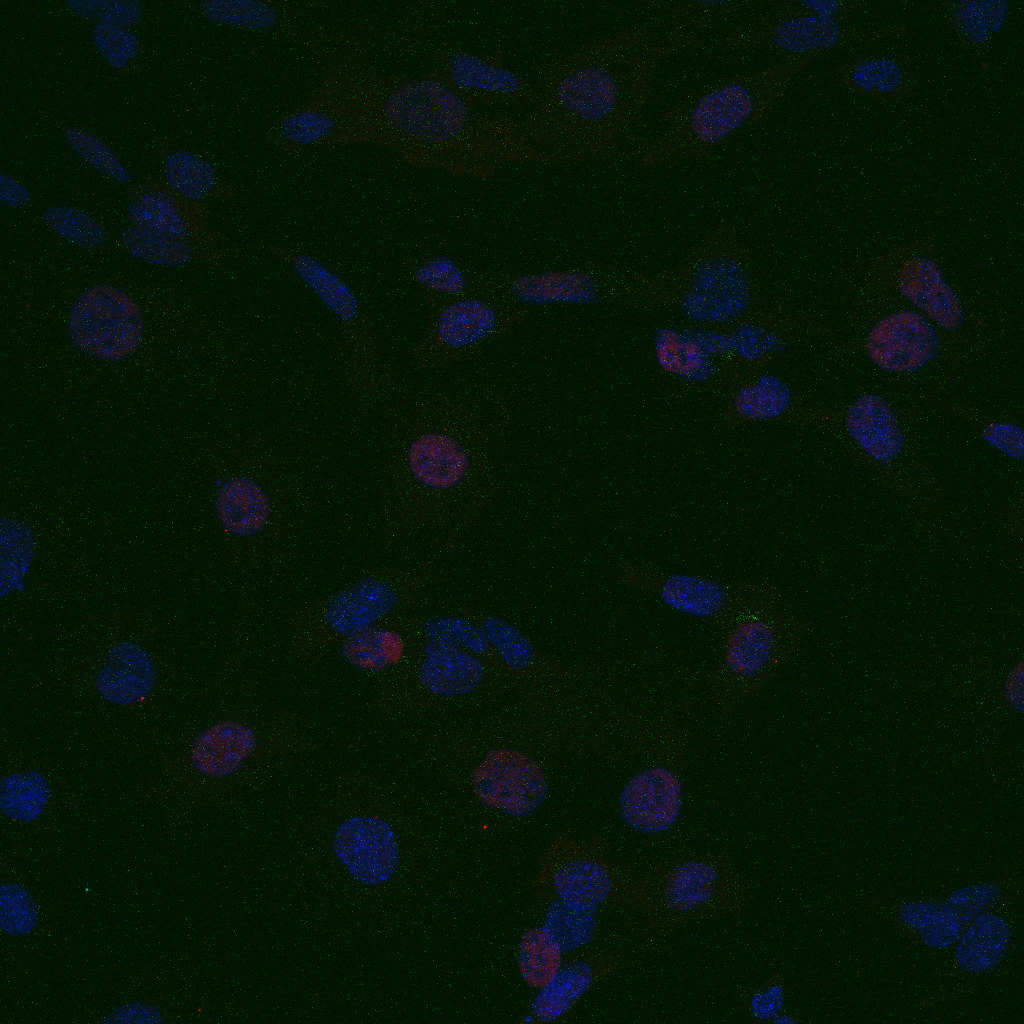

Supplement: Supplementary file 7 — Source Data Fig. 4 [file 44318_2024_44_MOESM7_ESM.zip › Fig 4/Fig 4H/Fig4H_SVEC_DAPI_p65_cicd_merge (RGB).tif]

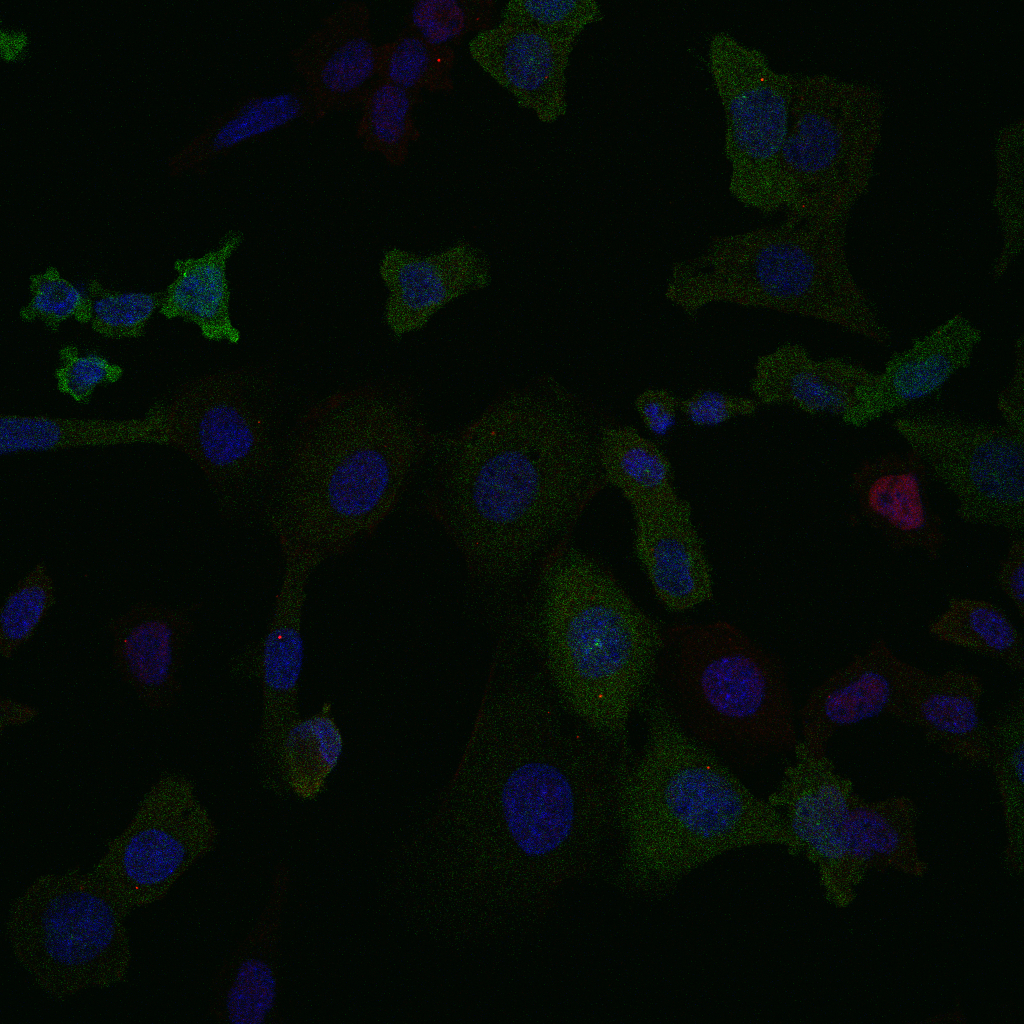

Supplement: Supplementary file 7 — Source Data Fig. 4 [file 44318_2024_44_MOESM7_ESM.zip › Fig 4/Fig 4H/Fig4H_SVEC GFP-NEMOZF_DAPI_p65_cicd_merge(RGB).tif]

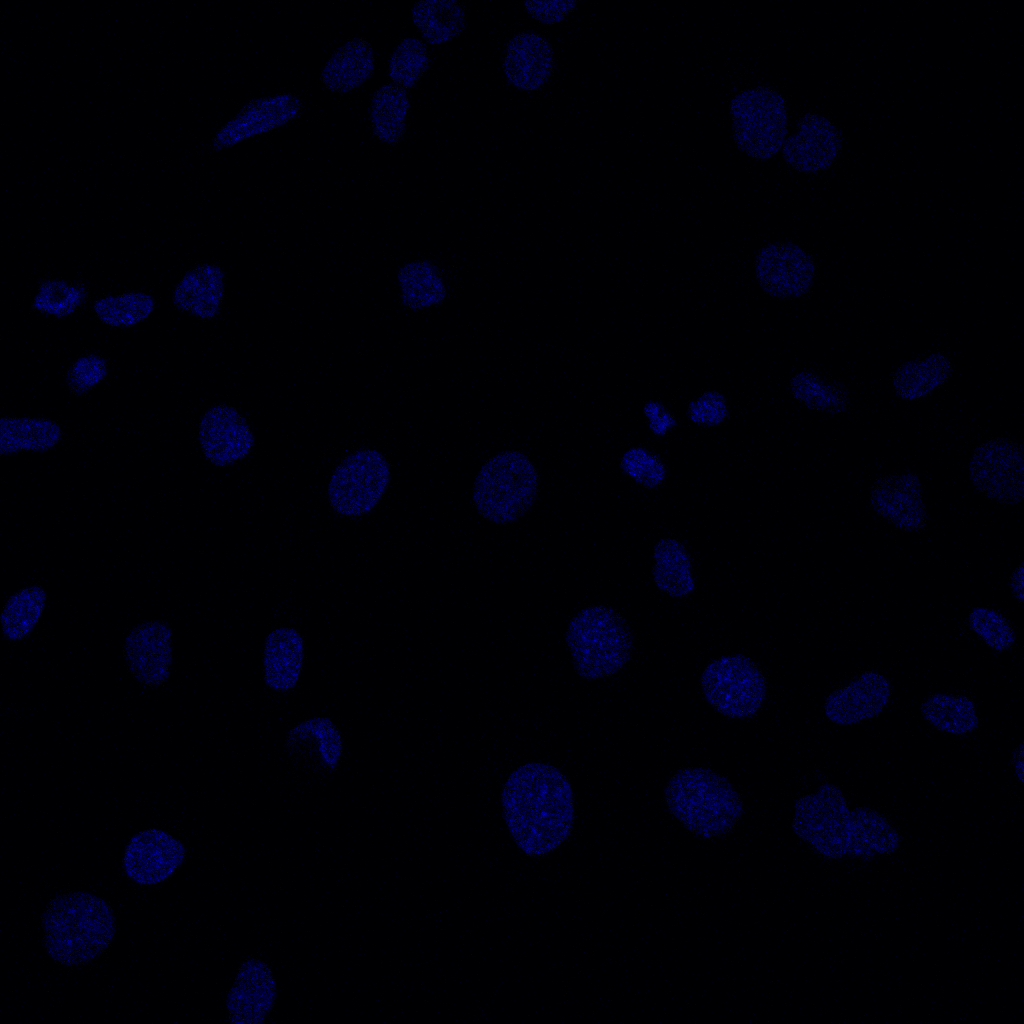

Supplement: Supplementary file 7 — Source Data Fig. 4 [file 44318_2024_44_MOESM7_ESM.zip › Fig 4/Fig 4H/Fig4H_SVEC GFP-NEMOZF_DAPI_p65_cicd_DAPI (RGB).tif]

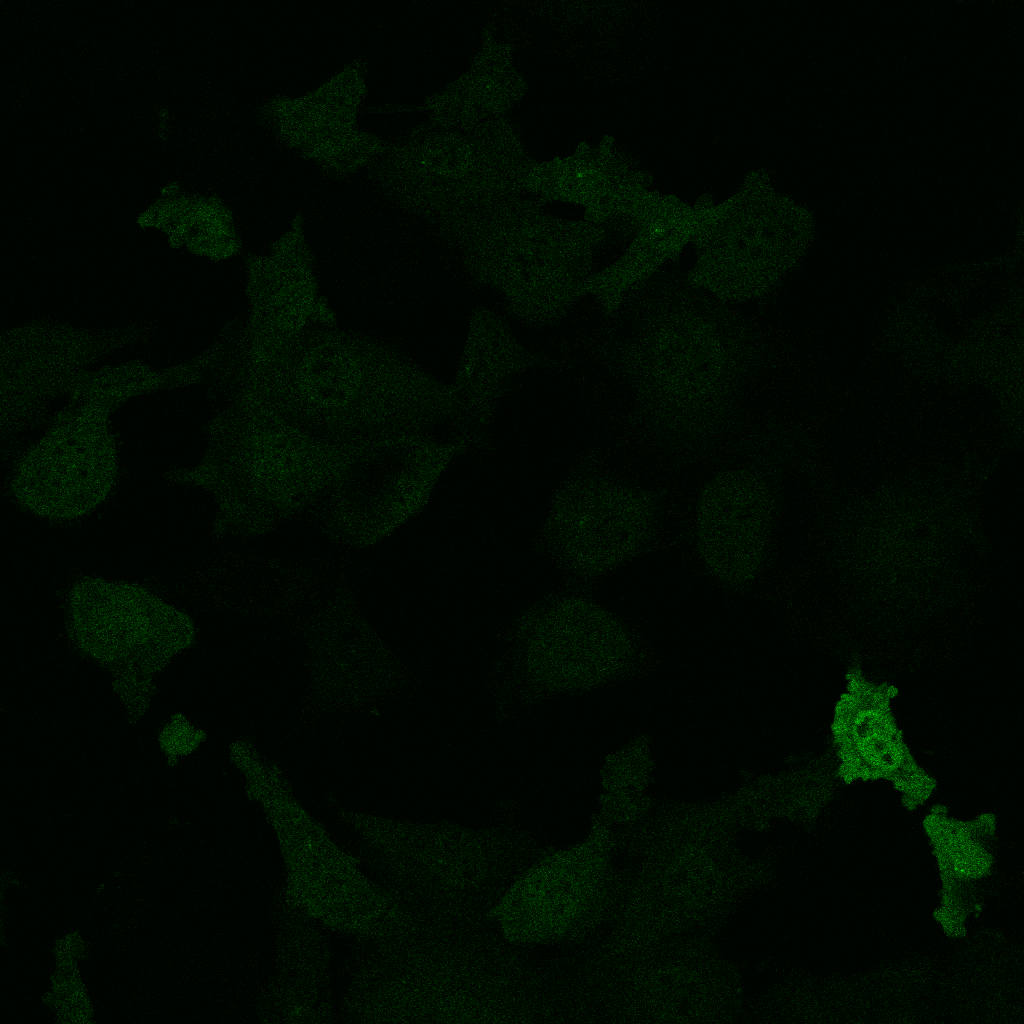

Supplement: Supplementary file 7 — Source Data Fig. 4 [file 44318_2024_44_MOESM7_ESM.zip › Fig 4/Fig 4H/Fig4H_SVEC GFP-NEMOD311N_DAPI_p65_cicd_GFP (RGB).tif]

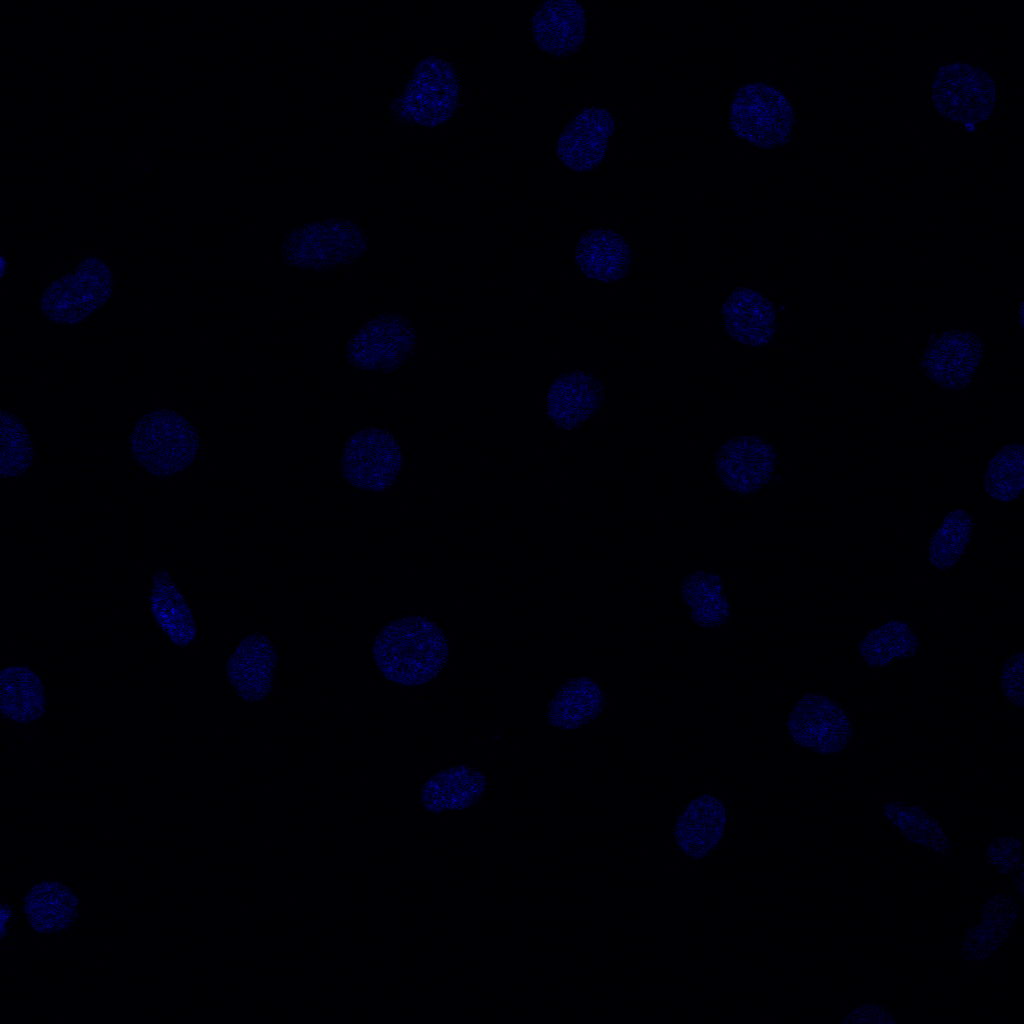

Supplement: Supplementary file 7 — Source Data Fig. 4 [file 44318_2024_44_MOESM7_ESM.zip › Fig 4/Fig 4H/Fig4H_SVEC_GFP-NEMO_DAPI_p65_cicd_DAPI (RGB).tif]

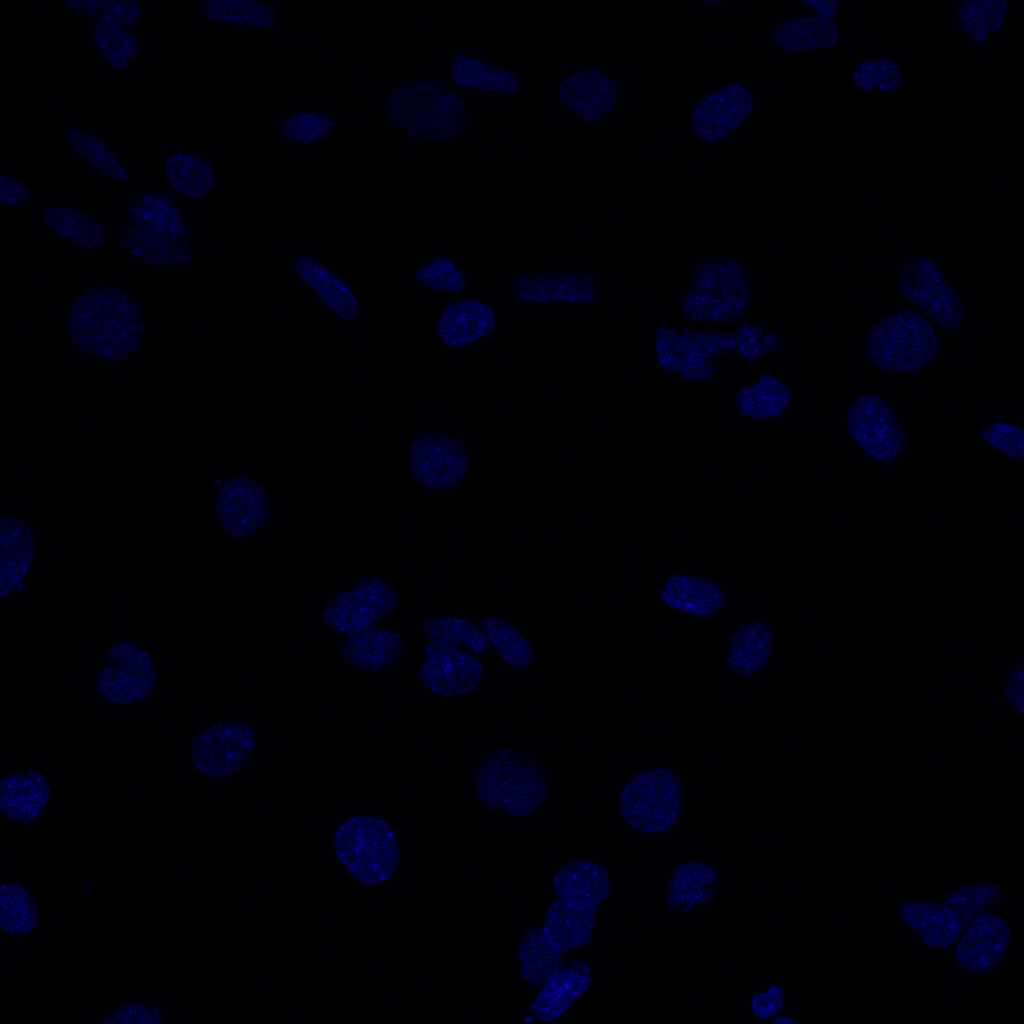

Supplement: Supplementary file 7 — Source Data Fig. 4 [file 44318_2024_44_MOESM7_ESM.zip › Fig 4/Fig 4H/Fig4H_SVEC_DAPI_p65_cicd_DAPI (RGB).tif]

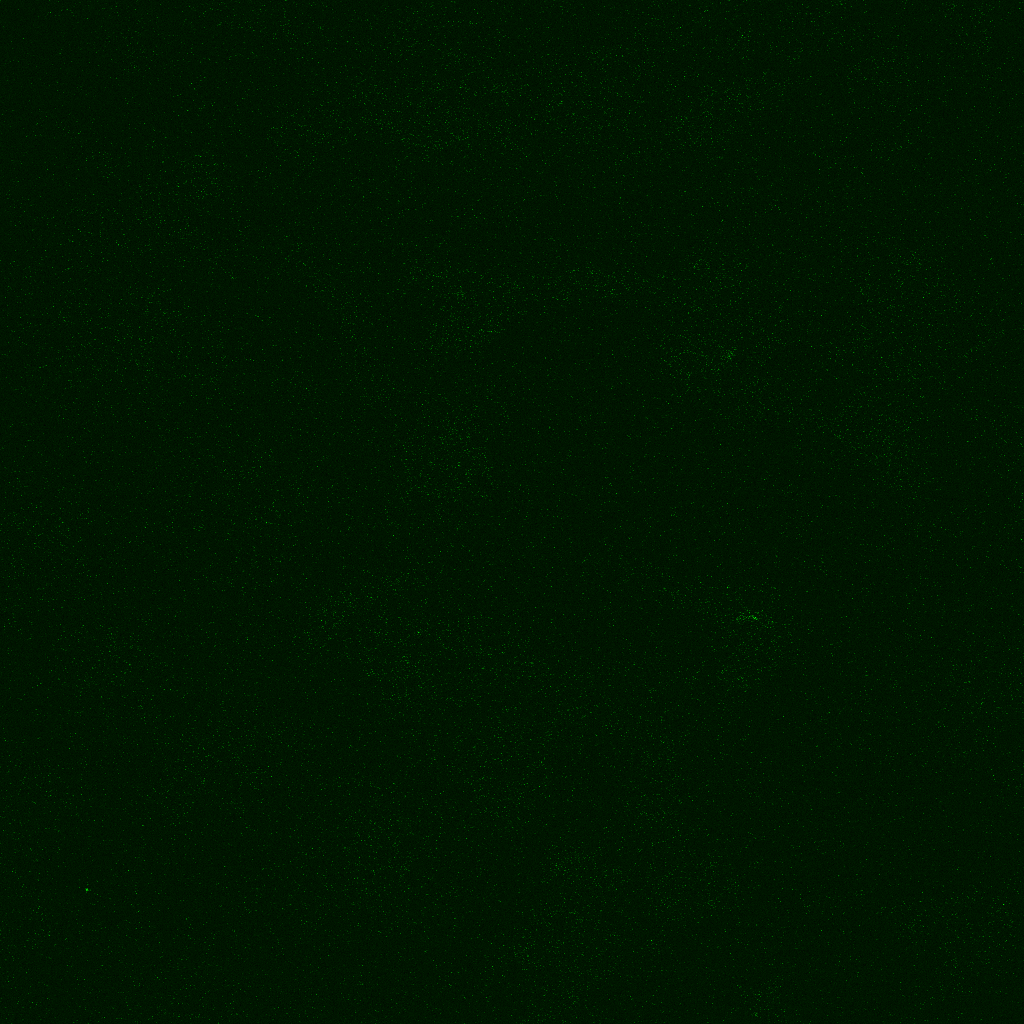

Supplement: Supplementary file 7 — Source Data Fig. 4 [file 44318_2024_44_MOESM7_ESM.zip › Fig 4/Fig 4H/Fig4H_SVEC_DAPI_p65_cicd_GFP (RGB).tif]

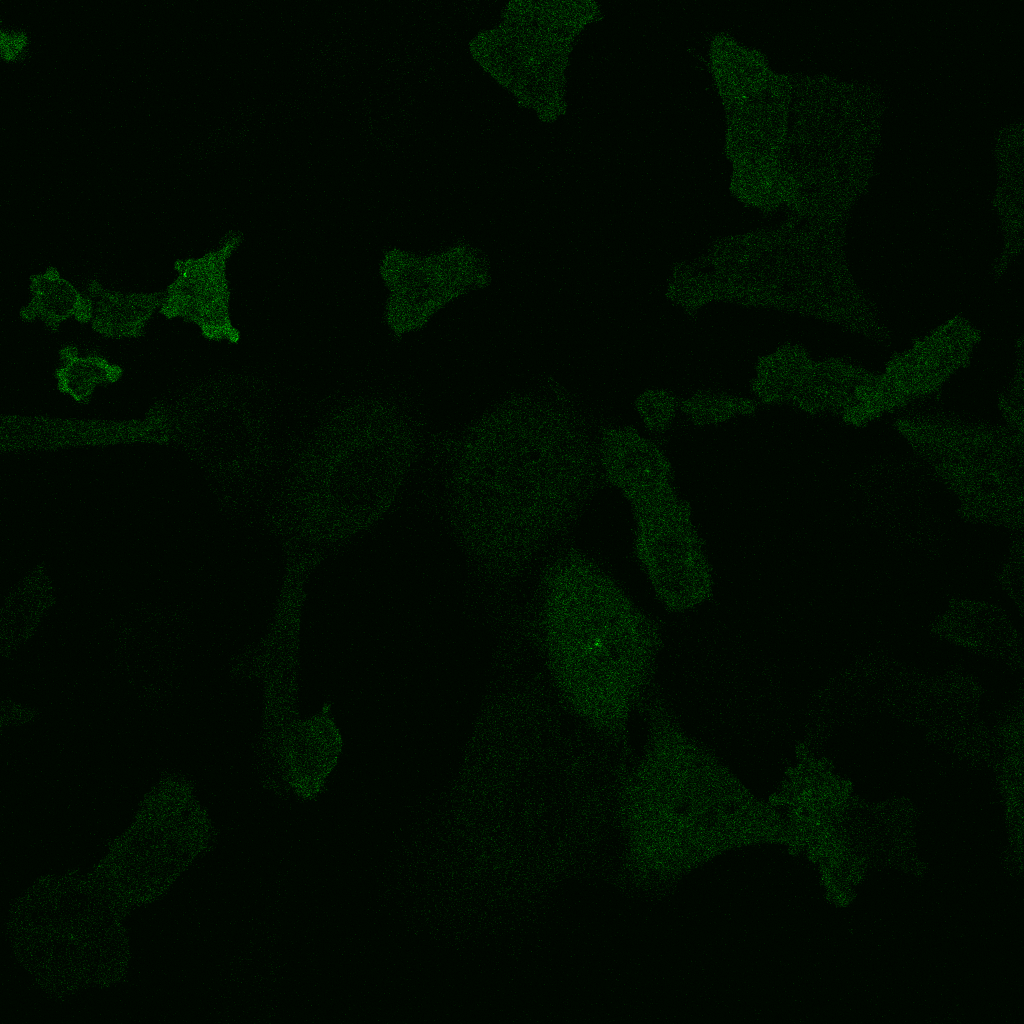

Supplement: Supplementary file 7 — Source Data Fig. 4 [file 44318_2024_44_MOESM7_ESM.zip › Fig 4/Fig 4H/Fig4H_SVEC GFP-NEMOZF_DAPI_p65_cicd_GFP (RGB).tif]

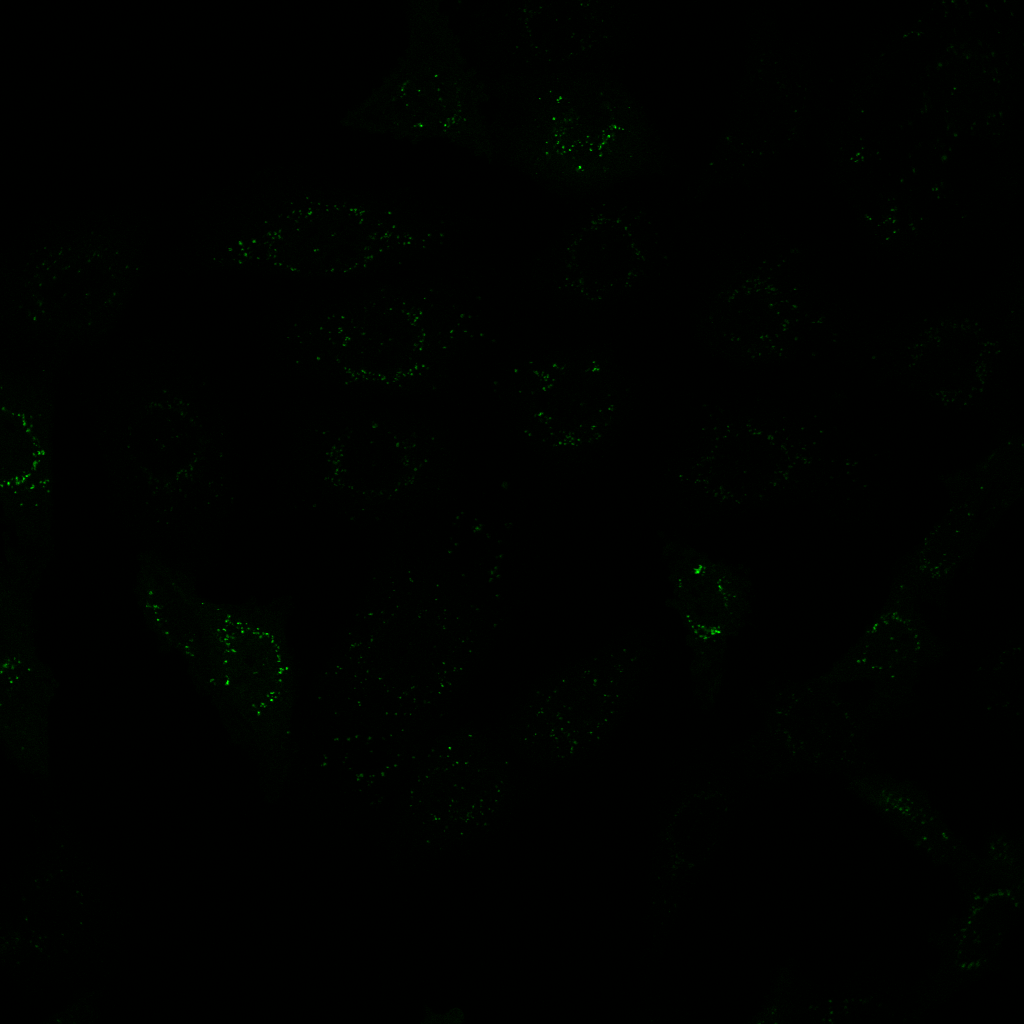

Supplement: Supplementary file 7 — Source Data Fig. 4 [file 44318_2024_44_MOESM7_ESM.zip › Fig 4/Fig 4H/Fig4H_SVEC_GFP-NEMO_DAPI_p65_cicd_GFP (RGB).tif]

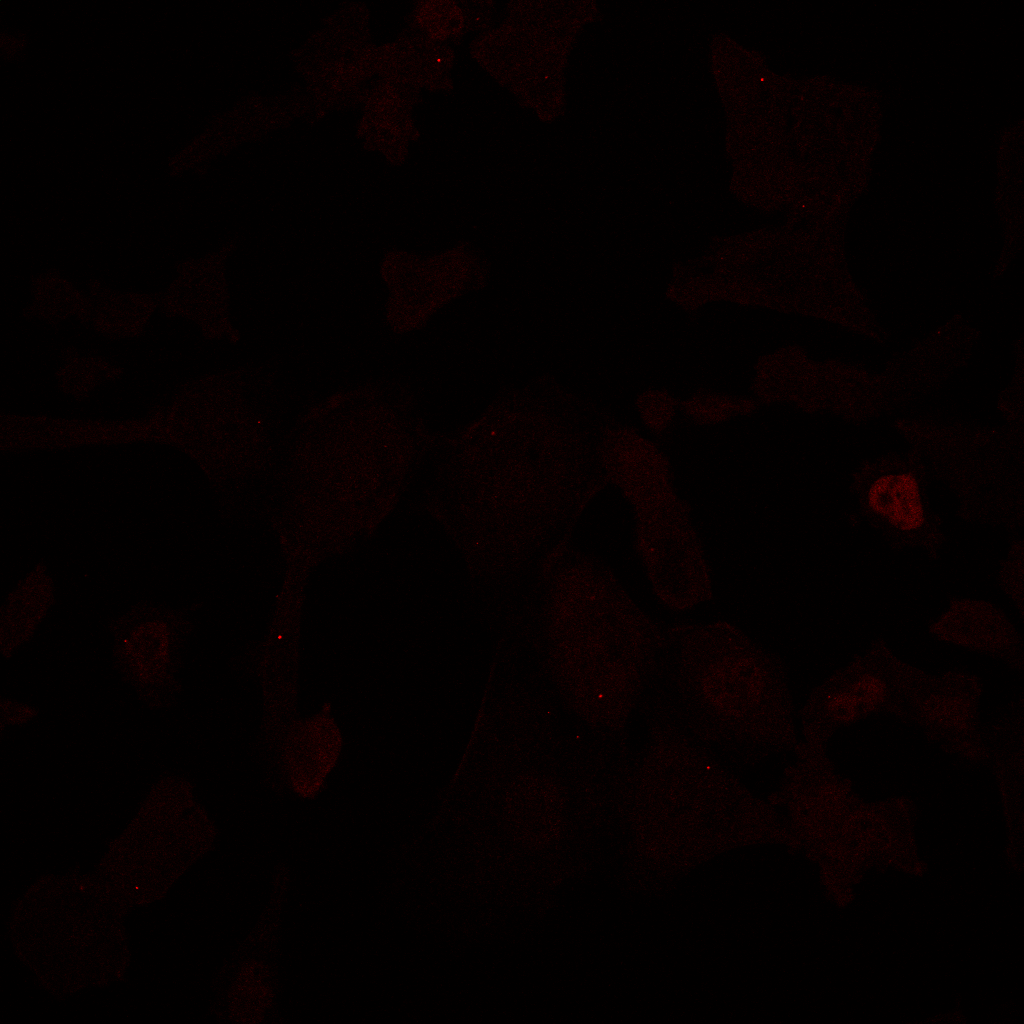

Supplement: Supplementary file 7 — Source Data Fig. 4 [file 44318_2024_44_MOESM7_ESM.zip › Fig 4/Fig 4H/Fig4H_SVEC GFP-NEMOZF_DAPI_p65_cicd_p65 (RGB).tif]

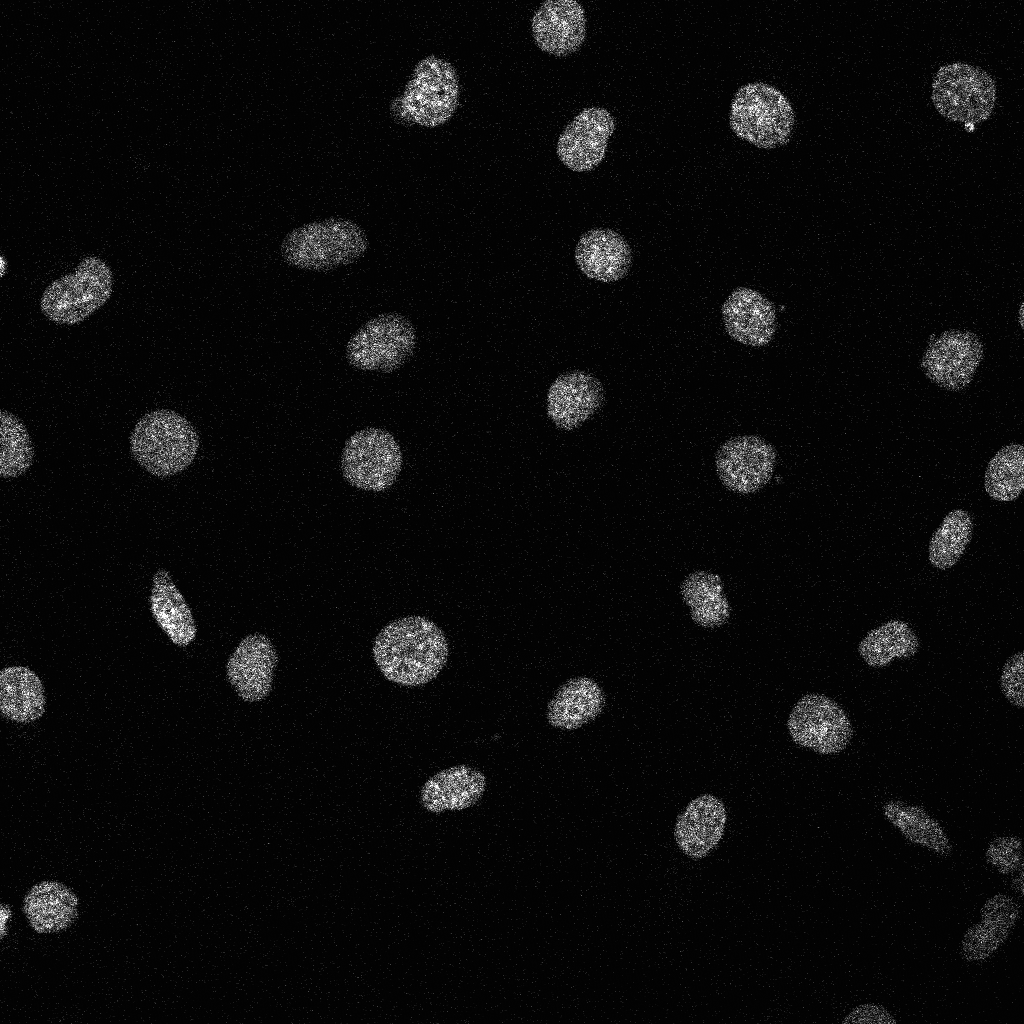

Supplement: Supplementary file 7 — Source Data Fig. 4 [file 44318_2024_44_MOESM7_ESM.zip › Fig 4/Fig 4H/Fig4H_SVEC_GFP-NEMO_DAPI_p65_cicd_p65 (RGB).tif]

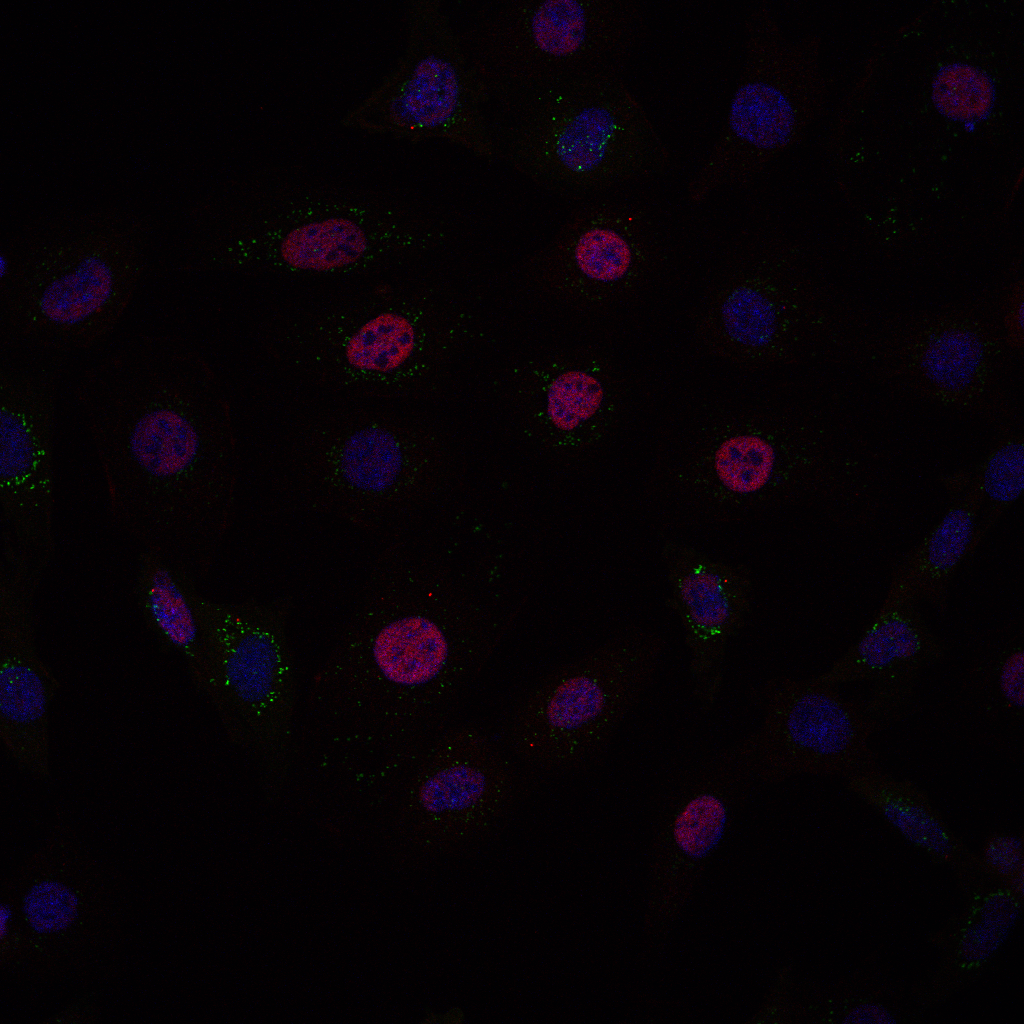

Supplement: Supplementary file 7 — Source Data Fig. 4 [file 44318_2024_44_MOESM7_ESM.zip › Fig 4/Fig 4H/Fig4H_SVEC_GFP-NEMO_DAPI_p65_cicd_merge (RGB).tif]

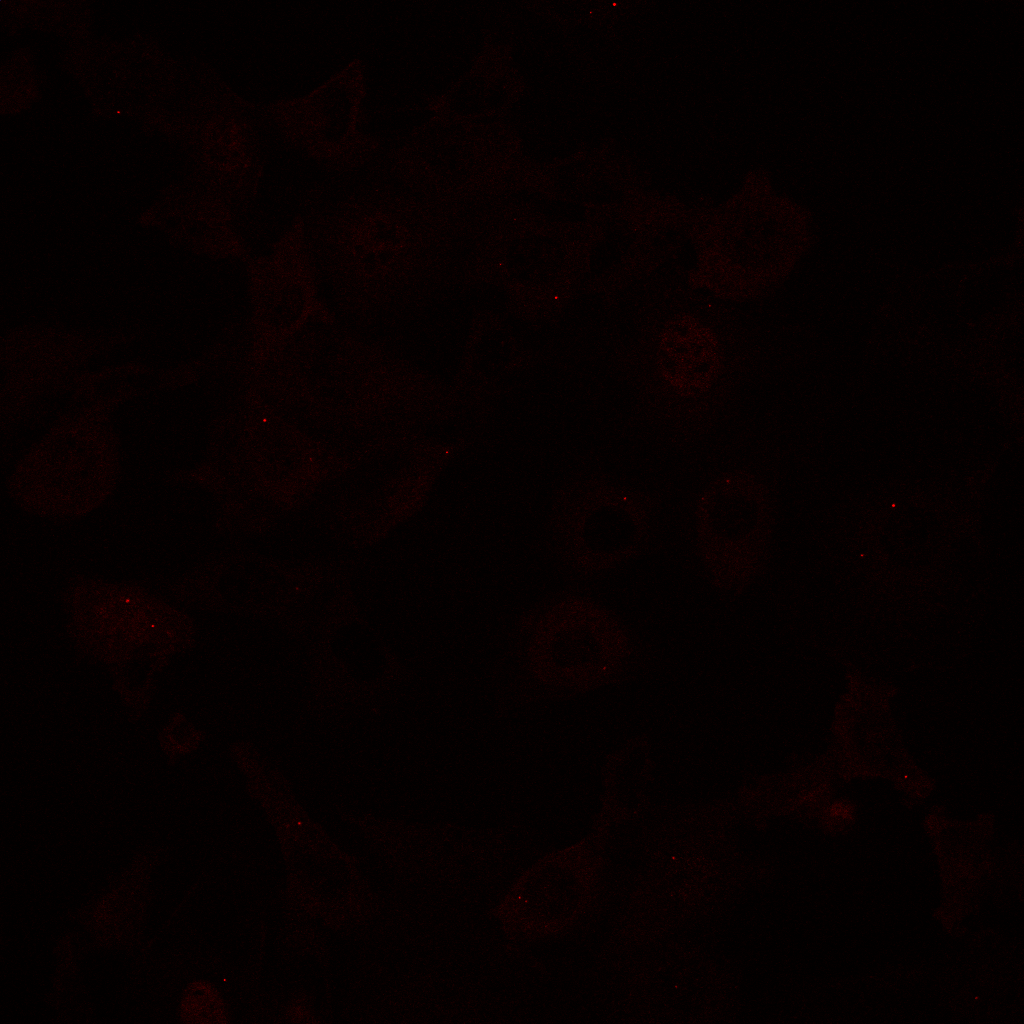

Supplement: Supplementary file 7 — Source Data Fig. 4 [file 44318_2024_44_MOESM7_ESM.zip › Fig 4/Fig 4H/Fig4H_SVEC GFP-NEMOD311N_DAPI_p65_cicd_p65 (RGB).tif]

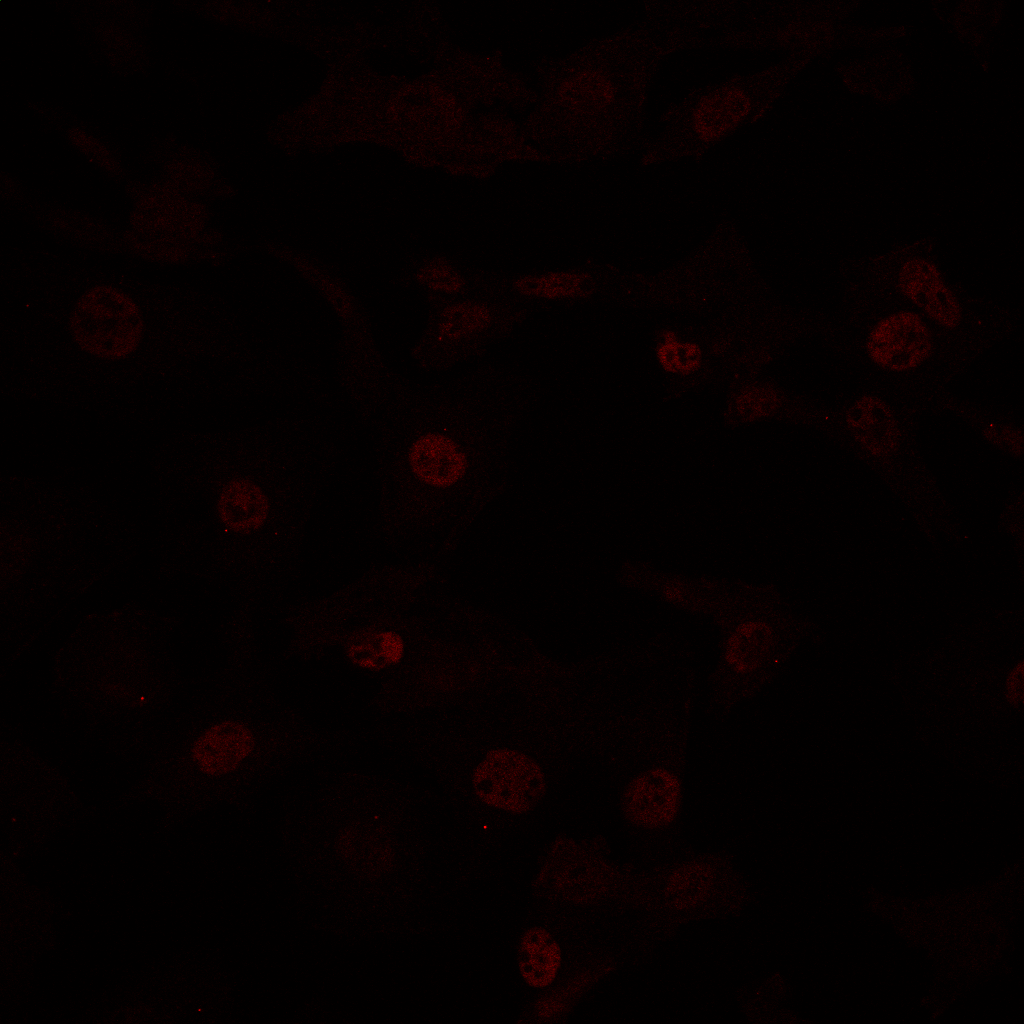

Supplement: Supplementary file 7 — Source Data Fig. 4 [file 44318_2024_44_MOESM7_ESM.zip › Fig 4/Fig 4H/Fig4H_SVEC_DAPI_p65_cicd_p65 (RGB).tif]
