# Supplementary material for: Mitochondrial outer membrane integrity regulates a ubiquitin-dependent and NF-κB-mediated inflammatory response
Source: EMBO J. 2024 Feb 9;43(6):904–30. doi: 10.1038/s44318-024-00044-1 (PMC10943237; doi:10.1038/s44318-024-00044-1)
Supplement: Supplementary file 7 — Source Data Fig. 4 [file 44318_2024_44_MOESM7_ESM.zip › Fig 4/Fig 4A/batch_macro_fijiimagej.docx]

// // Authors: Rosalie Heilig, Claire Mitchell, and Ryan Corbyn

// // Date: 15/08/2023 mod.: 04/10/2023

// Select the directory containing the images to analyse.

dir = getDirectory("Select folder of images");

// Get all of the File names from the folder.

list = getFileList(dir);

// Select the two Weka segmentation models for

// Mitochondria

model_mito = File.openDialog("Select classifer mitochondria model file");

// Set-up the measurements needed for the analysis.

run("Set Measurements...", "integrated area_fraction redirect=None decimal=3");

// Create new directories for the results of the analysis

// and the detected spot locations.

newdir = File.getParent(dir) + "/results/spot_location/";

savedir = File.getParent(dir) + "/results/";

File.makeDirectory(savedir);

File.makeDirectory(newdir);

// Loop around all images in the folder.

for (i=0; i<lengthOf(list); i++) {

// Generate a file to contain the analysis.

f = File.open(savedir + substring(list[i], 0 , lengthOf(list[i])-4) + "_analysis.csv");

// Add the headers to the file.

print(f, "Area (um^2)" + " ," + "Count" + "\n");

// Use the bioformats to open up the .czi image file.

run("Bio-Formats Importer", "open=[" + dir + list[i] + "] autoscale color_mode=Default rois_import=[ROI manager] view=Hyperstack stack_order=XYCZT");

// Split the image channels.

run("Split Channels");

// Close channel 3.

close("C3-" + list[i]);

// Select the first channel (mitochondria) image.

selectImage("C1-" + list[i]);

// Run the Weka segmentation for the first channel image.

run("Trainable Weka Segmentation");

// Wait is needed to avoid program crashing.

wait(3000);

// Run the segmentation and generate the classified image.

selectImage("Trainable Weka Segmentation v3.3.2");

call("trainableSegmentation.Weka_Segmentation.loadClassifier", model_mito);

call("trainableSegmentation.Weka_Segmentation.getResult");

// Select the binary image generated by the Weka segmentation.

selectImage("Classified image");

// To remove the background from the image all values are multiplied

// by 255 to allow for the inversion of the pixel values to work properly.

// Once inverted, the image values are divided by 255 to generate the binary mask.

run("Multiply...", "value=255 stack");

run("Invert", "stack");

run("Divide...", "value=255 stack");

// The image calculator allows to multiply to images resulting in only our mitohcondrial

// selection image 1 is the NEMO channel image 2 is the clasified mitochondria

imageCalculator("Multiply create stack", "Classified image","C2-" + list[i]);

selectImage("Result of Classified image");

// Create a new variable count_array.

count_array = newArray(nSlices);

// Loop around all of the frames in the image:

// "Results of Classified image"

for (j = 1; j <= nSlices; j++) {

// Pick the slice you wish to analyse.

setSlice(j);

// Find the bringht spots in the image.

run("Find Maxima...", "prominence=40 output=List");

// Save the bright-spot locations to a .csv file.

saveAs("Results", newdir + substring(list[i], 0 , lengthOf(list[i])-4) + j + "_output_list.csv");

// Clear the results.

run("Clear Results");

// Find out how many bright spots were found above.

run("Find Maxima...", "prominence=40 output=Count");

// Add the total number of spots to the count_array variable.

count_array[j] = getResult("Count", 0);

// Clear the results variable.

run("Clear Results");

}

// Save the results of the Cell Area calulation and the

// count for the number of spots observed to a .csv file.

for (k = 1; k <= nSlices; k++) {

print(f, d2s(count_array[k],3) + "\n");

}

// Close the file containing the results of analysis.

File.close(f);

// Close every open window.

run("Close All");

}
