# Supplementary figures and images for: Mitochondrial outer membrane integrity regulates a ubiquitin-dependent and NF-κB-mediated inflammatory response (part 2 of 2)
Source: EMBO J. 2024 Feb 9;43(6):904–30. doi: 10.1038/s44318-024-00044-1 (PMC10943237; doi:10.1038/s44318-024-00044-1)

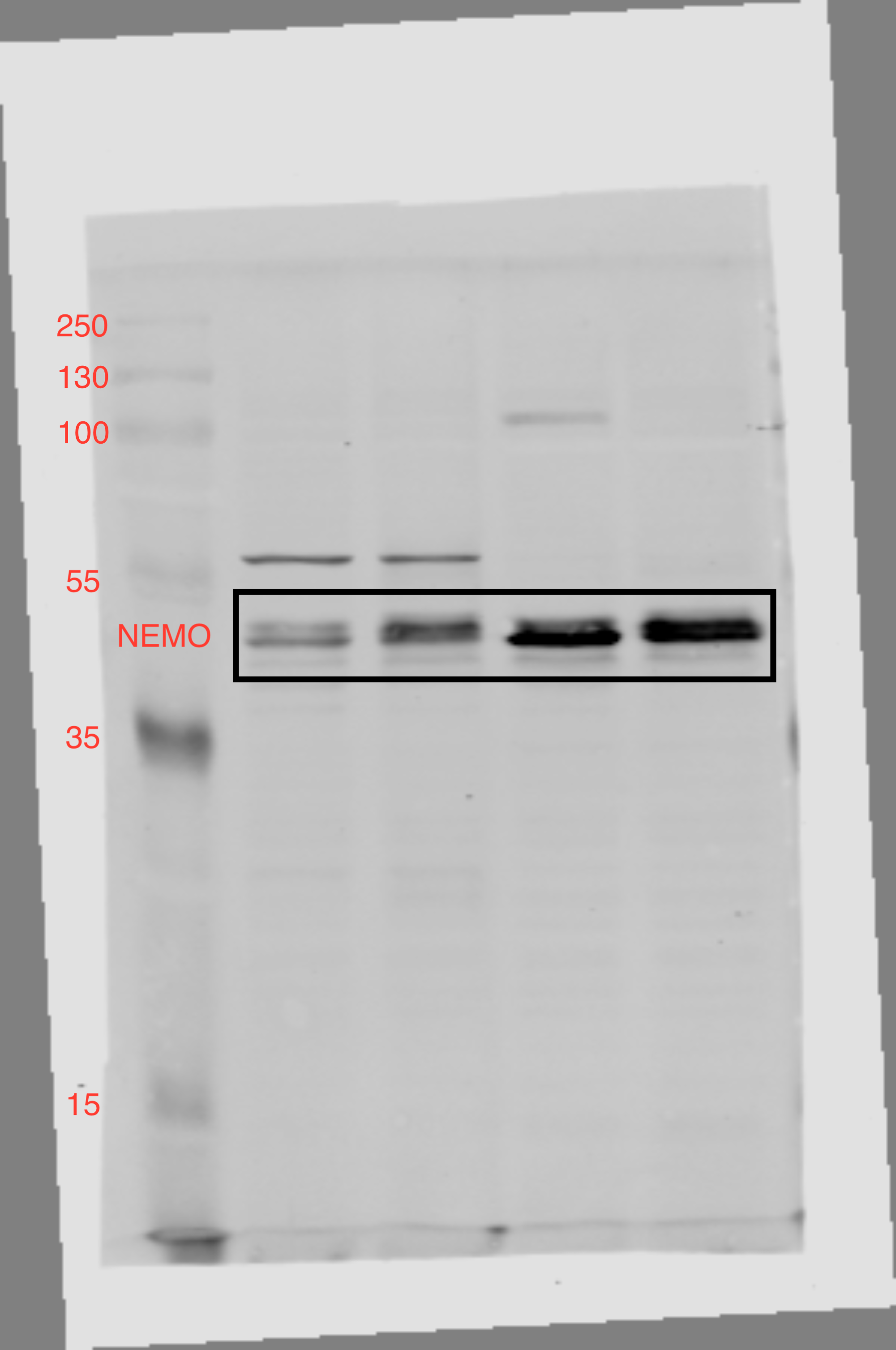

Supplement: Supplementary file 7 — Source Data Fig. 4 [file 44318_2024_44_MOESM7_ESM.zip › Fig 4/Fig 4C/Fig4C_NEMO.tif]

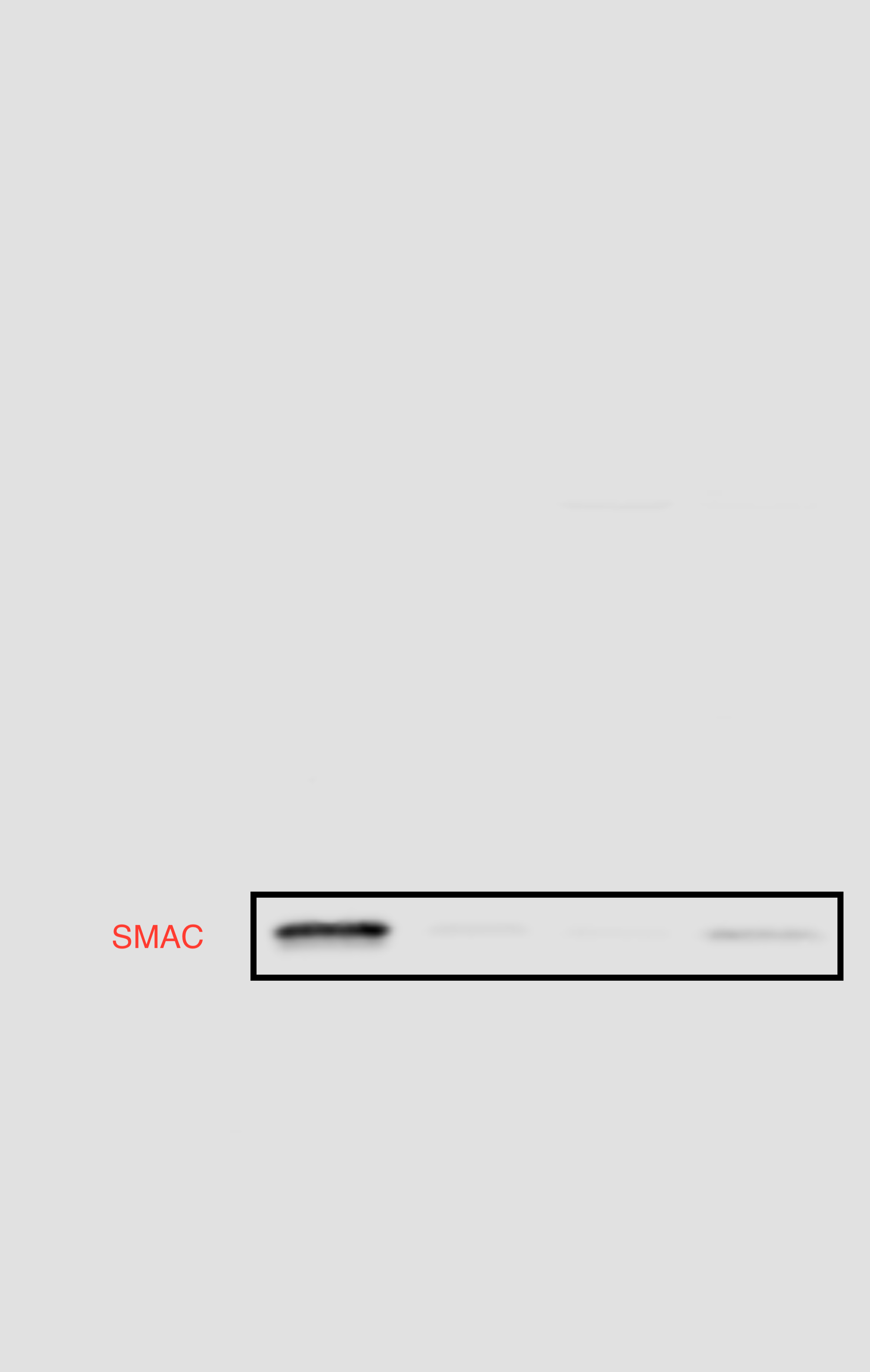

Supplement: Supplementary file 7 — Source Data Fig. 4 [file 44318_2024_44_MOESM7_ESM.zip › Fig 4/Fig 4C/Fig4C_NEMO_SMAC.tif]

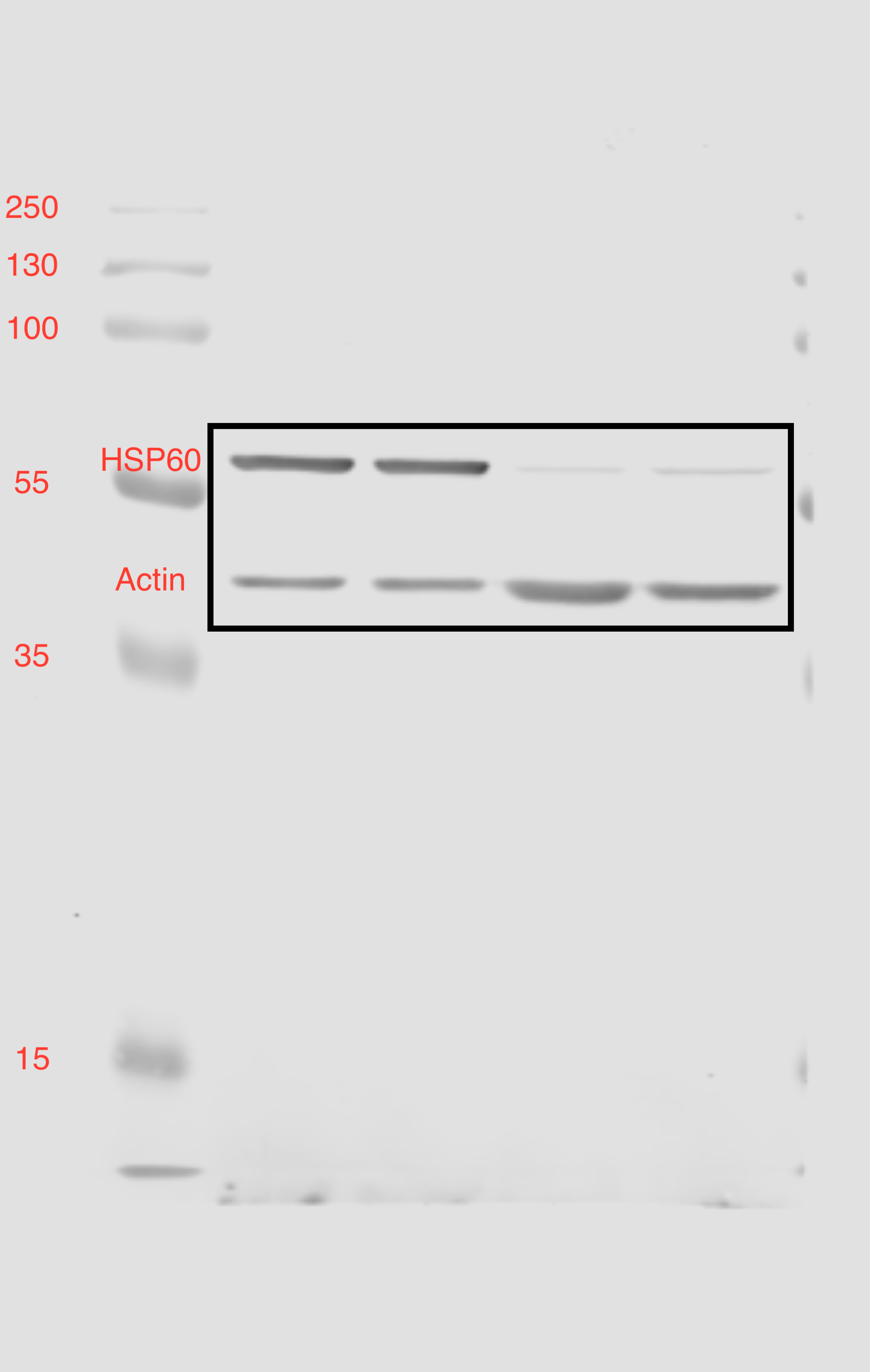

Supplement: Supplementary file 7 — Source Data Fig. 4 [file 44318_2024_44_MOESM7_ESM.zip › Fig 4/Fig 4C/Fig4C_NEMO_SMAC_HSP60_Actin.tif]

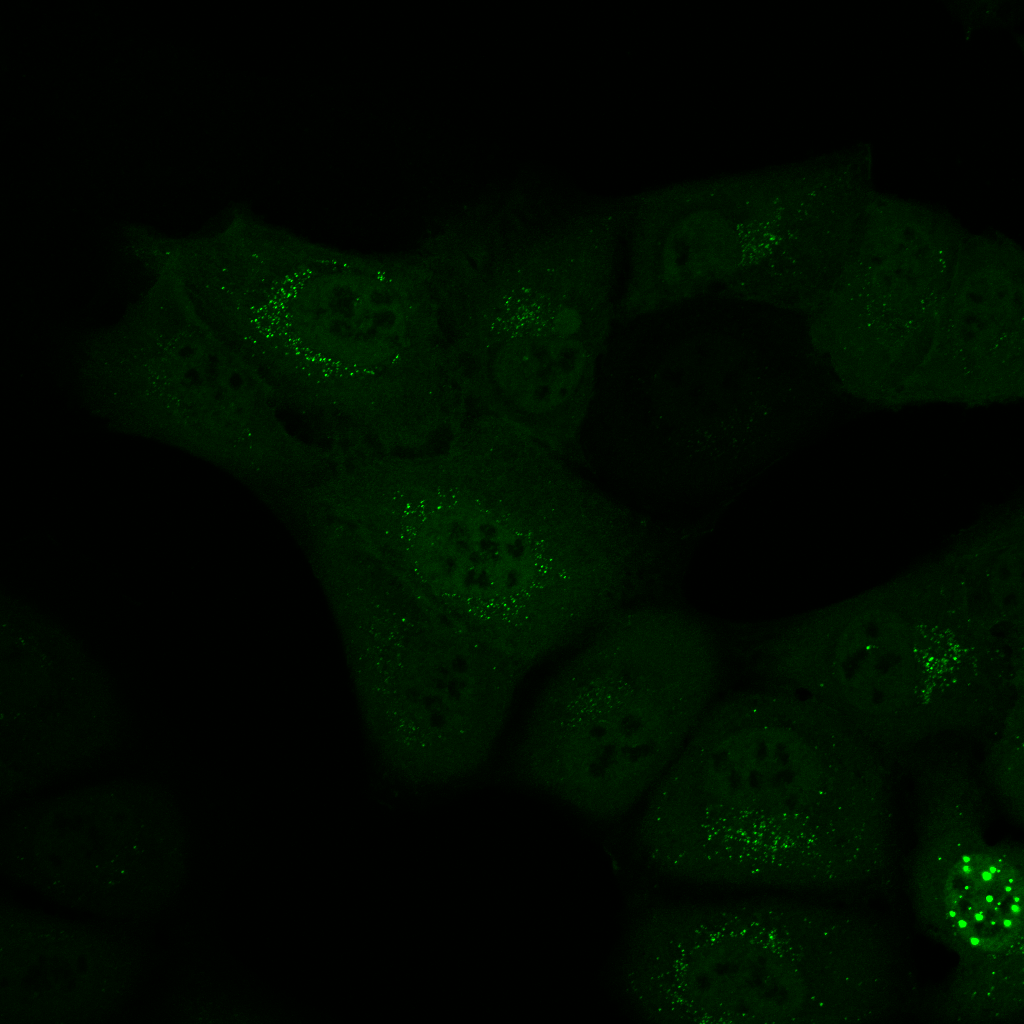

Supplement: Supplementary file 7 — Source Data Fig. 4 [file 44318_2024_44_MOESM7_ESM.zip › Fig 4/Fig 4D/Single channel/Fig4D_GFP_Uncropped_MAX_U20S EMPTY CICD (RGB).tif]

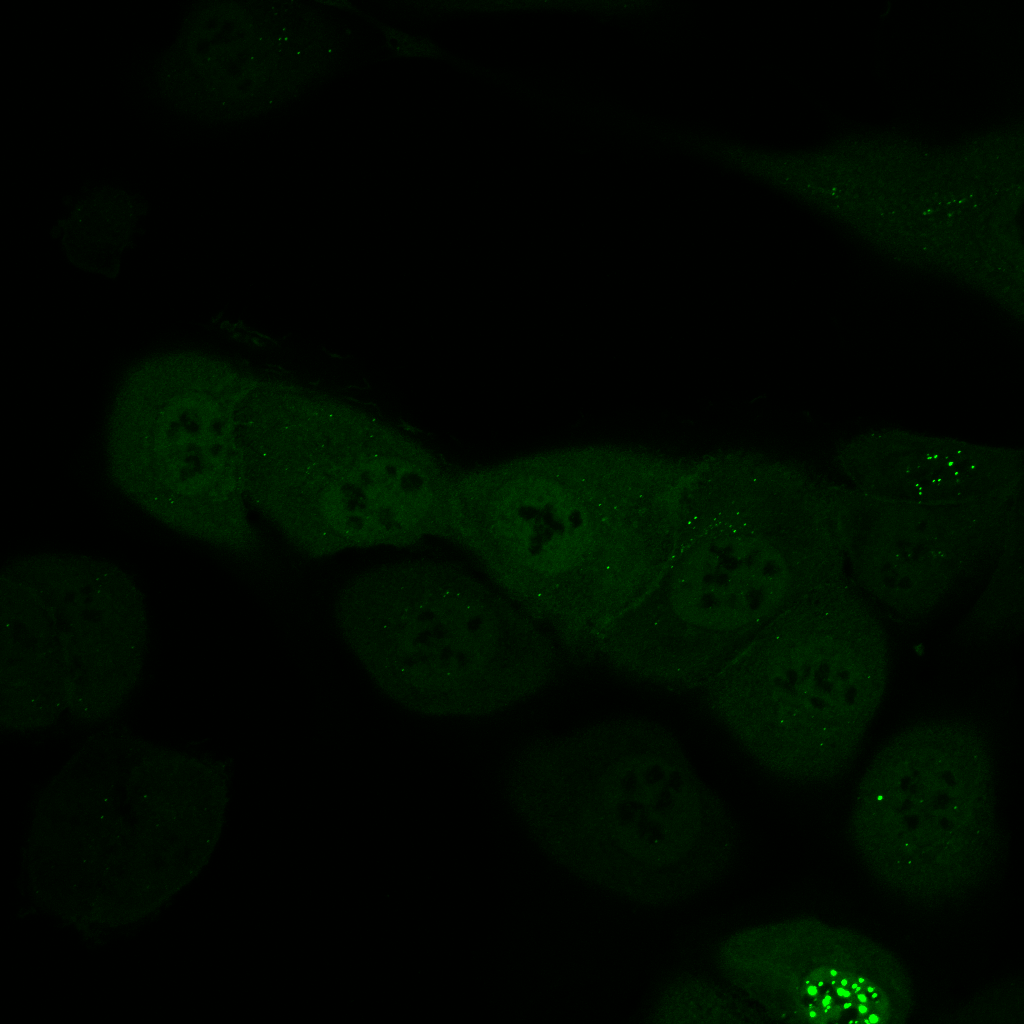

Supplement: Supplementary file 7 — Source Data Fig. 4 [file 44318_2024_44_MOESM7_ESM.zip › Fig 4/Fig 4D/Single channel/Fig4D_GFP_Uncropped_MAX_U20S EMPTY CTRL (RGB).tif]

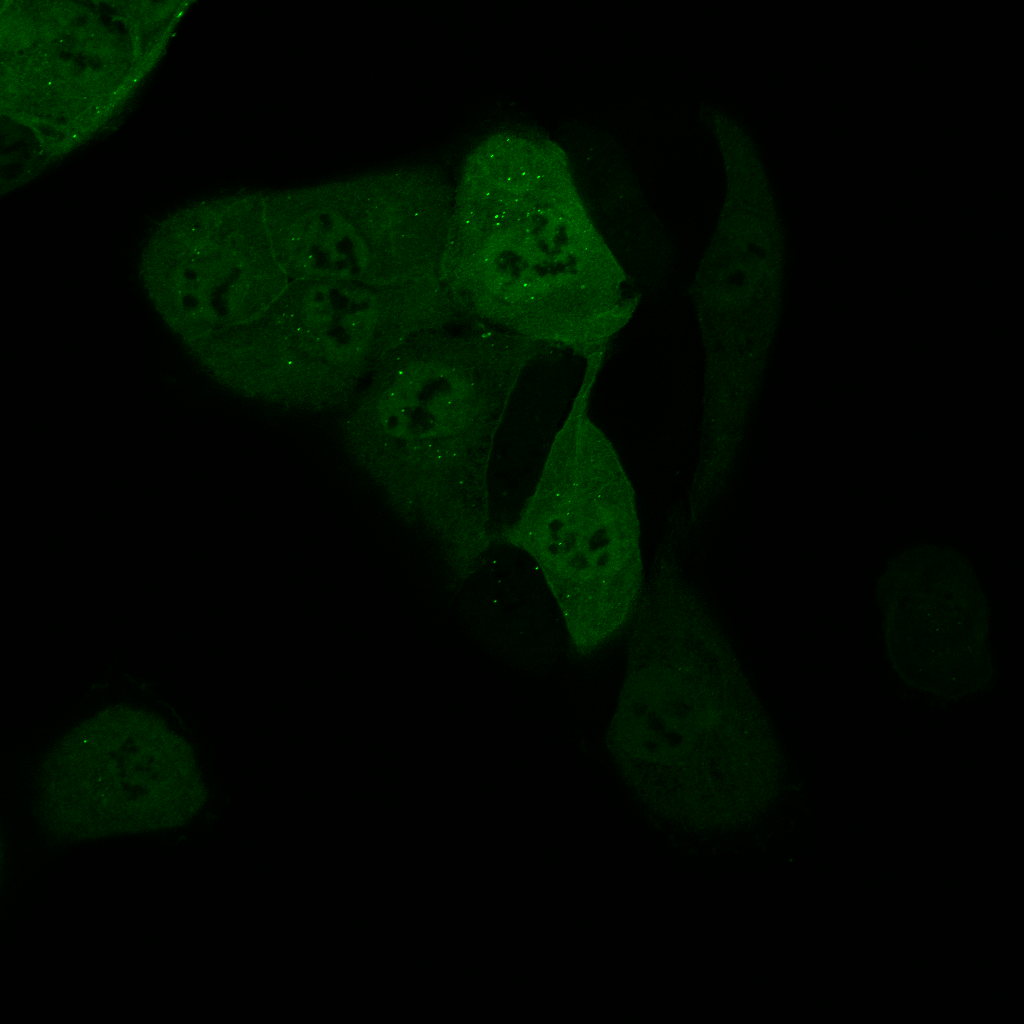

Supplement: Supplementary file 7 — Source Data Fig. 4 [file 44318_2024_44_MOESM7_ESM.zip › Fig 4/Fig 4D/Single channel/Fig4D_GFP_Uncropped_MAX_U20S BB CICD (RGB).tif]

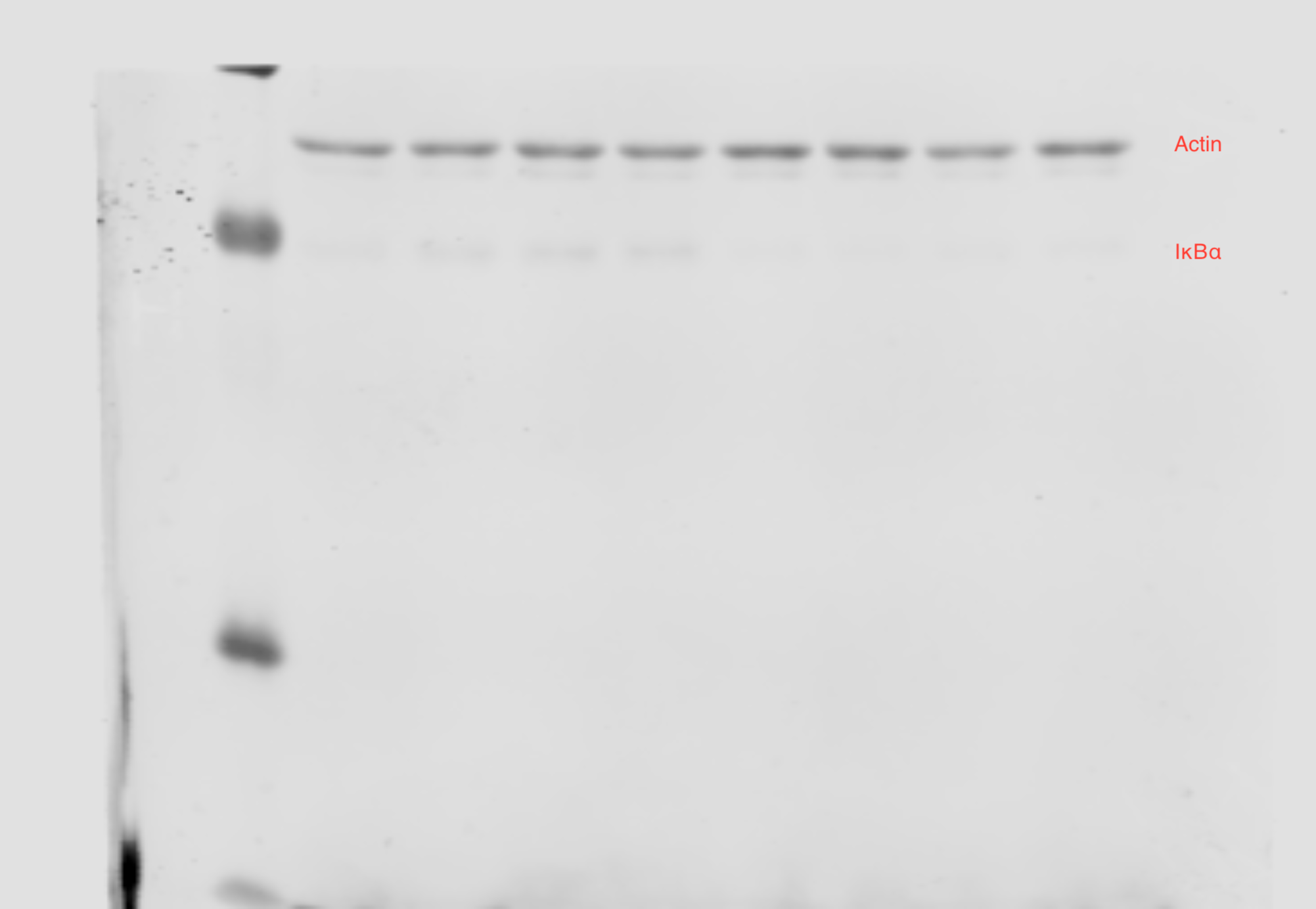

Supplement: Supplementary file 8 — Source Data Fig. 5 [file 44318_2024_44_MOESM8_ESM.zip › Fig 5/Fig 5D/Fig5B_ikba_actin.tif]

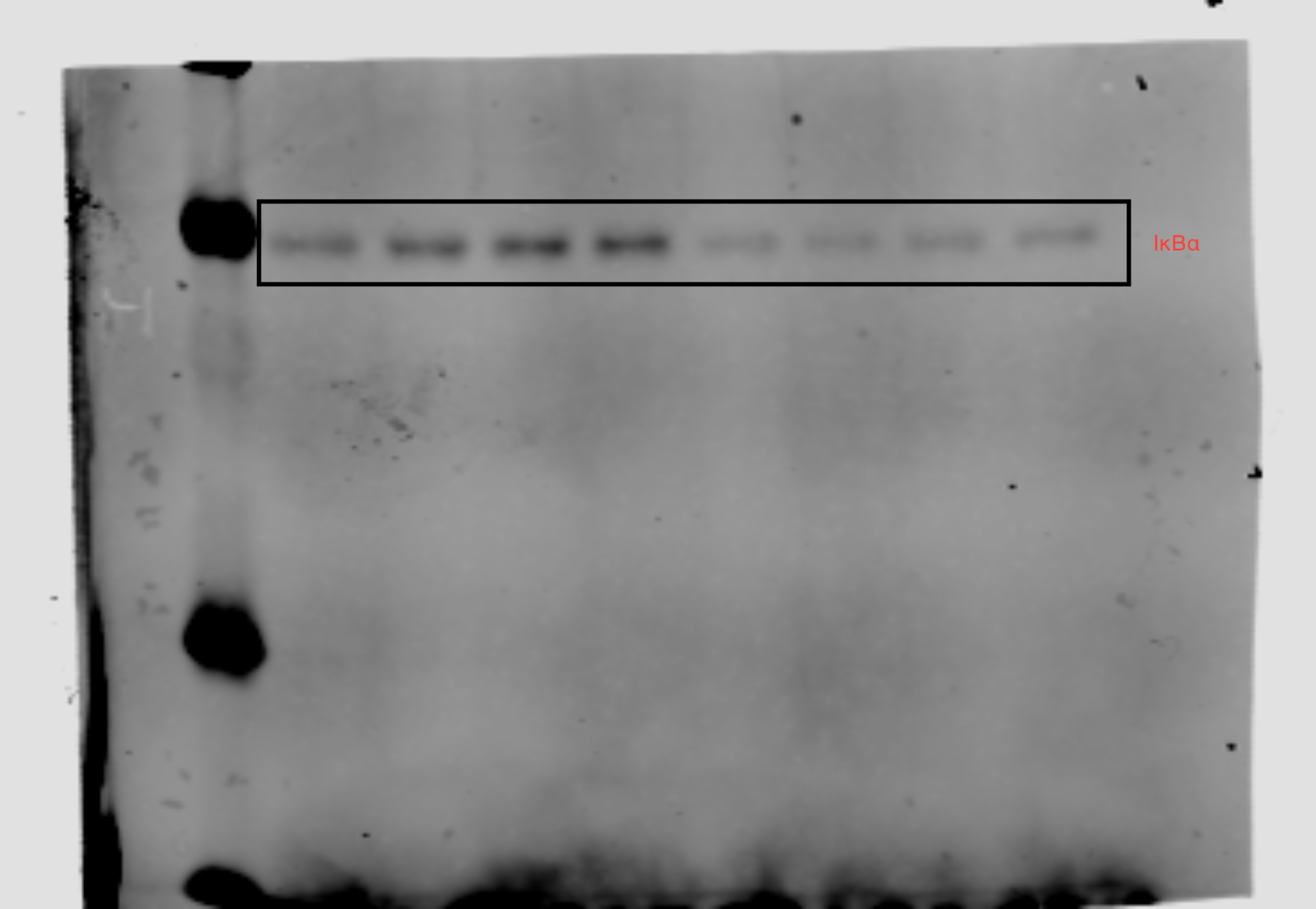

Supplement: Supplementary file 8 — Source Data Fig. 5 [file 44318_2024_44_MOESM8_ESM.zip › Fig 5/Fig 5D/Fig5D_ikba.tif]

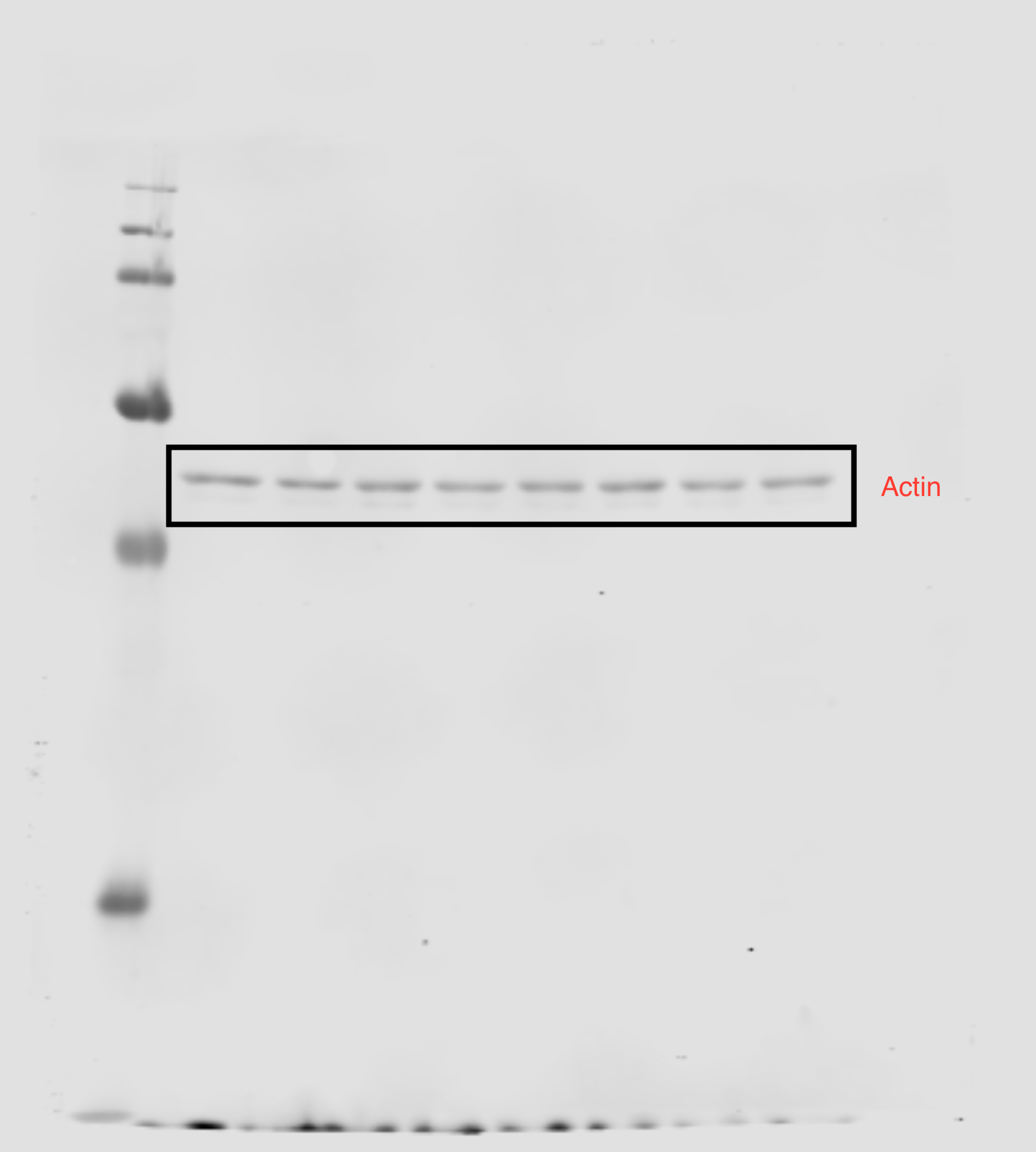

Supplement: Supplementary file 8 — Source Data Fig. 5 [file 44318_2024_44_MOESM8_ESM.zip › Fig 5/Fig 5D/Fig5D_March5_actin.tif]

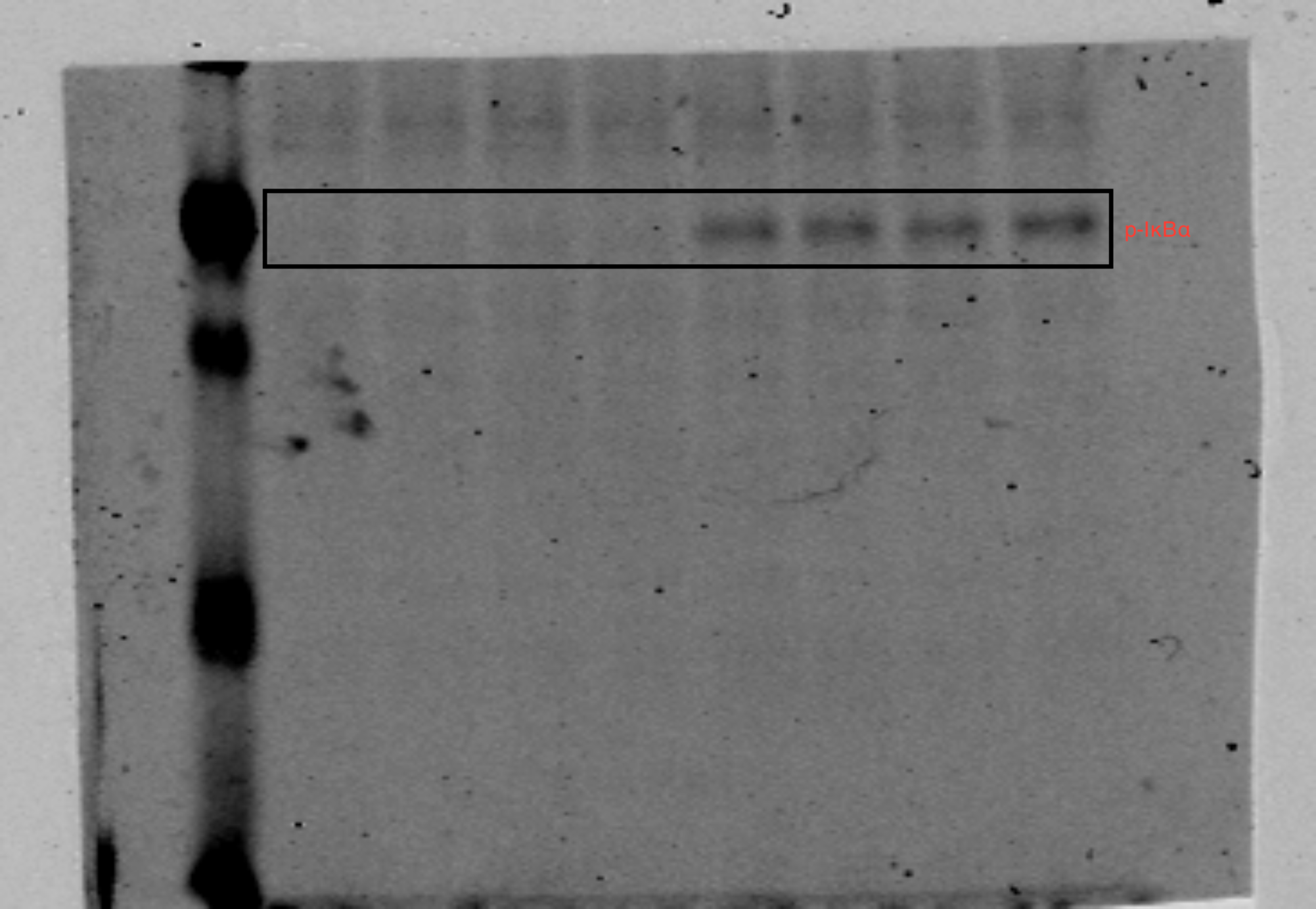

Supplement: Supplementary file 8 — Source Data Fig. 5 [file 44318_2024_44_MOESM8_ESM.zip › Fig 5/Fig 5D/Fig5D_pikba.tif]

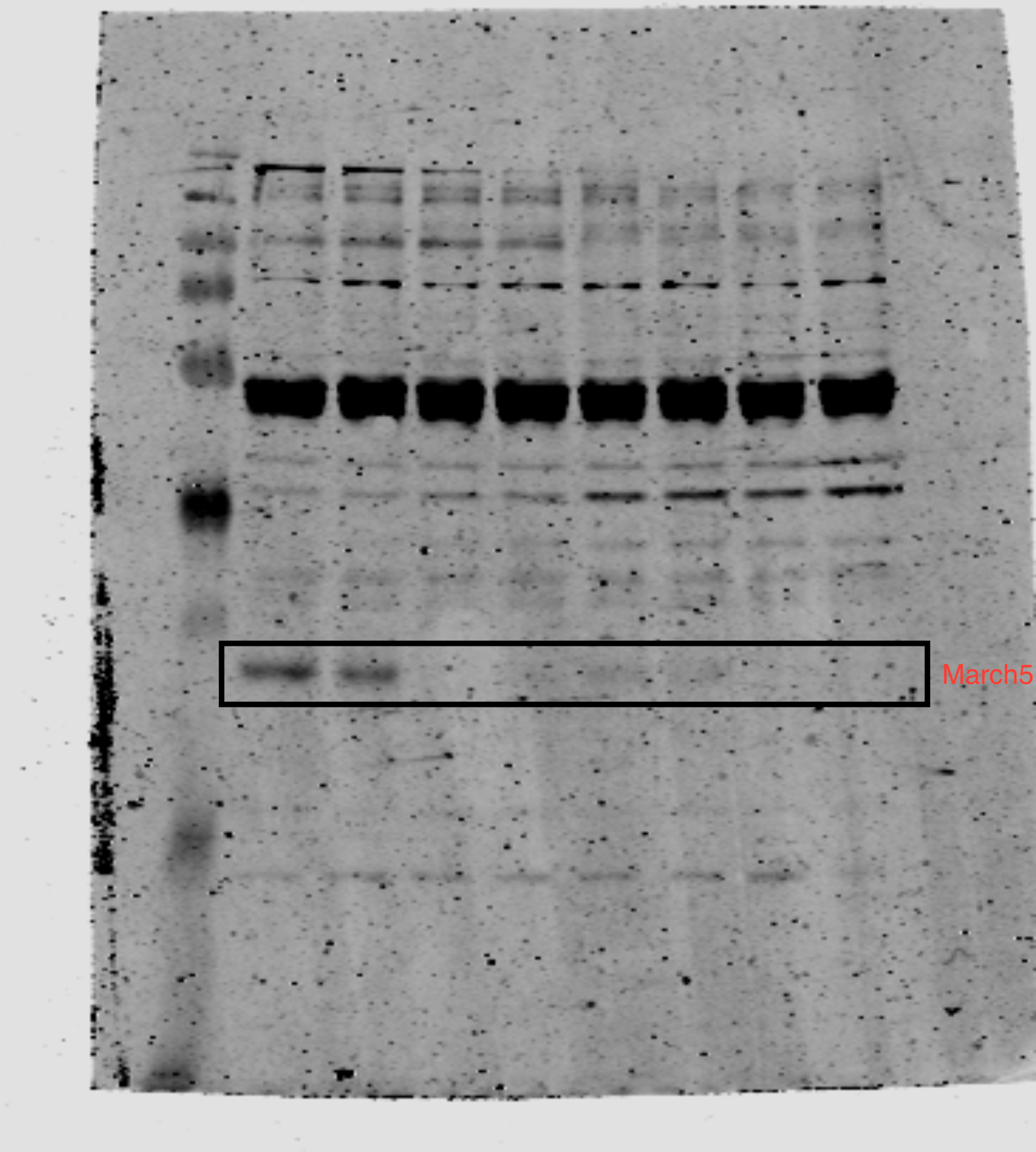

Supplement: Supplementary file 8 — Source Data Fig. 5 [file 44318_2024_44_MOESM8_ESM.zip › Fig 5/Fig 5D/Fig5D_March5.tif]

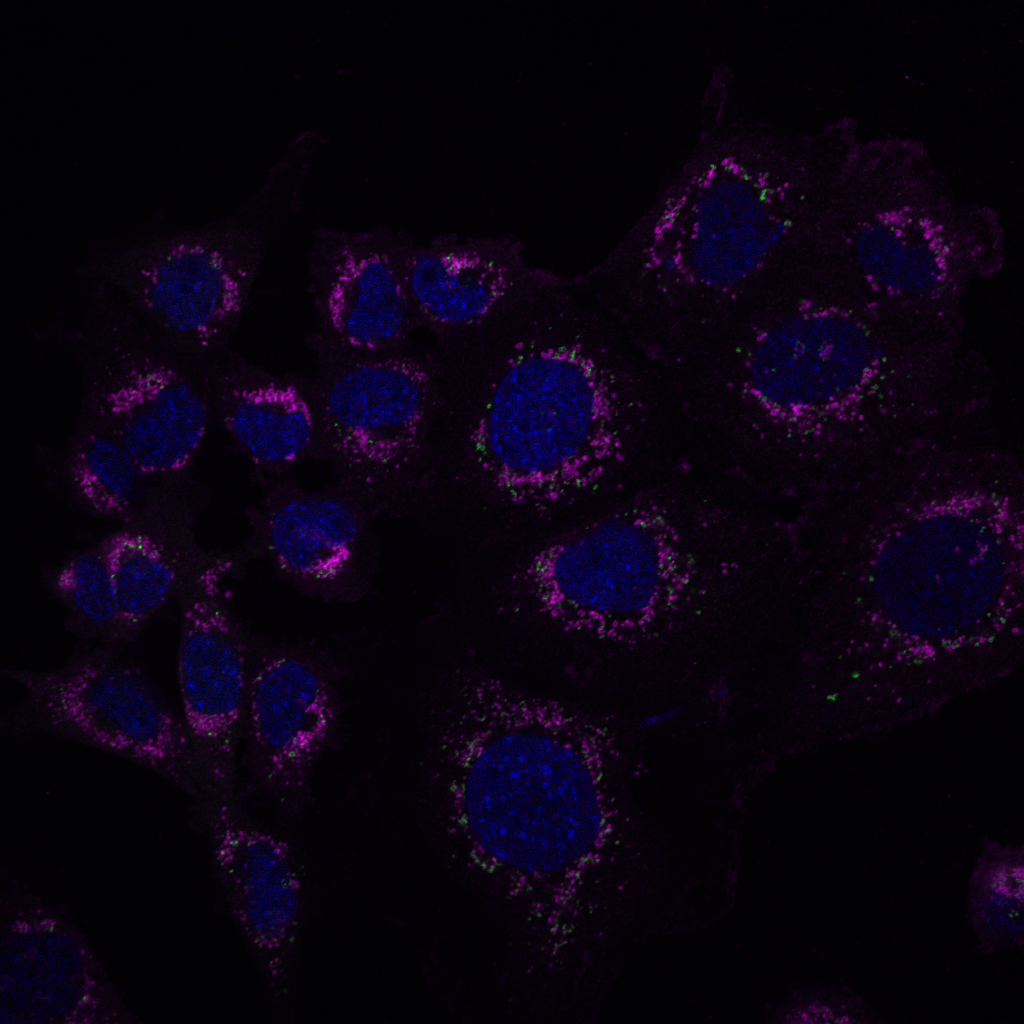

Supplement: Supplementary file 8 — Source Data Fig. 5 [file 44318_2024_44_MOESM8_ESM.zip › Fig 5/Fig 5B/Fig5B_svec_gfp-nemo_empty_tom20_cicd(RGB).tif]

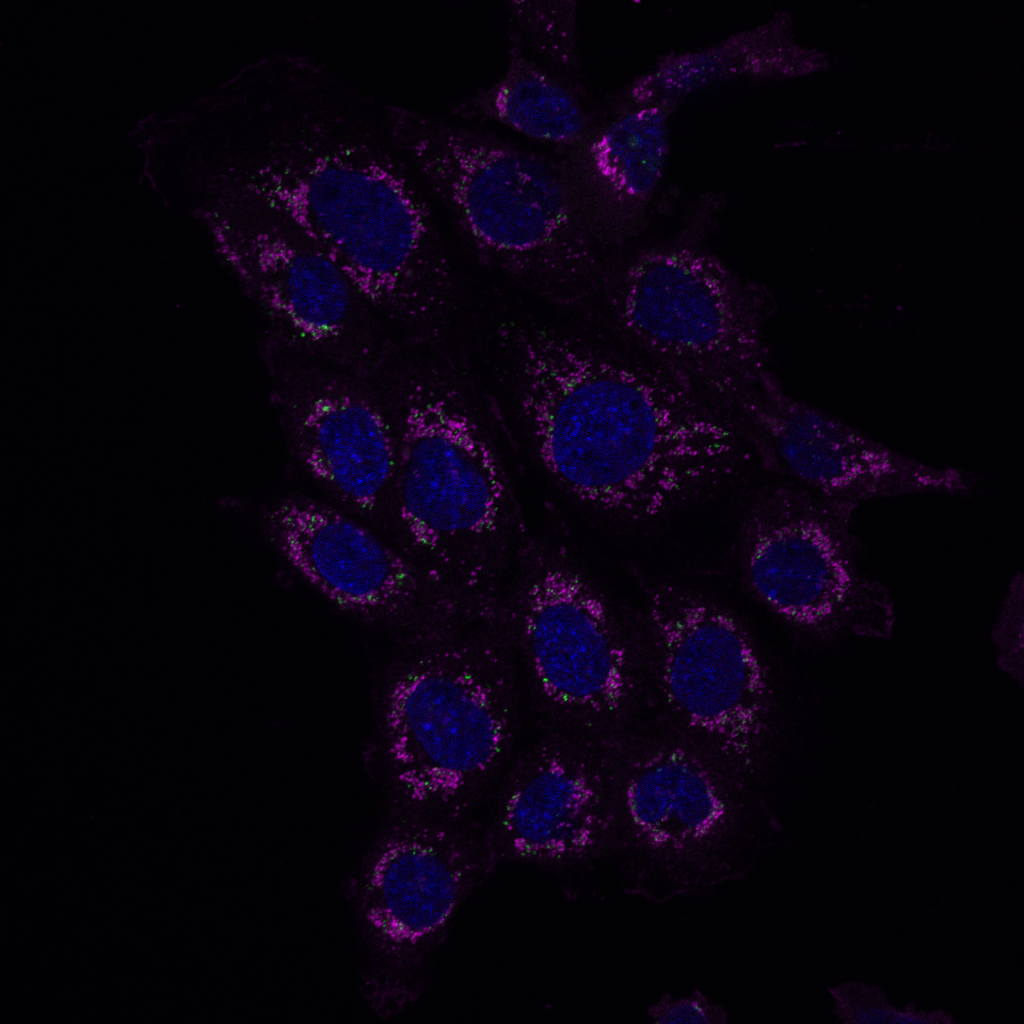

Supplement: Supplementary file 8 — Source Data Fig. 5 [file 44318_2024_44_MOESM8_ESM.zip › Fig 5/Fig 5B/Fig5B_svec_gfp-nemo_pink1_tom20_cicd(RGB).tif]

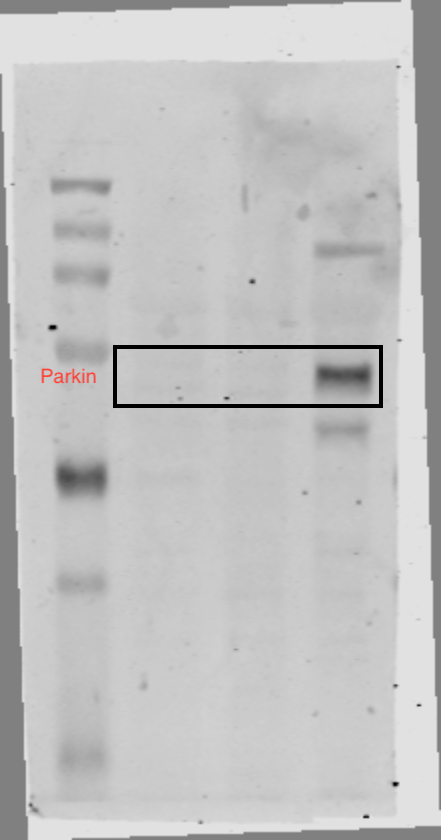

Supplement: Supplementary file 8 — Source Data Fig. 5 [file 44318_2024_44_MOESM8_ESM.zip › Fig 5/Fig 5A/Fig5A_Parkin.tif]

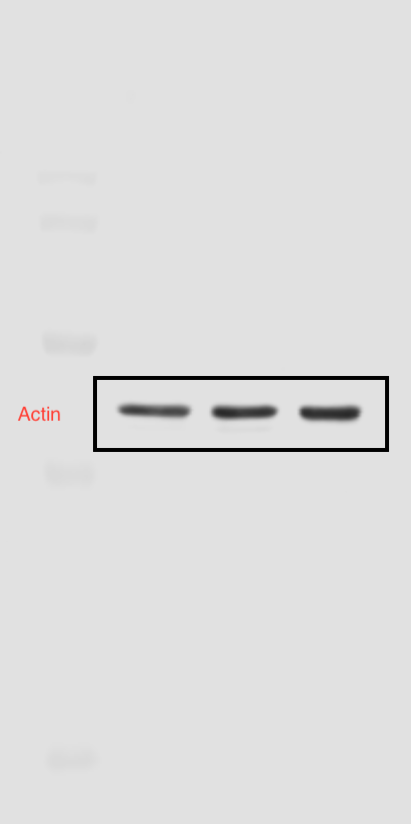

Supplement: Supplementary file 8 — Source Data Fig. 5 [file 44318_2024_44_MOESM8_ESM.zip › Fig 5/Fig 5A/Fig5A_Actin.tif]

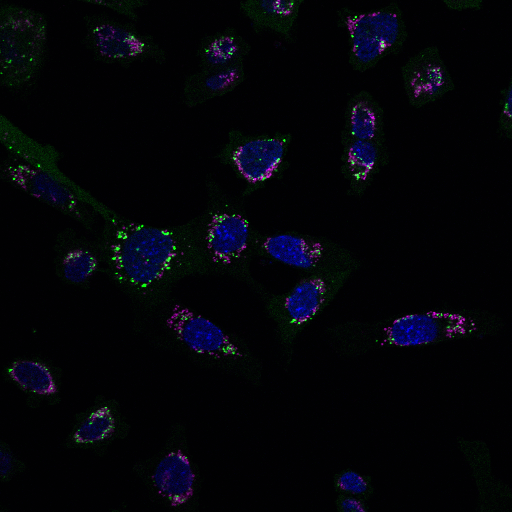

Supplement: Supplementary file 8 — Source Data Fig. 5 [file 44318_2024_44_MOESM8_ESM.zip › Fig 5/Fig 5I/Fig5I_svec gfp-nemo nik tom20 cicd(RGB).tif]

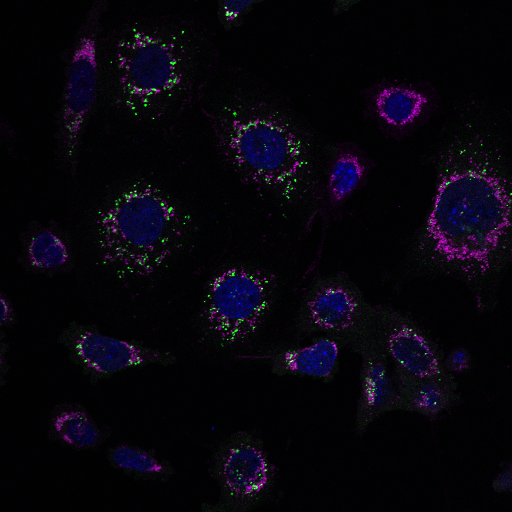

Supplement: Supplementary file 8 — Source Data Fig. 5 [file 44318_2024_44_MOESM8_ESM.zip › Fig 5/Fig 5I/Fig5I_svec gfp-nemo empty tom20 cicd(RGB).tif]

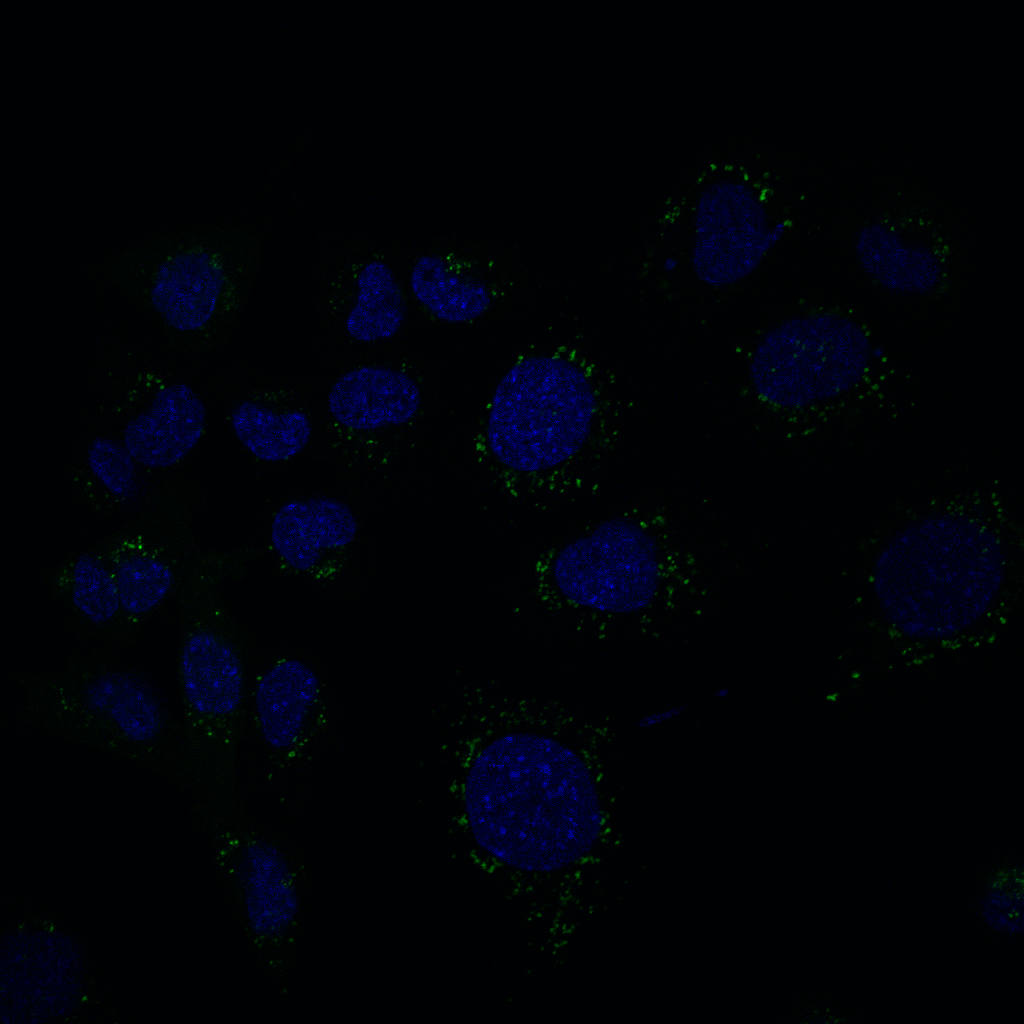

Supplement: Supplementary file 8 — Source Data Fig. 5 [file 44318_2024_44_MOESM8_ESM.zip › Fig 5/Fig 5B/Single channels/Fig5B_DAPI_GFP_svec_gfp-nemo_empty_tom20_cicd(RGB).tif]

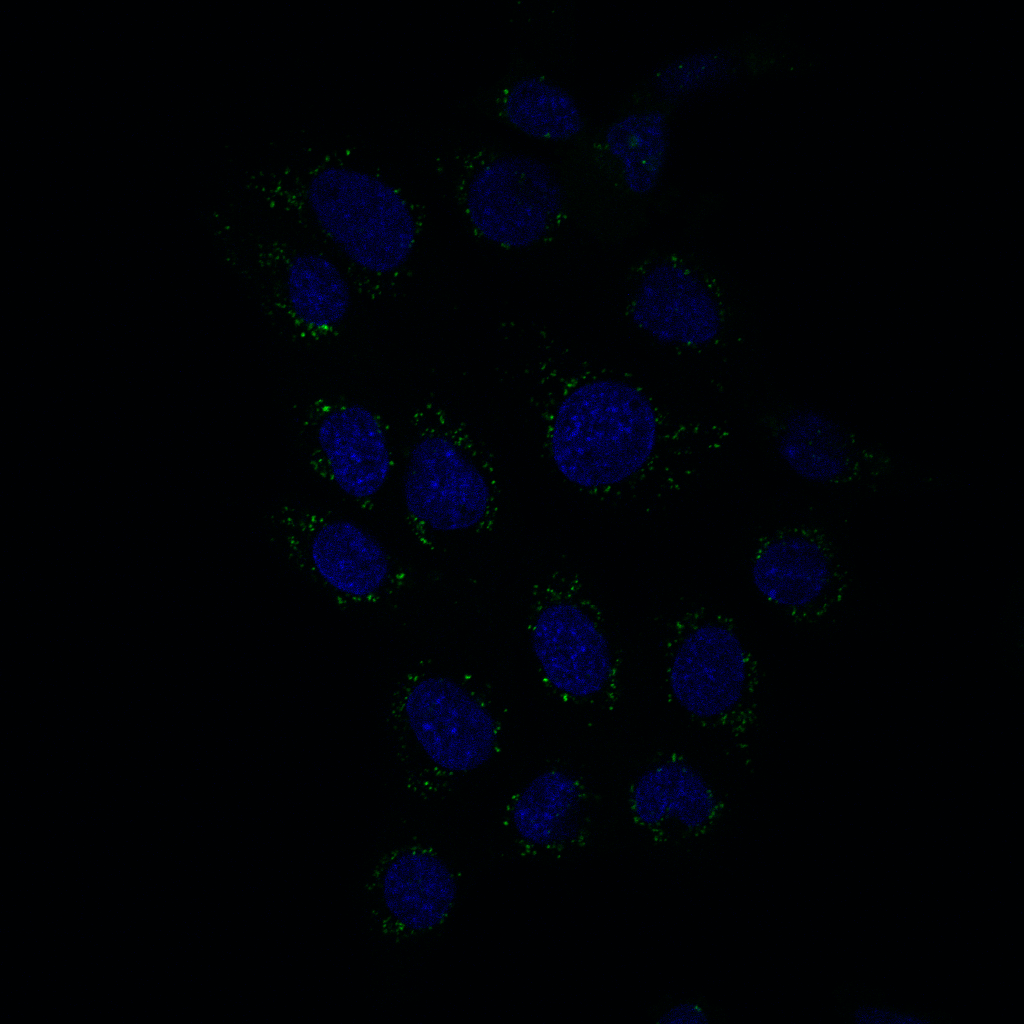

Supplement: Supplementary file 8 — Source Data Fig. 5 [file 44318_2024_44_MOESM8_ESM.zip › Fig 5/Fig 5B/Single channels/Fig5B_DAPI_GFP_svec_gfp-nemo_pink1_tom20_cicd(RGB).tif]

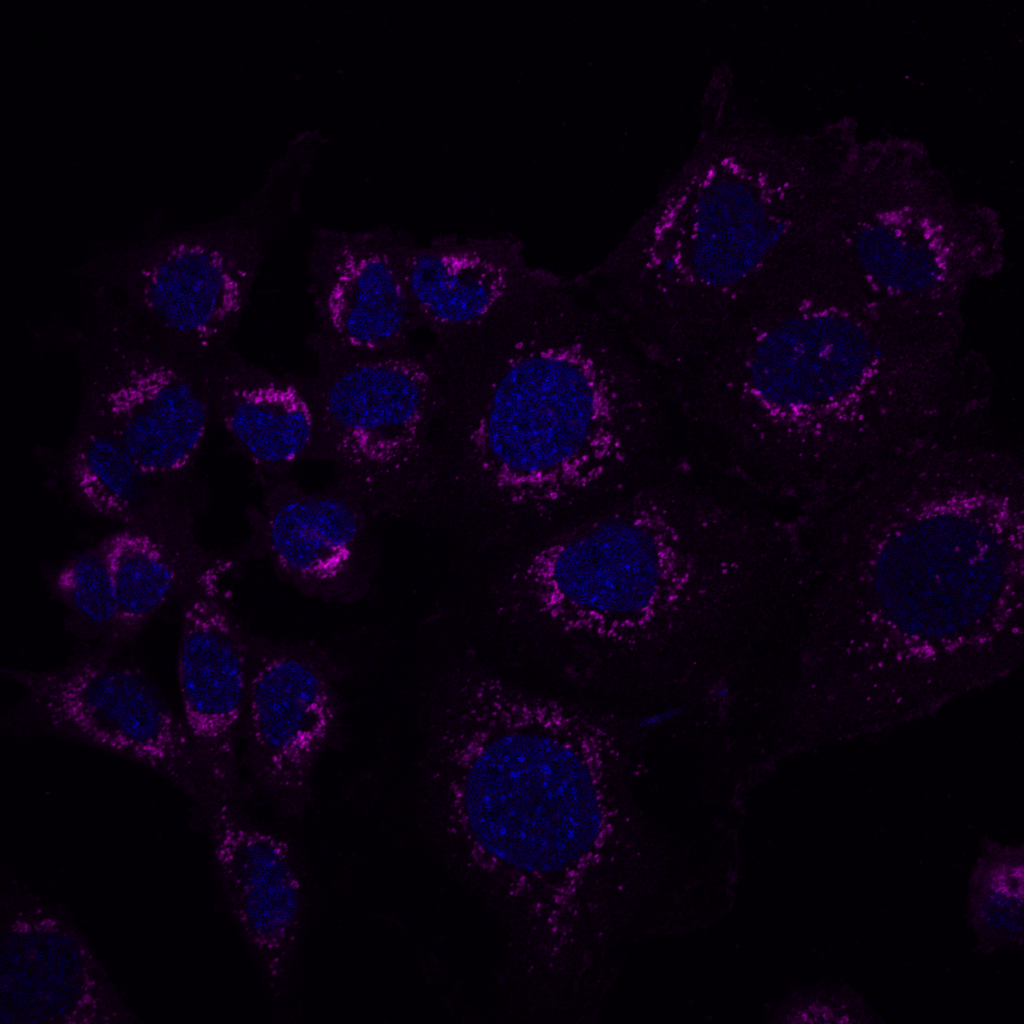

Supplement: Supplementary file 8 — Source Data Fig. 5 [file 44318_2024_44_MOESM8_ESM.zip › Fig 5/Fig 5B/Single channels/Fig5B_DAPI_TOM20_svec_gfp-nemo_empty_tom20_cicd(RGB).tif]

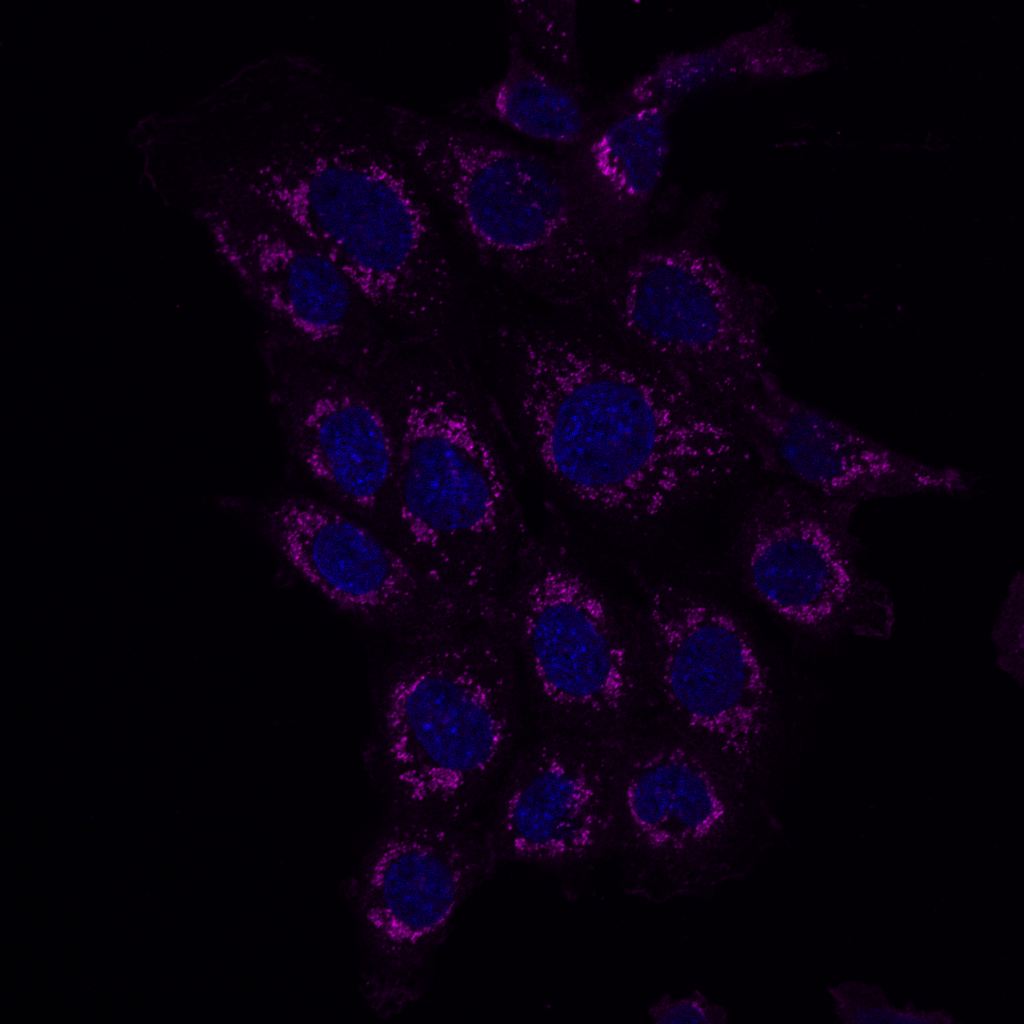

Supplement: Supplementary file 8 — Source Data Fig. 5 [file 44318_2024_44_MOESM8_ESM.zip › Fig 5/Fig 5B/Single channels/Fig5B_DAPI_TOM20_svec_gfp-nemo_pink1_tom20_cicd(RGB).tif]

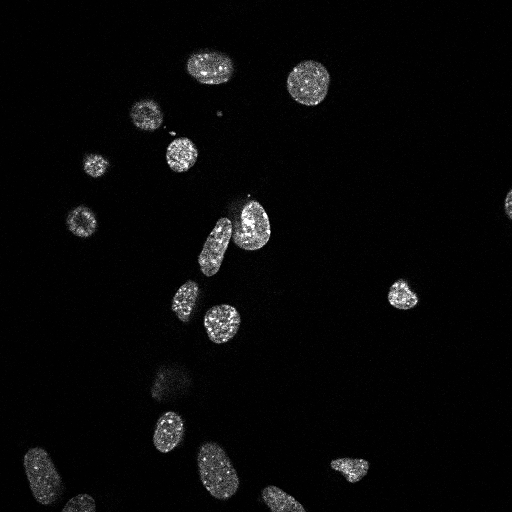

Supplement: Supplementary file 8 — Source Data Fig. 5 [file 44318_2024_44_MOESM8_ESM.zip › Fig 5/Fig 5G/TIFF/Fig_5G-svec gfp-nemo empty fk2 tom20 cicd.tif]

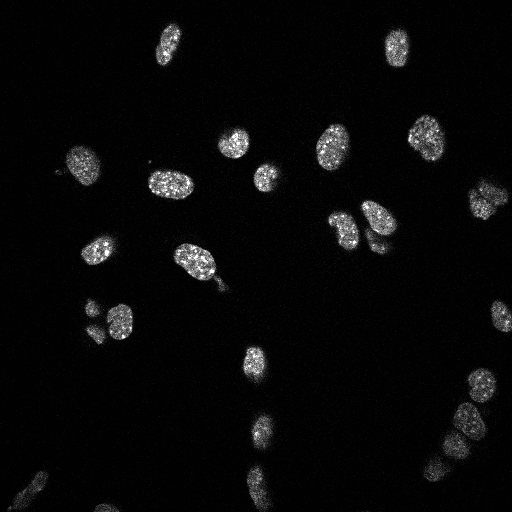

Supplement: Supplementary file 8 — Source Data Fig. 5 [file 44318_2024_44_MOESM8_ESM.zip › Fig 5/Fig 5G/TIFF/Fig_5G-svec gfp-nemo xiap fk2 tom20 cicd.tif]

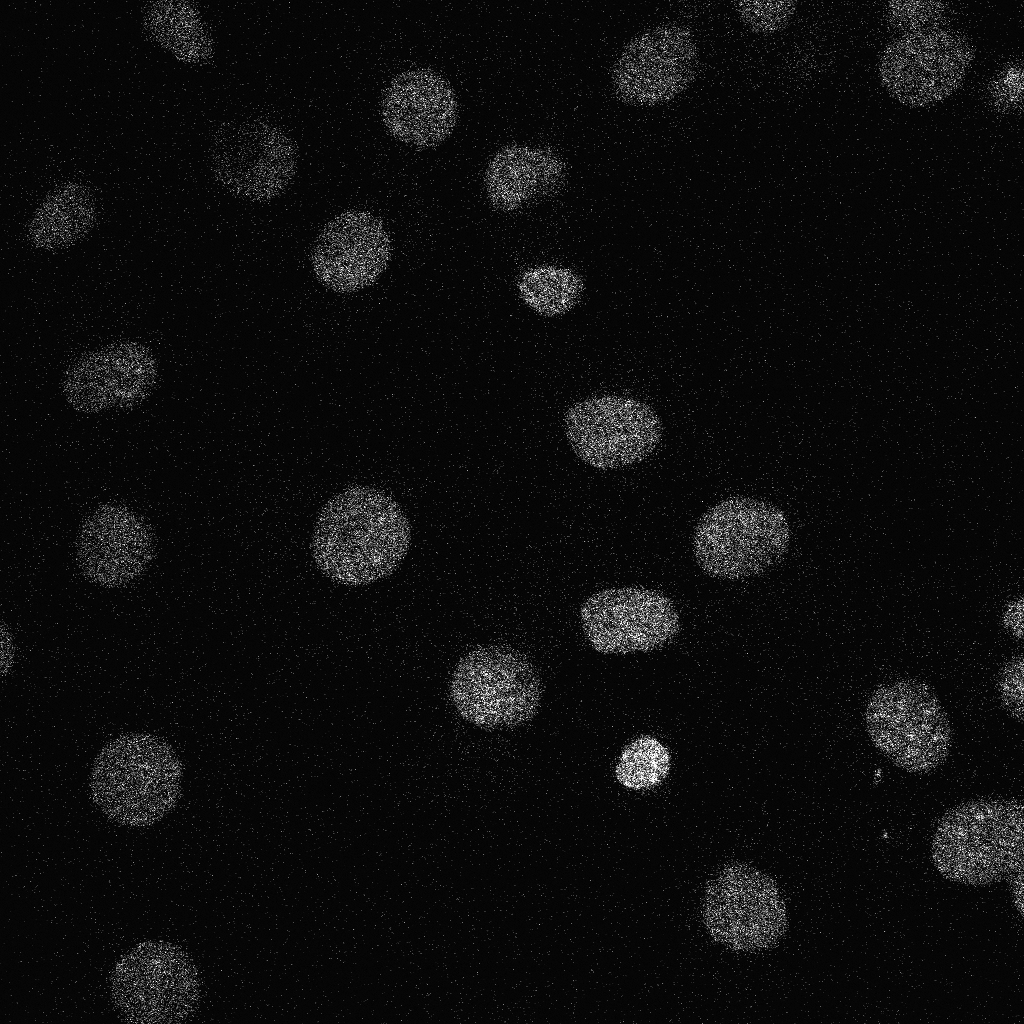

Supplement: Supplementary file 8 — Source Data Fig. 5 [file 44318_2024_44_MOESM8_ESM.zip › Fig 5/Fig 5E/TIFF/Fig_5E-svec_empty_gfp-nemo_ubcj2_tom20_cicd.tif]

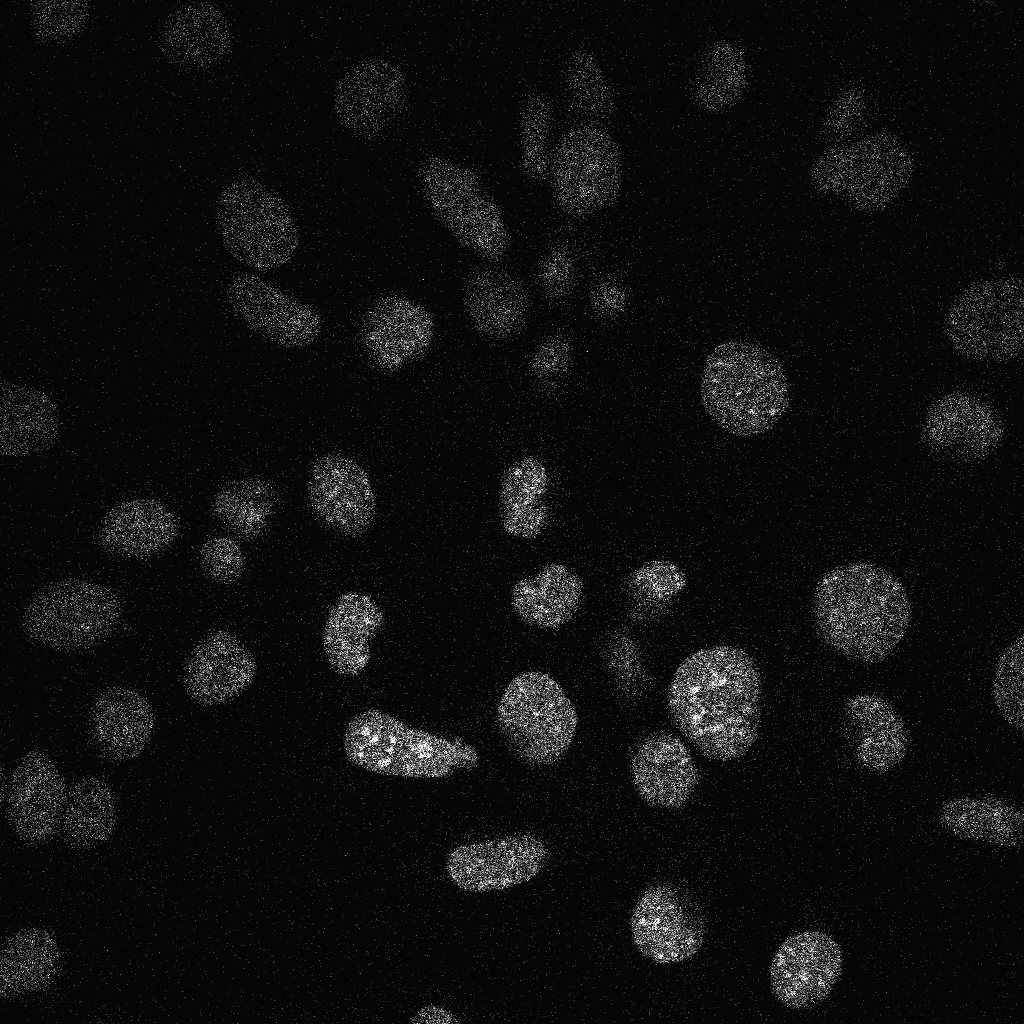

Supplement: Supplementary file 8 — Source Data Fig. 5 [file 44318_2024_44_MOESM8_ESM.zip › Fig 5/Fig 5E/TIFF/Fig_5E-svec_mul1march5_gfp-nemo_ubcj2_tom20_cicd.tif]

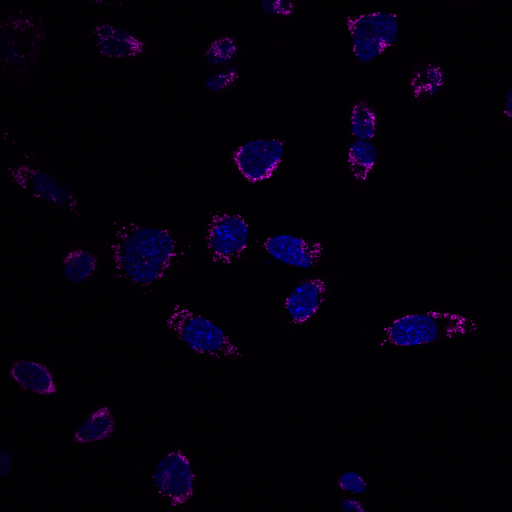

Supplement: Supplementary file 8 — Source Data Fig. 5 [file 44318_2024_44_MOESM8_ESM.zip › Fig 5/Fig 5I/Single channels/Fig5I_DAPI_TOM20_svec gfp-nemo nik tom20 cicd(RGB).tif]

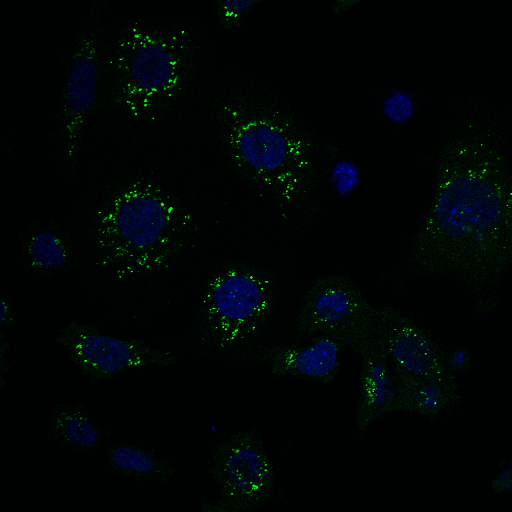

Supplement: Supplementary file 8 — Source Data Fig. 5 [file 44318_2024_44_MOESM8_ESM.zip › Fig 5/Fig 5I/Single channels/Fig5I_DAPI_GFP_svec gfp-nemo empty tom20 cicd(RGB).tif]

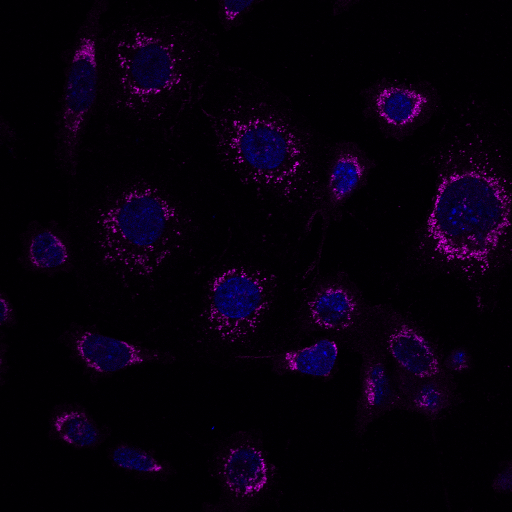

Supplement: Supplementary file 8 — Source Data Fig. 5 [file 44318_2024_44_MOESM8_ESM.zip › Fig 5/Fig 5I/Single channels/Fig5I_DAPI_TOM20_svec gfp-nemo empty tom20 cicd(RGB).tif]

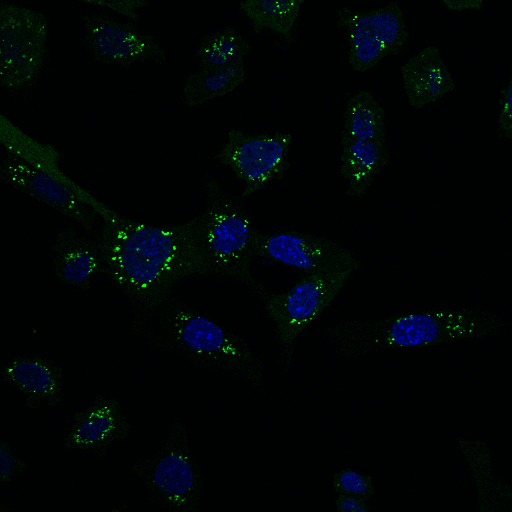

Supplement: Supplementary file 8 — Source Data Fig. 5 [file 44318_2024_44_MOESM8_ESM.zip › Fig 5/Fig 5I/Single channels/Fig5I_DAPI_GFP_svec gfp-nemo nik tom20 cicd(RGB).tif]

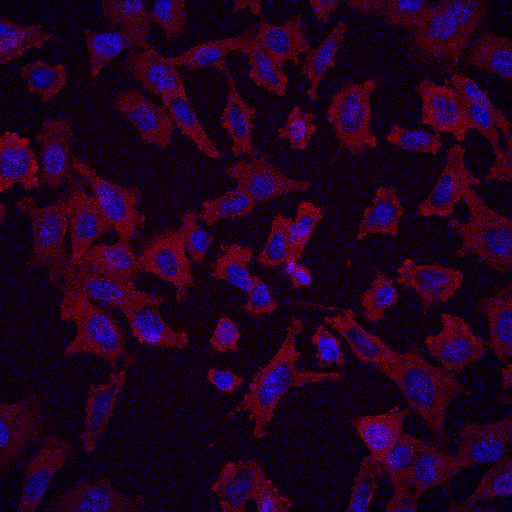

Supplement: Supplementary file 9 — Source Data Fig. 6 [file 44318_2024_44_MOESM9_ESM.zip › Fig 6/Fig 6C/Fig_6C-svec empty p65 cytc dmso.tif]

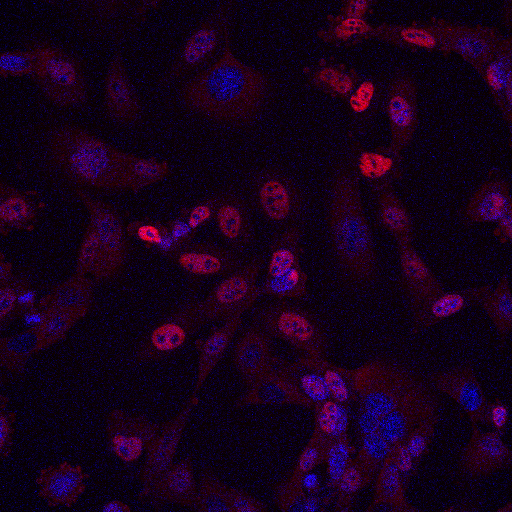

Supplement: Supplementary file 9 — Source Data Fig. 6 [file 44318_2024_44_MOESM9_ESM.zip › Fig 6/Fig 6C/Fig_6C-svec empty p65 cytc rapqvd.tif]

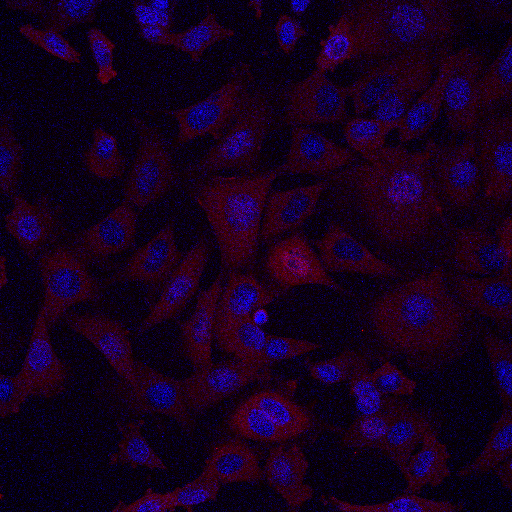

Supplement: Supplementary file 9 — Source Data Fig. 6 [file 44318_2024_44_MOESM9_ESM.zip › Fig 6/Fig 6C/Fig_6C-svec bb p65 cytc cicd.tif]

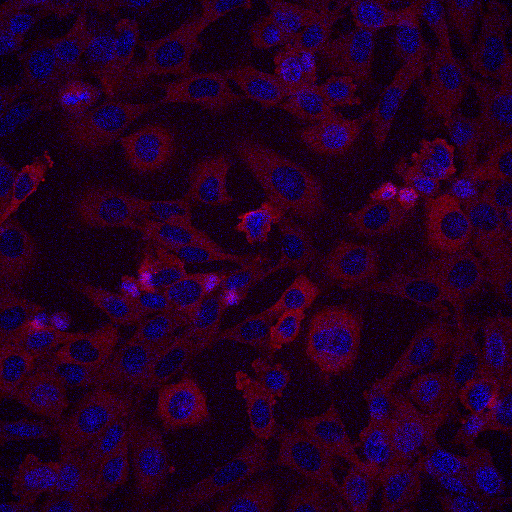

Supplement: Supplementary file 9 — Source Data Fig. 6 [file 44318_2024_44_MOESM9_ESM.zip › Fig 6/Fig 6C/Fig_6C-svec bb p65 cytc dmso.tif]

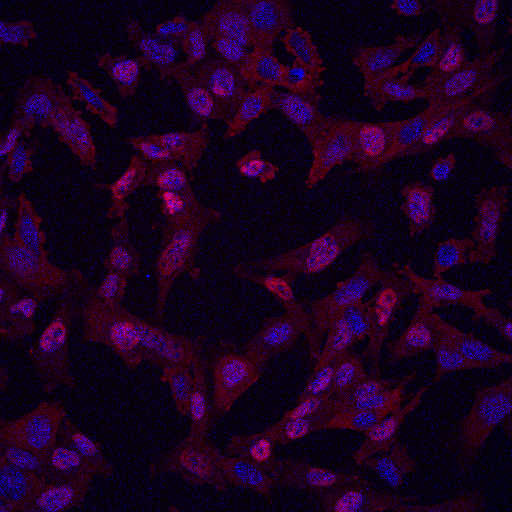

Supplement: Supplementary file 9 — Source Data Fig. 6 [file 44318_2024_44_MOESM9_ESM.zip › Fig 6/Fig 6C/Fig_6C-svec empty p65 cytc cicd.tif]

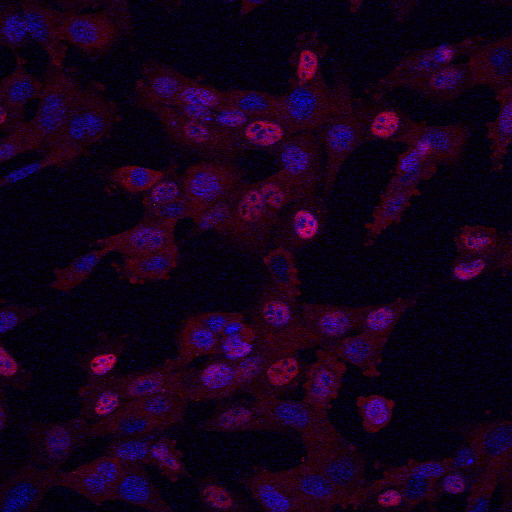

Supplement: Supplementary file 9 — Source Data Fig. 6 [file 44318_2024_44_MOESM9_ESM.zip › Fig 6/Fig 6C/Fig_6C-svec bb p65 cytc rapqvd.tif]

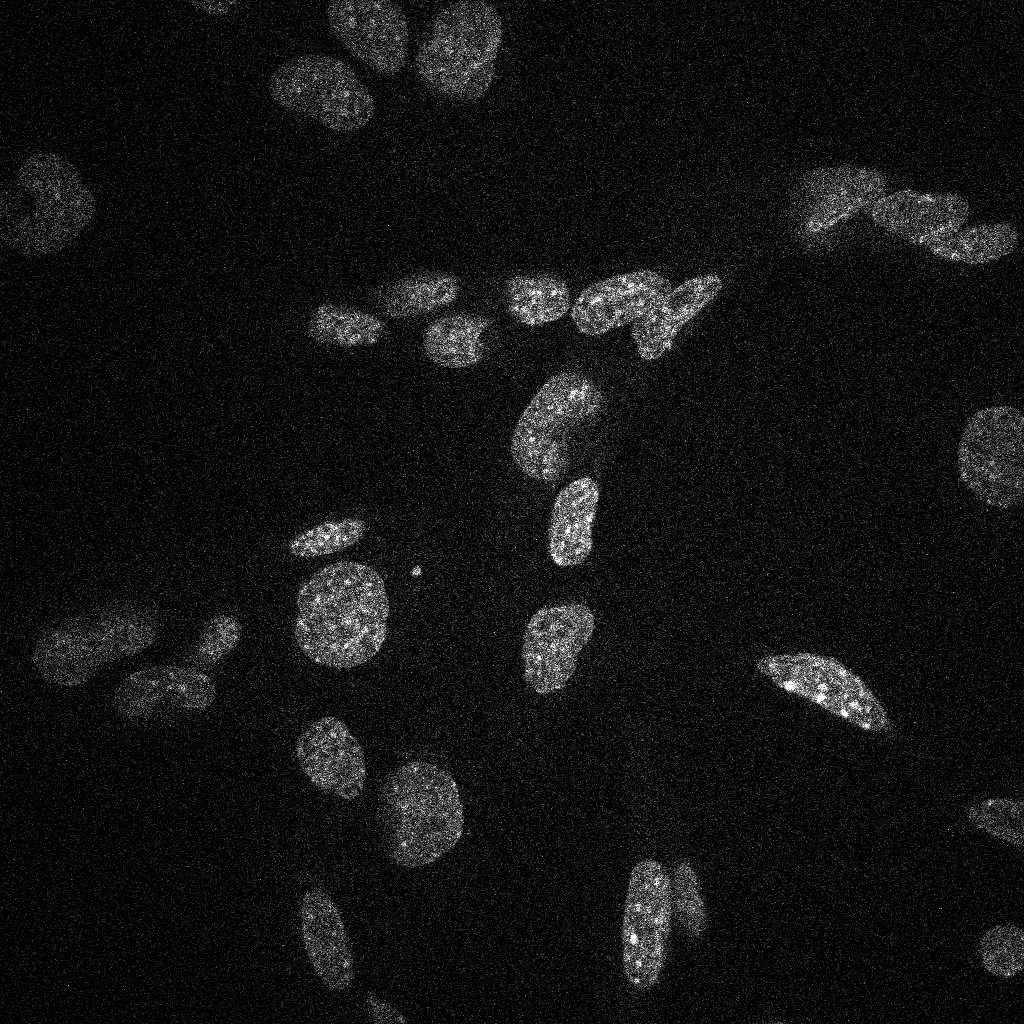

Supplement: Supplementary file 9 — Source Data Fig. 6 [file 44318_2024_44_MOESM9_ESM.zip › Fig 6/Fig 6A/TIFF/Fig6-A-svec gfp-nemo fk2 tom20 cicd.tif]

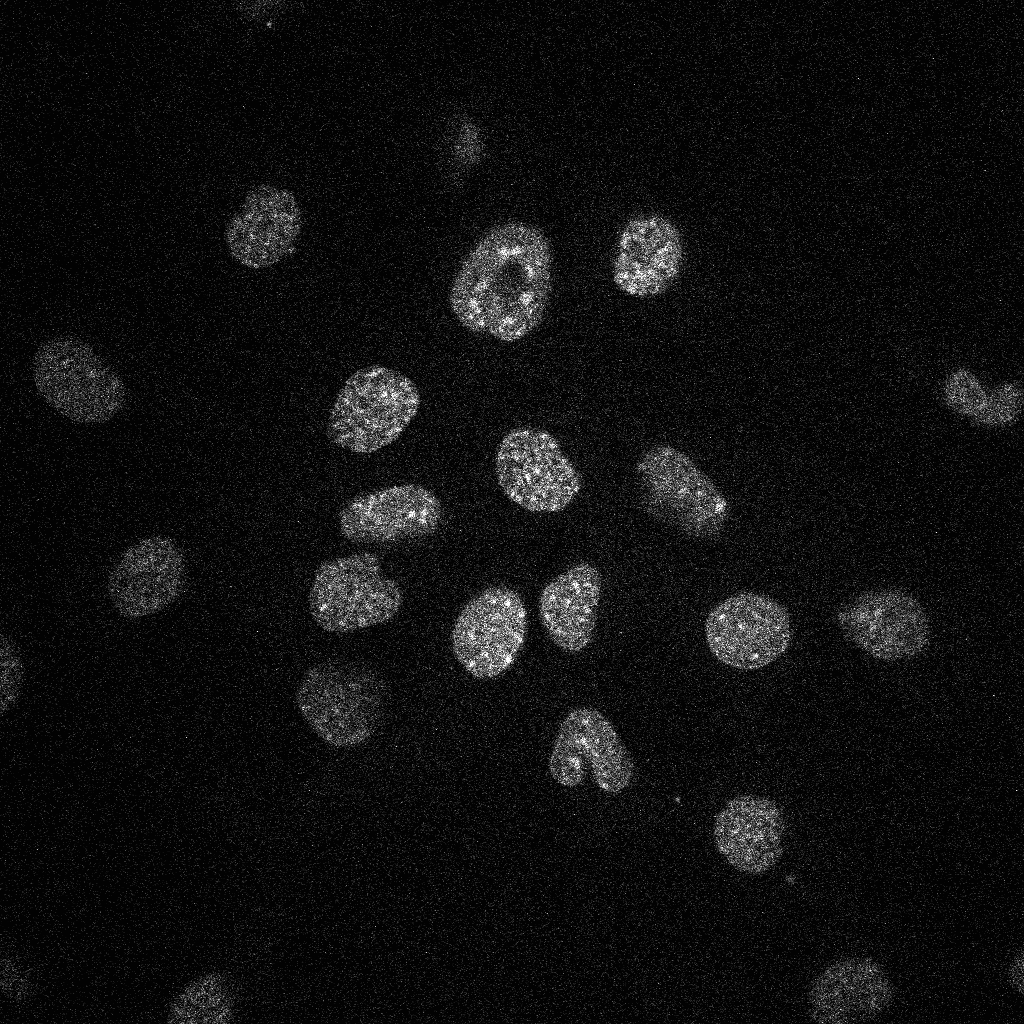

Supplement: Supplementary file 9 — Source Data Fig. 6 [file 44318_2024_44_MOESM9_ESM.zip › Fig 6/Fig 6A/TIFF/Fig6_A-svec gfp-nemo fk2 tom20 ctrl.tif]

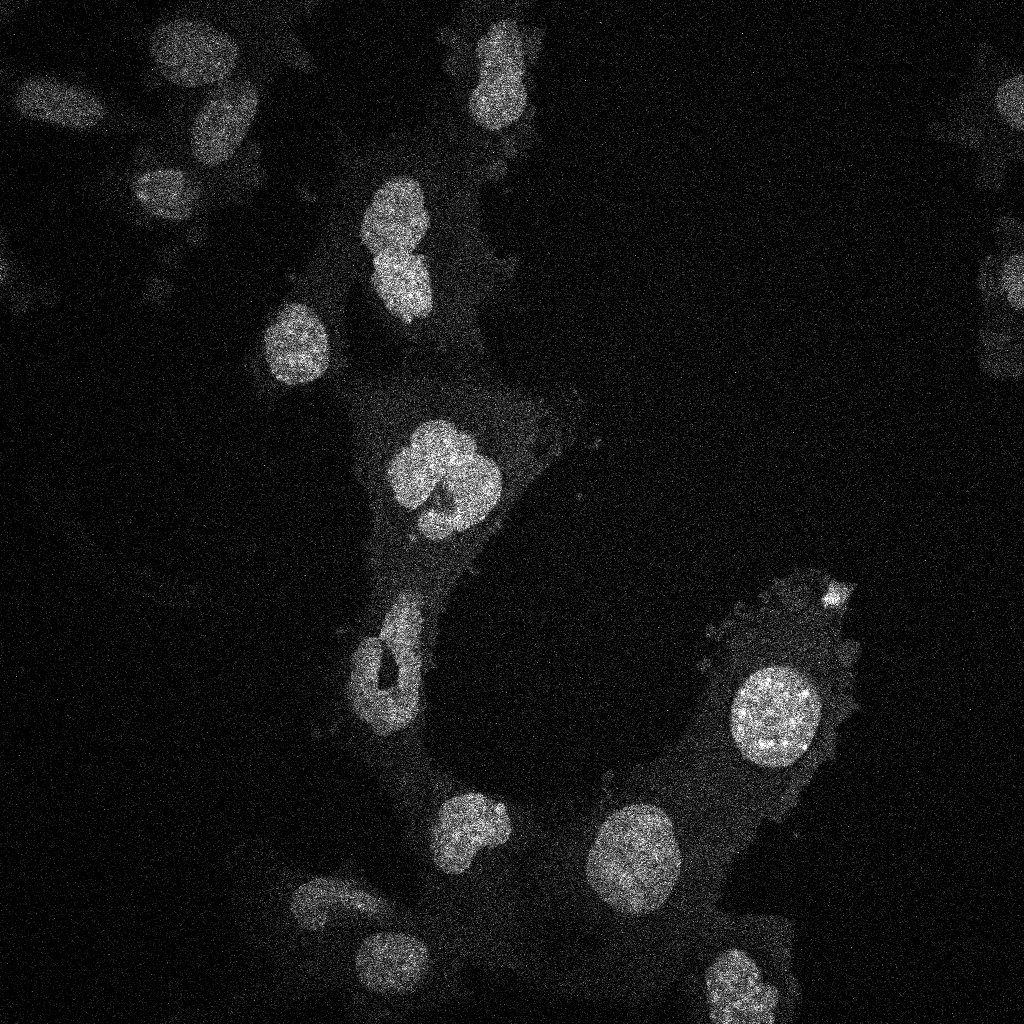

Supplement: Supplementary file 9 — Source Data Fig. 6 [file 44318_2024_44_MOESM9_ESM.zip › Fig 6/Fig 6A/TIFF/Fig6_A-svec gfp-nemo fk2 tom20 rapqvd.tif]
