# Supplementary material for: Mitochondrial outer membrane integrity regulates a ubiquitin-dependent and NF-κB-mediated inflammatory response
Source: EMBO J. 2024 Feb 9;43(6):904–30. doi: 10.1038/s44318-024-00044-1 (PMC10943237; doi:10.1038/s44318-024-00044-1)
Supplement: Supplementary file 12 — Expanded View Figures [file 44318_2024_44_MOESM12_ESM.pdf]

Expanded View Figures

Figure EV1. Ubiquitylation of mitochondria is dependent on MOMP by BAX/BAK pores, but independent of caspase activity.

(**A**) U2OS EMPTY^{CRISPR} and BAX/BAK^{CRISPR} cells were treated with 10 μM ABT-737, 2 μM S63845 with or without 20 μM Q-VD-OPh. Cell death was determined using Sytox Green inclusion normalised to starting confluence. Graph is representative of three independent experiments and displays mean values ± s.e.m. (error bars) of technical triplicates. (**B**) Lysates from U2OS EMPTY^{CRISPR} and BAX/BAK^{CRISPR} cells were blotted for BAX, BAK and Actin. (**C**) SVEC4-10 EMPTY^{CRISPR} and BAX/BAK^{CRISPR} cells were treated for 1 h with 10 μM ABT-737, 10 μM S63845 and 30 μM Q-VD-OPh. Mitochondria were isolated using digitonin fractionation buffer and blotted for ubiquitin (UBCJ2), BAX, BAK, HSP60 and Actin. (**D**) SVEC4-10 cells were treated for 1 h with 10 μM ABT-737, 10 μM S63845 with or without 30 μM Q-VD-OPh. Mitochondria were isolated using digitonin fractionation buffer and lysates were blotted for ubiquitin (UBCJ2), HSP60 and actin. (**E**) U2OS cells were treated for 7 h with 10 μM ABT-737, 2 μM S63845 and 20 μM Q-VD-OPh with or without the addition of 10 μM MG-132. Lysates were blotted for ubiquitin (UBCJ2) and GAPDH. (**F**) U2OS cells were treated for 24 h with 10 μM ABT-737, 2 μM S63845 and 20 μM Q-VD-OPh with or without the addition of 10 μM MG-132. Mitochondrial depletion was assessed by blotting for several mitochondrial proteins and GAPDH. (**G**) U2OS cells were treated for 7 h with 10 μM ABT-737, 2 μM S63845 and 20 μM Q-VD-OPh with or without the addition of 1 μM TAK-243. Lysates were blotted for ubiquitin (UBCJ2) and GAPDH. (**H**) U2OS cells were treated for 24 h with 10 μM ABT-737, 2 μM S63845 and 20 μM Q-VD-OPh with or without the addition of 1 μM TAK-243. Mitochondrial depletion was assessed by blotting for several mitochondrial proteins and GAPDH. Data information: (**C**, **D**, **E**, **F**, **G**, **H**) blots are representative of three independent experiments.

 © The Author(s)

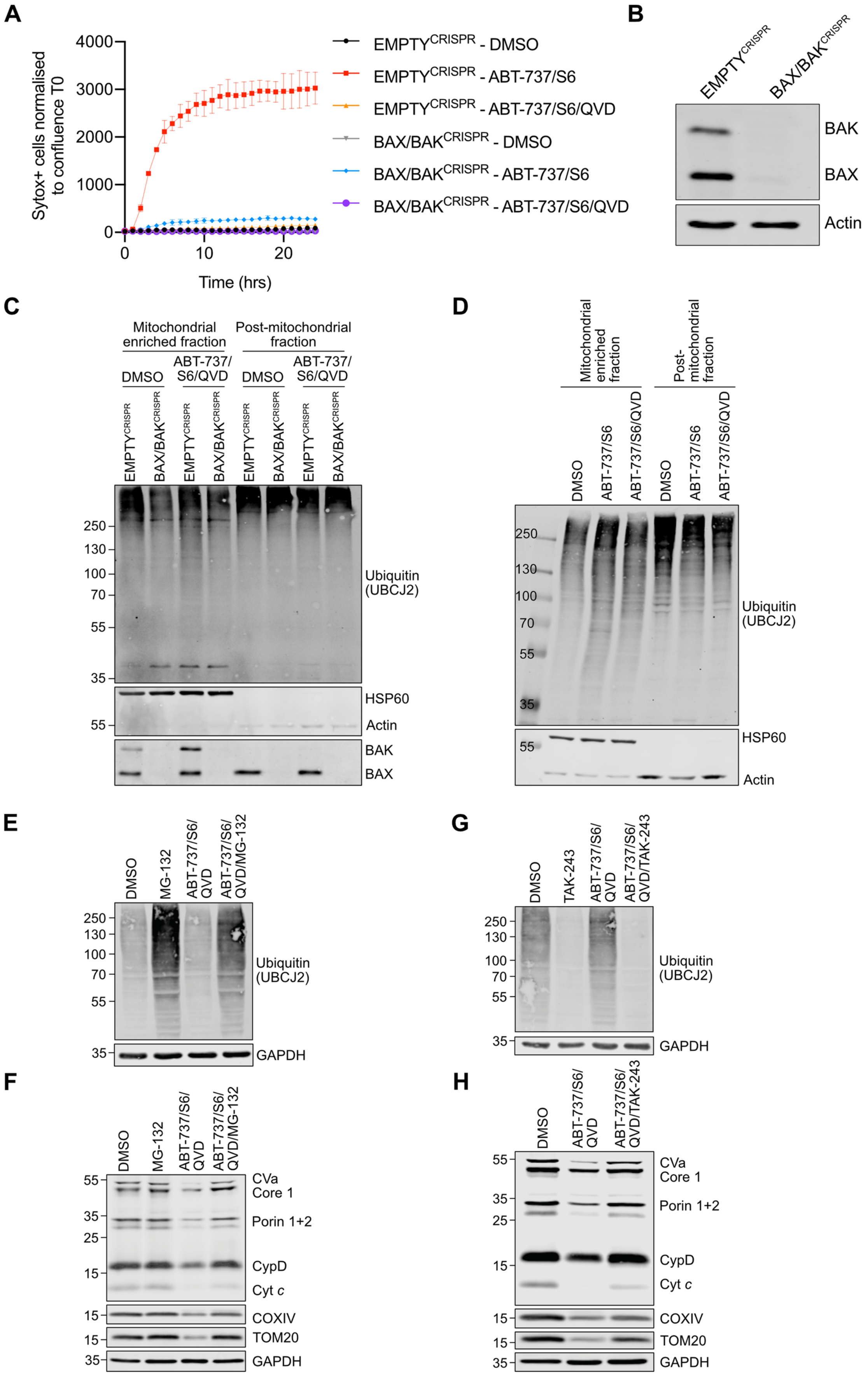
A
Sytox+ cells normalised to confluence T0
4000
3000
2000
1000
0
0
10
20
Time (hrs)
EMPTYCRISPR - DMSO
EMPTYCRISPR - ABT-737/S6
EMPTYCRISPR - ABT-737/S6/QVD
BAX/BAKCRISPR - DMSO
BAX/BAKCRISPR - ABT-737/S6
BAX/BAKCRISPR - ABT-737/S6/QVD
B
EMPTYCRISPR
BAX/BAKCRISPR
BAK
BAX
Actin
C
Mitochondrial enriched fraction
Post-mitochondrial fraction
DMSO
ABT-737/S6/QVD
EMPTYCRISPR
BAX/BAKCRISPR
250
130
100
70
55
35
Ubiquitin (UBCJ2)
HSP60
Actin
BAK
BAX
D
Mitochondrial enriched fraction
Post-mitochondrial fraction
DMSO
ABT-737/S6
ABT-737/S6/QVD
Ubiquitin (UBCJ2)
HSP60
Actin
E
DMSO
MG-132
ABT-737/S6/QVD
ABT-737/S6/QVD/MG-132
Ubiquitin (UBCJ2)
GAPDH
F
CVa
Core 1
Porin 1+2
CypD
Cyt c
COXIV
TOM20
GAPDH
G
DMSO
TAK-243
ABT-737/S6/QVD
ABT-737/S6/QVD/TAK-243
Ubiquitin (UBCJ2)
GAPDH
H
DMSO
ABT-737/S6/QVD
ABT-737/S6/QVD/TAK-243
CVa
Core 1
Porin 1+2
CypD
Cyt c
COXIV
TOM20
GAPDH

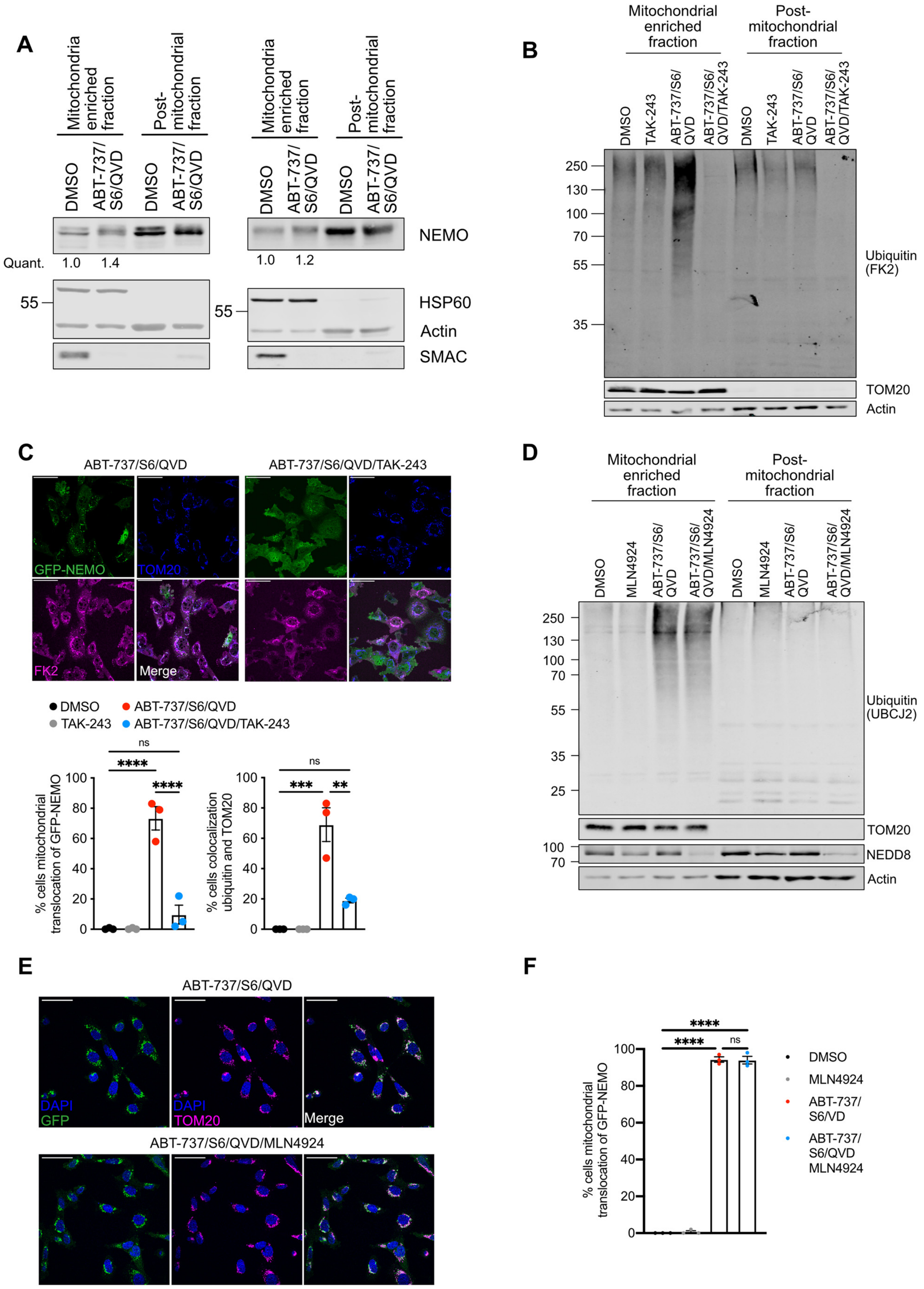
A
Mitochondria enriched fraction
Post-mitochondrial fraction
DMSO
ABT-737/S6/QVD
NEMO
Quant.
1.0
1.4
1.0
1.2
55
HSP60
Actin
SMAC
B
Mitochondrial enriched fraction
Post-mitochondrial fraction
DMSO
TAK-243
ABT-737/S6/QVD
ABT-737/S6/QVD/TAK-243
250
130
100
70
55
35
Ubiquitin (FK2)
TOM20
Actin
C
ABT-737/S6/QVD
ABT-737/S6/QVD/TAK-243
GFP-NEMO
TOM20
FK2
Merge
DMSO
ABT-737/S6/QVD
TAK-243
ABT-737/S6/QVD/TAK-243
ns
****
***
**
% cells mitochondrial translocation of GFP-NEMO
% cells colocalization ubiquitin and TOM20
100
80
60
40
20
0
D
Mitochondrial enriched fraction
Post-mitochondrial fraction
DMSO
MLN4924
ABT-737/S6/QVD
ABT-737/S6/QVD/MLN4924
250
130
100
70
55
35
25
Ubiquitin (UBCJ2)
TOM20
NEDD8
Actin
E
ABT-737/S6/QVD
DAPI
GFP
DAPI
TOM20
Merge
ABT-737/S6/QVD/MLN4924
F
****
****
ns
% cells mitochondrial translocation of GFP-NEMO
DMSO
MLN4924
ABT-737/S6/VD
ABT-737/S6/QVD MLN4924

 © The Author(s)

Figure EV2. Mitochondrial ubiquitylation and GFP-NEMO translocation can be blocked by E1 inhibition and is independent of neddylation.

(**A**) SVEC4-10 cells treated for 3 h with 10 μM ABT-737, 10 μM S63845 and 30 μM Q-VD-OPh. Mitochondria were isolated using Dounce homogeniser and cellular fractions were probed with relevant antibodies. Mitochondrial localised NEMO was quantified normalising to mitochondrial content defined by HSP60 signal. (**B**) SVEC4-10 cells pre-treated with 2 μM TAK-243 for 1 h followed by additional 1 h treatment with 10 μM ABT-737, 10 μM S63845 and 30 μM Q-VD-OPh with or without 2 μM TAK-243. Blots are representative for four independent experiments. (**C**) Upper: SVEC4-10 cells expressing GFP-NEMO were pre-treated for 1 h with 2 μM TAK-243 followed by 1 h treatment of 10 μM ABT-737, 10 μM S63845, 30 μM Q-VD-OPh with or without 2 μM TAK-243. Cells were immunostained for TOM20 and ubiquitin (FK2). Scale bar is 50 μm and images are representative for three independent experiments. Lower: quantification showing the percentage of cells with mitochondrial localised GFP-NEMO and ubiquitin puncta. (**D**) SVEC4-10 cells pre-treated with 1 μM MLN4924 (NAE inhibitor) for 1 h followed by additional 1 h treatment with 10 μM ABT-737, 10 μM S63845 and 30 μM Q-VD-OPh with or without 1 μM MLN4924. Blots are representative for two independent experiments. (**E**) SVEC4-10 cells expressing GFP-NEMO pre-treated with 1 μM MLN4924 for 1 h followed by additional 1 h treatment with 10 μM ABT-737, 10 μM S63845 and 30 μM Q-VD-OPh with or without 1 μM MLN4924. Cells were immunostained for mitochondrial TOM20 and DAPI. Images are representative for three independent experiments and are shown with a 50 μm scale bar. (**F**) Quantification of (**E**) showing the percentage of cells with mitochondrial translocation of GFP-NEMO. Data information: graphs in (**C**, **F**) display mean values ± s.e.m. (error bars) of $n = 3$ independent experiments. Statistics were performed using two-way ANOVA with Tukey correction. ****$P < 0.0001$.

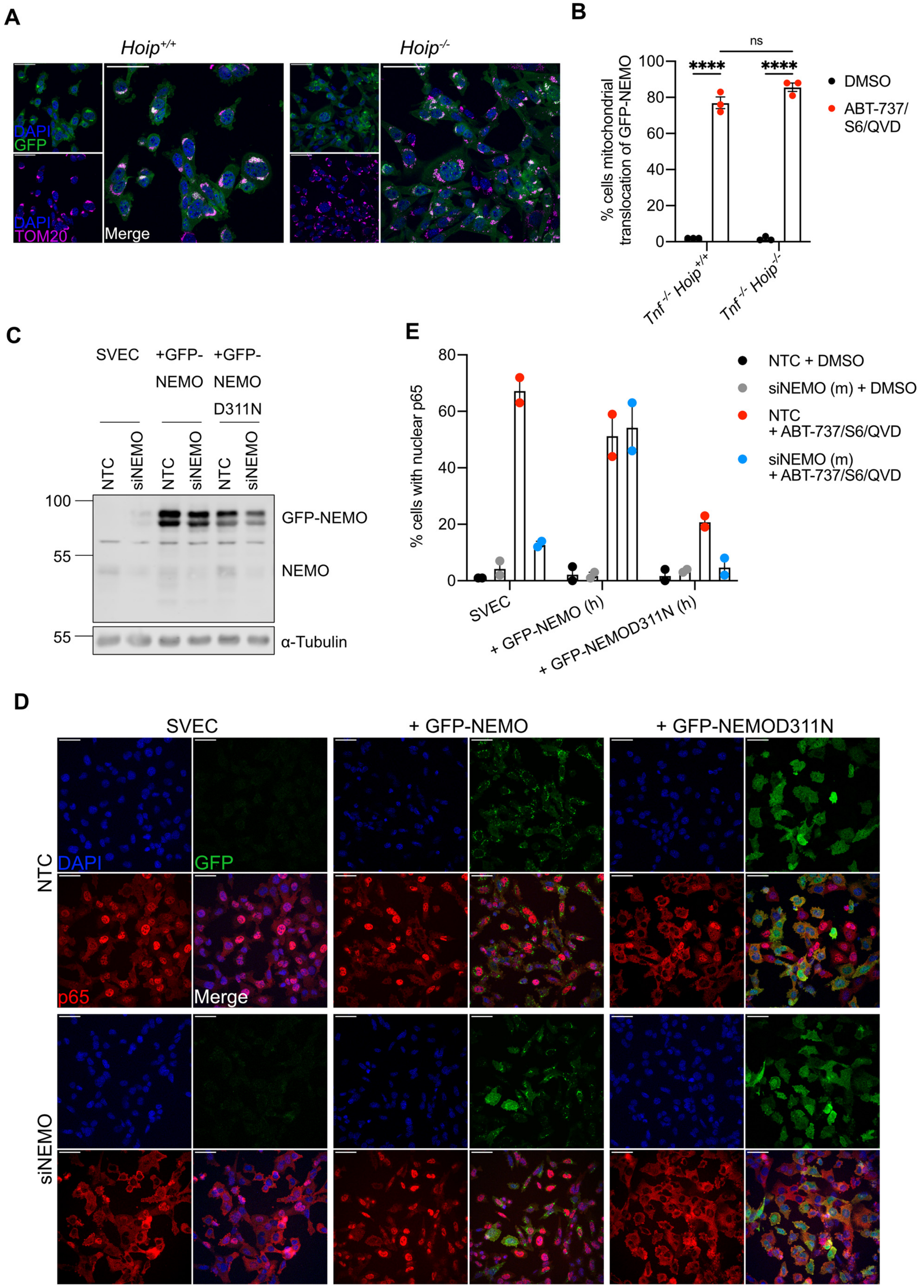
A
Hoip+/+
Hoip-/-
DAPI
GFP
DAPI
TOM20
Merge
B
ns
****
****
% cells mitochondrial translocation of GFP-NEMO
100
80
60
40
20
0
DMSO
ABT-737/ S6/QVD
Tnf-/- Hoip+/+
Tnf-/- Hoip-/-
C
SVEC
+GFP- NEMO
+GFP- NEMO D311N
NTC
siNEMO
NTC
siNEMO
NTC
siNEMO
100
55
55
GFP-NEMO
NEMO
α-Tubulin
E
% cells with nuclear p65
80
60
40
20
0
SVEC
+ GFP-NEMO (h)
+ GFP-NEMOD311N (h)
NTC + DMSO
siNEMO (m) + DMSO
NTC + ABT-737/S6/QVD
siNEMO (m) + ABT-737/S6/QVD
D
SVEC
+ GFP-NEMO
+ GFP-NEMOD311N
NTC
siNEMO
DAPI
GFP
p65
Merge

 © The Author(s)

Figure EV3. Loss of NEMO cannot be rescued during CICD by expressing non-ubiquitin-binding mutants of NEMO.

(**A**) MEF Tnf$^{-/-}$/Hoip$^{+/+}$ and Tnf$^{-/-}$/Hoip$^{-/-}$ expressing GFP-NEMO were treated for 3 h with 10 μM ABT-737, 5 μM S63845 and 30 μM Q-VD-OPh. Cells were immunostained for mitochondrial TOM20 and DAPI. Images are representative of three independent experiments. (**B**) Quantification of A showing the percentage of cells with mitochondrial translocation of GFP-NEMO. Graph displays mean values ± s.e.m. (error bars) of $n = 3$ independent experiments. Statistical analysis was performed using two-way ANOVA with Tukey correction. (**C**) Validation of SVEC4-10, SVEC4-10 GFP-NEMO and SVEC4-10 GFP-D311N cells transfected with NTC or siNEMO. Lysates were blotted for NEMO and α-tubulin. (**D**) SVEC4-10, SVEC4-10 GFP-NEMO and SVEC4-10 GFP-D311N cells transfected with NTC or siNEMO were treated for 1 h with 10 μM ABT-737, 10 μM S63845 and 30 μM Q-VD-OPh. Cells were immunostained for p65 and DAPI. Images are representative of two independent experiments. (**E**) Quantification of (**D**) showing the percentage of cells with nuclear translocation of p65. Graph displays mean values ± s.e.m. (error bars) of $n = 2$ independent experiments. ****$P < 0.0001$.

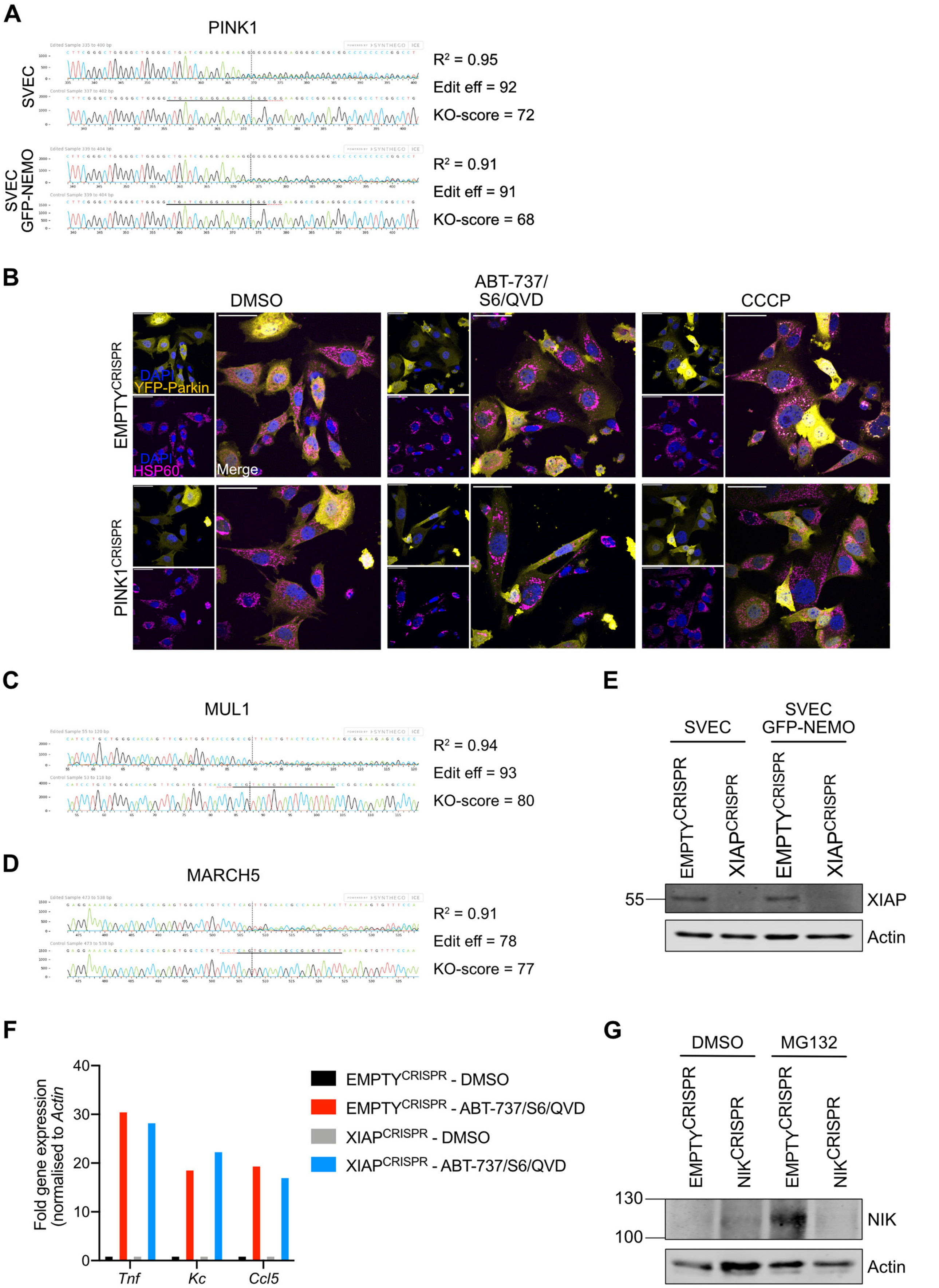
A
PINK1
SVEC
SVEC GFP-NEMO
R² = 0.95
Edit eff = 92
KO-score = 72
R² = 0.91
Edit eff = 91
KO-score = 68
B
DMSO
ABT-737/ S6/QVD
CCCP
EMPTYCRISPR
PINK1CRISPR
DAPI
YFP-Parkin
DAPI
HSP60
Merge
C
MUL1
R² = 0.94
Edit eff = 93
KO-score = 80
D
MARCH5
R² = 0.91
Edit eff = 78
KO-score = 77
E
SVEC
SVEC GFP-NEMO
EMPTYCRISPR
XIAPCRISPR
EMPTYCRISPR
XIAPCRISPR
55
XIAP
Actin
F
Fold gene expression (normalised to Actin)
40
30
20
10
0
Tnf
Kc
Ccl5
EMPTYCRISPR - DMSO
EMPTYCRISPR - ABT-737/S6/QVD
XIAPCRISPR - DMSO
XIAPCRISPR - ABT-737/S6/QVD
G
DMSO
MG132
EMPTYCRISPR
NIKCRISPR
EMPTYCRISPR
NIKCRISPR
130
100
NIK
Actin

Figure EV4. Validation of PINK1^{CRISPR}, NIK^{CRISPR}, MUL1/MARCH5^{CRISPR} and XIAP^{CRISPR} knockout cell lines.

(**A**) Validation of PINK1 knockout in SVEC4-10 cells with or without GFP-NEMO expression using genomic PCR and ICE (inference of CRISPR edits) analysis. (**B**) SVEC4-10 EMPTY^{CRISPR} and PINK1^{CRISPR} cells expressing YFP-Parkin were treated for 1 h with 10 μM ABT-737, 10 μM S63845 and 30 μM Q-VD-OPh or for 3 h with 10 μM CCCP. Mitochondria were immunostained with HSP60 and DAPI. Images are representative of two independent experiments and displayed with 50 μm scale bar. (**C**) Validation of MUL1 knockout in SVEC4-10 MUL1/MARCH5^{CRISPR} cells using genomic PCR and ICE analysis. (**D**) Validation of MARCH5 knockout in SVEC4-10 MUL1/MARCH5^{CRISPR} cells using genomic PCR and ICE analysis. (**E**) Validation of SVEC4-10 XIAP^{CRISPR} cells with and without GFP-NEMO expression using western blot. Lysates were blotted for XIAP and Actin. (**F**) *Tnf*, *Kc*, and *Ccl5* expression of SVEC4-10 EMPTY^{CRISPR} and XIAP^{CRISPR} cells treated with 10 μM ABT-737, 10 μM S63845 and 30 μM Q-VD for 3 h. Graph is representative of three independent experiments. (**G**) Validation of NIK knockout in GFP-NEMO expressing SVEC4-10 EMPTY^{CRISPR} and NIK^{CRISPR} cells. Cells were treated for 2 h with 10 μM MG-132. Lysates were blotted for NIK and actin.

© The Author(s)

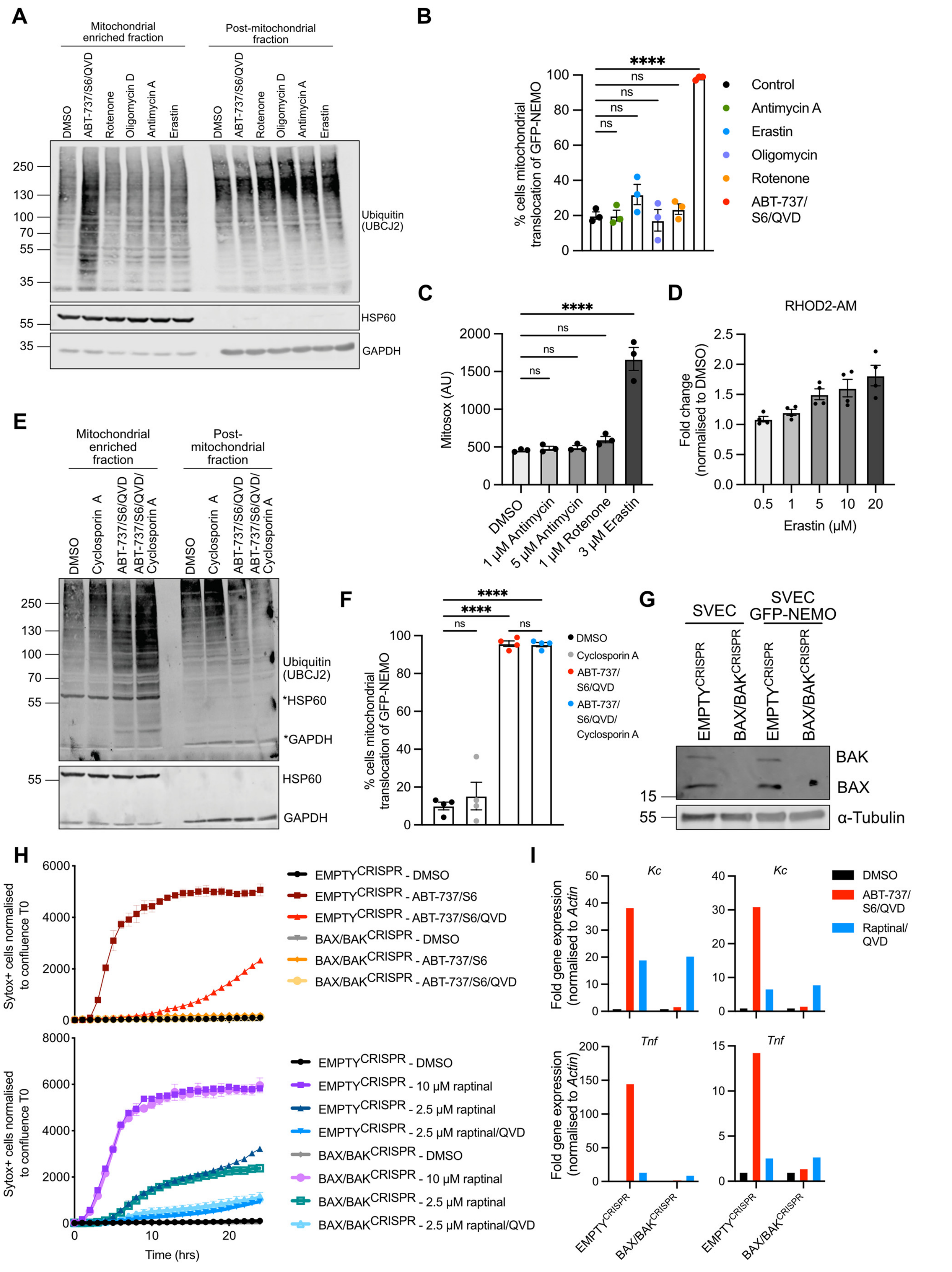
A
Mitochondrial enriched fraction
Post-mitochondrial fraction
DMSO
ABT-737/S6/QVD
Rotenone
Oligomycin D
Antimycin A
Erastin
Ubiquitin (UBCJ2)
HSP60
GAPDH
B
% cells mitochondrial translocation of GFP-NEMO
Control
Antimycin A
Erastin
Oligomycin
Rotenone
ABT-737/ S6/QVD
C
Mitosox (AU)
DMSO
1 µM Antimycin
5 µM Antimycin
1 µM Rotenone
3 µM Erastin
D
RHOD2-AM
Fold change (normalised to DMSO)
Erastin (µM)
E
Cyclosporin A
ABT-737/S6/QVD/ Cyclosporin A
*HSP60
*GAPDH
F
G
SVEC
SVEC GFP-NEMO
EMPTYCRISPR
BAX/BAKCRISPR
BAK
BAX
α-Tubulin
H
Sytox+ cells normalised to confluence T0
EMPTYCRISPR - DMSO
EMPTYCRISPR - ABT-737/S6
EMPTYCRISPR - ABT-737/S6/QVD
BAX/BAKCRISPR - DMSO
BAX/BAKCRISPR - ABT-737/S6
BAX/BAKCRISPR - ABT-737/S6/QVD
EMPTYCRISPR - 10 µM raptinal
EMPTYCRISPR - 2.5 µM raptinal
EMPTYCRISPR - 2.5 µM raptinal/QVD
BAX/BAKCRISPR - 10 µM raptinal
BAX/BAKCRISPR - 2.5 µM raptinal
BAX/BAKCRISPR - 2.5 µM raptinal/QVD
Time (hrs)
I
Kc
Tnf
Fold gene expression (normalised to Actin)
DMSO
ABT-737/ S6/QVD
Raptinal/ QVD

 © The Author(s)

Figure EV5. Raptinal induces cell death independent of mitochondrial permeabilization by BAX and BAK.

(**A**) SVEC4-10 cells were treated for 2 h with 1 μM rotenone, 1 μM oligomycin, 5 μM antimycin A, 3 μM erastin or the combination 10 μM ABT-737, 10 μM S63845 and 30 μM Q-VD-OPh. Mitochondria were isolated using digitonin fractionation buffer and antibodies against ubiquitin (UBCJ2), HSP60 and GAPDH were used. (**B**) SVEC4-10 cells expressing GFP-NEMO were treated for 2 h with 1 μM rotenone, 1 μM oligomycin, 5 μM antimycin A, 3 μM erastin or the combination 10 μM ABT-737, 10 μM S63845 and 30 μM Q-VD-OPh. Cells were immunostained with mitochondrial TOM20 and DAPI for confocal microscopy. Graph shows the quantification of three independent experiments in which the percentage of cells with mitochondrial localisation of GFP-NEMO was analysed. (**C**) SVEC4-10 cells were treated for 2 h with 1 μM rotenone, 1 and 5 μM antimycin A or 3 μM erastin. ROS levels were determined using MitoSOX Red via flow cytometry. Graph displays mean values ± s.e.m. (error bars) of $n = 3$ independent experiments. (**D**) SVEC4-10 cells were treated with 0.5, 1, 5, 10 and 20 μM erastin. Mitochondrial calcium was measured using Rhod2-AM via flow cytometry. Graph displays mean values ± s.e.m. (error bars) of $n = 3$ independent experiments. (**E**) SVEC4-10 cells were treated for 2 h with 10 μM ABT-737, 10 μM S63845 and 30 μM Q-VD-OPh with or without 25 μM cyclosporin A. Mitochondria were isolated using digitonin fractionation buffer and antibodies against ubiquitin (UBCJ2), HSP60 and GADPH were used. (**F**) SVEC4-10 cells expressing GFP-NEMO were treated for 2 h with 10 μM ABT-737, 10 μM S63845 and 30 μM Q-VD-OPh with or without 25 μM cyclosporin A. Cells were immunostained with mitochondrial TOM20 and DAPI for confocal microscopy. Graph shows the quantification of three independent experiments showing the percentage of cells with mitochondrial localisation of GFP-NEMO, error bars represent s.e.m. (**G**) EMPTY^{CRISPR} and BAX/BAK^{CRISPR} validation of SVEC4-10 cells and SVEC4-10 cells expressing GFP-NEMO. Lysates for blotted for BAX, BAK and α-tubulin. (**H**) SVEC4-10 EMPTY^{CRISPR} and SVEC4-10 BAX/BAK^{CRISPR} cells treated with 10 μM ABT-737 and 10 μM S63845 or treated with 2.5 or 10 μM raptinal. Caspase-dependency of death was assessed using 30 μM Q-VD-OPh. Cell viability was measured using Sytox Green exclusion. Graphs are representative of two independent experiments and display the mean and s.e.m. of two replicates. (**I**) SVEC4-10 cells treated for 3 h with 10 μM ABT-737, 10 μM S63856 and 30 μM Q-VD-OPh or 2.5 μM raptinal and 30 μM Q-VD-OPh. Expression of *Kc*, *Tnf* and *Actin* were validated using RT-qPCR. Two repeats of Fig. 6E. Data information: (**A**, **E**) blots are representative for three independent experiments. Statistics performed using two-way ANOVA with Tukey correction. ***$P < 0.001$, ****$P < 0.0001$.
